# Redox-enabled direct stereoconvergent heteroarylation of simple alcohols

**DOI:** 10.1038/s41467-021-25268-1

**Published:** 2021-08-19

**Authors:** Yongbing Liu, Ran Tao, Zhi-Keng Lin, Guoqiang Yang, Yu Zhao

**Affiliations:** 1 Department of Chemistry, National University of Singapore, Singapore, Republic of Singapore; 2 Joint School of National University of Singapore and Tianjin University, International Campus of Tianjin University, Binhai New City, Fuzhou, China

**Keywords:** Asymmetric catalysis, Synthetic chemistry methodology

## Abstract

The direct transformation of racemic feedstock materials to valuable enantiopure compounds is of significant importance for sustainable chemical synthesis. Toward this goal, the radical mechanism has proven uniquely effective in stereoconvergent carbon-carbon bond forming reactions. Here we report a mechanistically distinct redox-enabled strategy for an efficient enantioconvergent coupling of pyrroles with simple racemic secondary alcohols. In such processes, chirality is removed from the substrate via dehydrogenation and reinstalled in the catalytic reduction of a key stabilized cationic intermediate. This strategy provides significant advantage of utilizing simple pyrroles to react with feedstock alcohols without the need for leaving group incorporation. This overall redox-neutral transformation is also highly economical with no additional reagent nor waste generation other than water. In our studies, oxime-derived iridacycle complexes are introduced, which cooperate with a chiral phosphoric acid to enable heteroarylation of alcohols, accessing a wide range of valuable substituted pyrroles in high yield and enantioselectivity.

## Abstract (web summary)

Synthesizing complex structures of high enantiomeric excess from racemic feedstock is an enduring challenge. Here, the authors couple racemic secondary alcohols with pyrroles to form enantioenriched 2-substituted heteroarenes, via a borrowing hydrogen mechanism using the combination of an iridium catalyst and chiral phosphoric acid.

## Introduction

Effective and stereoselective construction of carbon−carbon bonds remains a central theme in chemical synthesis with wide applications in medicinal and material sciences. With a strong push towards economy and sustainability in modern chemical synthesis^1^, the direct incorporation of renewable feedstock materials in enantioselective carbon−carbon bond formation has attracted much research interest. As a large number of feedstock materials from nature or the petrochemical industry (e.g., alcohols, carboxylic acids) are racemic, how to convert them to value-added enantiopure compounds in high yield has been pursued as a holy grail in catalysis and synthesis^2^. Mechanistically, achieving such enantioconvergent transformations necessitates the removal of chirality from the substrate, which is typically realized by the formation of achiral ionic intermediates. The classical nucleophilic substitution approach^3^, which requires the generation of stabilized carbocation/carbanion intermediates for effective chirality control, is limited to the specially substituted or activated substrates and not applicable towards the reaction of non-functionalized feedstock materials^4–9^.

**Fig. 1:**
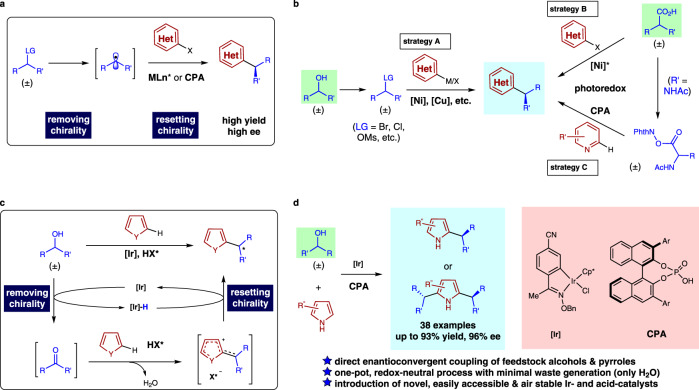
Redox- vs. radical-based enantioconvergent (hetero)arylation. **a** General mechanism of radical-based enantioconvergent (hetero)arylation. **b** Different strategies achieved for enantioconvergent (hetero)arylation using the radical mechanism. **c** The working hypothesis of redox-enabled enantioconvergent heteroarylation of feedstock secondary alcohols. **d** This work: enantioconvergent reaction of feedstock secondary alcohols with pyrroles catalyzed by oxime-derived iridacycle and chiral phosphoric acid (**CPA**) catalysts.

An important breakthrough in this field of research came from catalytic systems involving a radical intermediate, which enabled enantioconvergent C−C bond formation from simple, racemic alkyl precursors^10,11^. The general mechanism of sp^2^−sp^3^ type cross-coupling reactions leading to effective, enantioconvergent (hetero)arene functionalization is illustrated in Fig. 1a, the products of which are ubiquitous structural features in pharmaceuticals and agrichemicals. The Fu group and others developed a series of highly efficient, enantioconvergent base metal-catalyzed cross-coupling reactions to access a wide range of chiral products from racemic alkyl halides or mesylates (Strategy A, Fig. 1b)^12,13^. Enantioselective reductive cross-coupling of two readily available electrophiles has also been achieved by the Reisman group and others^14^. In an alternative approach, the combination of photoredox and nickel catalysis was introduced by the Molander group, the MacMillan group, and others, resulting in highly efficient, enantioconvergent coupling of racemic organotrifluoroborates^15^ or carboxylic acids with (hetero)aryl halides (Strategy B, Fig. 1b)^16^. For the above strategies, it is noteworthy that pre-functionalization of both cross partners (use of alkyl halides and haloarenes instead of the feedstock alcohols and simple arenes) was needed. More recently, the Phipps group and later the Fu group introduced an elegant approach combining photoredox and chiral phosphoric acid catalysis, which achieved direct asymmetric functionalization of pyridines, although the MPHI esters were used instead of the corresponding simple carboxylic acids (Strategy C, Fig. 1b)^17,18^.

In an effort to achieve truly practical enantioconvergent C−C bond formation employing all feedstock materials, we considered the redox process as a distinct racemization mechanism to allow more step- and atom-economical heteroarene functionalization. As shown by our hypothesis in Fig. 1c, readily available feedstock alcohols, instead of the corresponding organohalides, are adopted as the substrate to achieve direct heteroarylation with privileged heteroarenes such as pyrroles. The postulated dehydrogenation of simple secondary alcohols by the metal catalyst realizes the removal of chirality from the racemic substrate. This is then followed by heteroarene addition to the resultant ketone and dehydration to generate a stabilized cationic intermediate, to which catalyst-controlled stereoselective hydride transfer takes place to deliver enantioenriched substituted heteroarenes. This cascade process couples two commercially available, non-activated starting materials to deliver versatile, valuable products, is overall redox-neutral, and produces no side product other than water.

The above catalytic cascade proceeds through a borrowing hydrogen mechanism to achieve alcohol substitution, which has been widely recognized as an attractive strategy for green chemical synthesis^19–21^. The most explored transformations in this area of research include the alkylation of amines or ketone enolates using alcohols, with a range of non-stereoselective systems reported in the literature. Enantioselective variants of these transformations, on the other hand, have remained underdeveloped^22,23^. Highly enantioselective alkylation of amines and ketones has only been achieved in recent years by our group^24–28^, the Beller group^29^, the Donohoe group^30,31^, and others^32–37^ to access chiral amines, ketones, alcohols, etc. The application of borrowing hydrogen strategy to an enantioconvergent heteroarylation, to our knowledge, has never been reported in the literature^38^. In sharp contrast, the Krische group has developed an alternative hydrogen auto-transfer process for the achievement of highly powerful and versatile stereoselective lower-to-higher-alcohol conversions via carbonyl addition^39,40^. The successful realization of direct, stereoconvergent heteroarylation of alcohols will provide a general toolbox for accessing valuable enantiopure heteroarenes bearing diverse alkyl substituents. Compared to the recent elegant and atom-economical approach of C−H alkylation of arenes using alkene reagents^41–43^, our strategy aims for intermolecular coupling of two feedstock substrates without the use of directing group and is also mechanistically intriguing as an enantioconvergent process.

Herein, we report our development of catalytic enantioconvergent heteroarylation of readily available, unactivated secondary alcohols with pyrroles (Fig. 1d). Under the cooperative catalysis of an oxime-derived iridacycle complex and chiral phosphoric acid, enantioconvergent heteroarylation of simple secondary alcohols with pyrroles are achieved to deliver a wide range of valuable substituted heteroarenes in high yields and enantioselectivities.

## Results and discussion

### Catalyst development for enantioconvergent heteroarylation of alcohols with pyrroles

**Fig. 2:**
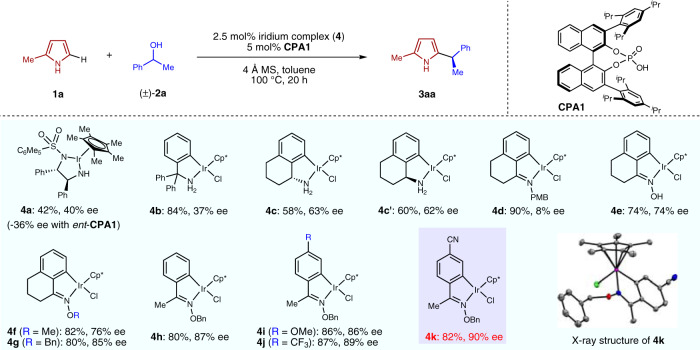
Catalyst development for enantioconvergent heteroarylation of alcohols with pyrroles. Reaction conditions. **1a** (0.20 mmol), **2a** (0.40 mmol), **4** (0.005 mmol), **CPA1** (0.01 mmol) and 4 Å MS (20 mg) in toluene (0.5 mL) at 100 °C under N_2_ for 20 h.

Pyrroles are very important heterocyclic structures in pharmaceutical research and material science. The development of efficient methods for preparing pyrrole derivatives is of great interest in the chemical community^44^. We decided to initiate our investigation with the enantioconvergent heteroarylation of commercially available, simple alcohols such as (±)-1-phenylethanol **2a** with simple pyrrole **1a**. As shown in Fig. 2, early studies using the dual catalytic system of a chiral iridium complex (**4a**) and chiral phosphoric acid **CPA1**^24^ only provided the desired substituted pyrrole **3aa** in a moderate yield (42%) and enantioselectivity (40% ee). The use of the diastereomeric pair of **4a** and *ent*-**CPA1** showed that the enantioselectivity for **3aa** formation (−36% ee) was almost entirely determined by the chiral phosphoric acid. We then turned our attention to the use of another family of iridacycle complexes such as **4b**^26^, which yielded **3aa** in higher yield with a similar level of enantioselectivity. Further catalyst modifications led to the discovery of **4c** derived from a bicyclic chiral amine as a better choice, which yielded **3aa** in an improved 63% ee. When catalyst **4c′** derived from the enantiomeric chiral amine was examined in combination with **CPA1**, essentially the same result was observed for **3aa**, indicating once more that CPA serves as the determining factor for asymmetric induction. Since the chirality from the iridium complexes seemed to play a minimal role in this transformation, we decided to further explore iridacycle catalysts that keep the tricyclic skeleton but do not necessarily possess any chirality. Imine-derived iridacycle **4d**^45,46^ was examined next, which yielded **3aa** in an excellent yield but unfortunately with low enantioselectivity. Oxime-derived iridium complexes that have rarely been utilized were also prepared and tested for our reaction. To our pleasure, complexes **4e**−**4g** proved to be active catalysts for this transformation, with the enantioselectivity gradually increased by the use of bulkier oxime substituents and reached a promising 85% ee in the presence of **4g**. At this stage, further variations on this series of iridium complexes were examined and we were happy to observe similar efficiency and selectivity with **4h**, which is easily accessible from simple acetophenone. This allowed an efficient synthesis of various analogous complexes starting from commercially available substituted acetophenones. Finally, complex **4k** bearing a cyano substituent proved to be the optimal choice among **4i**−**4k**, which delivered **3aa** in a high yield of 82% with an excellent 90% ee. It is noteworthy that all these iridium chloride complexes are air-stable and easy to use, and can be easily prepared via simple operations.

### Substrate scope for enantioconvergent heteroarylation of alcohols with pyrroles

**Fig. 3:**
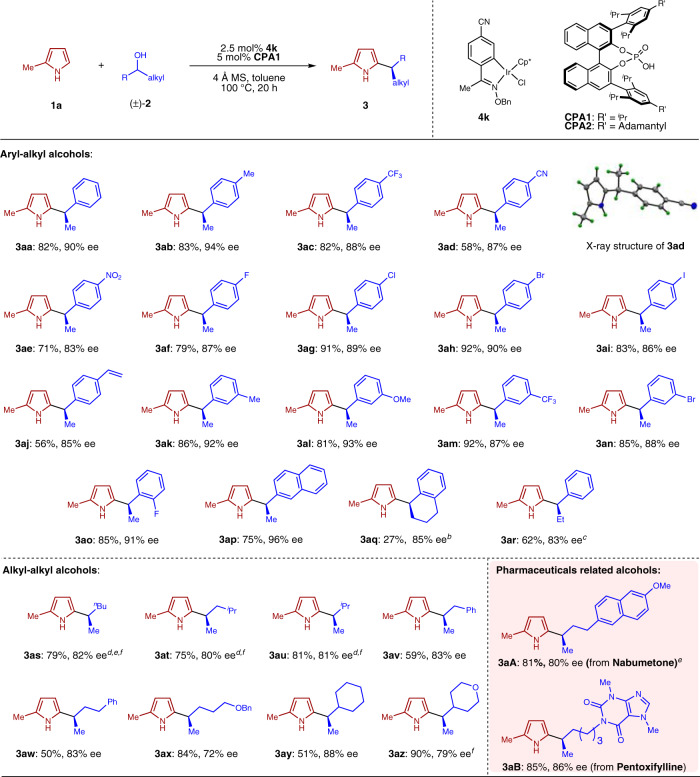
Scope of alcohols for enantioconvergent heteroarylation using pyrrole **1a**^*a*^. ^*a*^Reaction conditions: **1a** (0.20 mmol), **2a** (0.40 mmol), **4** (0.005 mmol), **CPA1** (0.01 mmol) and 4 Å MS (20 mg) in toluene (0.5 mL) at 100 °C under N_2_ for 20 h, unless otherwise noted. See supplementary information for details. ^*b*^Reaction performed at 130 °C. ^*c*^Reaction performed with 3 equiv. of alcohol. ^*d*^Reaction performed with **CPA2** instead of **CPA1**. ^*e*^Reaction performed at 90 °C. ^*f*^Reaction performed with 4 mol% **4k** and 8 mol% **CPA**.

With the optimal conditions in hand, we moved on to explore the scope of this catalytic system. As shown in Fig. 3, a wide range of secondary alcohols were examined first, starting with racemic secondary aryl−alkyl alcohols. Various *para*-, *meta*- and *ortho*-substituents on the aryl rings, either electron-withdrawing or donating, could be well-tolerated to deliver products **3ab**−**3ao** in good to excellent levels of enantioselectivity. In addition to alkyl and halogen substituents, it is noteworthy that cyano and nitro groups were compatible in our system to produce **3ad** and **3ae** in good yields, which is rare in related systems involving a metal hydride reduction. Interestingly, the alcohol-containing a vinyl substitution underwent substitution to yield **3aj** in 56% yield with a high 85% ee, although partial over-reduction of the vinyl group was observed to some extent. In addition, the reaction with 1-(2-naphthyl)ethanol produced **3ap** in a good yield of 75% and an excellent 96% ee. As an important extension, the reaction was not limited to alcohols bearing a methyl substituent. A bicyclic alcohol also participated in the reaction to yield **3aq** in good enantioselectivity, albeit in a low yield. Product **3ar** bearing an ethyl group was also obtained in good level of enantioselectivity. Single crystal X-ray analysis of **3ad** managed to establish the absolute configuration of this series of substituted pyrroles.

In addition to the above examples using benzylic alcohols, we were excited to observe that even simple aliphatic secondary alcohols worked well using this catalytic procedure to produce pyrroles **3as**−**3az** in good yields with good to high enantioselectivities. The differentiation of a methyl unit with either a branched or linear alkyl chain turned out to be equally effective. It is important to note that this class of unactivated aliphatic alcohols would be extremely challenging substrates for enantioconvergent nucleophilic substitution through the classical S_N_1 mechanism.

To further showcase the scope and utility of our system, we decided to explore the functionalization of alcohols bearing a more complicated structure and especially those that are derived from commercial drugs. Two representative examples were demonstrated in Fig. 3: alcohols derived from Nabumetone and Pentoxifylline were converted smoothly to the corresponding enantioenriched **3aA** and **3aB** in high efficiency and selectivity.

**Fig. 4:**
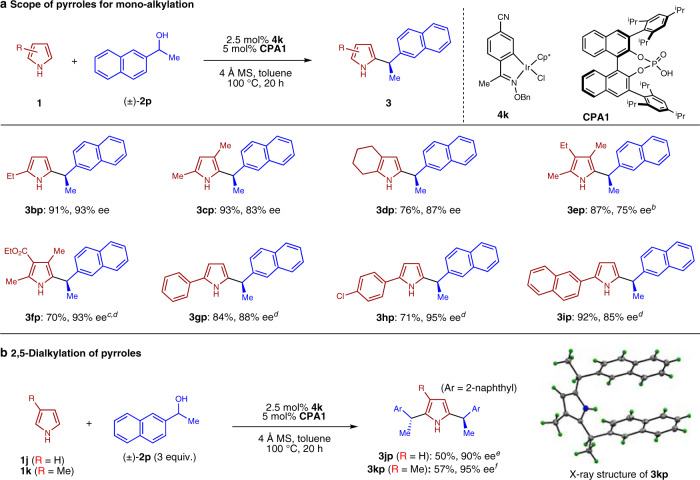
Scope of pyrroles for enantioconvergent heteroarylation using alcohol **2p**^***a***^. **a** Scope of pyrroles for enantioconvergent heteroarylation of **2p**. **b** 2,5-Dialkylation of pyrroles. ^*a*^Reaction conditions: **1a** (0.20 mmol), **2a** (0.40 mmol), **4** (0.005 mmol), **CPA1** (0.01 mmol) and 4 Å MS (20 mg) in toluene (0.5 mL) at 100 °C under N_2_ for 20 h, unless otherwise noted. See supplementary information for details. ^*b*^Reaction performed at 80 °C. ^*c*^Reaction performed at 130 °C. ^*d*^Reaction performed with 5 mol% **4k** and 10 mol% **CPA1**. ^*e*^Reaction performed with 4 mol% **4k** and 8 mol% **CPA**. ^*f*^Reaction performed at 90 °C.

With a wide range of alcohols explored for this enantioconvergent heteroarylation, we then turned our attention to examine the scope of various substituted pyrroles, using 1-(2-naphthyl)ethanol **2p** as the alkylating reagent. As shown in Fig. 4a, mono-, di- as well as trialkyl substituted pyrroles underwent alkylation smoothly to produce **3bp**−**3ep** in good yields and high enantioselectivities. For pyrrole **1f** bearing an electron-withdrawing ester substituent, an elevated temperature of 130 °C and higher catalyst loading were needed to yield **3fp** in good yield. Even under such harsh conditions excellent level of enantioselectivity was obtained. Our catalytic system was not limited to alkyl-substituted pyrroles. 2-Arylpyrroles proved to be suitable substrates as well, and the alkylated products (**3gp**−**3ip**) were obtained in high yields and enantioselectivities.

All the above examples focused on mono-alkylation of pyrroles bearing one *ortho*-substituent. To further diversify our catalytic system, we also explored the di-substitution of pyrroles without such substituents. As shown in Fig. 4b, representative reactions of **1j** and **1k** with alcohol **2p** proceeded smoothly under similar catalytic conditions, providing **3jp** and **3kp** as the major products in excellent enantiopurity. The corresponding *syn*-diastereomers were also formed in a small amount (see supplementary information for details). It is interesting to note that this represents another example of the so-called “Horeau Principle”^47^, which involves amplification of enantioselectivity for the major diastereomer formation in a double enantioselective transformation by converting the minor enantiomer of the monoadduct predominantly to the meso-diastereomer^48,49^. The synthesis of doubly substituted pyrroles also greatly expanded the synthetic utility of our catalytic enantioconvergent heteroarylation.

### Mechanistic studies for enantioconvergent heteroarylation of alcohols with pyrroles

Mechanistic studies were performed to shed some light on the reaction pathway of this catalytic system. Compared to previous reports of heteroarene substitution using activated alcohols through a S_N_1 pathway that was solely catalyzed by chiral phosphoric acid, the iridium catalyst in our system was believed to be essential for the redox chemistry to engage the unactivated alcohols. Key control experiments were carried out first to confirm the effect of iridium catalyst.

**Fig. 5:**
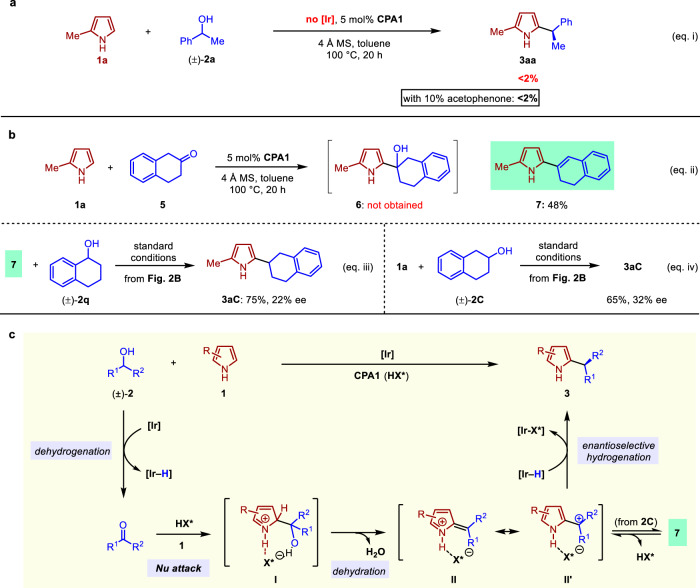
Mechanistic aspects of enantioconvergent heteroarylation of alcohols. **a** Confirmation of the role of iridium catalyst for enantioconvergent heteroarylation of alcohols. **b** Isolation and investigation of **9** as an off-cycle isomer of the key carbocation intermediate for enantioconvergent heteroarylation of alcohols. **c** Proposed catalytic pathway for enantioconvergent heteroarylation of alcohols with pyrroles.

As shown in Fig. 5a, for enantioconvergent heteroarylation of alcohol **2a** using pyrrole **1a** (eq. i), no desired product was observed at all in the absence of iridium catalyst under otherwise identical conditions with **CPA1** as the sole catalyst. This ruled out a simple acid-catalyzed intermolecular S_N_1 substitution pathway. We also noted that a non-stereoselective heteroarylation of alcohols catalyzed by Brønsted acid was reported, in which the essential additive of acetophenone served as an effective initiator to promote a redox chain reaction^50^. To rule out this possibility for our system, we carried out the reaction in the absence of iridium catalyst but with the addition of 10 mol% acetophenone together with 5 mol% **CPA1**. This set of conditions led to no conversion to **3aa** at all either. These observations were consistent with our hypothesis that our reaction goes through a redox pathway under the cooperative catalysis of iridium and chiral phosphoric acid.

We then spent much effort trying to capture the important intermediates in this catalytic transformation. The attempted direct observation of the key carbocation intermediate under the catalytic conditions proved to be futile with much effort. Notably, a significant amount of ketone intermediate could be observed, which is believed to be the product of Ir-catalyzed alcohol dehydrogenation. We then focused on the following steps by studying the separate reactions between pyrrole **1a** and various ketones including acetophenone, 2-acetonaphthone, phenylacetone, and 2-tetralone, etc. Intriguingly, when we carried out the reaction of **1a** with 2-tetralone **5** using **CPA1** as the catalyst (eq. ii, Fig. 5b), the direct product of pyrrole addition to ketone, i.e., tertiary alcohol **6** was not observed in this reaction, presumably due to its rapid dehydration. On the other hand, we were delighted to isolate alkenyl pyrrole **7** in a 48% yield, which was believed to be one form of the dehydration product from **6** and likely the deprotonation product of the key carbocation/conjugated iminium intermediate we tried to identify.

To provide support for **7** as an off-cycle species related to the key intermediate in the catalytic cycle, we subjected it to catalytic transfer hydrogenation conditions using the same Ir/CPA catalysts with **2q** as the hydrogen donor. Indeed, the pyrrole alkylation product **3aC** was obtained in 75% yield with 22% ee (eq. iii, Fig. 5b). As a key control experiment, catalytic redox-neutral alkylation of **1a** with **2C**, the alcohol corresponding to **5**, under standard conditions led to the formation of **3aC** in comparable yield and 32% ee (eq. iv, Fig. 5b). The low enantioselectivity for this substrate was not surprising considering the minimal size difference of the alcohol substituents. These results provided strong support that **7** could re-enter the redox cycle through equilibrium with the formal carbocation intermediate (shown by **II** or **II′** in Fig. 5c) in the borrowing hydrogen mechanism for enantioconvergent heteroarylation of alcohols.

Based on the above evidence, a plausible mechanism for this catalytic enantioconvergent heteroarylation of simple alcohols with pyrroles is proposed in Fig. 5c. Iridium-catalyzed dehydrogenation of racemic alcohol **2** provides the ketone and iridium hydride species. This represents the stereoablative step in this catalytic cascade. Acid-promoted nucleophilic addition of pyrrole to ketone then follows to yield an acid-bound tertiary alcohol **I**. Dehydration of **I** can proceed smoothly to produce the conjugated iminium intermediate **II**, which is in resonance form with carbocation **II′** paired with the chiral phosphate. Finally, enantioselective hydride transfer from iridium hydride to **II/II′** delivers the enantioenriched product and regenerates the catalysts. The chiral induction of hydride transfer comes from the chiral phosphate presumably through ion-pair and hydrogen bonding interactions in **II** or **II′**, which was nicely demonstrated in CPA and iridium co-catalyzed asymmetric hydrogenation of quinolines involving a 1,4-hydride addition step^51^.

In conclusion, we have developed a direct, enantioconvergent coupling of unactivated racemic alcohols with pyrroles by the introduction of oxime-derived iridacycle and chiral phosphoric acid catalytic systems. The innovative operating mechanism through borrowing hydrogen enabled the access to a stabilized ionic intermediate from simple alcohol and heteroarenes substrates and led to a general, enantioconvergent heteroarylation of unactivated secondary alcohols using pyrroles. The enantioconvergent coupling of other families of heteroarenes with alcohols is under investigation in our laboratory and will be reported in due course.

## Methods

### Representative procedure for enantioconvergent heteroarylation of alcohols

In a nitrogen-filled glove box, an 8 mL vial was charged with iridium complex (**4k**, 3.1 mg, 0.0050 mmol), **CPA1** (7.5 mg, 0.010 mmol), 4 Å molecular sieves (20 mg), 2-methylpyrrole (**1a**, 16.2 mg, 0.200 mmol) and 1-phenylethanol (**2a**, 48.9 mg, 0.400 mmol) and toluene (0.5 mL). The reaction tube was then sealed, taken outside the glovebox, heated to 100 °C, and allowed to stir for 20 h. The resulting mixture was cooled to room temperature and then purified by column chromatography (silica gel, hexanes/Et_2_O/Et_3_N = 15/1/0.15) to provide the desired alkylated product 2-methyl-5-(1-phenylethyl)-1H-pyrrole (**3aa**) as a colorless oil. For other related products, hexanes/Et_2_O/Et_3_N = 20/1/0.2 – 2/1/0.03 or hexanes/CH_2_Cl_2_/Et_3_N = 5/1/0.05 – 3/1/0.03 were used as the eluents for purification.

## Supplementary information

Supplementary Information

## Data Availability

Experimental details, characterization of compounds, and copies of NMR data are available with the submitted paper. The X-ray crystallographic coordinates for structures reported in this study have been deposited at the Cambridge Crystallographic Data Center (CCDC), under deposition numbers 2018968, 2018969, and 2018974. These data can be obtained free of charge from The Cambridge Crystallographic Data Center via www.ccdc.cam.ac.uk/data_request/cif.
