# Supplementary material for: Redox-enabled direct stereoconvergent heteroarylation of simple alcohols
Source: Nat Commun. 2021 Aug 19;12:5035. doi: 10.1038/s41467-021-25268-1 (PMC8376995; doi:10.1038/s41467-021-25268-1)
Supplement: Supplementary file 1 — Supplementary Information [file 41467_2021_25268_MOESM1_ESM.pdf]

Redox-Enabled Direct Stereoconvergent Heteroarylation of Simple Alcohols

Liu *et al.*

Supplementary Methods

1. General Information

Chemicals and solvents were purchased from commercial suppliers and used as received. **^{1}H and ^{13}C NMR** spectra were recorded on a Bruker ACF300 (300 MHz), ACF400 (400 MHz) or AMX500 (500 MHz) spectrometer. Chemical shifts were reported in parts per million (ppm), and the residual solvent peak was used as an internal reference: proton (chloroform δ 7.26; dichloromethane 5.32) or carbon (chloroform δ 77.16; dichloromethane 53.84). Data are reported as follows: chemical shift, multiplicity (s = singlet, d = doublet, t = triplet, q = quartet, m = multiplet, br = broad), coupling constants (Hz) and integration. High resolution mass spectra (**HRMS**) were obtained on Bruker MicroTOF-QII LCMS spectrometer (ESI-TOF) or Agilent 7200 Q-TOF GCMS (EI-TOF). **Optical Rotations** were recorded on an mrc AP81 automatic polarimeter. Enantiomeric excess (**ee**) were determined by HPLC analysis on SHIMAZU HPLC units.

All reactions were carried out under nitrogen atmosphere unless otherwise noted. All commercially available reagents and catalysts were used as received for the reactions without any purification. All solvents were dried on alumina columns using a solvent dispensing system. All of pyrroles, alcohols, and **CPAs** were known compounds, which were purchased from commercial source or prepared according to reported methods.

2. Preparation of Oxime Ligands and Iridium Complexes

2.1. Preparation of oxime ligand

Most oxime ligands used in this study are known compounds. The following new oxime ligands were prepared according to a reported procedure[1].

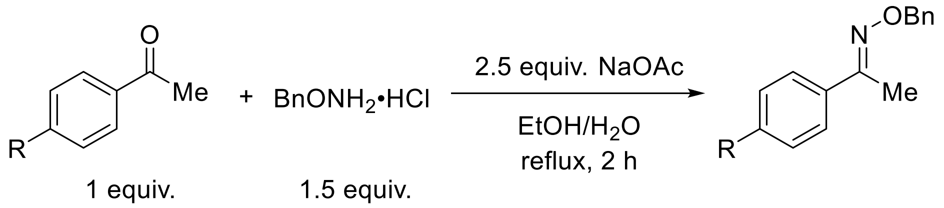

To a 25 mL round bottom flask were added *O*-benzylhydroxylamine hydrochloride (718 mg, 4.50 mmol), sodium acetate (615 mg, 7.50 mmol), ketone (3.00 mmol), EtOH (5 mL) and water (1.3 mL). The reaction mixture was refluxed with stirring for 2 h and then allowed to cool to room temperature. The mixture was treated with water (20 mL) and ethyl acetate (20 mL). The organic phase was separated and the aqueous solution was extracted with ethyl acetate (20 mL). The combined organic phase was dried over anhydrous sodium sulfate, filtered, concentrated under reduced pressure and purified by column chromatography (silica gel, hexanes/ethyl acetate = 100/1 – 40/1) to provide the desired product in pure form.

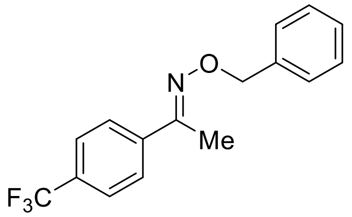

(*E*)-1-(4-(Trifluoromethyl)phenyl)ethan-1-one *O*-benzyl oxime: 472 mg (80% yield). White solid. (2 mmol 4′-(trifluoromethyl)acetophenone was used). **^{1}H NMR** (400 MHz, $CDCl_3$): δ 7.78 (d, *J* = 8.2 Hz, 2H), 7.63 (d, *J* = 8.4 Hz, 2H), 7.55 – 7.27 (m, 5H), 5.29 (s, 2H), 2.30 (s, 3H); **^{13}C NMR** (100 MHz, $CDCl_3$): δ 153.6, 140.0, 137.8, 130.8 (q, *J* = 32.5 Hz), 128.4, 128.2, 127.9, 126.3, 125.3 (q, *J* = 3.8 Hz), 124.1 (q, *J* = 272 Hz), 76.5, 12.7; **^{19}F NMR** (377 MHz, $CDCl_3$): δ -62.7. **HRMS** (EI-TOF): *m/z* for $[C_{16}H_{14}F_3NO]^+$ (M-e$^-$) calcd.: 293.1022; found: 293.1032.

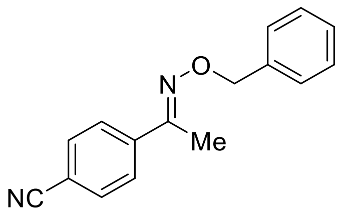

(*E*)-4-(1-((Benzyloxy)imino)ethyl)benzonitrile: 615 mg (82% yield). White solid. **^{1}H NMR** (400 MHz, $CDCl_3$): δ 7.80 – 7.70 (m, 2H), 7.68 – 7.58 (m, 2H), 7.49 – 7.27 (m, 5H), 5.28 (s, 2H), 2.27 (s, 3H); **^{13}C NMR** (100 MHz, $CDCl_3$): δ 153.1, 140.7, 137.5, 132.1, 128.4, 128.2, 127.9, 126.5, 118.6, 112.3, 76.6, 12.4. **HRMS** (EI-TOF): *m/z* for $[C_{16}H_{14}N_2O]^+$ (M-e$^-$) calcd.: 250.1101; found: 250.1106.

2.2. Preparation of iridium complexes

Iridium complexes (**4a** and **4b**) used in this study are known compounds. The following new iridium complexes (**4c** and **4c′**) were prepared according to a reported procedure[2].

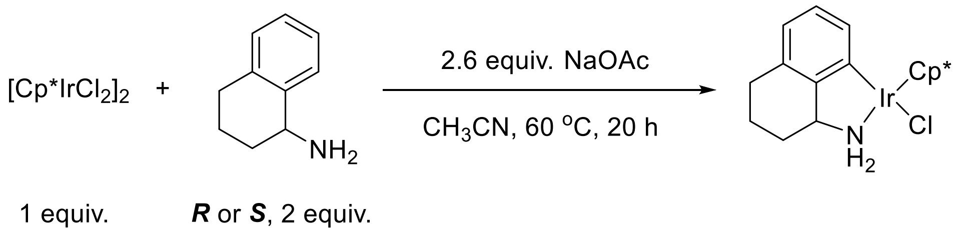

In a nitrogen-filled glove box, a Schlenk tube was charged with (*R*)-1,2,3,4-tetrahydro-1-naphthylamine or (*S*)-1,2,3,4-tetrahydro-1-naphthylamine (36.8 mg, 0.250 mmol), $[Cp^*IrCl_2]_2$ (100.0 mg, 0.126 mmol), sodium acetate (27.0 mg, 0.330 mmol) and acetonitrile (5 mL). The tube was then sealed, and the reaction mixture was allowed to stir at 60 ˚C for 20 h. After cooling to room temperature, the mixture was filtered through a pad of celite and the filtrate was concentrated under reduced pressure. The resulting mixture was purified by column chromatography (silica gel, hexanes/ethyl acetate = 1/2) to provide the desired complex **4c** or **4c′** as a yellow solid.

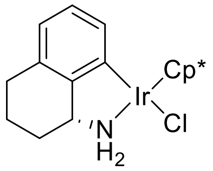

Iridium complex 4c: 84.5 mg (66% yield). Yellow solid. **^{1}H NMR** (400 MHz, $CDCl_3$): δ 7.30 (d,

J = 7.3 Hz, 1H), 6.99 (dd, J = 7.4 Hz, 7.4 Hz, 1H), 6.62 (d, J = 7.4 Hz, 1H), 4.26 – 3.98 (m, 1H), 3.74 – 3.48 (m, 2H), 2.76 – 2.52 (m, 2H), 2.29 – 2.14 (m, 1H), 1.97 – 1.88 (m, 1H), 1.74 (s, 15H), 1.66 – 1.61 (m, 1H), 1.58 – 1.48 (m, 1H); **^{13}C NMR** (100 MHz, $CDCl_3$): δ 157.9, 144.1, 133.9, 133.4, 128.1, 122.6, 86.3, 64.1, 34.2, 28.0, 22.9, 9.4. **HRMS** (ESI-TOF): *m/z* for $[C_{20}H_{27}IrN]^+$ (M-Cl^-) calcd.: 474.1768; found: 474.1766.

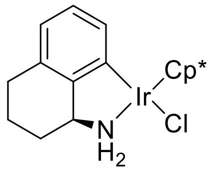

Iridium complex 4c′: 85.2 mg (67% yield). Yellow solid. **^{1}H NMR** (400 MHz, $CDCl_3$): δ 7.30 (d, J = 7.3 Hz, 1H), 6.98 (dd, J = 7.4 Hz, 7.4 Hz, 1H), 6.61 (d, J = 7.4 Hz, 1H), 4.30 – 4.03 (m, 1H), 3.73 – 3.45 (m, 2H), 2.78 – 2.52 (m, 2H), 2.30 – 2.12 (m, 1H), 1.99 – 1.87 (m, 1H), 1.73 (s, 15H), 1.67 – 1.61 (m, 1H), 1.57 – 1.47 (m, 1H); **^{13}C NMR** (100 MHz, $CDCl_3$): δ 157.9, 144.2, 133.9, 133.4, 128.0, 122.6, 86.3, 64.1, 34.1, 28.0, 22.9, 9.3. **HRMS** (ESI-TOF): *m/z* for $[C_{20}H_{27}IrN]^+$ (M-Cl^-) calcd.: 474.1768; found: 474.1770.

The following iridium complex (**4d**) was prepared according to a reported procedure[2].

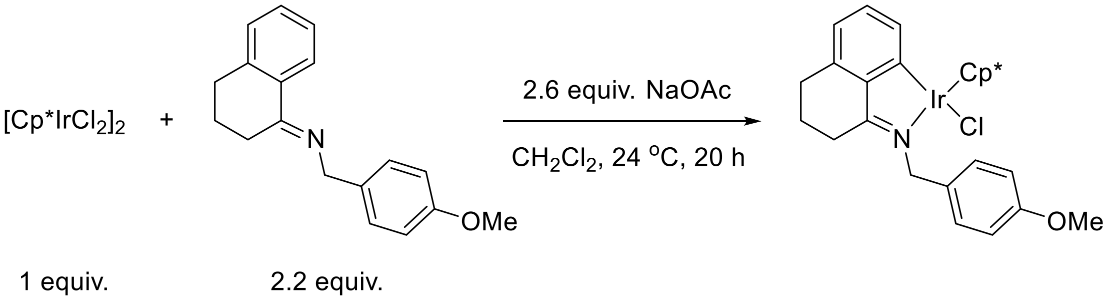

In a nitrogen-filled glove box, a Schlenk tube was charged with *N*-(4-methoxybenzyl)-3,4-dihydronaphthalen-1(2H)-imine (37.2 mg, 0.140 mmol), $[Cp^*IrCl_2]_2$ (50.0 mg, 0.0628 mmol), sodium acetate (13.5 mg, 0.165 mmol) and dichloromethane (2.5 mL). The tube was then sealed, and the reaction mixture was allowed to stir at 24 ˚C for 20 h. The resulting mixture was purified by column chromatography (silica gel, hexanes/ethyl acetate = 3/1 – 2/1) to provide the desired complex **4d** as a yellow solid.

Iridium complex 4d: 67.2 mg (85% yield). Yellow solid. **^{1}H NMR** (400 MHz, $CDCl_3$): δ 7.62 (d, J = 7.5 Hz, 1H), 7.17 – 7.05 (m, 3H), 6.94 – 6.82 (m, 2H), 6.75 (d, J = 7.3 Hz, 1H), 5.46 (d, J = 16.1

Hz, 1H), 5.23 (d, J = 16.1 Hz, 1H), 3.81 (s, 3H), 2.88 – 2.64 (m, 4H), 2.01 – 1.79 (m, 2H), 1.59 (s, 15H); **^{13}C NMR** (126 MHz, $CDCl_3$): δ 184.3, 167.6, 158.6, 144.7, 141.7, 132.7, 131.5, 128.9, 127.3, 120.8, 113.9, 88.8, 60.5, 55.2, 29.7, 28.8, 23.7, 9.2. **HRMS** (ESI-TOF): *m/z* for $[C_{20}H_{27}IrN]^+$ (M-Cl^-) calcd.: 592.2187; found: 592.2202.

The following iridium complexes (**4e-4k**) were prepared according to a reported procedure[2].

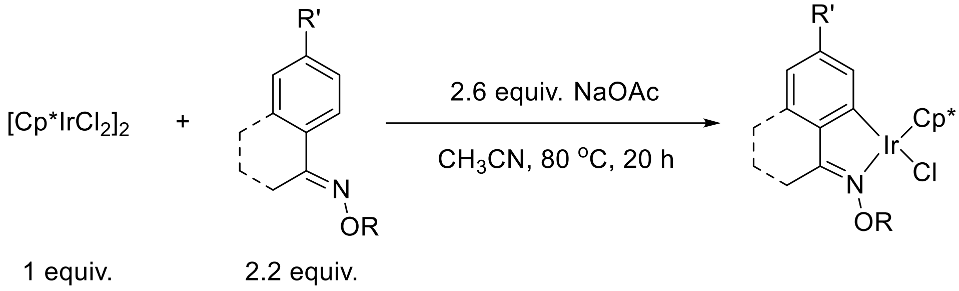

In a nitrogen-filled glove box, a Schlenk tube was charged with oxime (0.14 mmol), $[Cp^*IrCl_2]_2$ (50.0 mg, 0.0628 mmol), sodium acetate (13.5 mg, 0.165 mmol) and acetonitrile (2.5 mL). The tube was then sealed, and the reaction mixture was allowed to stir at 80 ˚C for 20 h. After cooling to room temperature, the mixture was filtered through a pad of celite and the filtrate was concentrated under reduced pressure. The resulting mixture was purified by column chromatography (silica gel, hexanes/ethyl acetate = 15/1 – 2/1) to provide the desired complex.

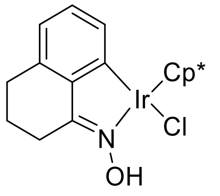

Iridium complex 4e: 51.2 mg, (78% yield). Yellow solid. **^{1}H NMR** (400 MHz, $CDCl_3$): δ 8.28 (brs, 1H), 7.49 (d, J = 7.4 Hz, 1H), 7.05 (dd, J = 7.4 Hz, 7.4 Hz, 1H), 6.75 (d, J = 7.4 Hz, 1H), 3.18 – 3.01 (m, 2H), 2.77 (dd, J = 5.9 Hz, 5.9 Hz, 2H), 1.97 – 1.83 (m, 2H), 1.74 (s, 15H); **^{13}C NMR** (126 MHz, $CDCl_3$): δ 171.1, 162.3, 139.9, 138.9, 132.3, 130.8, 121.3, 89.2, 28.8, 25.4, 22.7, 9.1. **HRMS** (ESI-TOF): *m/z* for $[C_{27}H_{31}IrNO]^+$ (M-Cl^-) calcd.: 488.1560; found: 488.1559.

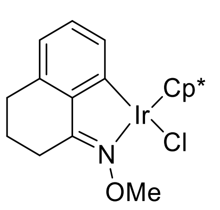

Iridium complex 4f: 54.6 mg (81% yield). Yellow solid. **^{1}H NMR** (400 MHz, $CDCl_3$): δ 7.51 (d, J

= 7.5 Hz, 1H), 7.08 (dd, J = 7.5 Hz, 7.5 Hz, 1H), 6.74 (d, J = 7.5 Hz, 1H), 3.82 (s, 3H), 3.28 – 3.15 (m, 1H), 3.06 – 2.95 (m, 1H), 2.77 (dd, J = 6.2 Hz, 6.1 Hz, 2H), 2.01 – 1.84 (m, 2H), 1.74 (s, 15H); **^{13}C NMR** (126 MHz, $CDCl_3$): δ 175.8, 163.6, 141.0, 138.1, 132.4, 131.6, 121.1, 89.0, 60.8, 28.9, 25.6, 22.8, 9.0. **HRMS** (ESI-TOF): *m/z* for $[C_{27}H_{31}IrNO]^+$ (M-Cl$^-$) calcd.: 502.1716; found: 502.1728.

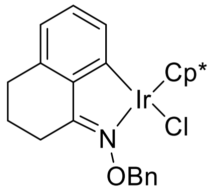

Iridium complex 4g: 42.2 mg (54% yield). Yellow solid. **^{1}H NMR** (400 MHz, $CDCl_3$): δ 7.65 – 7.45 (m, 3H), 7.44 – 7.27 (m, 3H), 7.11 (dd, J = 7.5 Hz, 7.5 Hz, 1H), 6.75 (d, J = 7.4 Hz, 1H), 5.21 (d, J = 11.6 Hz, 1H), 5.05 (d, J = 11.6 Hz, 1H), 3.13 (dt, J = 17.8 Hz, 6.2 Hz, 1H), 2.83 – 2.67 (m, 2H), 2.59 (dt, J = 17.7 Hz, 6.7 Hz, 1H), 1.90 – 1.65 (m, 17H); **^{13}C NMR** (126 MHz, $CDCl_3$): δ 177.2, 164.0, 141.1, 137.9, 135.8, 132.4, 131.8, 129.2, 128.5, 128.2, 121.3, 89.2, 75.0, 29.0, 26.4, 22.8, 9.1. **HRMS** (ESI-TOF): *m/z* for $[C_{27}H_{31}IrNO]^+$ (M-Cl$^-$) calcd.: 578.2030; found: 578.2034.

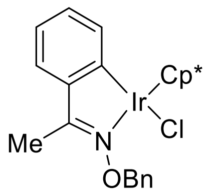

Iridium complex 4h: 65.8 mg (89%). Yellow solid. **^{1}H NMR** (400 MHz, $CDCl_3$): δ 7.75 (d, J = 7.6 Hz, 1H), 7.60 – 7.46 (m, 2H), 7.43 – 7.29 (m, 4H), 7.20 (td, J = 7.4 Hz, 1.3 Hz, 1H), 7.03 (td, J = 7.5 Hz, 1.0 Hz, 1H), 5.30 (d, J = 11.6 Hz, 1H), 5.05 (d, J = 11.6 Hz, 1H), 2.43 (s, 3H), 1.76 (s, 15H); **^{13}C NMR** (100 MHz, $CDCl_3$): δ 176.4, 164.0, 141.3, 135.5, 134.9, 131.6, 129.1, 128.5, 128.2, 126.9, 121.7, 89.4, 75.1, 12.9, 9.0. **HRMS** (ESI-TOF): *m/z* for $[C_{25}H_{29}IrNO]^+$ (M-Cl$^-$) calcd.: 552.1873; found: 552.1882.

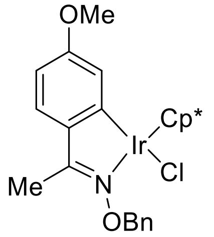

Iridium complex 4i: 66.4 mg (88% yield). Yellow solid. **^{1}H NMR** (400 MHz, $CDCl_3$): δ 7.64 – 7.46 (m, 2H), 7.41 – 7.26 (m, 5H), 6.59 (dd, J = 8.5 Hz, 2.4 Hz, 1H), 5.25 (d, J = 11.6 Hz, 1H), 5.01

(d, J = 11.6 Hz, 1H), 3.87 (s, 3H), 2.37 (s, 3H), 1.76 (s, 15H); **^{13}C NMR** (100 MHz, $CDCl_3$): δ 175.4, 166.2, 161.8, 135.6, 134.3, 129.1, 128.4, 128.1, 119.5, 107.9, 89.3, 75.1, 55.0, 12.9, 9.0. **HRMS** (ESI-TOF): *m/z* for $[C_{26}H_{31}IrNO_2]^+$ (M-Cl^-) calcd.: 582.1979; found: 582.1978.

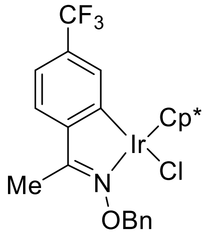

Iridium complex 4j: 38.6 mg (47% yield). Yellow solid. **^{1}H NMR** (400 MHz, $CDCl_3$): δ 7.96 (d, J = 1.6 Hz, 1H), 7.60 – 7.48 (m, 2H), 7.45 – 7.30 (m, 4H), 7.26 (dd, J = 8.0 Hz, 1.1 Hz, 1H), 5.29 (d, J = 11.7 Hz, 1H), 5.13 (d, J = 11.7 Hz, 1H), 2.41 (s, 3H), 1.76 (s, 15H); **^{13}C NMR** (126 MHz, $CDCl_3$): δ 175.9, 164.1, 144.9, 135.3, 132.0 (q, J = 30.7 Hz), 131.3 (q, J = 3.7 Hz), 129.3, 128.7, 128.5, 126.7, 124.3 (q, J = 273 Hz), 118.8 (q, J = 3.7 Hz), 90.1, 75.4, 13.2, 9.1; **^{19}F NMR** (377 MHz, $CDCl_3$): δ -62.3. **HRMS** (ESI-TOF): *m/z* for $[C_{26}H_{28}F_3IrNO]^+$ (M-Cl^-) calcd.: 620.1747; found: 620.1745.

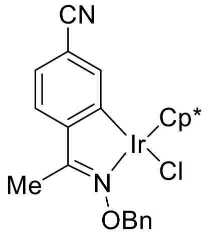

Iridium complex 4k: 26.7 mg (35% yield). Yellow solid. **^{1}H NMR** (400 MHz, $CDCl_3$): δ 7.96 (d, J = 1.3 Hz, 1H), 7.56 – 7.48 (m, 2H), 7.43 – 7.32 (m, 4H), 7.29 (dd, J = 7.9 Hz, 1.5 Hz, 1H), 5.28 (d, J = 11.6 Hz, 1H), 5.10 (d, J = 11.6 Hz, 1H), 2.39 (s, 3H), 1.76 (s, 15H); **^{13}C NMR** (100 MHz, $CDCl_3$): δ 175.7, 163.9, 145.9, 138.0, 135.0, 129.2, 128.6, 128.5, 126.5, 125.4, 119.5, 114.0, 90.2, 75.5, 13.1, 9.0. **HRMS** (ESI-TOF): *m/z* for $[C_{26}H_{28}IrN_2O]^+$ (M-Cl^-) calcd.: 577.1825; found: 577.1822.

3. Optimization of Enantioconvergent Heteroarylation of Simple Alcohols

Supplementary Table 1. Solvent screening for enantioconvergent heteroarylation of **2a** with **1a**.

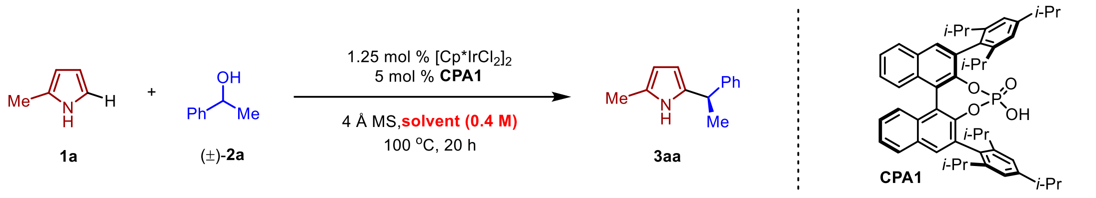

entry	solvent	yield of **3aa** (%)	ee of **3aa** (%)	entry	solvent	yield of **3aa** (%)	ee of **3aa** (%
1	toluene	79	55	9	diethyl carbonate	27	46
2	*p*-xylene	67	51	10	THF	24	37
3	mesitylene	62	49	11	1,4-dioxane	10	23
4	PhCl	83	53	12	CPME	54	52
5	anisole	38	52	13	^{t}BuOMe	86	37
6	cyclohexane	23	27	14	DCE	17	37
7	*tert*-amyl alcohol	6	37	15	Cl_4C	0	-
**8**	**dimethyl carbonate**	**42**	**76**	16	CH_3CN	trace	-

Reaction conditions: **1a** (0.20 mmol), **2a** (0.40 mmol), $[Cp^*IrCl_2]_2$ (0.005 mmol), **CPA1** (0.01 mmol) and 4 Å MS (20 mg) in solvent (0.5 mL) at 100 °C under N_2 for 20 h.

Supplementary Table 2. CPA screening for enantioconvergent heteroarylation of **2a** with **1a**.

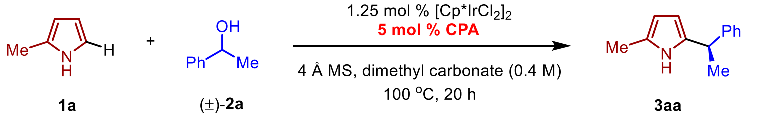

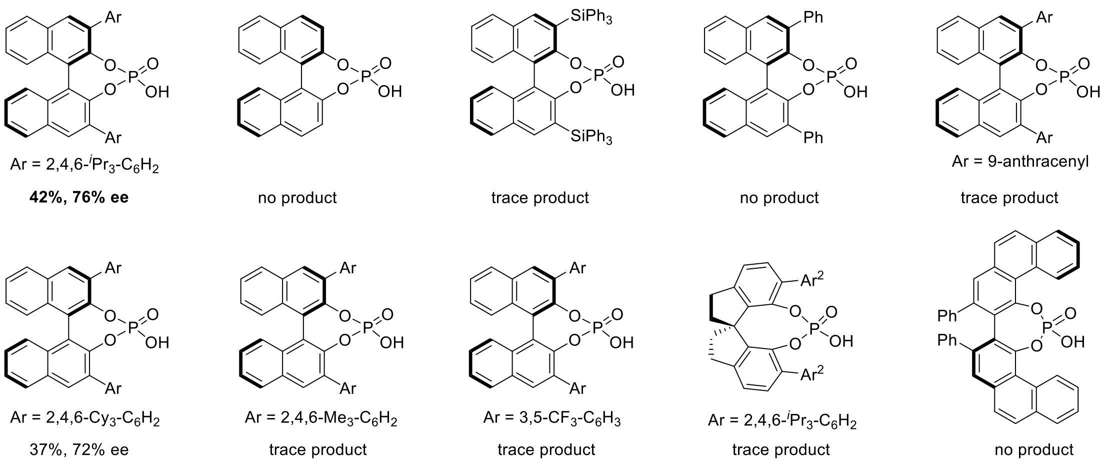

Reaction conditions: **1a** (0.20 mmol), **2a** (0.40 mmol), $[Cp^*IrCl_2]_2$ (0.005 mmol), **CPA** (0.01 mmol) and 4 Å MS (20 mg) in dimethyl carbonate (0.5 mL) at 100 °C under N_2 for 20 h.

4. Representative Procedures for Enantioconvergent Heteroarylation of Simple Alcohols

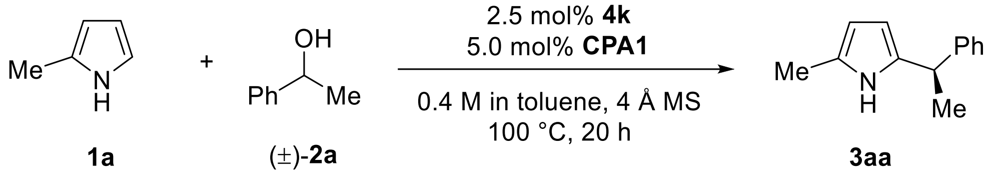

In a nitrogen-filled glove box, an 8 mL vial was charged with iridium complex (**4k**, 3.1 mg, 0.0050 mmol), **CPA1** (7.5 mg, 0.010 mmol), 4 Å molecular sieves (20 mg), 2-methylpyrrole (**1a**, 16.2 mg, 0.200 mmol) and 1-phenylethanol (**2a**, 48.9 mg, 0.400 mmol) and toluene (0.5 mL). The reaction tube was then sealed, taken outside the glovebox, heated to 100 ºC and allowed to stir for 20 h. The resulting mixture was cooled to room temperature and then purified by column chromatography (silica gel, hexanes/Et_2O/Et_3N = 15/1/0.15) to provide the desired alkylated product 2-methyl-5-(1-phenylethyl)-1H-pyrrole (**3aa**) as a colorless oil. For other related products, hexanes/Et_2O/Et_3N = 20/1/0.2 – 2/1/0.03 or hexanes/CH_2Cl_2/Et_3N = 5/1/0.05 – 3/1/0.03 were used as the eluents for purification.

5. Characterization of Products of Enantioconvergent Heteroarylation

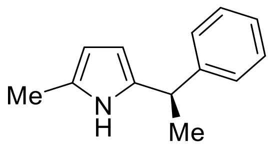

(*R*)-2-Methyl-5-(1-phenylethyl)-1*H*-pyrrole (**3aa**): 30.2 mg (82% yield). Colorless oil. **^{1}H NMR** (500 MHz, $CDCl_3$): δ 7.39 (brs, 1H), 7.34 – 7.28 (m, 2H), 7.25 – 7.15 (m, 3H), 5.93 (brs, 1H), 5.80 (brs, 1H), 4.04 (q, J = 7.1 Hz, 1H), 2.18 (s, 3H), 1.59 (d, J = 7.1 Hz, 3H); **^{13}C NMR** (100 MHz, $CDCl_3$): δ 145.8, 134.7, 128.6, 127.4, 126.8, 126.4, 105.4, 104.8, 38.7, 21.5, 13.0. **HRMS** (EI-TOF): *m/z* for $[C_{13}H_{15}N]^+$ (M-e$^-$) calcd.: 185.1199; found: 185.1201.

HPLC: Chiralcel OB-H column, hexanes/*i*-PrOH = 90/10, 220 nm, 1.0 mL/min, t_{R1} = 9.4 min (major), t_{R2} = 12.2 min (minor), **ee** = 90%. **Optical Rotation**: $[\alpha]_D^{25}$ = -29.4 (*c* 1.02, CH_2Cl_2). The absolute configuration of **3aa** was assigned by analogy to that of **3ad**.

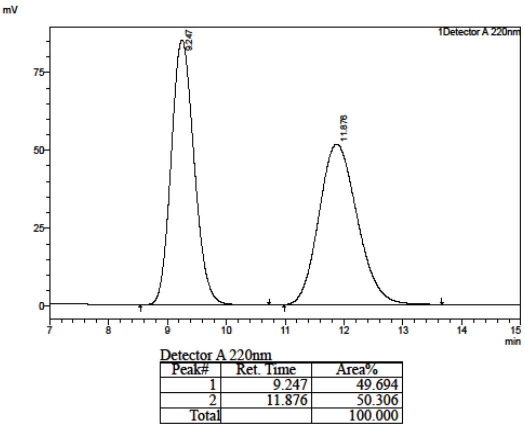

Detector A 220nm

Peak#	Ret. Time	Area%
1	9.247	49.694
2	11.876	50.306
Total		100.000

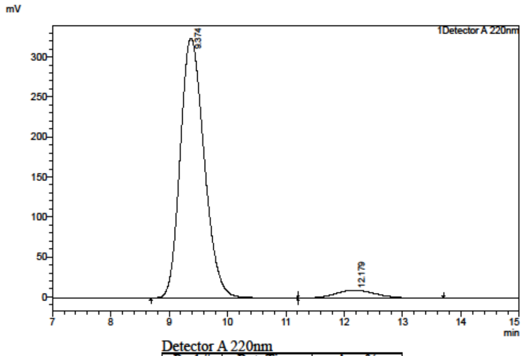

Detector A 220nm

Peak#	Ret. Time	Area%
1	9.374	95.175
2	12.179	4.825
Total		100.000

Supplementary Figure 1. HPLC Trace of **3aa**.

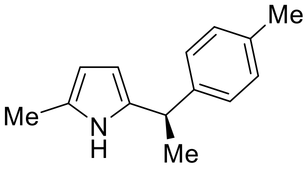

(*R*)-2-Methyl-5-(1-(*p*-tolyl)ethyl)-1*H*-pyrrole (**3ab**): 32.9 mg (83% yield). Colorless oil. **^{1}H NMR** (400 MHz, $CDCl_3$): δ 7.37 (brs, 1H), 7.21 – 6.99 (m, 4H), 5.92 (brs, 1H), 5.79 (brs, 1H), 4.01 (q, *J* = 7.1 Hz, 1H), 2.33 (s, 3H), 2.17 (s, 3H), 1.56 (d, *J* = 7.1 Hz, 3H); **^{13}C NMR** (100 MHz, $CDCl_3$): δ 142.7, 135.9, 134.9, 129.2, 127.3, 126.7, 105.4, 104.7, 38.2, 21.6, 21.0, 13.0. **HRMS** (EI-TOF): *m/z* for $[C_{14}H_{17}N]^+$ (M-e$^-$) calcd.: 199.1356; found: 199.1352.

HPLC: Chiralcel OB-H column, hexanes/*i*-PrOH = 90/10, 220 nm, 1.0 mL/min, t_{R1} = 11.8 min (major), t_{R2} = 20.9 min (minor), **ee =** 94%. **Optical Rotation**: $[\alpha]_D^{25}$ = -19.5 (*c* 1.03, CH_2Cl_2). The absolute configuration of **3ab** was assigned by analogy to that of **3ad**.

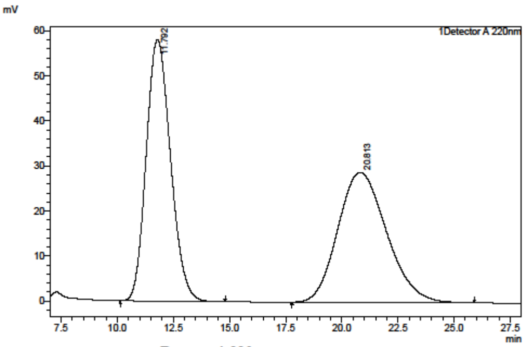

Detector A 220nm

Peak#	Ret. Time	Area%
1	11.792	50.168
2	20.813	49.832
Total		100.000

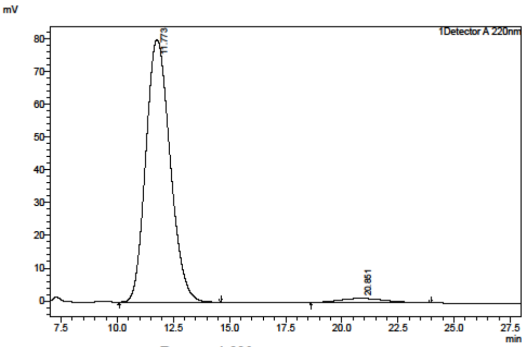

Detector A 220nm

Peak#	Ret. Time	Area%
1	11.773	97.015
2	20.851	2.985
Total		100.000

Supplementary Figure 2. HPLC Trace of **3ab**.

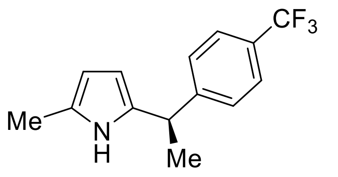

(*R*)-2-Methyl-5-(1-(4-(trifluoromethyl)phenyl)ethyl)-1*H*-pyrrole (**3ac**): 41.5 mg (82% yield). Colorless oil. **^{1}H NMR** (400 MHz, $CDCl_3$): δ 7.56 (d, *J* = 8.1 Hz, 2H), 7.39 (brs, 1H), 7.31 (d, *J* = 8.1 Hz, 2H), 5.99 – 5.89 (m, 1H), 5.86 – 5.75 (m, 1H), 4.10 (q, *J* = 7.2 Hz, 1H), 2.20 (s, 3H), 1.60 (d, *J* = 7.2 Hz, 3H); **^{13}C NMR** (75 MHz, $CDCl_3$): δ 150.0, 133.5, 128.7 (q, *J* = 32.3 Hz), 127.7, 127.3, 125.5 (q, *J* = 3.8 Hz), 124.2 (q, *J* = 272 Hz), 105.6, 105.4, 38.6, 21.3, 12.9; **^{19}F NMR** (377 MHz, $CDCl_3$): δ -62.3. **HRMS** (EI-TOF): *m/z* for $[C_{14}H_{14}F_3N]^+$ $(M-e^-)$ calcd.: 253.1073; found: 253.1072.

HPLC: Chiralcel OB-H column, hexanes/*i*-PrOH = 90/10, 220 nm, 0.8 mL/min, t_{R1} = 6.4 min (minor), t_{R2} = 7.7 min (major), **ee =** 88%. **Optical Rotation**: $[\alpha]_D^{25}$ = -19.0 (*c* 0.99, CH_2Cl_2). The absolute configuration of **3ac** was assigned by analogy to that of **3ad**.

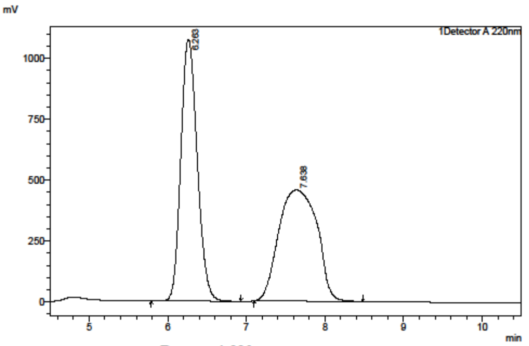

Detector A 220nm

Peak#	Ret. Time	Area%
1	6.263	49.961
2	7.638	50.039
Total		100.000

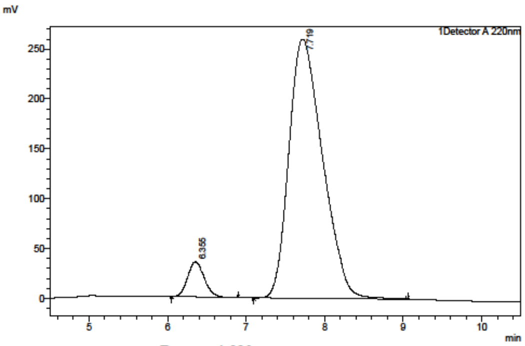

Detector A 220nm

Peak#	Ret. Time	Area%
1	6.355	6.233
2	7.719	93.767
Total		100.000

Supplementary Figure 3. HPLC Trace of **3ac**.

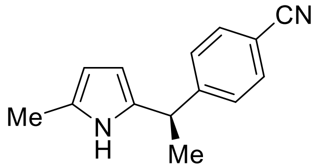

(*R*)-4-(1-(5-Methyl-1*H*-pyrrol-2-yl)ethyl)benzonitrile (**3ad**): 24.3 mg (58% yield). White solid. **^{1}H NMR** (300 MHz, $CDCl_3$): δ 7.61 – 7.54 (m, 2H), 7.46 (brs, 1H), 7.35 – 7.27 (m, 2H), 5.96 – 5.88 (m, 1H), 5.85 – 5.76 (m, 1H), 4.10 (q, *J* = 7.1 Hz, 1H), 2.20 (s, 3H), 1.59 (d, *J* = 7.1 Hz, 3H); **^{13}C NMR** (126 MHz, $CDCl_3$): δ 151.5, 132.8, 132.4, 128.2, 127.5, 118.9, 110.2, 105.7, 105.6, 38.8,

21.2, 13.0. **HRMS** (EI-TOF): *m/z* for $[C_{14}H_{14}N_2]^+$ (M-e^-) calcd.: 210.1151; found: 210.1153.

HPLC: Chiralcel OB-H column, hexanes/*i*-PrOH = 90/10, 220 nm, 1.0 mL/min, t_{R1} = 19.8 min (minor), t_{R2} = 32.9 min (major), **ee** = 87%. **Optical Rotation**: $[\alpha]_D^{25}$ = -13.9 (*c* 0.72, CH_2Cl_2). The absolute configuration of **3ad** was assigned to ***R*** by single crystal X-ray analysis.

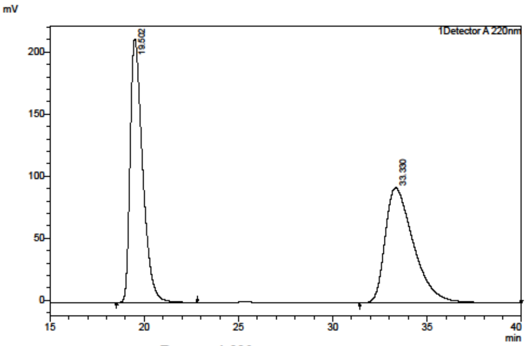

Detector A 220nm

Peak#	Ret. Time	Area%
1	19.502	49.973
2	33.330	50.027
Total		100.000

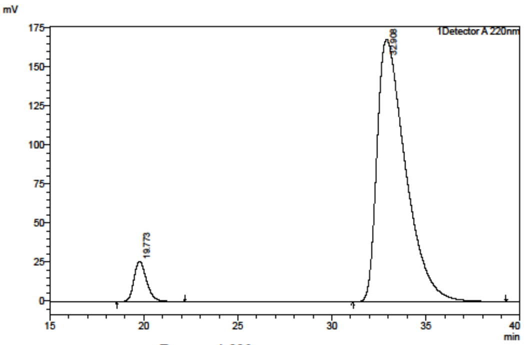

Detector A 220nm

Peak#	Ret. Time	Area%
1	19.773	6.383
2	32.908	93.617
Total		100.000

Supplementary Figure 4. HPLC Trace of **3ad**.

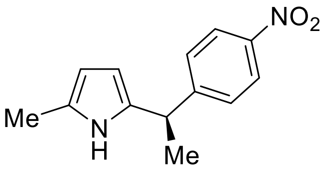

(*R*)-2-Methyl-5-(1-(4-nitrophenyl)ethyl)-1*H*-pyrrole (**3ae**): 32.6 mg (71% yield). Pale yellow solid. **^{1}H NMR** (400 MHz, CD_2Cl_2): δ 8.22 – 8.01 (m, 2H), 7.58 (brs, 1H), 7.42 – 7.31 (m, 2H), 5.96 – 5.83 (m, 1H), 5.81 – 5.68 (m, 1H), 4.17 (q, *J* = 7.2 Hz, 1H), 2.17 (s, 3H), 1.60 (d, *J* = 7.2 Hz, 3H); **^{13}C NMR** (126 MHz, CD_2Cl_2): δ 154.3, 147.0, 133.2, 128.6, 127.8, 124.1, 106.1, 105.9, 39.0, 21.4, 13.0. **HRMS** (EI-TOF): *m/z* for $[C_{13}H_{14}N_2O_2]^+$ (M-e^-) calcd.: 230.1050; found: 230.1051.

HPLC: Chiralcel OJ-H column, hexanes/*i*-PrOH = 90/10, 220 nm, 1.0 mL/min, t_{R1} = 22.3 min (minor), t_{R2} = 24.0 min (major), **ee** = 83%. **Optical Rotation**: $[\alpha]_D^{25}$ = -10.0 (*c* 0.99, CH_2Cl_2). The absolute configuration of **3ae** was assigned by analogy to that of **3ad**.

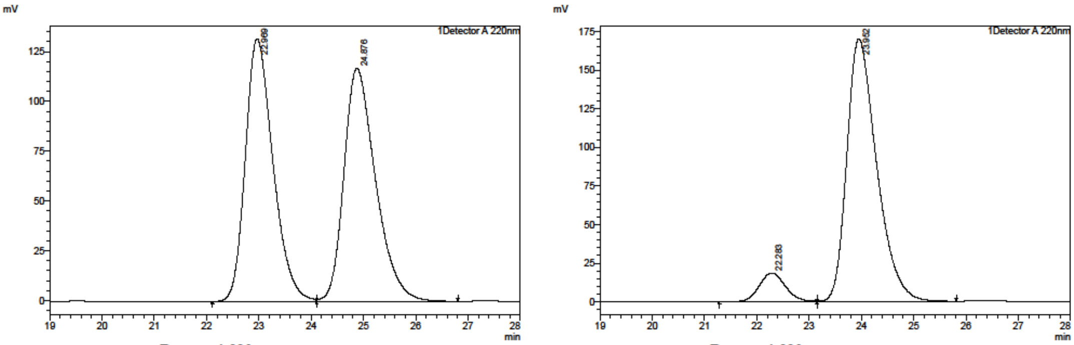

Detector A 220nm

Peak#	Ret. Time	Area%
1	22.969	49.733
2	24.876	50.267
Total		100.000

Detector A 220nm

Peak#	Ret. Time	Area%
1	22.283	8.705
2	23.952	91.295
Total		100.000

Supplementary Figure 5. HPLC Trace of **3ae**.

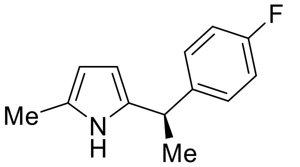

(*R*)-2-(1-(4-Fluorophenyl)ethyl)-5-methyl-1*H*-pyrrole (**3af**): 32.2 mg (79% yield). Colorless oil. **^{1}H NMR** (500 MHz, $CDCl_3$): δ 7.39 (brs, 1H), 7.23 – 7.13 (m, 2H), 7.06 – 6.94 (m, 2H), 5.97 – 5.87 (m, 1H), 5.86 – 5.73 (m, 1H), 4.04 (q, *J* = 7.1 Hz, 1H), 2.20 (s, 3H), 1.57 (d, *J* = 7.1 Hz, 3H); **^{13}C NMR** (126 MHz, $CDCl_3$): δ 161.5 (d, *J* = 245 Hz), 141.4 (d, *J* = 3.3 Hz), 134.5, 128.8 (d, *J* = 8.0 Hz), 127.0, 115.3 (d, *J* = 21.2 Hz), 105.5, 105.0, 38.0, 21.6, 13.0. **HRMS** (EI-TOF): *m/z* for $[C_{13}H_{14}FN]^+$ (M-e$^-$) calcd.: 203.1105; found: 203.1107.

HPLC: Chiralcel OJ-H column, hexanes/*i*-PrOH = 90/10, 220 nm, 1.0 mL/min, t_{R1} = 12.2 min (major), t_{R2} = 27.4 min (minor), **ee =** 87%. **Optical Rotation**: $[\alpha]_D^{25}$ = -24.0 (*c* 1.00, CH_2Cl_2). The absolute configuration of **3af** was assigned by analogy to that of **3ad**.

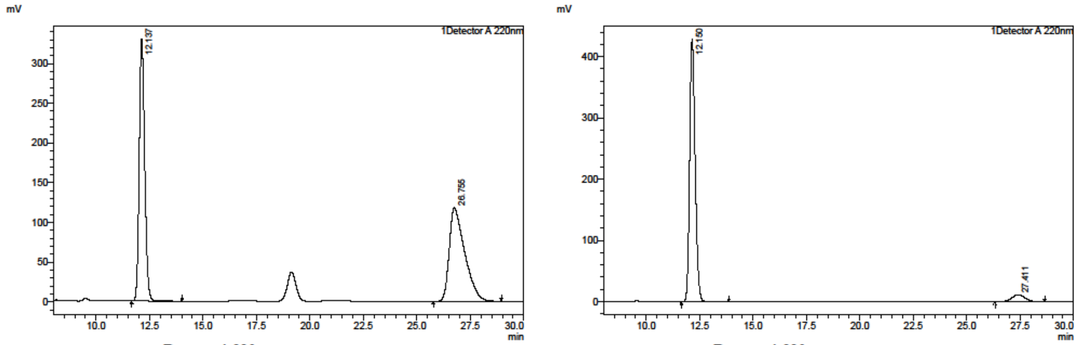

Detector A 220nm

Peak#	Ret. Time	Area%
1	12.137	49.973
2	26.755	50.027
Total		100.000

Detector A 220nm

Peak#	Ret. Time	Area%
1	12.150	93.706
2	27.411	6.294
Total		100.000

Supplementary Figure 6. HPLC Trace of **3af**.

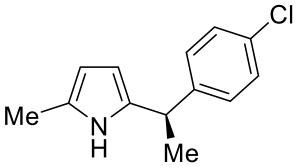

(*R*)-2-(1-(4-Chlorophenyl)ethyl)-5-methyl-1*H*-pyrrole (**3ag**): 39.8 mg (91% yield). Colorless oil. **^{1}H NMR** (400 MHz, CD_2Cl_2): δ 7.50 (brs, 1H), 7.32 – 7.24 (m, 2H), 7.20 – 7.11 (m, 2H), 5.94 – 5.80 (m, 1H), 5.80 – 5.64 (m, 1H), 4.03 (q, *J* = 7.2 Hz, 1H), 2.17 (s, 3H), 1.55 (d, *J* = 7.2 Hz, 3H); **^{13}C NMR** (100 MHz, CD_2Cl_2): δ 145.2, 134.4, 132.2, 129.2, 128.9, 127.3, 105.9, 105.3, 38.5, 21.6, 13.0. **HRMS** (EI-TOF): *m/z* for $[C_{13}H_{14}ClN]^+$ (M-e$^-$) calcd.: 219.0809; found: 219.0806.

HPLC: Chiralcel OJ-H column, hexanes/*i*-PrOH = 90/10, 220 nm, 1.0 mL/min, t_{R1} = 10.4 min (major), t_{R2} = 14.9 min (minor), **ee =** 89%. **Optical Rotation**: $[\alpha]_D^{25}$ = -11.8 (*c* 1.00, CH_2Cl_2). The absolute configuration of **3ag** was assigned by analogy to that of **3ad**.

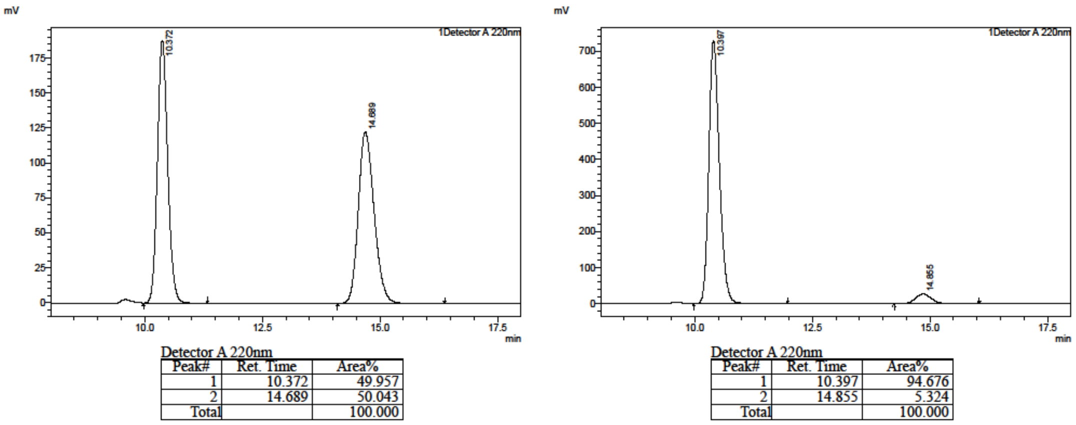

Detector A 220nm

Peak#	Ret. Time	Area%
1	10.372	49.957
2	14.689	50.043
Total		100.000

Detector A 220nm

Peak#	Ret. Time	Area%
1	10.397	94.676
2	14.855	5.324
Total		100.000

Supplementary Figure 7. HPLC Trace of **3ag**.

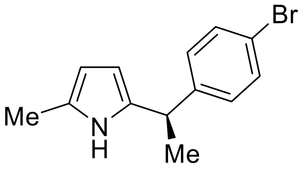

(*R*)-2-(1-(4-Bromophenyl)ethyl)-5-methyl-1*H*-pyrrole (**3ah**): 48.5 mg (92% yield). White solid. **^{1}H NMR** (400 MHz, CD_2Cl_2): δ 7.48 (brs, 1H), 7.44 – 7.27 (m, 2H), 7.15 – 7.02 (m, 2H), 5.94 – 5.79 (m, 1H), 5.79 – 5.61 (m, 1H), 4.00 (q, *J* = 7.2 Hz, 1H), 2.15 (s, 3H), 1.54 (d, *J* = 7.2 Hz, 3H); **^{13}C NMR** (126 MHz, CD_2Cl_2): δ 145.7, 134.3, 131.9, 129.6, 127.3, 120.3, 105.9, 105.4, 38.6, 21.6, 13.0. **HRMS** (EI-TOF): *m/z* for $[C_{13}H_{14}BrN]^+$ (M-e$^-$) calcd.: 263.0304; found: 263.0304.

HPLC: Chiralcel OJ-H column, hexanes/*i*-PrOH = 90/10, 220 nm, 1.0 mL/min, t_{R1} = 10.4 min (major), t_{R2} = 13.0 min (minor), **ee =** 90%. **Optical Rotation**: $[\alpha]_D^{25}$ = -7.3 (*c* 0.99, CH_2Cl_2). The absolute configuration of **3ah** was assigned by analogy to that of **3ad**.

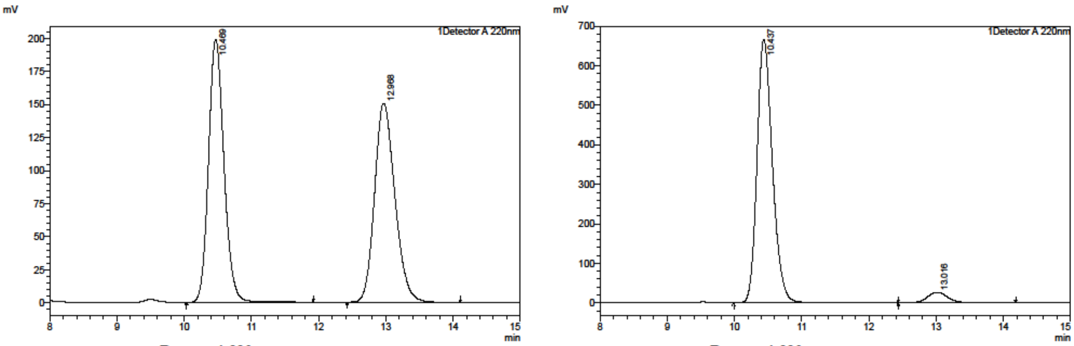

Detector A 220nm

Peak#	Ret. Time	Area%
1	10.469	50.105
2	12.968	49.895
Total		100.000

Detector A 220nm

Peak#	Ret. Time	Area%
1	10.437	94.860
2	13.016	5.140
Total		100.000

Supplementary Figure 8. HPLC Trace of **3ah**.

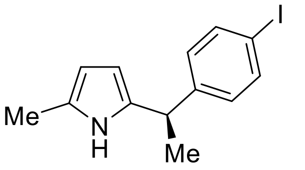

(*R*)-2-(1-(4-Iodophenyl)ethyl)-5-methyl-1*H*-pyrrole (**3ai**): 51.5 mg (83% yield). White solid. **^{1}H NMR** (400 MHz, CD_2Cl_2): δ 7.69 – 7.58 (m, 2H), 7.49 (brs, 1H), 7.05 – 6.83 (m, 2H), 5.85 (brs, 1H), 5.73 (brs, 1H), 3.98 (q, *J* = 7.1 Hz, 1H), 2.15 (s, 3H), 1.53 (d, *J* = 7.1 Hz, 3H); **^{13}C NMR** (126 MHz, CD_2Cl_2): δ 146.0, 137.5, 133.9, 129.5, 126.9, 105.5, 105.0, 91.3, 38.3, 21.2, 12.7. **HRMS** (EI-TOF): *m/z* for $[C_{13}H_{14}IN]^+$ (M-e$^-$) calcd.: 311.0165; found: 311.0163.

HPLC: Chiralcel OJ-H column, hexanes/*i*-PrOH = 90/10, 220 nm, 1.0 mL/min, t_{R1} = 10.9 min (major), t_{R2} = 11.7 min (minor), **ee =** 86%. **Optical Rotation**: $[\alpha]_D^{25}$ = 0.3 (*c* 0.95, CH_2Cl_2). The absolute configuration of **3ai** was assigned by analogy to that of **3ad**.

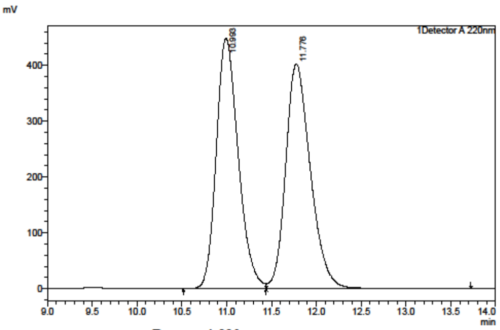

Detector A 220nm

Peak#	Ret. Time	Area%
1	10.993	49.697
2	11.776	50.303
Total		100.000

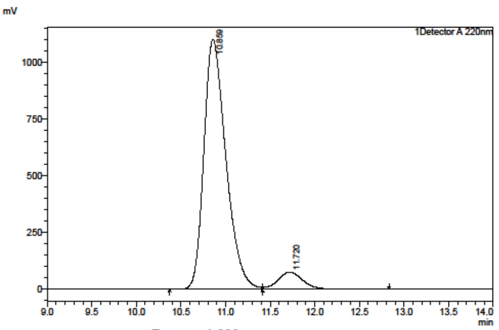

Detector A 220nm

Peak#	Ret. Time	Area%
1	10.859	92.922
2	11.720	7.078
Total		100.000

Supplementary Figure 9. HPLC Trace of **3ai**.

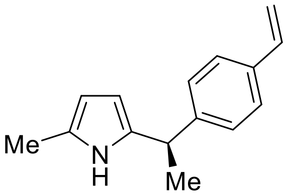

(*R*)-2-Methyl-5-(1-(4-vinylphenyl)ethyl)-1*H*-pyrrole (**3aj**): 23.7 mg (56% yield). Colorless oil. **^{1}H NMR** (400 MHz, CD_2Cl_2): δ 7.49 (brs, 1H), 7.40 – 7.28 (m, 2H), 7.23 – 7.11 (m, 2H), 6.71 (dd, *J* = 17.6 Hz, 10.9 Hz, 1H), 5.92 – 5.81 (m, 1H), 5.77 – 5.66 (m, 1H), 5.72 (dd, *J* = 17.6 Hz, 0.9 Hz, 1H), 5.21 (dd, *J* = 10.9 Hz, 0.9 Hz, 1H), 4.02 (q, *J* = 7.1 Hz, 1H), 2.15 (s, 3H), 1.55 (d, *J* = 7.1 Hz, 3H); **^{13}C NMR** (126 MHz, CD_2Cl_2): δ 146.2, 137.0, 136.3, 134.9, 127.9, 127.1, 126.8, 113.5, 105.9, 105.2, 38.8, 21.6, 13.1. **HRMS** (EI-TOF): *m/z* for $[C_{15}H_{17}N]^{+}$ (M-e^{-}) calcd.: 211.1356; found: 211.1356.

HPLC: Chiralcel OJ-H column, hexanes/*i*-PrOH = 90/10, 220 nm, 1.0 mL/min, t_{R1} = 15.8 min (major), t_{R2} = 25.5 min (minor), **ee** = 85%. **Optical Rotation**: $[\alpha]_D^{25}$ = -6.7 (*c* 1.03, CH_2Cl_2). The absolute configuration of **3aj** was assigned by analogy to that of **3ad**.

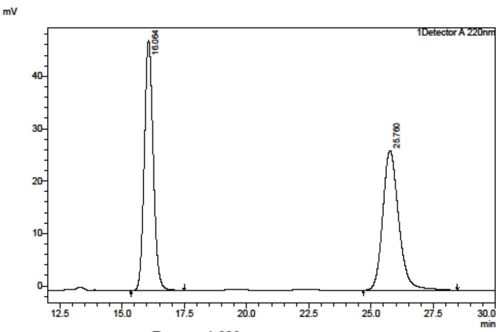

Detector A 220nm

Peak#	Ret. Time	Area%
1	16.064	50.057
2	25.760	49.943
Total		100.000

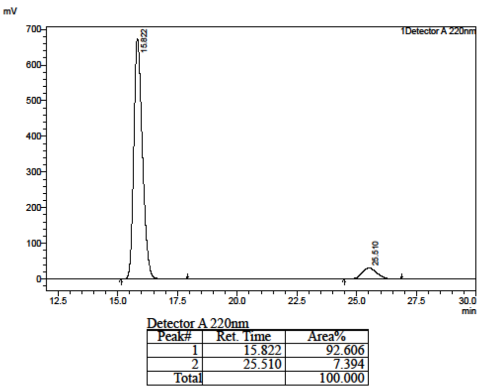

Detector A 220nm

Peak#	Ret. Time	Area%
1	15.822	92.606
2	25.510	7.394
Total		100.000

Supplementary Figure 10. HPLC Trace of **3aj**.

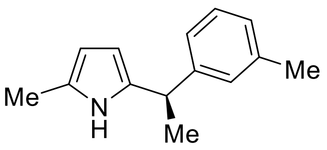

(*R*)-2-Methyl-5-(1-(*m*-tolyl)ethyl)-1*H*-pyrrole (**3ak**): 34.1 mg (86% yield). Colorless oil. **^{1}H NMR** (400 MHz, CD_2Cl_2): δ 7.49 (brs, 1H), 7.26 – 7.12 (m, 1H), 7.11 – 6.89 (m, 3H), 5.93 – 5.79 (m, 1H), 5.79 – 5.63 (m, 1H), 3.98 (q, *J* = 7.2 Hz, 1H), 2.32 (s, 3H), 2.15 (s, 3H), 1.55 (d, *J* = 7.2 Hz, 3H); **^{13}C NMR** (126 MHz, CD_2Cl_2): δ 146.4, 138.6, 135.2, 128.8, 128.4, 127.5, 127.0, 124.7, 105.8, 104.9, 39.0, 21.6, 13.0. **HRMS** (EI-TOF): *m/z* for $[C_{14}H_{17}N]^+$ (M-e$^-$) calcd.: 199.1356; found: 199.1356.

HPLC: Chiralcel OJ-H column, hexanes/*i*-PrOH = 90/10, 220 nm, 1.0 mL/min, t_{R1} = 12.2 min (major), t_{R2} = 32.9 min (minor), **ee =** 92%. **Optical Rotation**: $[\alpha]_D^{25}$ = -29.5 (*c* 0.97, CH_2Cl_2). The absolute configuration of **3ak** was assigned by analogy to that of **3ad**.

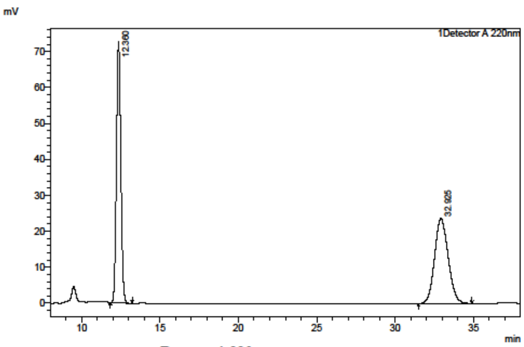

Detector A 220nm

Peak#	Ret. Time	Area%
1	12.360	49.906
2	32.925	50.094
Total		100.000

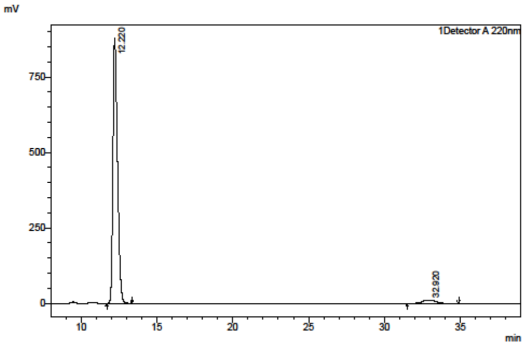

Detector A 220nm

Peak#	Ret. Time	Area%
1	12.220	96.150
2	32.920	3.850
Total		100.000

Supplementary Figure 11. HPLC Trace of **3ak**.

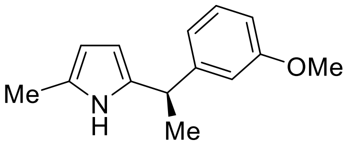

(*R*)-2-(1-(3-Methoxyphenyl)ethyl)-5-methyl-1*H*-pyrrole (**3al**): 34.7 mg (81% yield). Colorless oil. **^{1}H NMR** (400 MHz, CD_2Cl_2): δ 7.54 (brs, 1H), 7.28 – 7.19 (m, 1H), 6.90 – 6.62 (m, 3H), 5.94 – 5.82 (m, 1H), 5.79 – 5.67 (m, 1H), 4.01 (q, *J* = 7.1 Hz, 1H), 3.78 (s, 3H), 2.17 (s, 3H), 1.57 (d, *J* = 7.1 Hz, 3H); **^{13}C NMR** (126 MHz, CD_2Cl_2): δ 160.4, 148.2, 134.9, 129.9, 127.0, 120.1, 113.7, 111.8, 105.9, 105.1, 55.5, 39.1, 21.5, 13.1. **HRMS** (EI-TOF): *m/z* for $[C_{14}H_{17}NO]^+$ (M-e$^-$) calcd.: 215.1305; found: 215.1308.

HPLC: Chiralcel OB-H column, hexanes/*i*-PrOH = 90/10, 220 nm, 1.0 mL/min, t_{R1} = 12.7 min (major), t_{R2} = 19.9 min (minor), **ee =** 93%. **Optical Rotation**: $[\alpha]_D^{25}$ = -21.2 (*c* 0.99, CH_2Cl_2). The absolute configuration of **3al** was assigned by analogy to that of **3ad**.

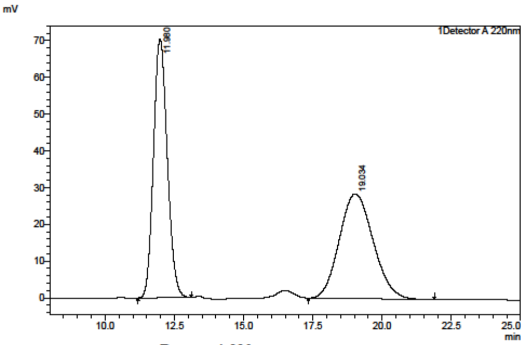

Detector A 220nm

Peak#	Ret. Time	Area%
1	11.980	50.198
2	19.034	49.802
Total		100.000

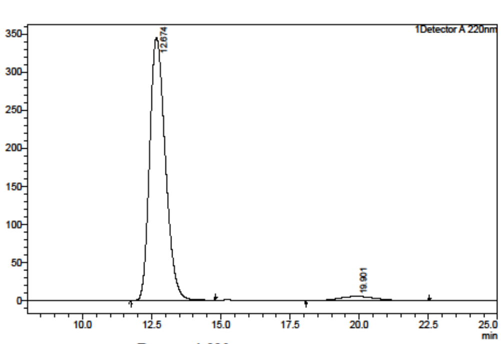

Detector A 220nm

Peak#	Ret. Time	Area%
1	12.674	96.277
2	19.901	3.723
Total		100.000

Supplementary Figure 12. HPLC Trace of **3al**.

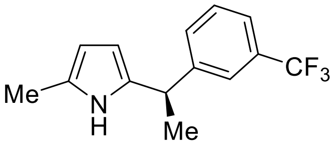

(*R*)-2-Methyl-5-(1-(3-(trifluoromethyl)phenyl)ethyl)-1*H*-pyrrole (**3am**): 46.4 mg (92% yield). Colorless oil. **^{1}H NMR** (400 MHz, CD_2Cl_2): δ 7.81 – 7.12 (m, 5H), 5.92 – 5.81 (m, 1H), 5.80 – 5.67 (m, 1H), 4.11 (q, *J* = 7.2 Hz, 1H), 2.17 (s, 3H), 1.58 (d, *J* = 7.2 Hz, 3H); **^{13}C NMR** (126 MHz, CD_2Cl_2): δ 147.3, 133.6, 131.0, 130.6 (q, *J* = 31.6 Hz), 129.1, 127.2, 124.5 (q, *J* = 272 Hz), 124.1 (q, *J* = 3.6 Hz), 123.2 (q, *J* = 3.6 Hz), 105.6, 105.3, 38.7, 21.3, 12.7; **^{19}F NMR** (377 MHz, CD_2Cl_2): δ -62.8. **HRMS** (EI-TOF): *m/z* for $[C_{14}H_{14}F_3N]^+$ (M-e$^-$) calcd.: 253.1073; found: 253.1076.

HPLC: Chiralcel OJ-H column, hexanes/*i*-PrOH = 90/10, 220 nm, 1.0 mL/min, t_{R1} = 5.9 min (major), t_{R2} = 8.0 min (minor), **ee =** 87%. **Optical Rotation**: $[\alpha]_D^{25}$ = -19.5 (*c* 1.02, CH_2Cl_2). The absolute configuration of **3am** was assigned by analogy to that of **3ad**.

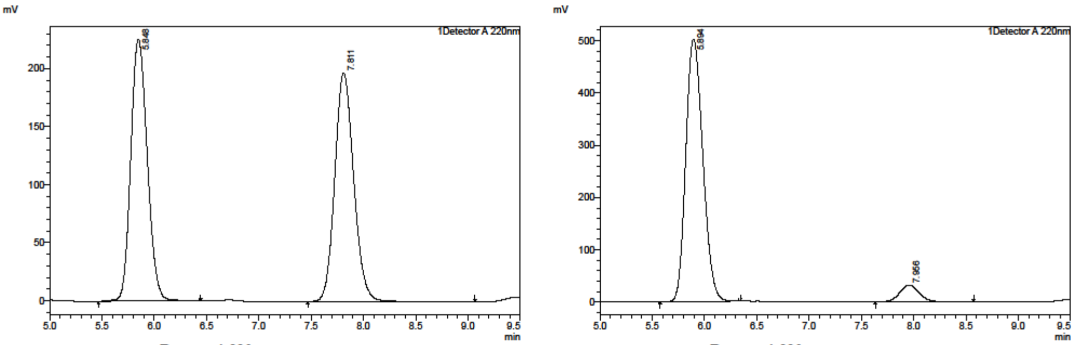

Detector A 220nm

Peak#	Ret. Time	Area%
1	5.848	50.039
2	7.811	49.961
Total		100.000

Detector A 220nm

Peak#	Ret. Time	Area%
1	5.894	93.421
2	7.956	6.579
Total		100.000

Supplementary Figure 13. HPLC Trace of **3am**.

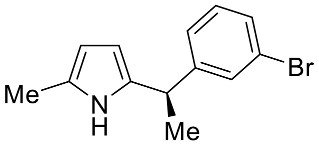

(*R*)-2-(1-(3-Bromophenyl)ethyl)-5-methyl-1*H*-pyrrole (**3an**): 45.1 mg (85% yield). Colorless oil. ^{1}**H NMR** (500 MHz, CD_2Cl_2): δ 7.52 (brs, 1H), 7.39 – 7.31 (m, 1H), 7.23 – 7.12 (m, 3H), 5.91 – 5.81 (m, 1H), 5.78 – 5.68 (m, 1H), 4.01 (q, *J* = 7.2 Hz, 1H), 2.17 (s, 3H), 1.55 (d, *J* = 7.2 Hz, 3H); ^{13}**C NMR** (126 MHz, CD_2Cl_2): δ 149.1, 134.1, 130.7, 130.6, 129.8, 127.4, 126.5, 122.9, 105.9, 105.4, 38.8, 21.5, 13.0. **HRMS** (EI-TOF): *m/z* for $[C_{13}H_{14}BrN]^+$ (M-e^-) calcd.: 263.0304; found: 263.0305.

HPLC: Chiralcel OJ-H column, hexanes/*i*-PrOH = 90/10, 220 nm, 1.0 mL/min, t_{R1} = 9.8 min (major), t_{R2} = 31.6 min (minor), **ee =** 88%. **Optical Rotation**: $[\alpha]_D^{25}$ = -11.9 (*c* 1.00, CH_2Cl_2). The absolute configuration of **3an** was assigned by analogy to that of **3ad**.

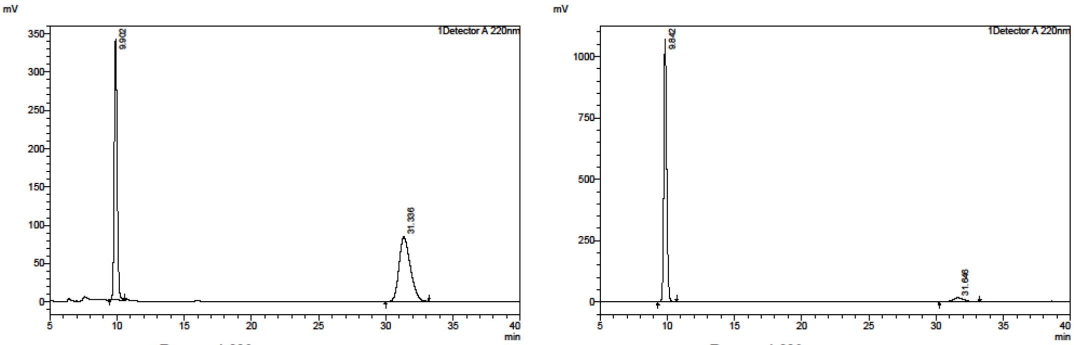

Detector A 220nm

Peak#	Ret. Time	Area%
1	9.902	50.408
2	31.336	49.592
Total		100.000

Detector A 220nm

Peak#	Ret. Time	Area%
1	9.842	94.230
2	31.646	5.770
Total		100.000

Supplementary Figure 14. HPLC Trace of **3an**.

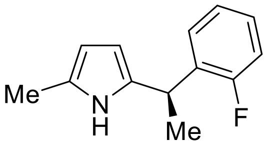

(*R*)-2-(1-(2-Fluorophenyl)ethyl)-5-methyl-1*H*-pyrrole (**3ao**): 34.4 m (85% yield). Colorless oil. **^{1}H NMR** (400 MHz, CD_2Cl_2): δ 7.65 (brs, 1H), 7.39 – 6.80 (m, 4H), 5.99 – 5.84 (m, 1H), 5.84 – 5.63 (m, 1H), 4.39 (q, *J* = 7.2 Hz, 1H), 2.18 (s, 3H), 1.58 (d, *J* = 7.2 Hz, 3H); **^{13}C NMR** (126 MHz, CD_2Cl_2): δ 160.8 (d, *J* = 244 Hz), 133.7, 133.2 (d, *J* = 14.1 Hz), 129.0 (d, *J* = 4.6 Hz), 128.2 (d, *J* = 8.3 Hz), 127.1, 124.8 (d, *J* = 3.3 Hz), 115.7 (d, *J* = 22.8 Hz), 105.9, 105.4, 31.7 (d, *J* = 2.8 Hz), 20.1, 13.1; **^{19}F NMR** (377 MHz, CD_2Cl_2): δ -120.2. **HRMS** (EI-TOF): *m/z* for $[C_{13}H_{14}FN]^+$ ($M-e^-$) calcd.: 203.1105; found: 203.1104.

HPLC: Chiralcel OJ-H column, hexanes/*i*-PrOH = 90/10, 220 nm, 1.0 mL/min, t_{R1} = 10.9 min (major), t_{R2} = 21.0 min (minor), **ee =** 91%. **Optical Rotation**: $[\alpha]_D^{25}$ = -6.1 (*c* 0.99, CH_2Cl_2). The absolute configuration of **3ao** was assigned by analogy to that of **3ad**.

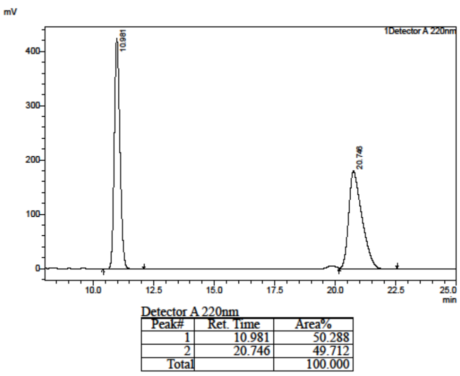

Detector A 220nm

Peak#	Ret. Time	Area%
1	10.981	50.288
2	20.746	49.712
Total		100.000

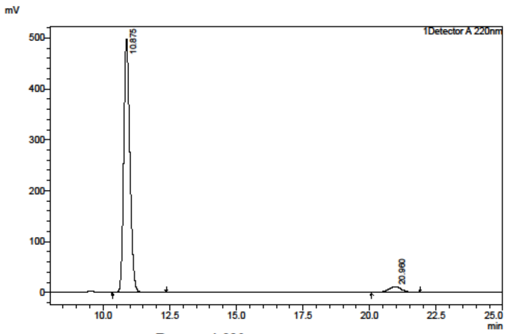

Detector A 220nm

Peak#	Ret. Time	Area%
1	10.875	95.746
2	20.960	4.254
Total		100.000

Supplementary Figure 15. HPLC Trace of **3ao**.

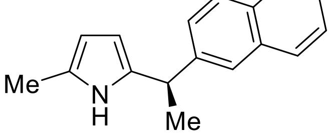

(*R*)-2-Methyl-5-(1-(naphthalen-2-yl)ethyl)-1*H*-pyrrole (**3ap**): 35.3 mg (75% yield). Colorless oil. **^{1}H NMR** (400 MHz, CD_2Cl_2): δ 7.91 – 7.75 (m, 3H), 7.69 (d, *J* = 0.8 Hz, 1H), 7.63 – 7.39 (m, 3H), 7.35 (dd, *J* = 8.5 Hz, 1.8 Hz, 1H), 6.05 – 5.86 (m, 1H), 5.86 – 5.63 (m, 1H), 4.21 (q, *J* = 7.1 Hz, 1H), 2.15 (s, 3H), 1.67 (d, *J* = 7.1 Hz, 3H); **^{13}C NMR** (126 MHz, CD_2Cl_2): δ 143.9, 134.9, 134.0, 132.8, 128.5, 128.0, 127.9, 127.2, 126.5, 126.4, 125.8, 125.7, 105.9, 105.2, 39.2, 21.4, 13.0. **HRMS**

(EI-TOF): *m/z* for $[C_{17}H_{17}N]^+$ (M-e$^-$) calcd.: 235.1356; found: 235.1354.

HPLC: Chiralcel OB-H column, hexanes/*i*-PrOH = 90/10, 254 nm, 1.0 mL/min, t_{R1} = 13.9 min (major), t_{R2} = 18.8 min (minor), **ee** = 96%. **Optical Rotation**: $[\alpha]_D^{25}$ = -47.2 (*c* 1.00, CH_2Cl_2). The absolute configuration of **3ap** was assigned by analogy to that of **3ad**.

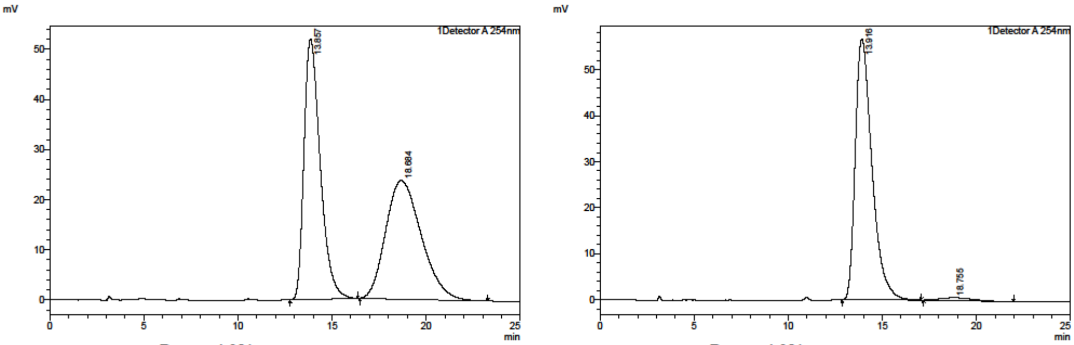

Detector A 254nm

Peak#	Ret. Time	Area%
1	13.857	49.917
2	18.684	50.083
Total		100.000

Detector A 254nm

Peak#	Ret. Time	Area%
1	13.916	97.902
2	18.755	2.098
Total		100.000

Supplementary Figure 16. HPLC Trace of **3ap**.

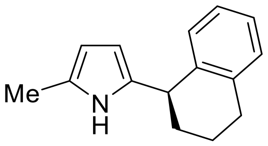

(*R*)-2-Methyl-5-(1,2,3,4-tetrahydronaphthalen-1-yl)-1*H*-pyrrole (**3aq**): 11.5 mg (27% yield). Colorless oil. **^{1}H NMR** (400 MHz, CD_2Cl_2): δ 7.53 (brs, 1H), 7.14 – 7.00 (m, 4H), 5.85 – 5.57 (m, 2H), 4.10 (t, *J* = 6.2 Hz, 1H), 2.90 – 2.75 (m, 2H), 2.18 (s, 3H), 2.13 – 2.03 (m, 1H), 1.97 – 1.84 (m, 2H), 1.82 – 1.72 (m, 1H); **^{13}C NMR** (126 MHz, CD_2Cl_2): δ 138.7, 137.8, 135.3, 130.0, 129.5, 126.6, 126.5, 126.0, 106.6, 105.9, 38.8, 32.0, 29.9, 21.4, 13.1. **HRMS** (EI-TOF): *m/z* for $[C_{15}H_{17}N]^+$ (M-e$^-$) calcd.: 211.1356; found: 211.1355.

HPLC: Chiralcel OD-H column, hexanes/*i*-PrOH = 99/1, 220 nm, 0.3 mL/min, t_{R1} = 25.3 min (major), t_{R2} = 27.2 min (minor), **ee** = 85%. **Optical Rotation**: $[\alpha]_D^{25}$ = 13.6 (*c* 1.00, CH_2Cl_2). The absolute configuration of **3aq** was assigned by analogy to that of **3ad**.

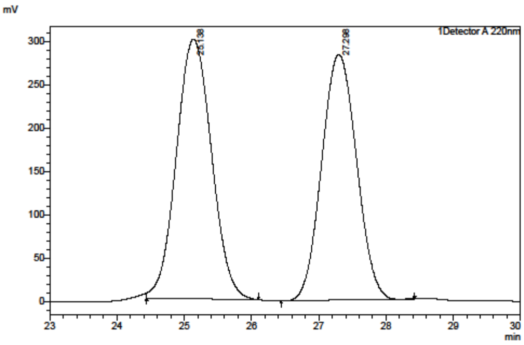

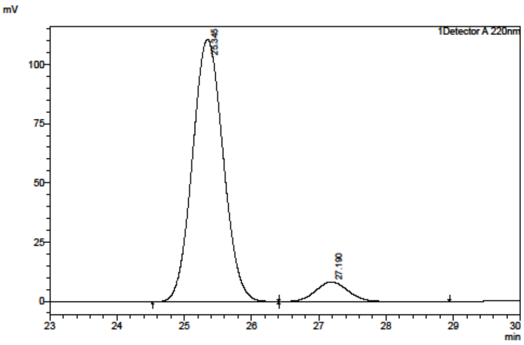

Detector A 220nm

Peak#	Ret. Time	Area%
1	25.138	51.737
2	27.298	48.263
Total		100.000

Detector A 220nm

Peak#	Ret. Time	Area%
1	25.345	92.325
2	27.190	7.675
Total		100.000

Supplementary Figure 17. HPLC Trace of **3aq**.

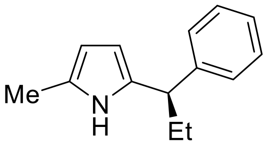

(*R*)-2-Methyl-5-(1-phenylpropyl)-1*H*-pyrrole (**3ar**): 24.8 mg (62% yield). Colorless oil. **^{1}H NMR** (400 MHz, CD_2Cl_2): δ 7.50 – 7.26 (m, 3H), 7.26 – 7.14 (m, 3H), 5.94 (brs, 1H), 5.79 (brs, 1H), 3.72 (dd, *J* = 8.8 Hz, 6.4 Hz, 1H), 2.17 (s, 3H), 2.14 – 2.04 (m, 1H), 1.96 – 1.83 (m, 1H), 0.89 (dd, *J* = 7.4 Hz, 7.4 Hz, 3H); **^{13}C NMR** (126 MHz, CD_2Cl_2): δ 144.9, 134.2, 128.8, 128.3, 126.8, 126.7, 105.8, 105.1, 47.0, 28.7, 13.0, 12.7. **HRMS** (EI-TOF): *m/z* for $[C_{14}H_{17}N]^+$ (M-e$^-$) calcd.: 199.1356; found: 199.1358.

HPLC: Chiralcel OD-H column, hexanes/*i*-PrOH = 90/10, 220 nm, 0.5 mL/min, t_{R1} = 8.6 min (minor), t_{R2} = 9.2 min (major), **ee =** 83%. **Optical Rotation**: $[\alpha]_D^{25}$ = -27.8 (*c* 0.95, CH_2Cl_2). The absolute configuration of **3ar** was assigned by analogy to that of **3ad**.

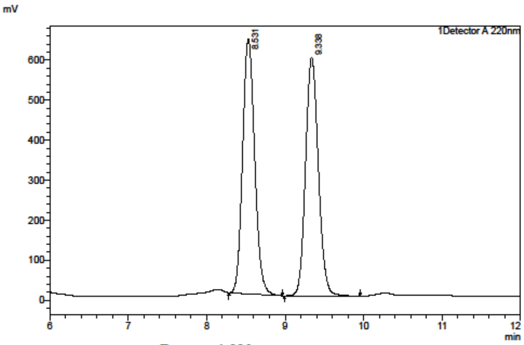

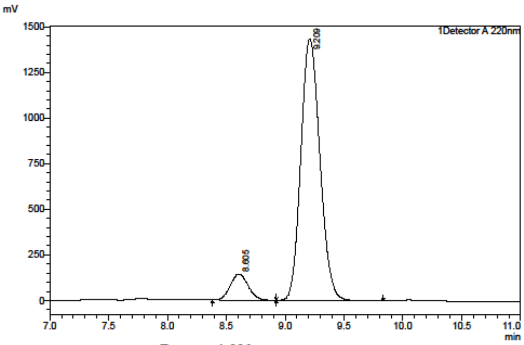

Detector A 220nm

Peak#	Ret. Time	Area%
1	8.531	50.130
2	9.338	49.870
Total		100.000

Detector A 220nm

Peak#	Ret. Time	Area%
1	8.605	8.558
2	9.209	91.442
Total		100.000

Supplementary Figure 18. HPLC Trace of **3ar**.

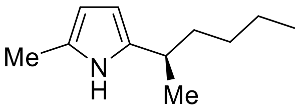

(*R*)-2-(Hexan-2-yl)-5-methyl-1*H*-pyrrole (**3as**): 26.1 mg (79% yield). Colorless oil. **^{1}H NMR** (400 MHz, CD_2Cl_2): δ 7.64 (brs, 1H), 5.84 – 5.45 (m, 2H), 2.78 – 2.54 (m, 1H), 2.21 (s, 3H), 1.59 – 1.51 (m, 1H), 1.49 – 1.42 (m, 1H), 1.34 – 1.23 (m, 4H), 1.19 (d, J = 7.0 Hz, 3H), 0.88 (t, J = 7.0 Hz, 3H); **^{13}C NMR** (126 MHz, CD_2Cl_2): δ 136.9, 125.8, 105.7, 103.8, 37.7, 33.0, 30.1, 23.2, 21.1, 14.3, 13.1. **HRMS** (EI-TOF): *m/z* for $[C_{13}H_{21}N]^+$ (M-e$^-$) calcd.: 165.1512; found: 165.1513.

HPLC: Chiralcel OD-H column, hexanes/*i*-PrOH = 99/1, 210 nm, 0.5 mL/min, t_{R1} = 10.8 min (major), t_{R2} = 11.9 min (minor), **ee =** 82%. **Optical Rotation**: $[\alpha]_D^{25}$ = -13.1 (*c* 0.42, CH_2Cl_2). The absolute configuration of **3as** was assigned by analogy to that of **3ad**.

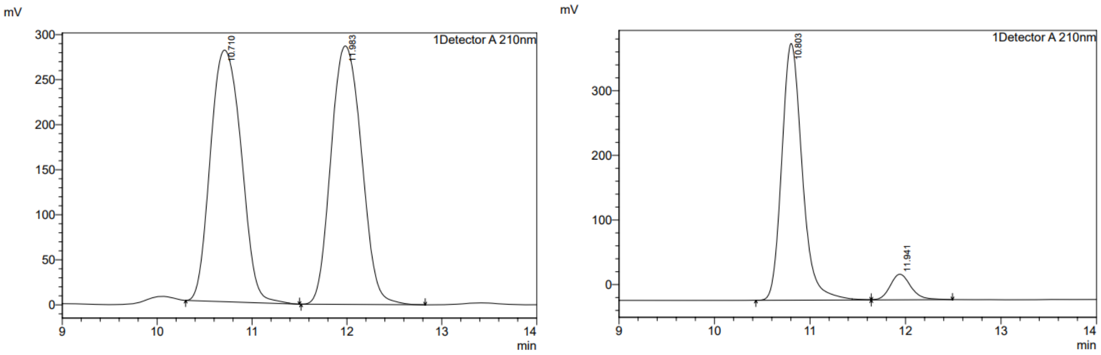

Detector A 210nm

Peak#	Ret. Time	Area%
1	10.710	49.535
2	11.983	50.465
Total		100.000

Detector A 210nm

Peak#	Ret. Time	Area%
1	10.803	90.802
2	11.941	9.198
Total		100.000

Supplementary Figure 19. HPLC Trace of **3as**.

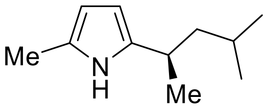

(*R*)-2-Methyl-5-(4-methylpentan-2-yl)-1*H*-pyrrole (**3at**): 24.8 mg (75% yield). Colorless oil. **^{1}H NMR** (400 MHz, CD_2Cl_2): δ 7.64 (brs, 1H), 5.90 – 5.52 (m, 2H), 2.84 – 2.68 (m, 1H), 2.22 (s, 3H), 1.61 – 1.53 (m, 1H), 1.49 – 1.41 (m, 1H), 1.36 – 1.28 (m, 1H), 1.18 (d, J = 6.9 Hz, 3H), 0.90 (d, J = 6.6 Hz, 3H), 0.88 (d, J = 6.6 Hz, 3H); **^{13}C NMR** (126 MHz, CD_2Cl_2): δ 136.9, 125.8, 105.6, 103.8, 47.3, 30.8, 26.0, 22.9, 22.7, 21.7, 13.1. **HRMS** (EI-TOF): *m/z* for $[C_{13}H_{21}N]^+$ (M-e$^-$) calcd.: 165.1512; found: 165.1514.

HPLC: Chiralcel OJ-H column, hexanes/*i*-PrOH = 90/10, 210 nm, 1.0 mL/min, t_{R1} = 5.6 min

(major), t_{R2} = 7.5 min (minor), **ee** = 80%. **Optical Rotation**: $[\alpha]_D^{25}$ = -10.3 (*c* 0.72, CH_2Cl_2). The absolute configuration of **3at** was assigned by analogy to that of **3ad**.

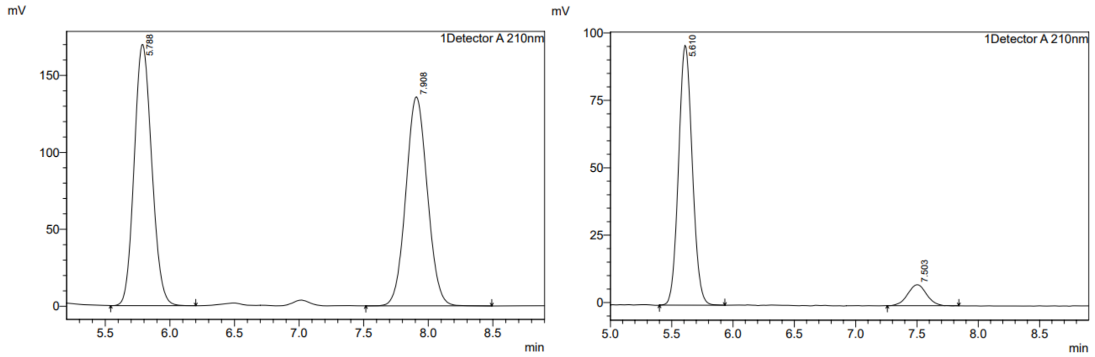

Detector A 210nm

Peak#	Ret. Time	Area%
1	5.788	50.537
2	7.908	49.463
Total		100.000

Detector A 210nm

Peak#	Ret. Time	Area%
1	5.610	90.059
2	7.503	9.941
Total		100.000

Supplementary Figure 20. HPLC Trace of **3at**.

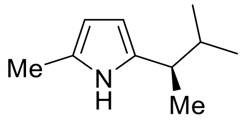

(*R*)-2-Methyl-5-(3-methylbutan-2-yl)-1*H*-pyrrole (**3au**): 24.6 mg (81% yield). Colorless oil. **^{1}H NMR** (400 MHz, CD_2Cl_2): δ 7.61 (brs, 1H), 5.90 – 5.50 (m, 2H), 2.57 – 2.49 (m, 1H), 2.21 (s, 3H), 1.81 – 1.67 (m, 1H), 1.17 (d, *J* = 7.2 Hz, 3H), 0.86 (d, *J* = 6.8 Hz, 3H), 0.84 (d, *J* = 6.8 Hz, 3H); **^{13}C NMR** (126 MHz, CD_2Cl_2): δ 135.5, 125.6, 105.6, 104.8, 39.6, 34.2, 20.5, 19.7, 16.9, 13.1. **HRMS** (EI-TOF): *m/z* for $[C_{13}H_{21}N]^+$ (M-e$^-$) calcd.: 151.1356; found: 151.1353.

HPLC: Chiralcel OJ-H column, hexanes/*i*-PrOH = 90/10, 210 nm, 1.0 mL/min, t_{R1} = 5.5 min (major), t_{R2} = 6.4 min (minor), **ee** = 81%. **Optical Rotation**: $[\alpha]_D^{25}$ = -19.6 (*c* 0.62, CH_2Cl_2). The absolute configuration of **3au** was assigned by analogy to that of **3ad**.

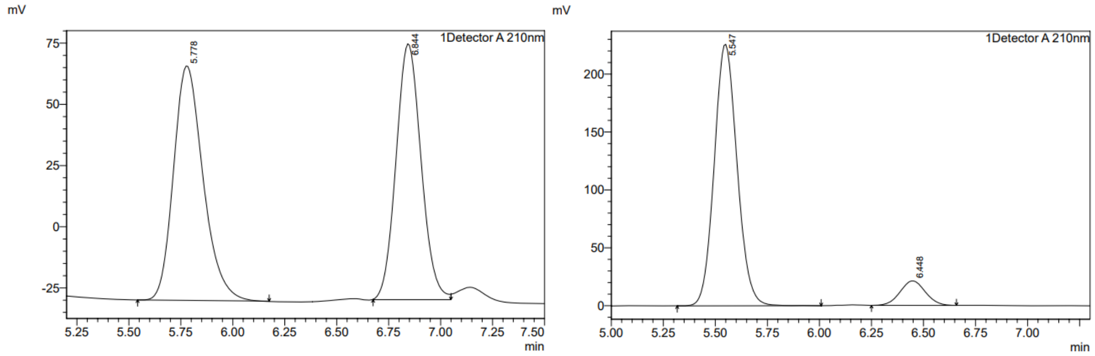

Detector A 210nm

Peak#	Ret. Time	Area%
1	5.778	51.953
2	6.844	48.047
Total		100.000

Detector A 210nm

Peak#	Ret. Time	Area%
1	5.547	90.614
2	6.448	9.386
Total		100.000

Supplementary Figure 21. HPLC Trace of **3au**.

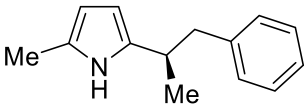

(*R*)-2-Methyl-5-(1-phenylpropan-2-yl)-1*H*-pyrrole (**3av**): 23.4 mg (59% yield). Colorless oil. **^{1}H NMR** (400 MHz, CD_2Cl_2): δ 7.52 (brs, 1H), 7.35 – 7.24 (m, 2H), 7.24 – 7.16 (m, 1H), 7.16 – 7.08 (m, 2H), 5.88 – 5.72 (m, 1H), 5.72 – 5.58 (m, 1H), 3.06 – 2.96 (m, 1H), 2.93 (dd, *J* = 13.2 Hz, 6.7 Hz, 1H), 2.73 (dd, *J* = 13.2 Hz, 7.7 Hz, 1H), 2.18 (s, 3H), 1.21 (d, *J* = 6.8 Hz, 3H); **^{13}C NMR** (126 MHz, CD_2Cl_2): δ 141.2, 136.0, 129.5, 128.5, 126.3, 126.1, 105.7, 104.0, 44.5, 34.9, 20.3, 13.0. **HRMS** (EI-TOF): *m/z* for $[C_{14}H_{17}N]^+$ (M-e$^-$) calcd.: 199.1356; found: 199.1358.

HPLC: Chiralcel OJ-H column, hexanes/*i*-PrOH = 90/10, 220 nm, 1.0 mL/min, t_{R1} = 13.56 min (major), t_{R2} = 20.64 min (minor), **ee =** 83%. **Optical Rotation**: $[\alpha]_D^{25}$ = -37.9 (*c* 0.95, CH_2Cl_2). The absolute configuration of **3av** was assigned by analogy to that of **3ad**.

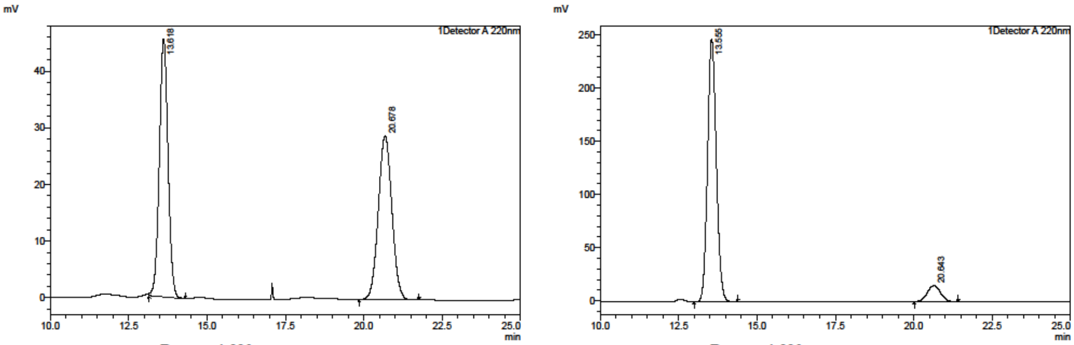

Detector A 220nm

Peak#	Ret. Time	Area%
1	13.618	50.360
2	20.678	49.640
Total		100.000

Detector A 220nm

Peak#	Ret. Time	Area%
1	13.555	91.425
2	20.643	8.575
Total		100.000

Supplementary Figure 22. HPLC Trace of **3av**.

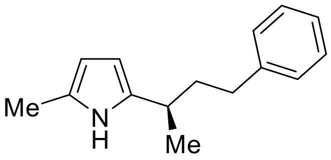

(*R*)-2-Methyl-5-(4-phenylbutan-2-yl)-1*H*-pyrrole (**3aw**): 21.3 mg (50% yield). Colorless oil. **^{1}H NMR** (400 MHz, CD_2Cl_2): δ 7.65 (brs, 1H), 7.31 – 7.23 (m, 2H), 7.23 – 7.09 (m, 3H), 5.80 – 5.73 (m, 1H), 5.73 – 5.64 (m, 1H), 2.77 – 2.68 (m, 1H), 2.64 – 2.55 (m, 2H), 2.22 (s, 3H), 1.95 – 1.75 (m, 2H), 1.26 (d, *J* = 7.0 Hz, 3H); **^{13}C NMR** (126 MHz, CD_2Cl_2): δ 143.1, 136.2, 128.8, 128.7, 126.0, 105.8, 104.1, 39.6, 34.0, 32.7, 21.2, 13.1. **HRMS** (EI-TOF): *m/z* for $[C_{15}H_{19}N]^+$ (M-e$^-$) calcd.: 213.1512; found: 213.1514.

HPLC: Chiralcel OJ-H column, hexanes/*i*-PrOH = 90/10, 220 nm, 1.0 mL/min, t_{R1} = 11.9 min

(major), t_{R2} = 13.8 min (minor), **ee =** 83%. **Optical Rotation**: $[\alpha]_D^{25}$ = -17.2 (*c* 0.96, CH_2Cl_2). The absolute configuration of **3aw** was assigned by analogy to that of **3ad**.

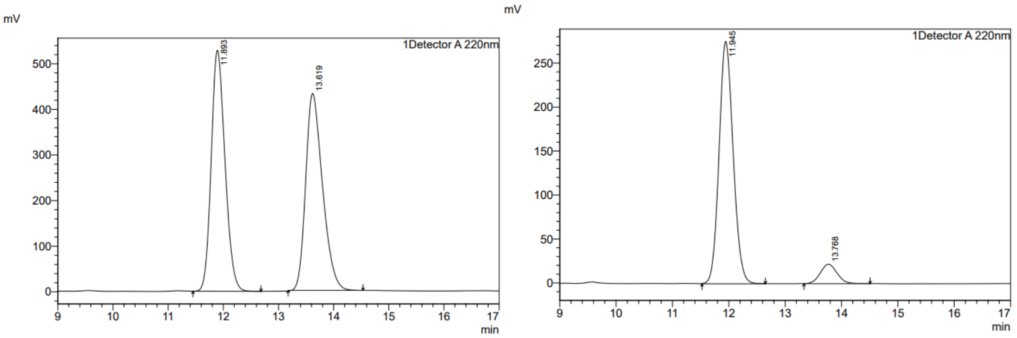

Detector A 220nm

Peak#	Ret. Time	Area%
1	11.893	50.035
2	13.619	49.965
Total		100.000

Detector A 220nm

Peak#	Ret. Time	Area%
1	11.945	91.269
2	13.768	8.731
Total		100.000

Supplementary Figure 23. HPLC Trace of **3aw**.

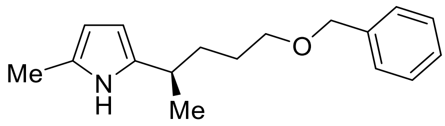

(*R*)-2-(5-(Benzyloxy)pentan-2-yl)-5-methyl-1*H*-pyrrole (**3ax**): 43.2 mg (84% yield). Colorless oil. **^{1}H NMR** (400 MHz, CD_2Cl_2): δ 7.79 (brs, 1H), 7.45 – 7.15 (m, 5H), 5.78 – 5.54 (m, 2H), 4.47 (s, 2H), 3.52 – 3.40 (m, 2H), 2.80 – 2.64 (m, 1H), 2.17 (s, 3H), 1.64 – 1.53 (m, 4H), 1.21 (d, *J* = 7.0 Hz, 3H); **^{13}C NMR** (126 MHz, CD_2Cl_2): δ 139.3, 136.4, 128.7, 128.0, 127.8, 125.9, 105.7, 103.8, 73.2, 71.1, 34.8, 32.8, 28.0, 21.1, 13.0. **HRMS** (EI-TOF): *m/z* for $[C_{15}H_{19}N]^+$ (M-e^-) calcd.: 257.1774; found: 257.1776.

HPLC: Chiralcel OJ-H column, hexanes/*i*-PrOH = 90/10, 210 nm, 1.0 mL/min, t_{R1} = 16.2 min (major), t_{R2} = 17.5 min (minor), **ee =** 72%. **Optical Rotation**: $[\alpha]_D^{25}$ = -7.5 (*c* 0.36, CH_2Cl_2). The absolute configuration of **3ax** was assigned by analogy to that of **3ad**.

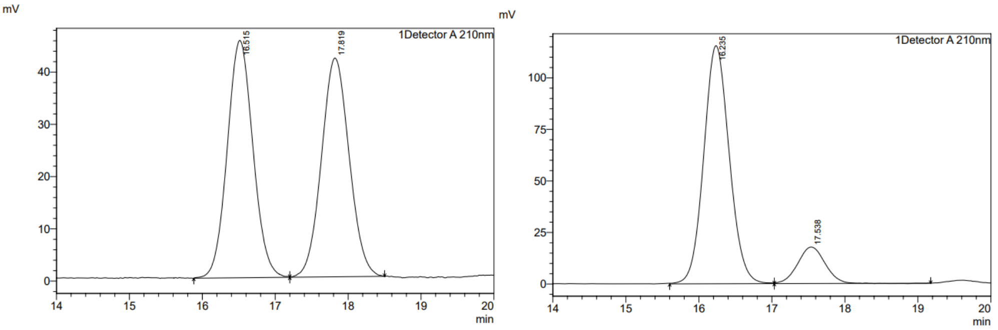

Detector A 210nm

Peak#	Ret. Time	Area%
1	16.515	50.185
2	17.819	49.815
Total		100.000

Detector A 210nm

Peak#	Ret. Time	Area%
1	16.235	86.083
2	17.538	13.917
Total		100.000

Supplementary Figure 24. HPLC Trace of **3ax**.

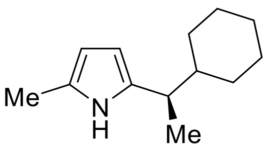

(*R*)-2-(1-Cyclohexylethyl)-5-methyl-1*H*-pyrrole (**3ay**): 19.6 mg (51% yield). Colorless oil. **^{1}H NMR** (400 MHz, CD_2Cl_2): δ 7.60 (brs, 1H), 5.90 – 5.41 (m, 2H), 2.55 – 2.45 (m, 1H), 2.21 (s, 3H), 1.75 – 1.58 (m, 4H), 1.38 – 0.86 (m, 10H); **^{13}C NMR** (126 MHz, CD_2Cl_2): δ 135.6, 125.5, 105.6, 104.7, 44.5, 39.0, 31.3, 30.7, 27.08, 27.06, 27.03, 17.4, 13.1. **HRMS** (EI-TOF): *m/z* for $[C_{13}H_{21}N]^+$ (M-e$^-$) calcd.: 191.1669; found: 191.1667.

HPLC: Chiralcel OD-H column, hexanes/*i*-PrOH = 99/1, 210 nm, 0.5 mL/min, t_{R1} = 10.9 min (major), t_{R2} = 12.3 min (minor), **ee =** 88%. **Optical Rotation**: $[\alpha]_D^{25}$ = -17.0 (*c* 0.93, CH_2Cl_2). The absolute configuration of **3ay** was assigned by analogy to that of **3ad**.

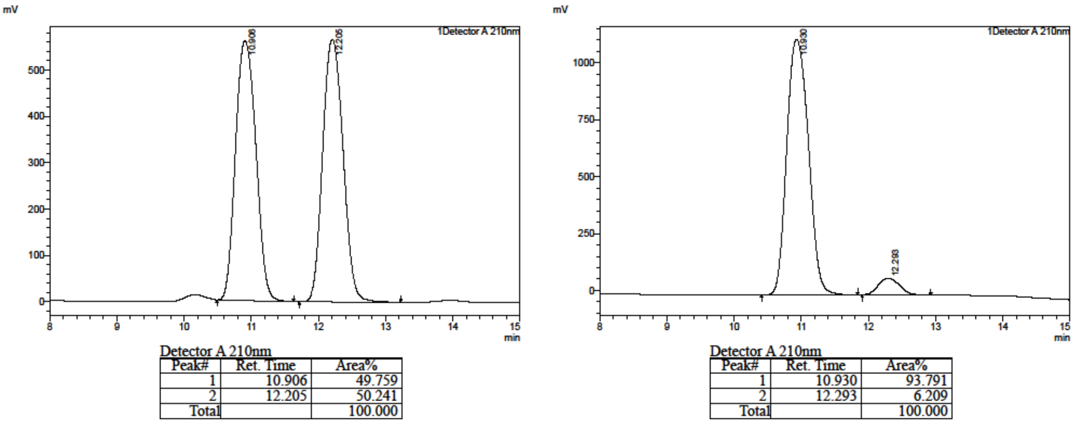

Detector A 210nm

Peak#	Ret. Time	Area%
1	10.906	49.759
2	12.205	50.241
Total		100.000

Detector A 210nm

Peak#	Ret. Time	Area%
1	10.930	93.791
2	12.293	6.209
Total		100.000

Supplementary Figure 25. HPLC Trace of **3ay**.

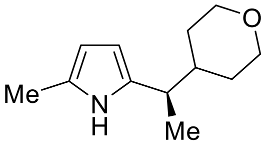

(*R*)-2-Methyl-5-(1-(tetrahydro-2*H*-pyran-4-yl)ethyl)-1*H*-pyrrole (**3az**): 34.7 mg (90% yield). Colorless oil. **^{1}H NMR** (500 MHz, CD_2Cl_2): δ 7.68 (brs, 1H), 5.78 – 5.59 (m, 2H), 3.96 – 3.90 (m, 1H), 3.90 – 3.84 (m, 1H), 3.35 – 3.24 (m, 2H), 2.55 – 2.39 (m, 1H), 2.21 (d, *J* = 0.7 Hz, 3H), 1.64 – 1.58 (m, 1H), 1.58 – 1.50 (m, 1H), 1.42 – 1.36 (m, 1H), 1.32 – 1.22 (m, 2H), 1.20 (d, *J* = 7.2 Hz, 3H); **^{13}C NMR** (126 MHz, CD_2Cl_2): δ 134.6, 125.8, 105.8, 104.9, 68.5, 68.4, 41.9, 38.8, 31.6, 31.1, 17.5, 13.1. **HRMS** (EI-TOF): *m/z* for $[C_{13}H_{21}N]^+$ (M-e$^-$) calcd.: 193.1461; found: 193.1462.

HPLC: Chiralcel OD-H column, hexanes/*i*-PrOH = 90/10, 210 nm, 0.3 mL/min, t_{R1} = 18.9 min

(major), t_{R2} = 19.8 min (minor), **ee =** 79%. **Optical Rotation**: $[\alpha]_D^{25}$ = -18.8 (*c* 0.47, CH_2Cl_2). The absolute configuration of **3az** was assigned by analogy to that of **3ad**.

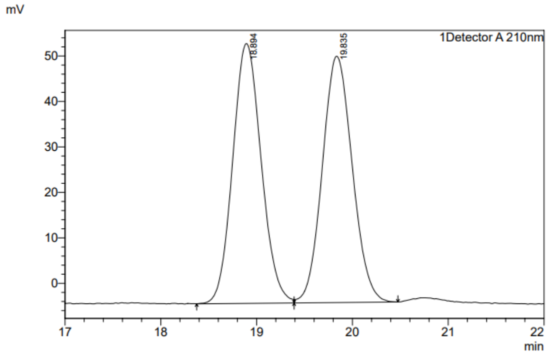

Detector A 210nm

Peak#	Ret. Time	Area%
1	18.894	50.077
2	19.835	49.923
Total		100.000

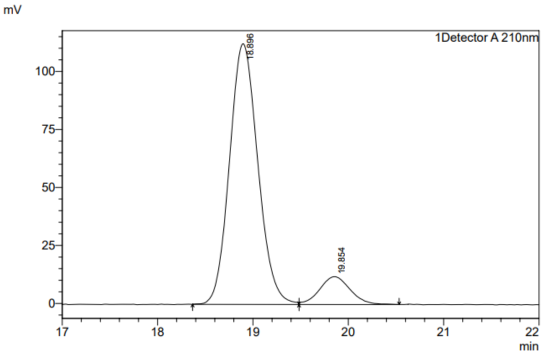

Detector A 210nm

Peak#	Ret. Time	Area%
1	18.896	89.673
2	19.854	10.327
Total		100.000

Supplementary Figure 26. HPLC Trace of **3az**.

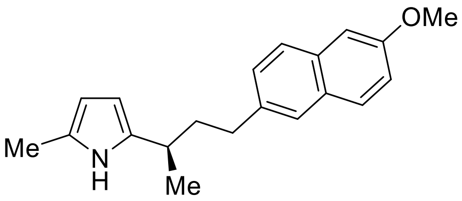

(*R*)-2-(4-(6-Methoxynaphthalen-2-yl)butan-2-yl)-5-methyl-1*H*-pyrrole (**3aA**): 47.5 mg (81% yield). Colorless oil. **^{1}H NMR** (500 MHz, CD_2Cl_2): δ 7.75 – 7.57 (m, 3H), 7.54 (d, *J* = 0.7 Hz, 1H), 7.30 (dd, *J* = 8.3 Hz, 1.7 Hz, 1H), 7.16 – 7.08 (m, 2H), 5.79 – 5.76 (m, 1H), 5.76 – 5.70 (m, 1H), 3.90 (s, 3H), 2.79 – 2.70 (m, 3H), 2.22 (d, *J* = 0.7 Hz, 3H), 2.02 – 1.83 (m, 2H), 1.28 (d, *J* = 7.0 Hz, 3H); **^{13}C NMR** (126 MHz, CD_2Cl_2): δ 157.6, 138.2, 136.2, 133.4, 129.5, 129.2, 128.3, 127.0, 126.5, 126.0, 118.9, 106.0, 105.8, 104.1, 55.6, 39.5, 33.9, 32.7, 21.2, 13.1. **HRMS** (EI-TOF): *m/z* for $[C_{13}H_{21}N]^+$ (M-e$^-$) calcd.: 293.1774; found: 293.177.

HPLC: Chiralcel OD-H column, hexanes/*i*-PrOH = 90/10, 254 nm, 0.3 mL/min, t_{R1} = 27.0 min (major), t_{R2} = 28.2 min (minor), **ee =** 80%. **Optical Rotation**: $[\alpha]_D^{25}$ = -22.4 (*c* 0.70, CH_2Cl_2). The absolute configuration of **3aA** was assigned by analogy to that of **3ad**.

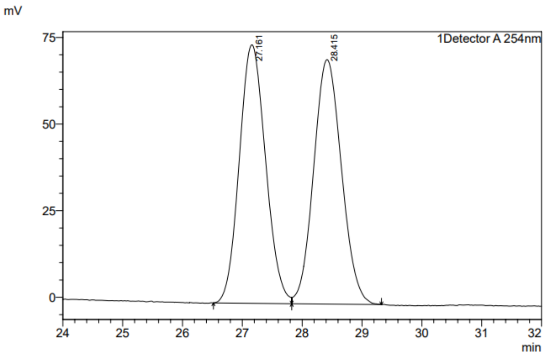

Detector A 254nm

Peak#	Ret. Time	Area%
1	27.161	49.937
2	28.415	50.063
Total		100.000

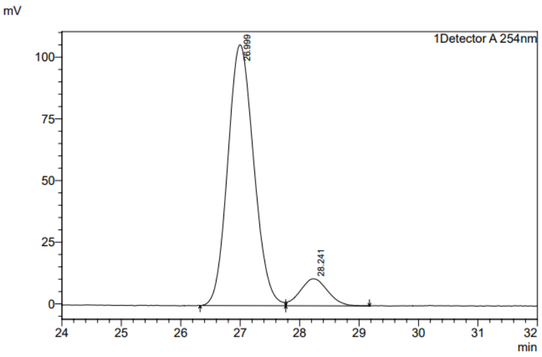

Detector A 254nm

Peak#	Ret. Time	Area%
1	26.999	90.140
2	28.241	9.860
Total		100.000

Supplementary Figure 27. HPLC Trace of **3aA**.

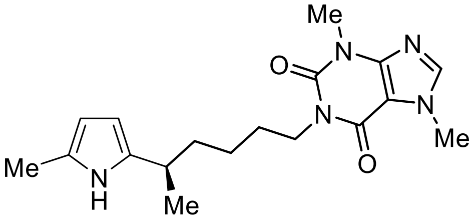

(*R*)-3,7-Dimethyl-1-(5-(5-methyl-1*H*-pyrrol-2-yl)hexyl)-3,7-dihydro-1*H*-purine-2,6-dione (**3aB**): 58.4 mg (85% yield). Colorless oil. **^{1}H NMR** (400 MHz, CD_2Cl_2): δ 7.97 (brs, 1H), 7.49 (d, J = 0.5 Hz, 1H), 5.78 – 5.48 (m, 2H), 3.97 – 3.92 (m, 5H), 3.52 (s, 3H), 2.77 – 2.63 (m, 1H), 2.22 (s, 3H), 1.69 – 1.57 (m, 3H), 1.55 – 1.45 (m, 1H), 1.38 – 1.31 (m, 2H), 1.19 (d, J = 6.9 Hz, 3H); **^{13}C NMR** (126 MHz, CD_2Cl_2): δ 155.6, 151.9, 149.1, 141.9, 136.6, 126.0, 108.0, 105.6, 103.7, 41.3, 37.4, 33.8, 32.7, 29.8, 28.1, 25.0, 20.8, 13.1. **HRMS** (ESI-TOF): *m/z* for $[C_{18}H_{26}N_5O_2]^+$ (M+H$^+$) calcd.: 344.2081; found: 344.2080.

HPLC: Chiralcel AD-H column, hexanes/*i*-PrOH = 90/10, 230 nm, 1.0 mL/min, t_{R1} = 24.7 min (minor), t_{R2} = 27.5 min (major), **ee** = 86%. **Optical Rotation**: $[\alpha]_D^{25}$ = -23.9 (c 0.51, CH_2Cl_2). The absolute configuration of **3aB** was assigned by analogy to that of **3ad**.

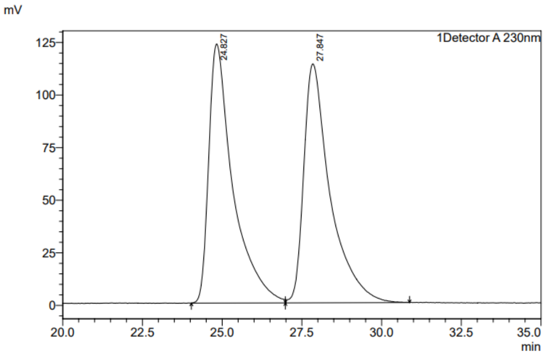

Peak#	Ret. Time	Area%
1	24.827	49.823
2	27.847	50.177
Total		100.000

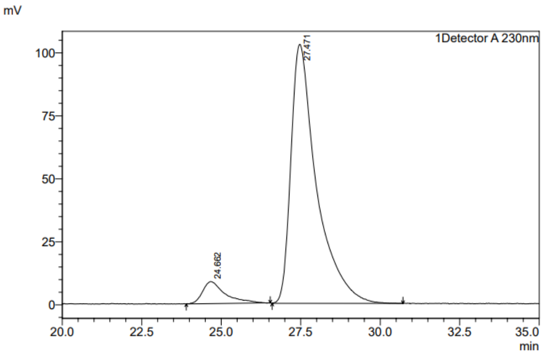

Peak#	Ret. Time	Area%
1	24.662	6.954
2	27.471	93.046
Total		100.000

Supplementary Figure 28. HPLC Trace of **3aB**.

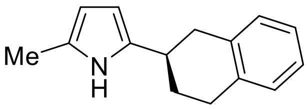

(*R*)-2-Methyl-5-(1,2,3,4-tetrahydronaphthalen-2-yl)-1*H*-pyrrole (**3aC**): 27.4 mg (65% yield). White solid. **^{1}H NMR** (400 MHz, CD_2Cl_2): δ 7.78 (brs, 1H), 7.16 – 7.07 (m, 4H), 5.84 – 5.79 (m, 1H), 5.79 – 5.70 (m, 1H), 3.14 – 3.05 (m, 1H), 3.05 – 2.96 (m, 1H), 2.95 – 2.85 (m, 3H), 2.25 (s, 3H), 2.23 – 2.17 (m, 1H), 1.90 – 1.71 (m, 1H); **^{13}C NMR** (126 MHz, CD_2Cl_2): δ 136.7, 136.6, 135.6, 129.4, 129.2, 126.4, 126.1, 126.0, 105.9, 103.9, 36.4, 33.9, 30.4, 29.5, 13.1. **HRMS** (EI-TOF): *m/z* for $[C_{15}H_{17}N]^+$ (M-e$^-$) calcd.: 211.1356; found: 211.1355.

HPLC: Chiralcel OJ-H column, hexanes/*i*-PrOH = 90/10, 220 nm, 1.0 mL/min, t_{R1} = 24.7 min (major), t_{R2} = 27.8 min (minor), **ee =** 32%. **Optical Rotation**: $[\alpha]_D^{25}$ = 15.2 (*c* 1.00, CH_2Cl_2). The absolute configuration of **3aC** was assigned by analogy to that of **3ad**.

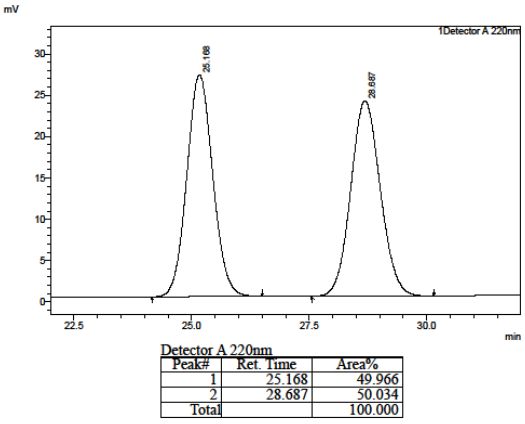

Peak#	Ret. Time	Area%
1	25.168	49.966
2	28.687	50.034
Total		100.000

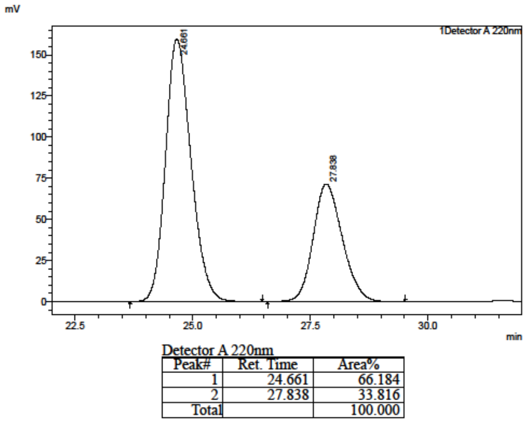

Peak#	Ret. Time	Area%
1	24.661	66.184
2	27.838	33.816
Total		100.000

Supplementary Figure 29. HPLC Trace of **3aC**.

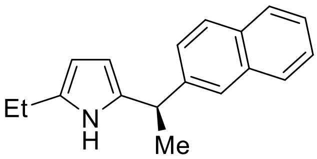

(*R*)-2-Ethyl-5-(1-(naphthalen-2-yl)ethyl)-1*H*-pyrrole (**3bp**): 45.3 mg (91% yield). Colorless oil. **^{1}H NMR** (400 MHz, CD_2Cl_2): δ 7.88 – 7.74 (m, 3H), 7.67 (d, *J* = 0.9 Hz, 1H), 7.65 – 7.38 (m, 3H), 7.34 (dd, *J* = 8.5 Hz, 1.8 Hz, 1H), 6.01 – 5.88 (m, 1H), 5.88 – 5.68 (m, 1H), 4.21 (q, *J* = 7.1 Hz, 1H), 2.49 (q, *J* = 7.6 Hz, 2H), 1.66 (d, *J* = 7.1 Hz, 3H), 1.15 (t, *J* = 7.6 Hz, 3H); **^{13}C NMR** (126 MHz, CD_2Cl_2): δ 144.0, 134.7, 134.1, 134.0, 132.8, 128.6, 128.02, 127.97, 126.5, 126.4, 125.9, 125.8, 105.1, 104.2, 39.3, 21.5, 21.2, 13.9. **HRMS** (EI-TOF): *m/z* for $[C_{18}H_{19}N]^+$ (M-e$^-$) calcd.: 249.1512; found: 249.1513.

HPLC: Chiralcel OJ-H column, hexanes/*i*-PrOH = 90/10, 254 nm, 1.0 mL/min, t_{R1} = 16.0 min (major), t_{R2} = 24.6 min (minor), **ee =** 93%. **Optical Rotation**: $[\alpha]_D^{25}$ = -30.7(*c* 1.11, CH_2Cl_2). The absolute configuration of **3bp** was assigned by analogy to that of **3ad**.

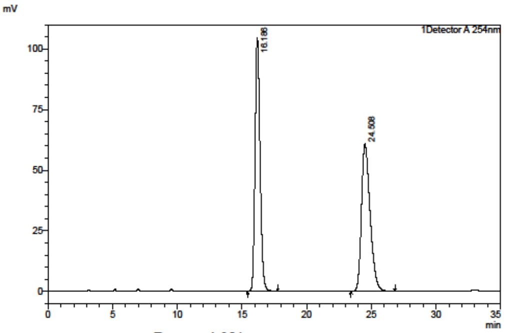

Detector A 254nm

Peak#	Ret. Time	Area%
1	16.186	49.992
2	24.508	50.008
Total		100.000

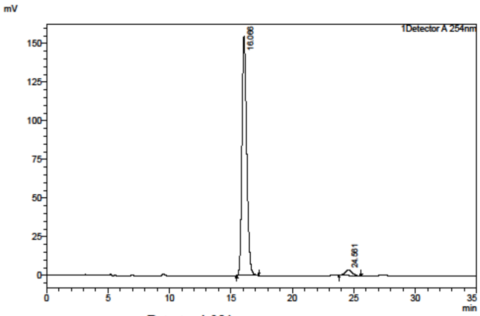

Detector A 254nm

Peak#	Ret. Time	Area%
1	16.066	96.684
2	24.561	3.316
Total		100.000

Supplementary Figure 30. HPLC Trace of **3bp**.

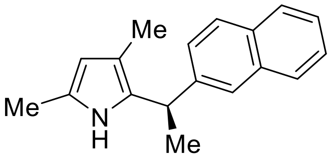

(*R*)-3,5-Dimethyl-2-(1-(naphthalen-2-yl)ethyl)-1*H*-pyrrole (**3cp**): 46.2 mg (93% yield). Colorless oil. **^{1}H NMR** (400 MHz, $CDCl_3$): δ 7.92 – 7.75 (m, 3H), 7.72 (s, 1H), 7.60 – 7.44 (m, 2H), 7.37 (dd, *J* = 8.5 Hz, 1.6 Hz, 1H), 7.27 (brs, 1H), 5.89 – 5.62 (m, 1H), 4.41 (q, *J* = 7.2 Hz, 1H), 2.18 (s, 3H), 2.08 (s, 3H), 1.71 (d, *J* = 7.2 Hz, 3H); **^{13}C NMR** (100 MHz, $CDCl_3$): δ 142.6, 133.5, 132.2, 129.5, 128.1, 127.7, 127.6, 126.7, 126.0, 125.4, 125.2, 124.7, 113.9, 108.1, 36.3, 20.5, 12.9, 11.1. **HRMS** (EI-TOF): *m/z* for $[C_{18}H_{19}N]^+$ (M-e$^-$) calcd.: 249.1512; found: 249.1508.

HPLC: Chiralcel OD-H column, hexanes/*i*-PrOH = 98/2, 254 nm, 0.5 mL/min, t_{R1} = 15.0 min (major), t_{R2} = 16.7 min (minor), **ee =** 83%. **Optical Rotation**: $[\alpha]_D^{25}$ = -71.8 (*c* 1.12, CH_2Cl_2). The absolute configuration of **3cp** was assigned by analogy to that of **3ad**.

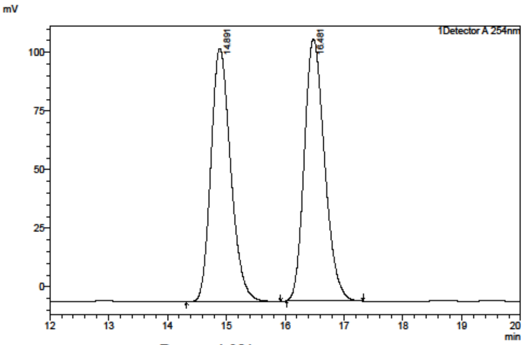

Detector A 254nm

Peak#	Ret. Time	Area%
1	14.891	48.596
2	16.481	51.404
Total		100.000

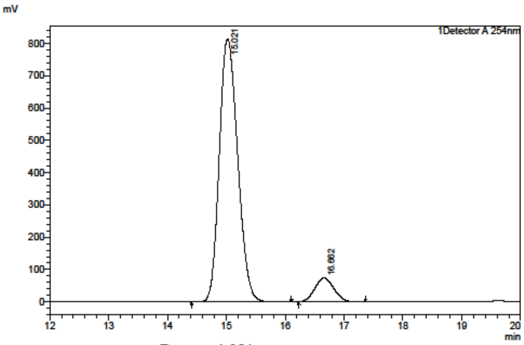

Detector A 254nm

Peak#	Ret. Time	Area%
1	15.021	91.366
2	16.662	8.634
Total		100.000

Supplementary Figure 31. HPLC Trace of **3cp**.

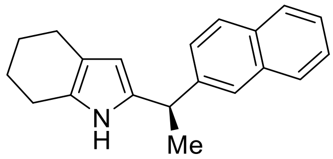

(*R*)-2-(1-(Naphthalen-2-yl)ethyl)-4,5,6,7-tetrahydro-1*H*-indole (**3dp**): 42.1 mg (76% yield). Colorless oil. **^{1}H NMR** (400 MHz, CD_2Cl_2): δ 7.88 – 7.77 (m, 3H), 7.71 (d, *J* = 1.0 Hz, 1H), 7.55 – 7.43 (m, 2H), 7.37 (dd, *J* = 8.5 Hz, 1.8 Hz, 1H), 7.32 (brs, 1H), 5.89 – 5.62 (m, 1H), 4.19 (q, *J* = 7.1 Hz, 1H), 2.51 – 2.40 (m, 4H), 1.79 – 1.70 (m, 4H), 1.66 (d, *J* = 7.1 Hz, 3H); **^{13}C NMR** (126 MHz, CD_2Cl_2): δ 143.8, 134.5, 134.0, 132.8, 128.5, 128.0, 127.9, 126.5, 126.4, 126.3, 125.7, 125.7, 116.8, 104.4, 39.3, 24.4, 24.0, 23.4, 23.0, 21.4. **HRMS** (EI-TOF): *m/z* for $[C_{20}H_{21}N]^+$ ($M-e^-$) calcd.: 275.1669; found: 275.1669.

HPLC: Chiralcel OJ-H column, hexanes/*i*-PrOH = 90/10, 254 nm, 1.0 mL/min, t_{R1} = 14.7 min (minor), t_{R2} = 17.3 min (major), **ee =** 87%. **Optical Rotation**: $[\alpha]_D^{25}$ = -46.9 (*c* 0.26, CH_2Cl_2). The absolute configuration of **3dp** was assigned by analogy to that of **3ad**.

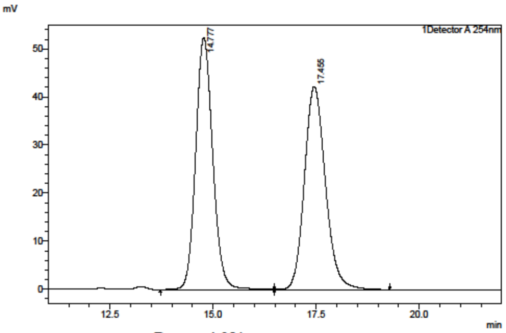

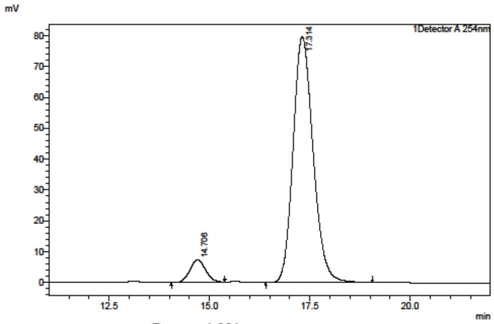

Detector A 254nm

Peak#	Ret. Time	Area%
1	14.777	50.090
2	17.455	49.910
Total		100.000

Detector A 254nm

Peak#	Ret. Time	Area%
1	14.706	6.658
2	17.314	93.342
Total		100.000

Supplementary Figure 32. HPLC Trace of **3dp**.

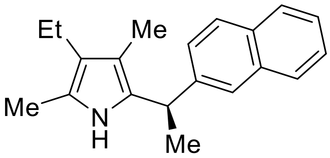

(*R*)-3-Ethyl-2,4-dimethyl-5-(1-(naphthalen-2-yl)ethyl)-1*H*-pyrrole (**3ep**): 48.0 mg (87% yield). (Colorless oil) **^{1}H NMR** (400 MHz, CD_2Cl_2): δ 7.90 – 7.77 (m, 3H), 7.74 (d, *J* = 0.7 Hz, 1H), 7.55 – 7.41 (m, 2H), 7.37 (dd, *J* = 8.5 Hz, 1.8 Hz, 1H), 7.25 (brs, 1H), 4.40 (q, *J* = 7.2 Hz, 1H), 2.39 (q, *J* = 7.5 Hz, 2H), 2.10 (s, 3H), 2.01 (s, 3H), 1.69 (d, *J* = 7.2 Hz, 3H), 1.09 (t, *J* = 7.5 Hz, 3H); **^{13}C NMR** (100 MHz, CD_2Cl_2): δ 143.5, 134.1, 132.6, 128.7, 128.4, 128.0, 127.9, 127.1, 126.4, 125.8, 124.9, 121.12, 121.09, 113.0, 36.7, 20.7, 18.0, 16.0, 11.1, 9.3. **HRMS** (EI-TOF): *m/z* for $[C_{20}H_{23}N]^{+}$ (M-e^{-}) calcd.: 277.1825; found: 277.1817.

HPLC: Chiralcel OJ-H column, hexanes/*i*-PrOH = 90/10, 254 nm, 1.0 mL/min, t_{R1} = 16.4 min (major), t_{R2} = 25.5 min (minor), **ee** = 75%. **Optical Rotation**: $[\alpha]_D^{25}$ = 22.2 (*c* 0.18, EtOAc). The absolute configuration of **3ep** was assigned by analogy to that of **3ad**.

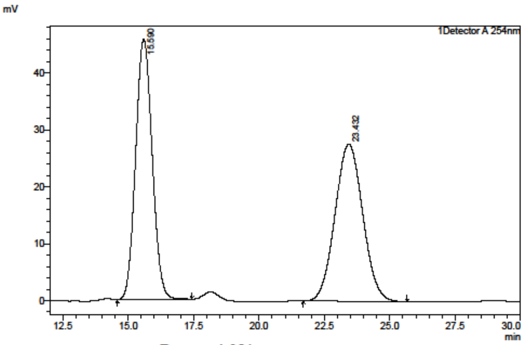

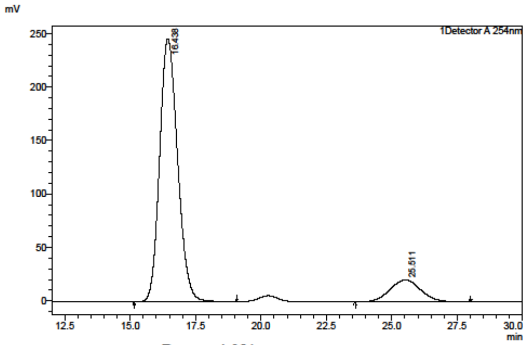

Detector A 254nm

Peak#	Ret. Time	Area%
1	15.590	49.741
2	23.432	50.259
Total		100.000

Detector A 254nm

Peak#	Ret. Time	Area%
1	16.438	87.368
2	25.511	12.632
Total		100.000

Supplementary Figure 33. HPLC Trace of **3ep**.

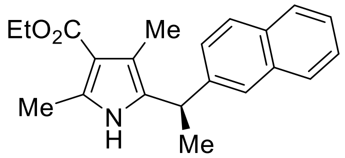

Ethyl (*R*)-2,4-dimethyl-5-(1-(naphthalen-2-yl)ethyl)-1*H*-pyrrole-3-carboxylate (**3fp**): 45.2 mg (70% yield). White solid. **^{1}H NMR** (400 MHz, CD_2Cl_2): δ 7.87 – 7.63 (m, 5H), 7.53 – 7.39 (m, 2H), 7.31 (dd, *J* = 8.5 Hz, 1.8 Hz, 1H), 4.42 (q, *J* = 7.3 Hz, 1H), 4.21 (q, *J* = 7.1 Hz, 2H), 2.37 (s, 3H), 2.22 (s, 3H), 1.67 (d, *J* = 7.3 Hz, 3H), 1.31 (t, *J* = 7.1 Hz, 3H); **^{13}C NMR** (126 MHz, CD_2Cl_2): δ 166.5, 142.4, 134.3, 134.0, 132.6, 130.3, 128.5, 128.1, 127.9, 126.9, 126.5, 126.0, 125.0, 116.1, 111.4, 59.3, 35.8, 20.3, 14.7, 14.1, 11.1 **HRMS** (EI-TOF): *m/z* for $[C_{21}H_{23}NO_2]^+$ (M-e$^-$) calcd.: 321.1723; found: 321.1720.

HPLC: Chiralcel OJ-H column, hexanes/*i*-PrOH = 80/20, 254 nm, 1.0 mL/min, t_{R1} = 15.5 min (major), t_{R2} = 28.2 min (minor), **ee =** 93%. **Optical Rotation**: $[\alpha]_D^{25}$ = 150.3 (*c* 0.37, CH_2Cl_2). The absolute configuration of **3fp** was assigned by analogy to that of **3ad**.

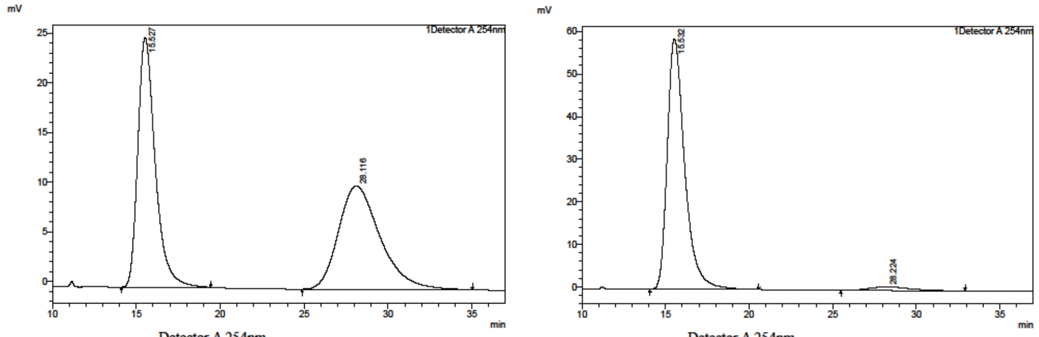

Detector A 254nm

Peak#	Ret. Time	Area%
1	15.527	50.051
2	28.116	49.949
Total		100.000

Detector A 254nm

Peak#	Ret. Time	Area%
1	15.532	96.685
2	28.224	3.315
Total		100.000

Supplementary Figure 34. HPLC Trace of **3fp**.

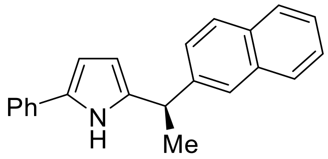

(*R*)-2-(1-(Naphthalen-2-yl)ethyl)-5-phenyl-1*H*-pyrrole (**3gp**): 49.9 mg (84% yield). White solid. **^{1}H NMR** (400 MHz, CD_2Cl_2): δ 8.09 (brs, 1H), 7.87 – 7.78 (m, 3H), 7.73 (d, *J* = 1.0 Hz, 1H), 7.52 – 7.43 (m, 2H), 7.43 – 7.34 (m, 3H), 7.34 – 7.25 (m, 2H), 7.19 – 7.06 (m, 1H), 6.52 – 6.43 (m, 1H), 6.23 – 6.14 (m, 1H), 4.33 (q, *J* = 7.1 Hz, 1H), 1.74 (d, *J* = 7.1 Hz, 3H); **^{13}C NMR** (126 MHz, CD_2Cl_2): δ 143.3, 137.9, 134.0, 133.2, 132.8, 131.6, 129.1, 128.7, 128.0, 128.0, 126.5, 126.4, 126.1, 125.94, 125.91, 123.7, 107.4, 106.2, 39.3, 21.6. **HRMS** (EI-TOF): *m/z* for $[C_{22}H_{19}N]^+$ (M-e$^-$) calcd.:

297.1512; found: 297.1513.

HPLC: Chiralcel OJ-H column, hexanes/*i*-PrOH = 90/10, 254 nm, 1.0 mL/min, t_{R1} = 39.9 min (minor), t_{R2} = 60.8 min (major), **ee =** 88%. **Optical Rotation**: $[\alpha]_D^{25}$ = 89.8 (*c* 0.92, CH_2Cl_2). The absolute configuration of **3gp** was assigned by analogy to that of **3ad**.

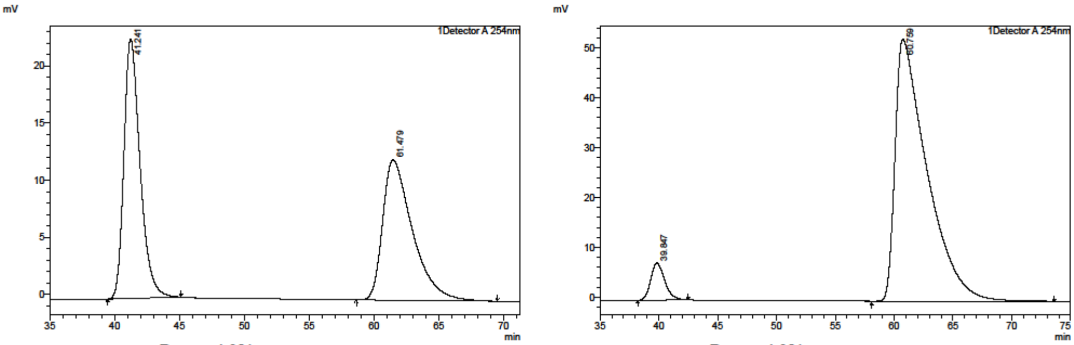

Detector A 254nm

Peak#	Ret. Time	Area%
1	41.241	50.167
2	61.479	49.833
Total		100.000

Detector A 254nm

Peak#	Ret. Time	Area%
1	39.847	5.778
2	60.759	94.222
Total		100.000

Supplementary Figure 35. HPLC Trace of **3gp**.

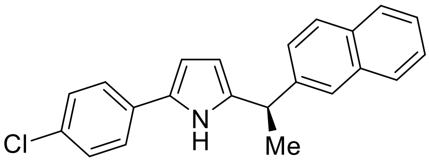

(*R*)-2-(4-Chlorophenyl)-5-(1-(naphthalen-2-yl)ethyl)-1*H*-pyrrole (**3hp**): 47.2 mg (71% yield). White solid. **^{1}H NMR** (400 MHz, CD_2Cl_2): δ 8.04 (brs, 1H), 7.89 – 7.77 (m, 3H), 7.72 (d, *J* = 0.9 Hz, 1H), 7.52 – 7.42 (m, 2H), 7.37 (dd, *J* = 8.5 Hz, 1.8 Hz, 1H), 7.32 – 7.28 (m, 2H), 7.28 – 7.23 (m, 2H), 6.55 – 6.37 (m, 1H), 6.28 – 6.08 (m, 1H), 4.32 (q, *J* = 7.1 Hz, 1H), 1.73 (d, *J* = 7.1 Hz, 3H); **^{13}C NMR** (126 MHz, CD_2Cl_2): δ 143.2, 138.4, 134.1, 132.9, 131.9, 131.5, 130.6, 129.2, 128.8, 128.1, 128.0, 126.6, 126.4, 126.03, 125.97, 125.0, 107.7, 106.9, 39.4, 21.6. **HRMS** (EI-TOF): *m/z* for $[C_{22}H_{18}ClN]^+$ (M-e$^-$) calcd.: 331.1122; found: 331.1121.

HPLC: Chiralcel OD-H column, hexanes/*i*-PrOH = 90/10, 254 nm, 0.5 mL/min, t_{R1} = 18.5 min (major), t_{R2} = 20.5 min (minor), **ee =** 95%. **Optical Rotation**: $[\alpha]_D^{25}$ = 87.7 (*c* 0.83, CH_2Cl_2). The absolute configuration of **3hp** was assigned by analogy to that of **3ad**.

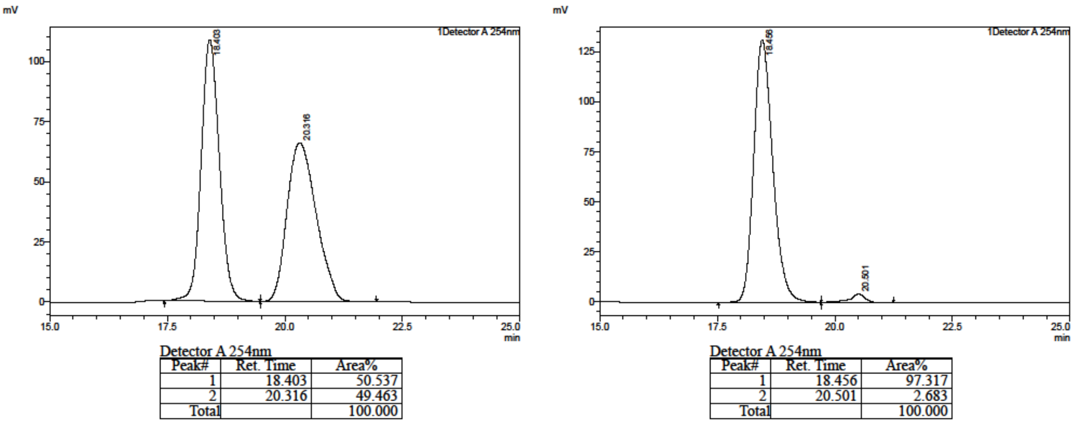

Detector A 254nm

Peak#	Ret. Time	Area%
1	18.403	50.537
2	20.316	49.463
Total		100.000

Detector A 254nm

Peak#	Ret. Time	Area%
1	18.456	97.317
2	20.501	2.683
Total		100.000

Supplementary Figure 36. HPLC Trace of **3hp**.

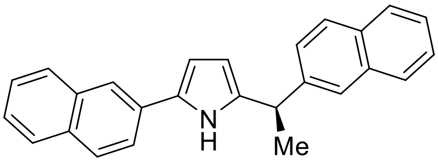

(*R*)-2-(Naphthalen-2-yl)-5-(1-(naphthalen-2-yl)ethyl)-1*H*-pyrrole (**3ip**): 64.0 mg (92% yiel). White solid. **^{1}H NMR** (400 MHz, CD_2Cl_2): δ 8.26 (brs, 1H), 7.89 – 7.82 (m, 3H), 7.81 – 7.68 (m, 5H), 7.61 (dd, *J* = 8.6 Hz, 1.8 Hz, 1H), 7.53 – 7.37 (m, 5H), 6.70 – 6.56 (m, 1H), 6.31 – 6.18 (m, 1H), 4.38 (q, *J* = 7.1 Hz, 1H), 1.77 (d, *J* = 7.1 Hz, 3H); **^{13}C NMR** (126 MHz, CD_2Cl_2): δ 143.4, 138.4, 134.3, 134.1, 132.9, 132.3, 131.7, 130.6, 128.8, 128.08, 128.04, 128.01, 127.9, 126.8, 126.55, 126.47, 126.0, 125.6, 123.4, 120.6, 107.7, 107.1, 39.5, 21.6. **HRMS** (EI-TOF): *m/z* for $[C_{26}H_{21}N]^+$ (M-e$^-$) calcd.: 347.1669; found: 347.1676.

HPLC: Chiralcel OD-H column, hexanes/*i*-PrOH = 90/10, 254 nm, 0.5 mL/min, t_{R1} = 31.9 min (major), t_{R2} = 34.1 min (minor), **ee =** 85%. **Optical Rotation**: $[\alpha]_D^{25}$ = 155.1 (*c* 0.88, CH_2Cl_2). The absolute configuration of **3ip** was assigned by analogy to that of **3ad**.

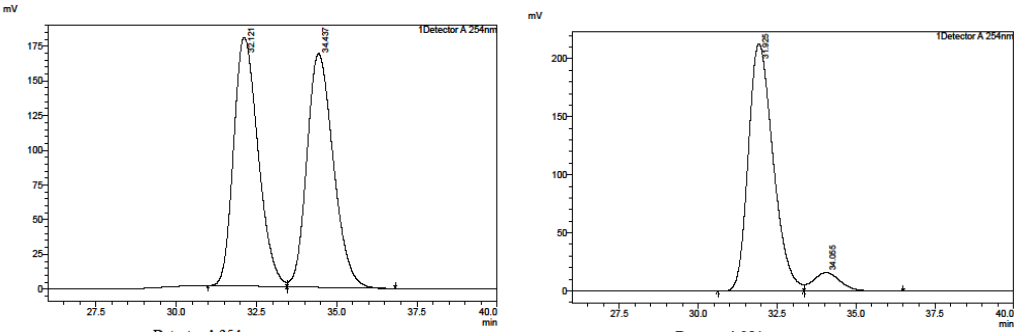

Detector A 254nm

Peak#	Ret. Time	Area%
1	32.121	49.682
2	34.437	50.318
Total		100.000

Detector A 254nm

Peak#	Ret. Time	Area%
1	31.925	92.434
2	34.055	7.566
Total		100.000

Supplementary Figure 37. HPLC Trace of **3ip**.

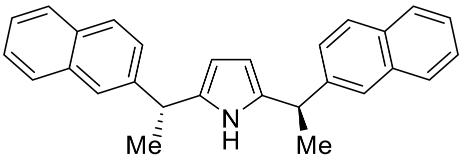

2,5-Bis((*R*)-1-(naphthalen-2-yl)ethyl)-1*H*-pyrrole (**3jp**, major): 37.4 mg (50% yield). Colorless oil. **^{1}H NMR** (400 MHz, CD_2Cl_2): δ 7.83 – 7.77 (m, 2H), 7.77 – 7.69 (m, 4H), 7.56 (s, 2H), 7.51 – 7.32 (m, 5H), 7.28 (dd, *J* = 8.4 Hz, 1.8 Hz, 2H), 6.04 (d, *J* = 2.7 Hz, 2H), 4.11 (q, *J* = 7.1 Hz, 2H), 1.61 (d, *J* = 7.1 Hz, 6H); **^{13}C NMR** (126 MHz, CD_2Cl_2): δ 144.0, 135.7, 134.0, 132.7, 128.5, 127.94, 127.89, 126.37, 126.35, 125.80, 125.76, 105.0, 39.2, 21.6. **HRMS** (EI-TOF): *m/z* for $[C_{28}H_{25}N]^+$ (M-e$^-$) calcd.: 375.1982; found: 375.1987.

HPLC: Chiralcel OD-H column, hexanes/*i*-PrOH = 90/10, 254 nm, 1.0 mL/min, t_{R1} = 5.3 min (minor), t_{R1} = 8.0 min (major), **ee =** 90%. **Optical Rotation**: $[\alpha]_D^{25}$ = 132.4 (*c* 0.85, CH_2Cl_2). The absolute configuration of **3jp** was assigned by analogy to that of **3ad**.

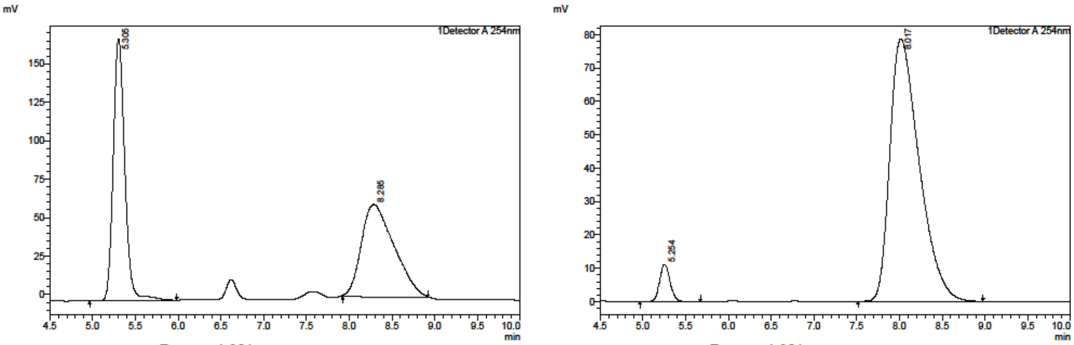

Detector A 254nm

Peak#	Ret. Time	Area%
1	5.305	49.935
2	8.285	50.065
Total		100.000

Detector A 254nm

Peak#	Ret. Time	Area%
1	5.254	4.790
2	8.017	95.210
Total		100.000

Supplementary Figure 38. HPLC Trace of **3jp**.

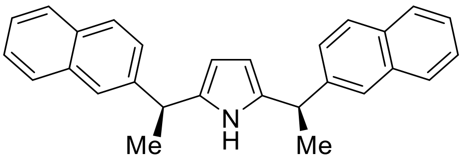

2-((*R*)-1-(Naphthalen-2-yl)ethyl)-5-((*S*)-1-(naphthalen-2-yl)ethyl)-1*H*-pyrrole (**3jp′**, minor, meso isomer): 16.8 mg (22% yield). White solid. **^{1}H NMR** (500 MHz, $CDCl_3$): δ 7.89 – 7.62 (m, 6H), 7.56 (s, 2H), 7.50 – 7.34 (m, 5H), 7.29 (dd, *J* = 8.4 Hz, 1.6 Hz, 2H), 6.06 (d, *J* = 2.5 Hz, 1H), 4.18 (q, *J* = 7.1 Hz, 2H), 1.65 (d, *J* = 7.1 Hz, 6H); **^{13}C NMR** (126 MHz, $CDCl_3$): δ 143.1, 135.3, 133.5, 132.3, 128.1, 127.6, 127.5, 126.1, 125.9, 125.39, 125.37, 104.9, 38.7, 21.5. **HRMS** (EI-TOF): *m/z* for $[C_{28}H_{25}N]^+$ (M-e$^-$) calcd.: 375.1982; found: 375.1974.

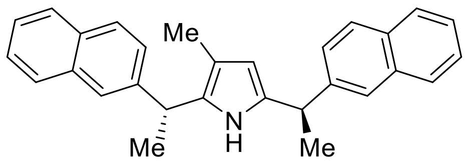

3-Methyl-2,5-bis((*R*)-1-(naphthalen-2-yl)ethyl)-1*H*-pyrrole (**3kp**, major): 44.6 mg (57% yield). Colorless oil. **^{1}H NMR** (500 MHz, $CDCl_3$): δ 7.83 – 7.79 (m, 1H), 7.79 – 7.72 (m, 3H), 7.70 (d, *J* = 8.7 Hz, 2H), 7.60 (s, 1H), 7.53 (s, 1H), 7.48 – 7.40 (m, 4H), 7.37 – 7.28 (m, 2H), 7.27 – 7.24 (m, 1H), 5.94 (d, *J* = 2.4 Hz, 1H), 4.35 (q, *J* = 7.3 Hz, 1H), 4.20 (q, *J* = 7.1 Hz, 1H), 2.07 (s, 3H), 1.66 (d, *J* = 7.1 Hz, 3H), 1.63 (d, *J* = 7.3 Hz, 3H); **^{13}C NMR** (126 MHz, $CDCl_3$): δ 143.3, 142.8, 133.5, 133.43, 133.41, 132.3, 132.0, 130.1, 128.1, 128.0, 127.65, 127.61, 127.6, 127.5, 126.2, 126.1, 125.93, 125.85, 125.5, 125.4, 125.3, 124.7, 113.8, 107.6, 38.8, 36.4, 21.5, 20.7, 11.2. **HRMS** (EI-TOF): *m/z* for $[C_{29}H_{27}N]^+$ (M-e$^-$) calcd.: 389.2138; found: 389.2137.

HPLC: Chiralcel OD-H column, hexanes/*i*-PrOH = 90/10, 254 nm, 1.0 mL/min, t_{R1} = 5.3 min (minor), t_{R2} = 5.8 min (major), **ee =** 95%. **Optical Rotation**: $[\alpha]_D^{25}$ = -64.3 (*c* 1.05, CH_2Cl_2). The absolute configuration of **3kp** was assigned by single crystal X-ray analysis..

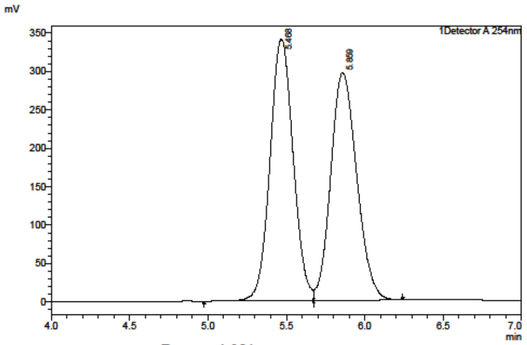

Detector A 254nm

Peak#	Ret. Time	Area%
1	5.468	50.705
2	5.859	49.295
Total		100.000

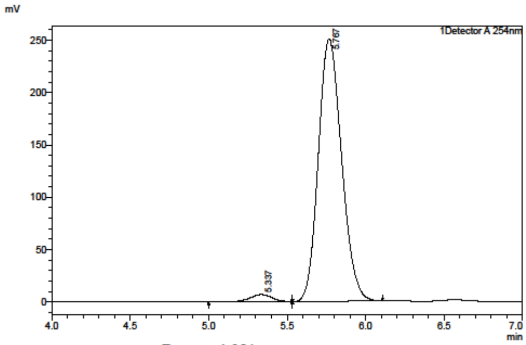

Detector A 254nm

Peak#	Ret. Time	Area%
1	5.337	2.535
2	5.767	97.465
Total		100.000

Supplementary Figure 39. HPLC Trace of **3kp**.

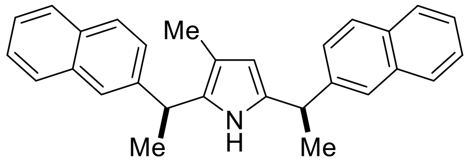

3-Methyl-5-(1-(naphthalen-2-yl)ethyl)-2-(1-(naphthalen-2-yl)ethyl)-1*H*-pyrrole (**3kp′**, minor): 21.1 mg (27% yield). Colorless oil. **^{1}H NMR** (500 MHz, $CDCl_3$): δ 7.88 – 7.70 (m, 6H), 7.62 (s, 1H), 7.61 (s, 1H), 7.52 – 7.42 (m, 4H), 7.34 (dd, *J* = 8.3 Hz, 1.5 Hz, 1H), 7.30 (dd, *J* = 8.4 Hz, 1.7

Hz, 1H), 7.24 (brs, 1H), 5.93 (d, *J* = 2.4 Hz, 1H), 4.36 (q, *J* = 7.2 Hz, 1H), 4.16 (q, *J* = 7.1 Hz, 1H), 2.09 (s, 3H), 1.64 (d, *J* = 7.3 Hz, 3H), 1.59 (d, *J* = 7.2 Hz, 3H); **^{13}C NMR** (126 MHz, $CDCl_3$): δ 143.2, 142.6, 133.53, 133.46, 133.41, 132.3, 132.1, 130.2, 128.2, 128.0, 127.69, 127.64, 127.57, 127.50, 126.5, 126.1, 125.93, 125.90, 125.5, 125.4, 125.3, 124.6, 113.6, 107.4, 38.8, 36.4, 21.5, 20.5, 11.3. **HRMS** (EI-TOF): *m/z* for $[C_{29}H_{27}N]^+$ (M-e$^-$) calcd.: 389.2138; found: 389.2139.

6. Isolation and Reactivity of 7 Related to Heteroarylation of Simple Alcohols

Procedure for the synthesis of alkene 7

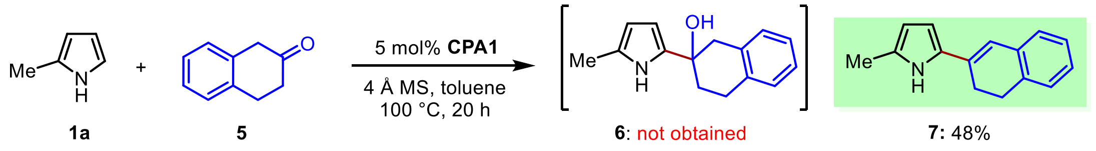

In a nitrogen-filled glove box, a vial was charged with **CPA1** (7.5 mg, 0.010 mmol), 4 Å molecular sieves (20 mg), 2-methylpyrrole (**1a**, 16.2 mg, 0.20 mmol), 2-tetralone (**7**, 58.5 mg, 0.40 mmol) and toluene (0.5 mL). The vial was sealed, taken outside the glovebox, heated to 100 °C and was allowed to stir for 20 h. The resulting mixture was cooled to room temperature, and then purified by column chromatography (silica gel, hexanes/dichloromethane/triethylamine = 3/1/0.03) to provide the alkene **7** as a pale yellow solid 20.0 mg (48% yield).

^{1}H NMR (400 MHz, CD_3OD): δ 7.11-6.96 (m, 4H), 6.62 (s, 1H), 6.16 (d, J = 3.4 Hz, 1H), 5.78 (dd, J = 3.4 Hz, 0.8 Hz, 1H), 2.86-2.77 (m, 2H), 2.61-2.51 (m, 2H), 2.26 (s, 3H); **^{13}C NMR** (126 MHz, CD_3OD): δ 136.8, 135.4, 132.31, 132.27, 131.3, 128.0, 127.4, 126.51, 126.49, 116.6, 108.6, 107.8, 29.2, 26.1, 13.0. **HRMS** (EI-TOF): *m/z* for $[C_{15}H_{15}N]^+$ (M-e$^-$) calcd.: 209.1199; found: 209.1199.

Procedure for transfer hydrogenation of alkene 7

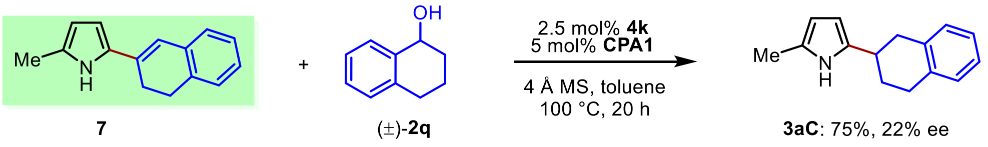

In a nitrogen-filled glove box, a vial was charged with iridium complex (**4k**, 3.1 mg, 0.0050 mmol), **CPA1** (7.5 mg, 0.010 mmol), 4 Å molecular sieves (20 mg), alkene (**7**, 41.9 mg, 0.20 mmol), α-tetralol (**2q**, 29.6 mg, 0.20 mmol) and toluene (0.5 mL). The vial was sealed, taken outside the glovebox, heated to 100 °C and was allowed to stir for 20 h. The resulting mixture was cooled to room temperature, and then purified by column chromatography (silica gel, hexanes/dichloromethane/ triethylamine = 3/1/0.03) to provide a pale yellow oil which contained the desired product **3aC** and α-tetralone. This oil was treated with anhydrous methanol (2 mL) and sodium borohydride (7.4 mg, 0.2 mmol) under argon. The reaction was allowed to stir at 24 °C for 2 h. Then, methanol was removed under reduced pressure and the resulting residue was purified by

column chromatography (silica gel, hexanes/diethyl ether/triethylamine = 15/1/0.15) to provide the desired product **3aC** as a colorless oil 31.8 mg (75% yield). The enantiomeric excess of **3aC** was determined to be 22% by HPLC using Daicel CHIRALCEL OJ-H column.

7. X-Ray Crystal Structure Data

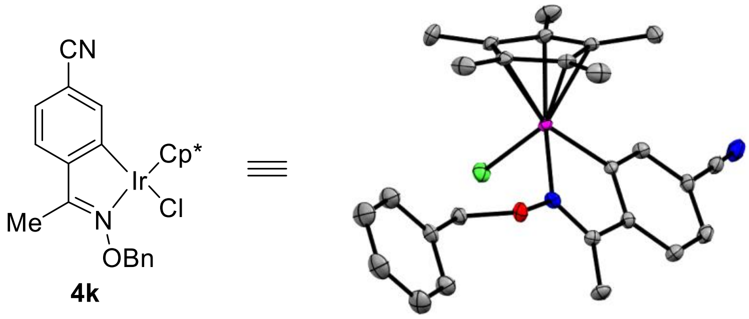

Orange crystals of **4k** were grown from a hexanes/DCM solution by slow volatilization. CCDC 2018968.

Identification code	J230	
Empirical formula	C26 H28 Cl Ir N2 O	
Formula weight	612.15	
Temperature	100(2) K	
Wavelength	0.71073 Å	
Crystal system	Triclinic	
Space group	P-1	
Unit cell dimensions	a = 8.4489(4) Å	α= 100.902(2)°.
	b = 12.2888(6) Å	β= 107.902(2)°.
	c = 12.5460(6) Å	γ = 103.991(2)°.
Volume	1152.78(10) Å^3	
Z	2	
Density (calculated)	1.764 Mg/m^3	
Absorption coefficient	5.928 mm^{-1}	
F(000)	600	
Crystal size	0.370 x 0.347 x 0.178 mm^3	
Theta range for data collection	3.350 to 28.276°.	
Index ranges	-11<=h<=11, -16<=k<=15, -15<=l<=16	
Reflections collected	20006	
Independent reflections	5706 [R(int) = 0.0347]	
Completeness to theta = 25.242°	99.8 %	
Absorption correction	Semi-empirical from equivalents	
Max. and min. transmission	0.7459 and 0.4927	
Refinement method	Full-matrix least-squares on F^2	
Data / restraints / parameters	5706 / 0 / 286	
Goodness-of-fit on F^2	1.231	
Final R indices [I>2sigma(I)]	R1 = 0.0184, wR2 = 0.0524	

R indices (all data)	R1 = 0.0188, wR2 = 0.0526
Extinction coefficient	n/a
Largest diff. peak and hole	1.977 and -0.893 e.Å^{-3}

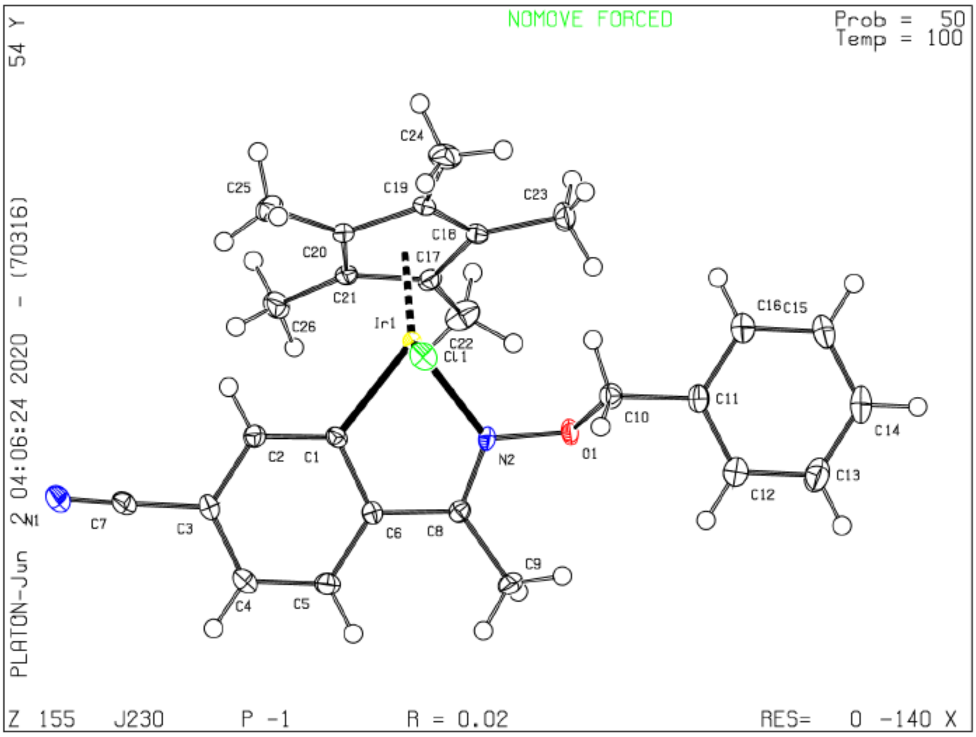

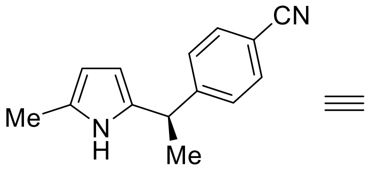

3ad

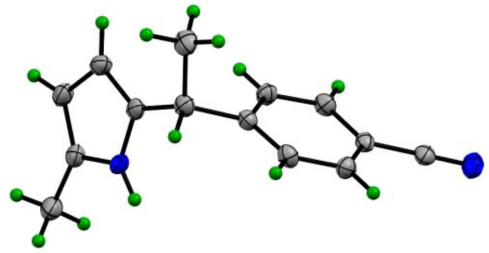

Colorless crystals of **3ad** were grown from a pentane/DCM solution by freezing in a -20 ºC refrigerator using 24 mg of **3ad** with 87% ee. CCDC 2018969.

Identification code	J027	
Empirical formula	C14 H14 N2	
Formula weight	210.27	
Temperature	100(2) K	
Wavelength	1.54178 Å	
Crystal system	Orthorhombic	
Space group	$P2_12_12_1$	
Unit cell dimensions	a = 10.0727(5) Å	α= 90°.
	b = 10.7413(5) Å	β= 90°.
	c = 11.1477(6) Å	γ = 90°.
Volume	1206.11(10) $Å^3$	
Z	4	
Density (calculated)	1.158 Mg/m^3	
Absorption coefficient	0.535 mm^{-1}	
F(000)	448	
Crystal size	0.362 x 0.289 x 0.217 mm^3	
Theta range for data collection	5.720 to 68.311°.	
Index ranges	-12<=h<=12, -12<=k<=12, -13<=l<=13	
Reflections collected	24730	
Independent reflections	2204 [R(int) = 0.0268]	
Completeness to theta = 67.679°	99.8 %	
Absorption correction	Semi-empirical from equivalents	
Max. and min. transmission	0.7531 and 0.6842	
Refinement method	Full-matrix least-squares on F^2	
Data / restraints / parameters	2204 / 0 / 151	
Goodness-of-fit on F^2	1.093	
Final R indices [I>2sigma(I)]	R1 = 0.0274, wR2 = 0.0693	
R indices (all data)	R1 = 0.0274, wR2 = 0.0693	
Absolute structure parameter	0.02(5)	
Extinction coefficient	n/a	
Largest diff. peak and hole	0.123 and -0.192 e.$Å^{-3}$	

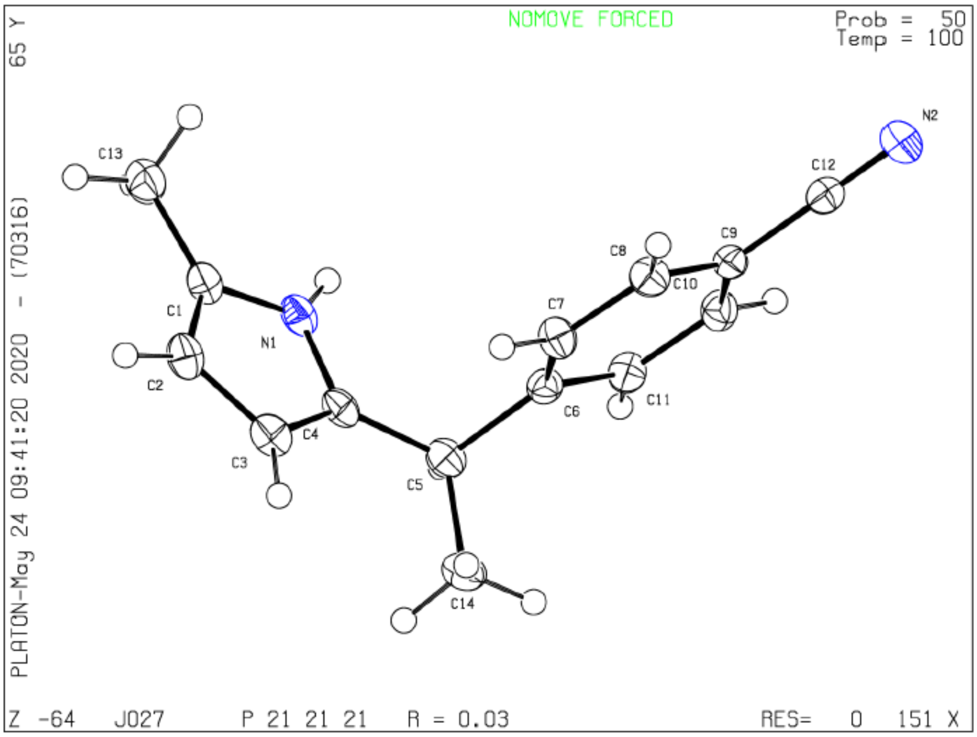
NOMOVE FORCED
Prob = 50
Temp = 100
65 Y
PLATON-May 24 09:41:20 2020 - (70316)
N2
C12
C13
C9
C8
C10
C1
N1
C7
C2
C6
C11
C4
C3
C5
C14
Z -64 J027 P 21 21 21 R = 0.03 RES= 0 151 X

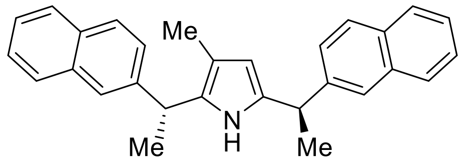

3kp

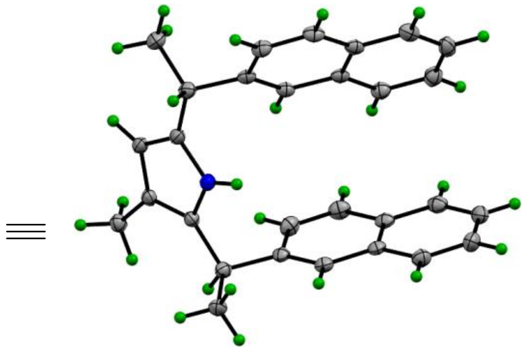

Yellow crystals of **3kp** were grown from a pentane/DCM solution by freezing in a -20 °C refrigerator using 44 mg of **3kp** with 95% ee. CCDC 2018974.

Identification code	K229	
Empirical formula	C29 H27 N	
Formula weight	389.51	
Temperature	100(2) K	
Wavelength	1.54178 Å	
Crystal system	Orthorhombic	
Space group	$P2_12_12_1$	
Unit cell dimensions	a = 5.7475(2) Å	α= 90°.
	b = 16.5692(6) Å	β= 90°.
	c = 22.1692(8) Å	γ = 90°.
Volume	2111.21(13) Å^3	
Z	4	
Density (calculated)	1.225 Mg/m^3	
Absorption coefficient	0.530 mm^{-1}	
F(000)	832	
Crystal size	0.092 x 0.063 x 0.059 mm^3	
Theta range for data collection	3.330 to 70.194°.	
Index ranges	-7<=h<=7, -20<=k<=18, -27<=l<=27	
Reflections collected	37244	
Independent reflections	3994 [R(int) = 0.0285]	
Completeness to theta = 67.679°	100.0 %	
Absorption correction	Semi-empirical from equivalents	
Max. and min. transmission	0.7533 and 0.7105	
Refinement method	Full-matrix least-squares on F^2	
Data / restraints / parameters	3994 / 0 / 278	
Goodness-of-fit on F^2	1.034	
Final R indices [I>2sigma(I)]	R1 = 0.0270, wR2 = 0.0709	
R indices (all data)	R1 = 0.0276, wR2 = 0.0716	

Absolute structure parameter	0.08(13)
Extinction coefficient	n/a
Largest diff. peak and hole	0.132 and -0.188 e.Å^{-3}

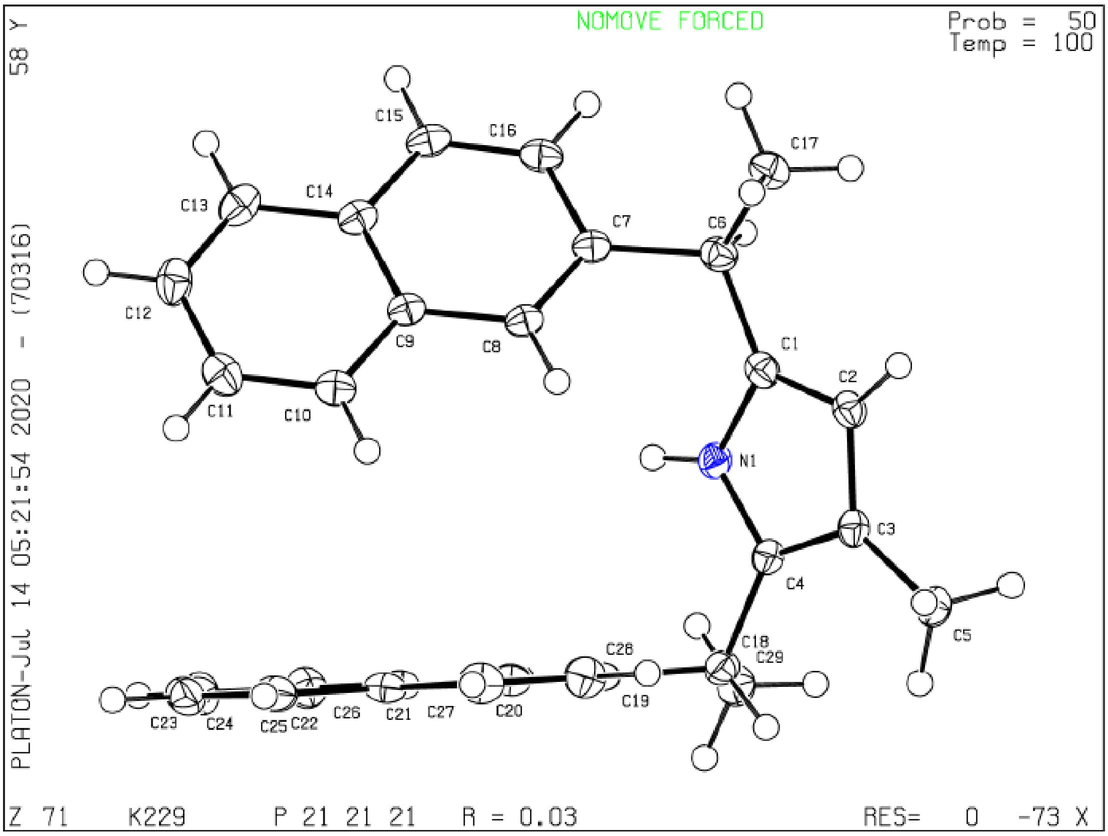

8. NMR Spectra

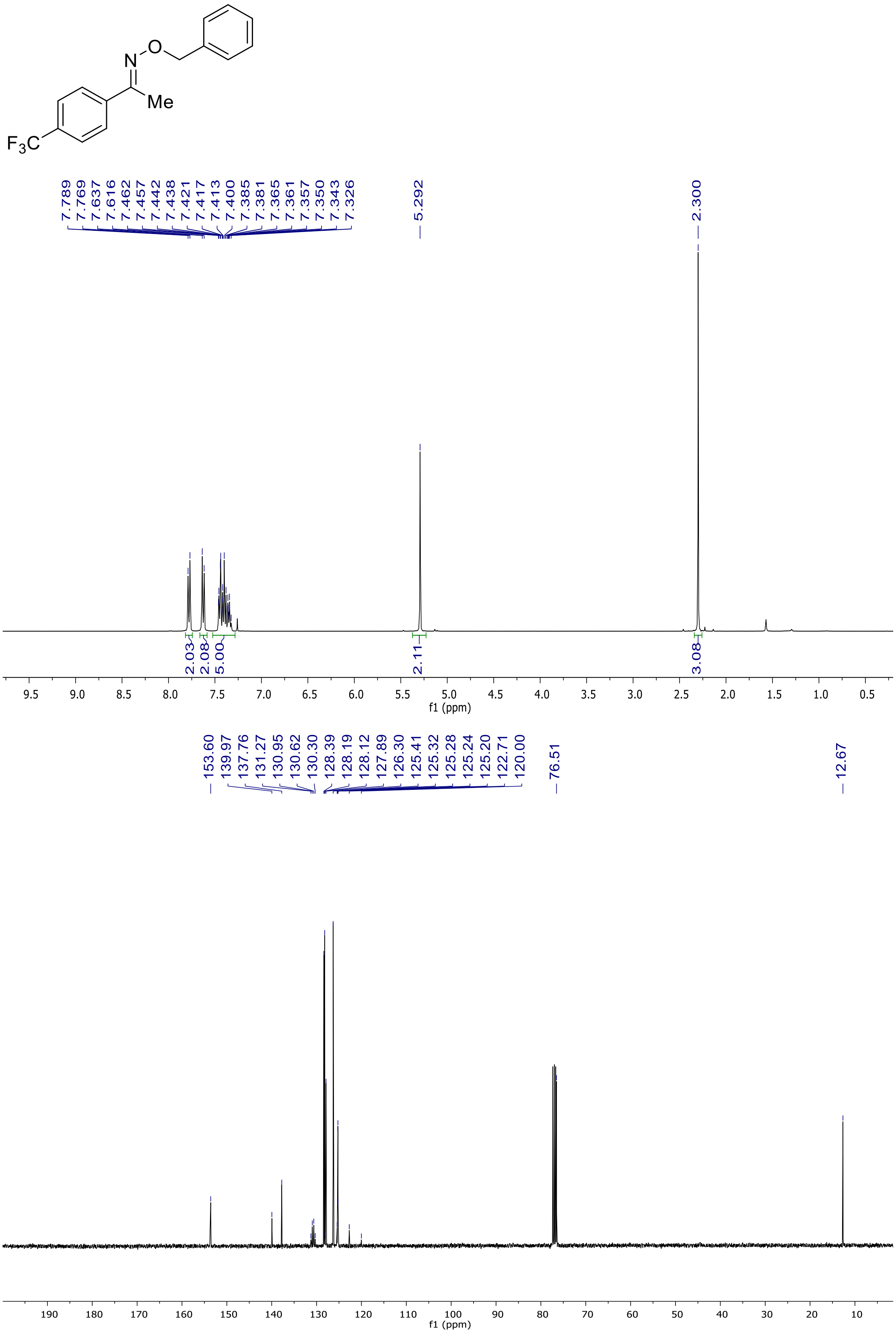

Supplementary Figure 40. ^{1}H NMR and ^{13}C NMR spectra of (*E*)-1-(4-(Trifluoromethyl)phenyl) ethan-1-one *O*-benzyl oxime.

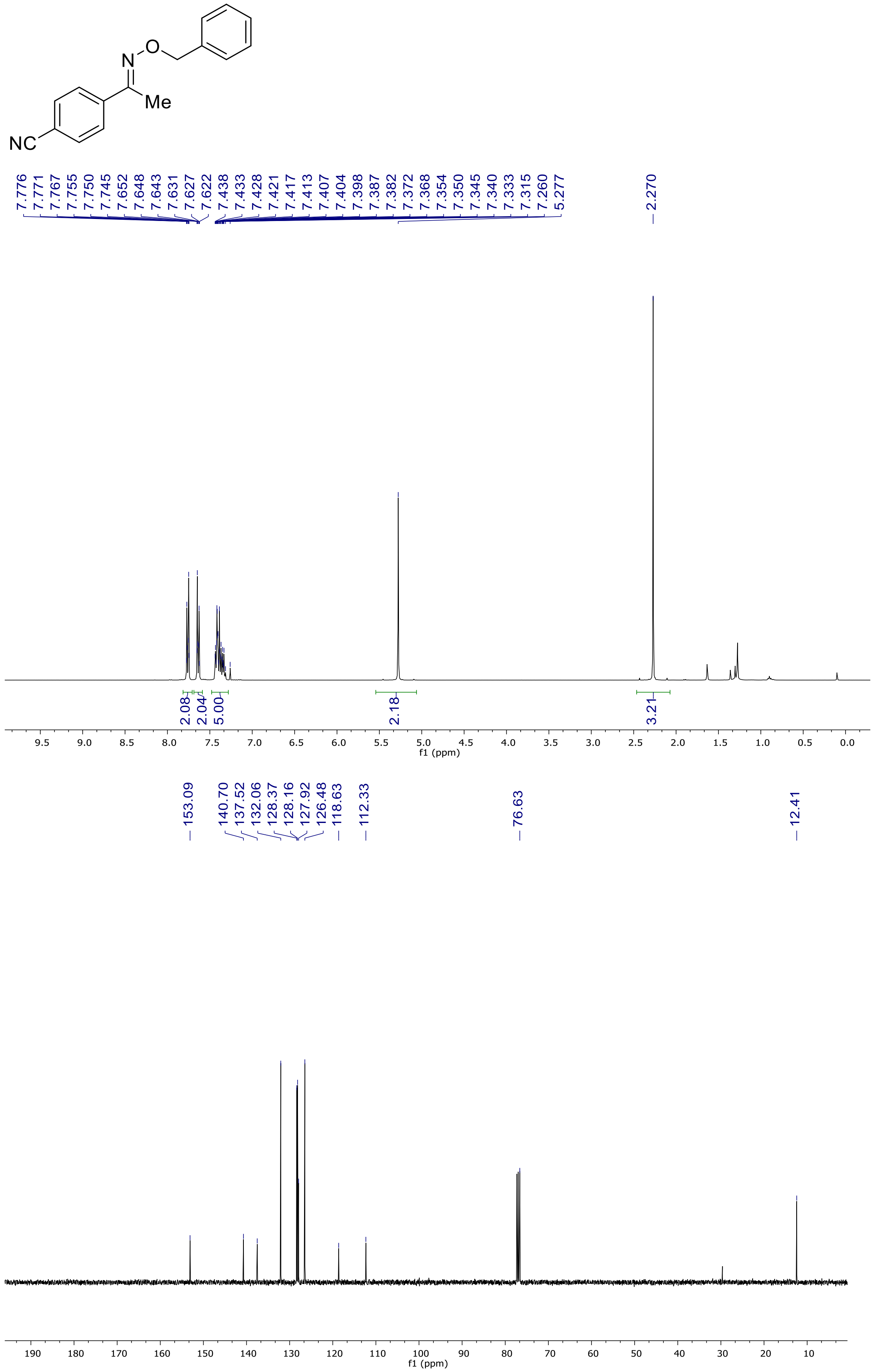

Supplementary Figure 41. ^{1}H NMR and ^{13}C NMR spectra of (*E*)-4-(1-((Benzyloxy)imino)ethyl) benzonitrile

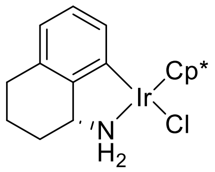

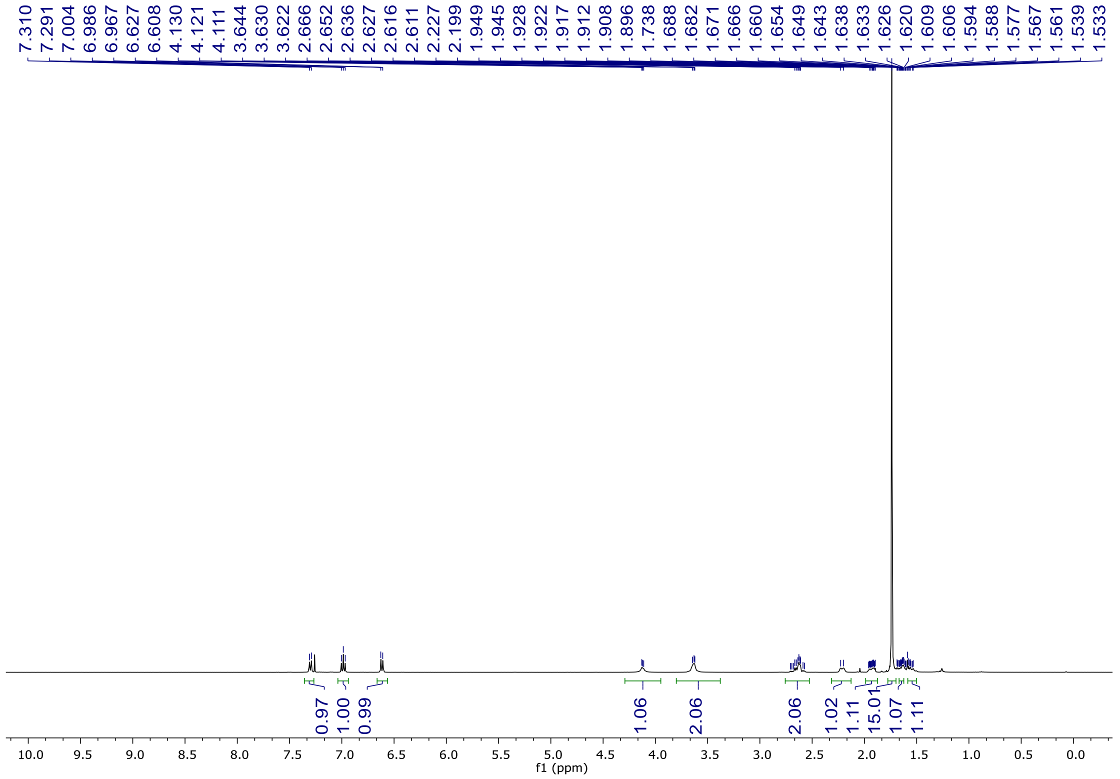

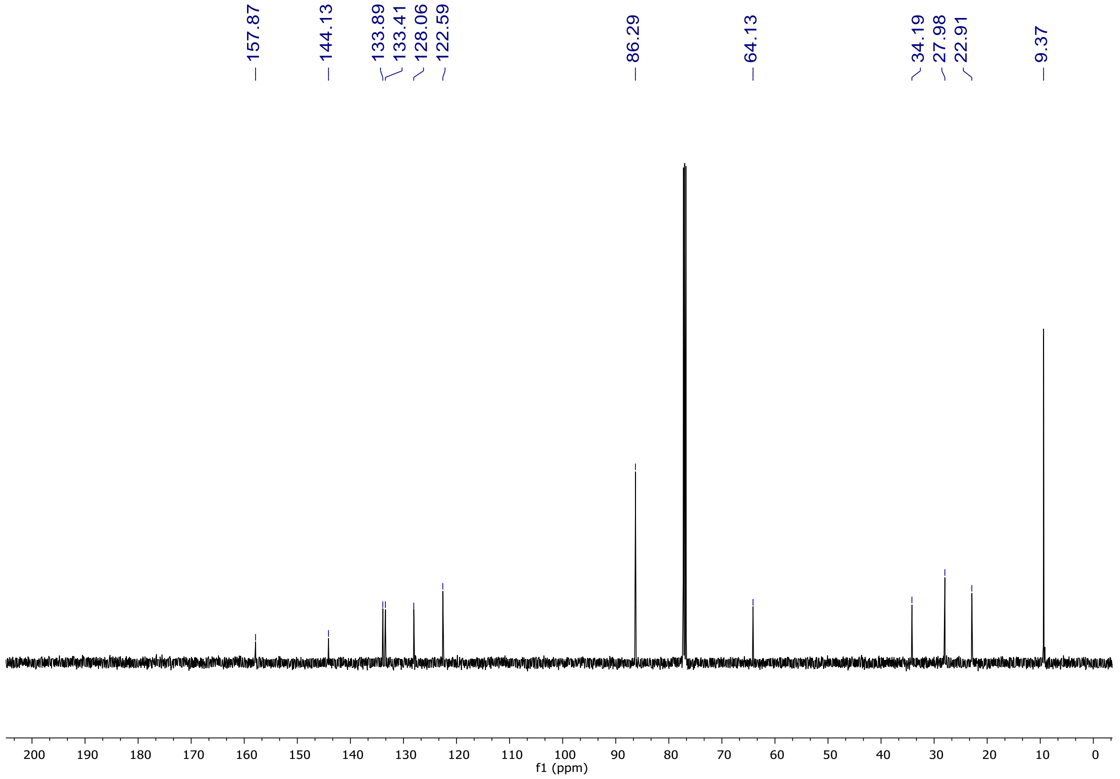

Supplementary Figure 42. ^{1}H NMR and ^{13}C NMR spectra of **4c**.

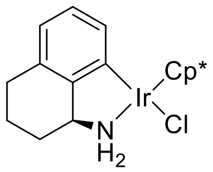

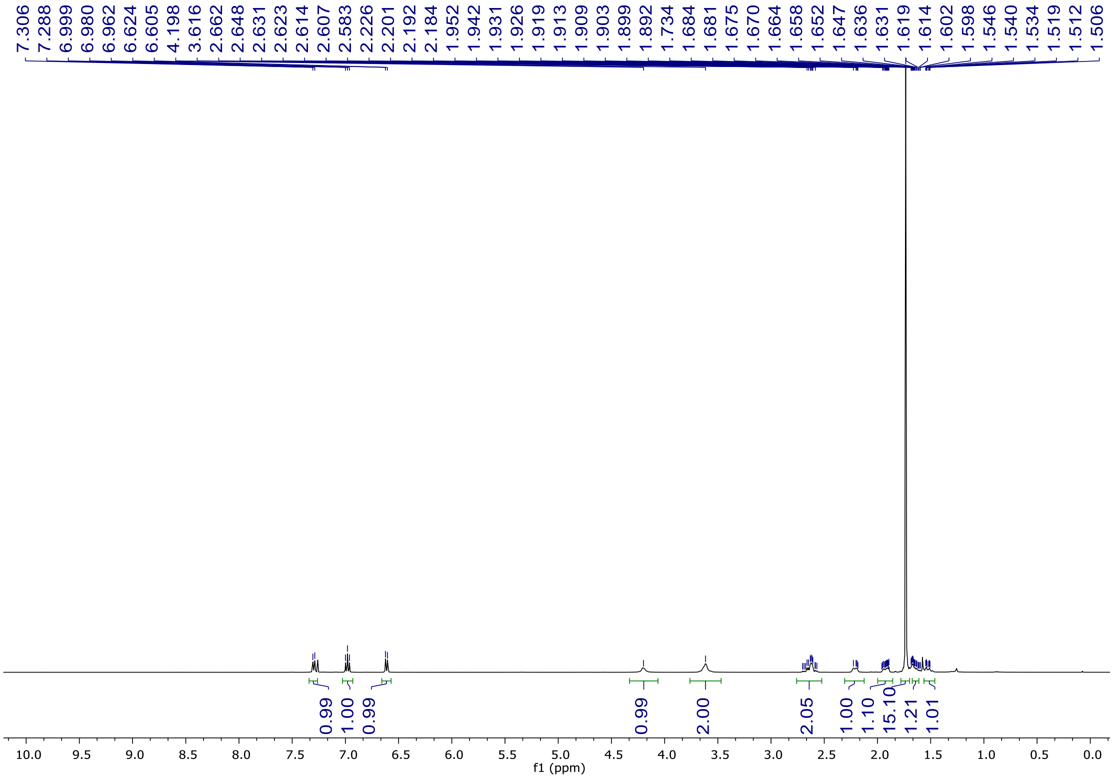

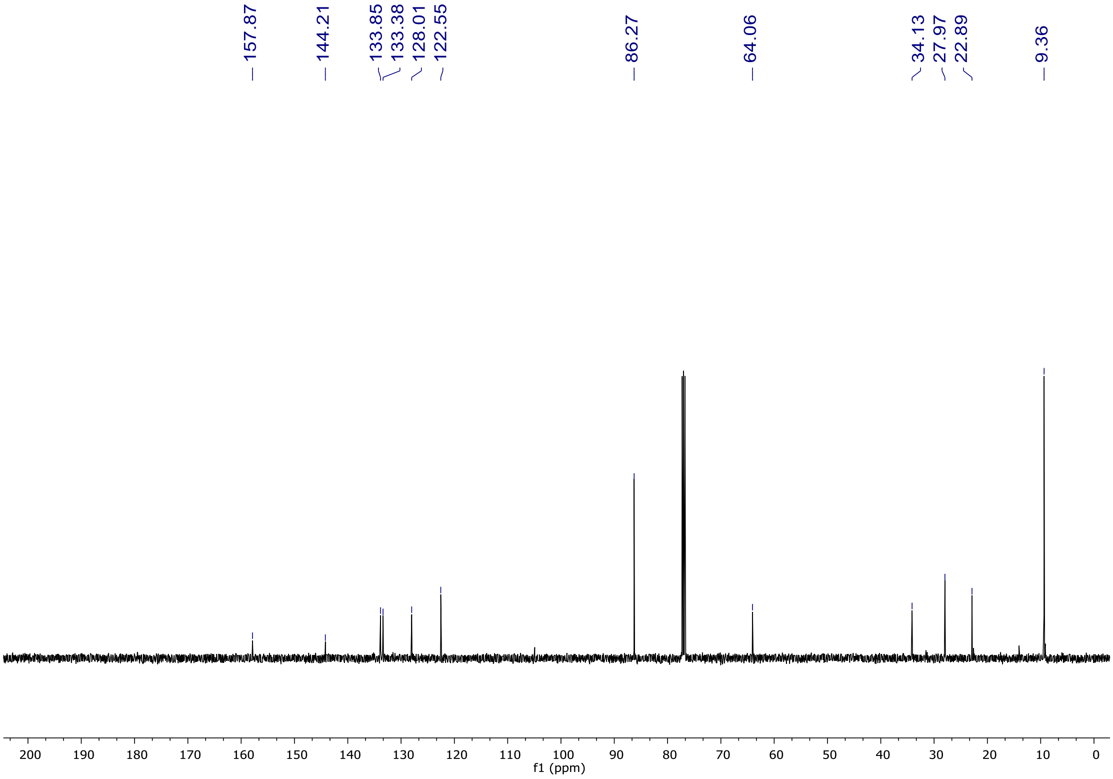

Supplementary Figure 43. ^{1}H NMR and ^{13}C NMR spectra of **4c′**.

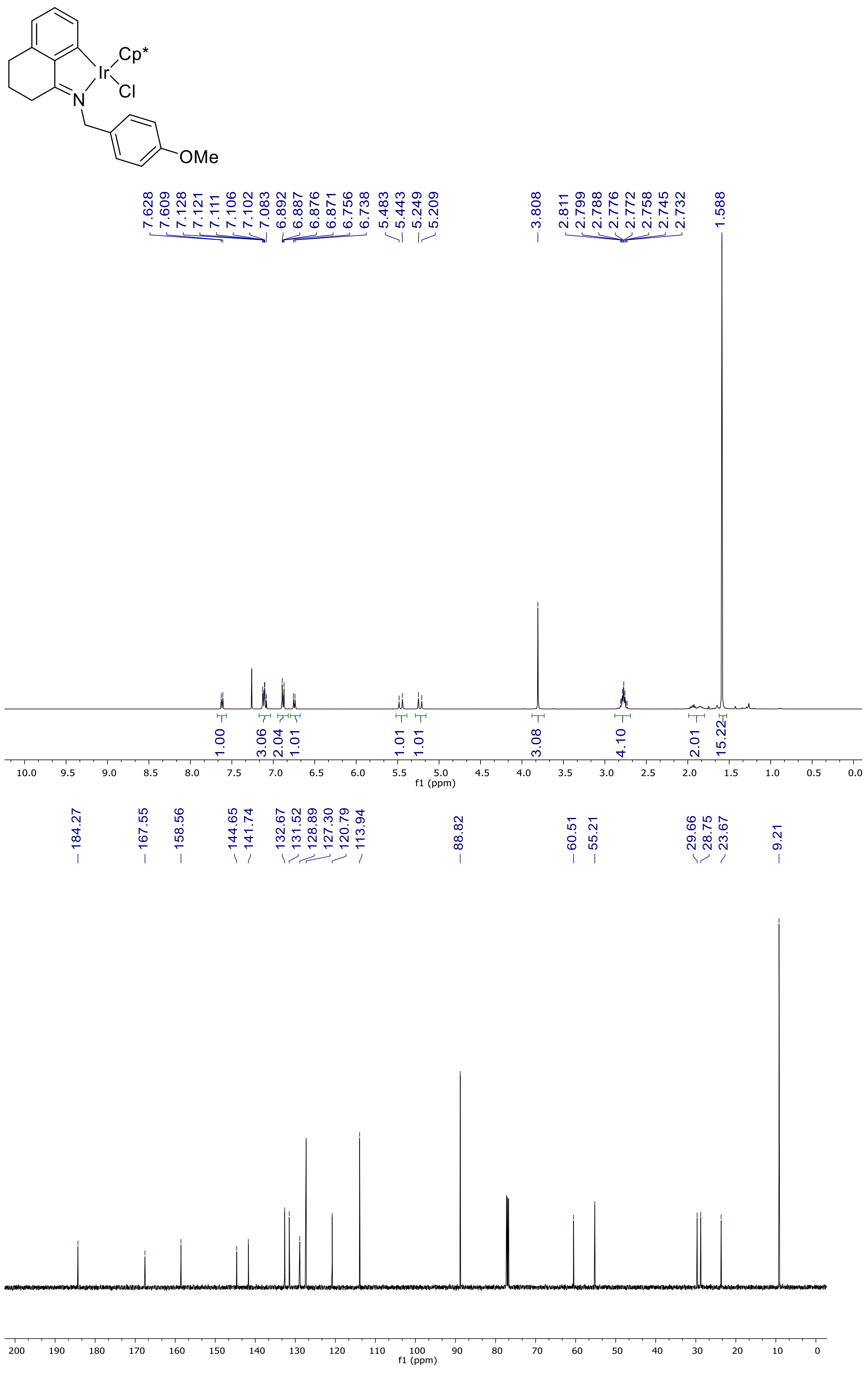

Supplementary Figure 44. ^{1}H NMR and ^{13}C NMR spectra of **4d**.

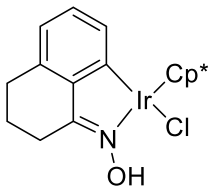

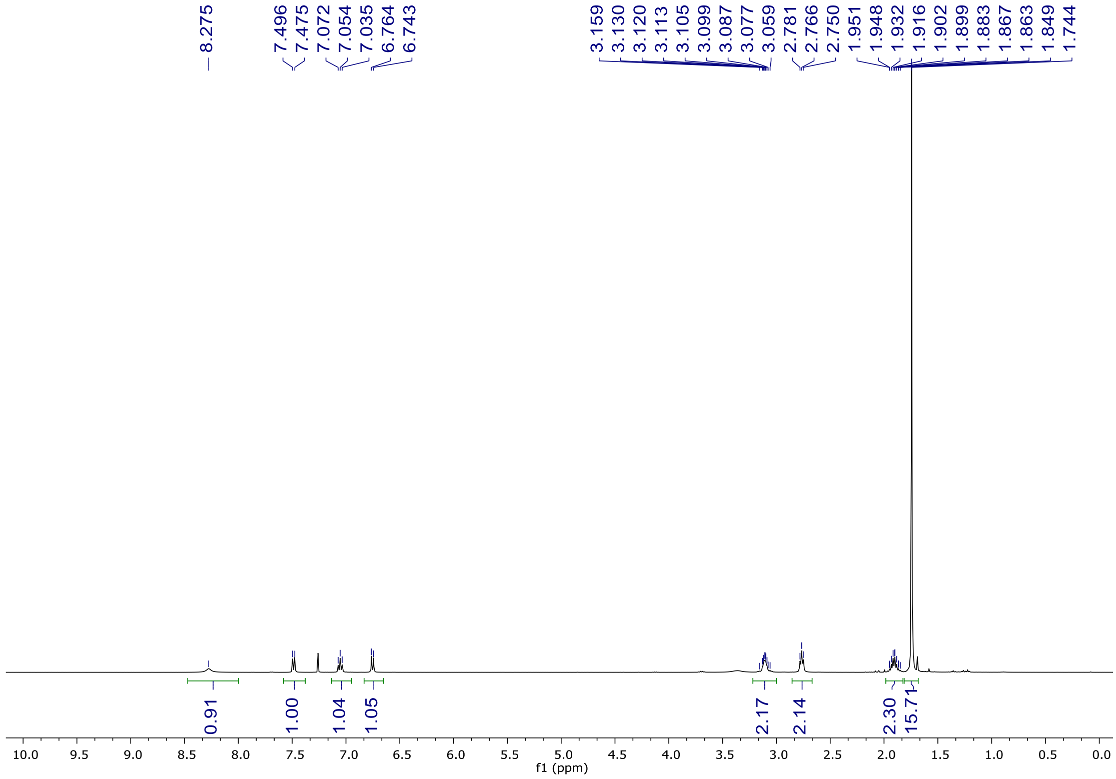

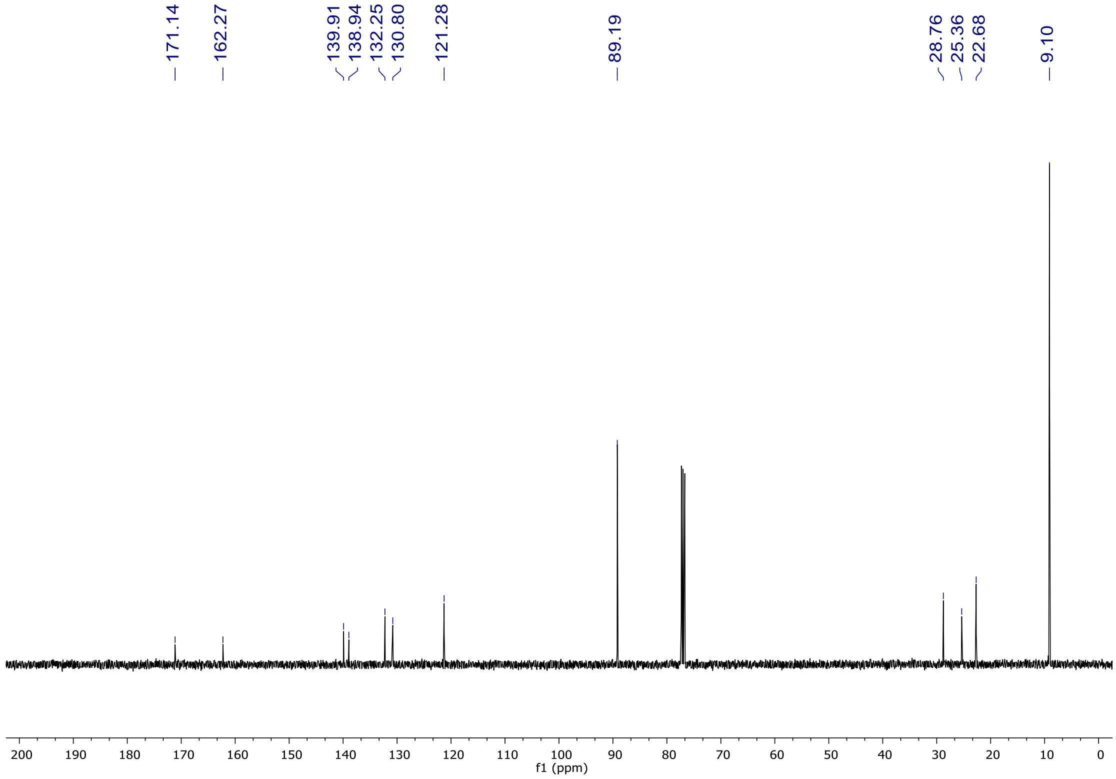

Supplementary Figure 45. ^{1}H NMR and ^{13}C NMR spectra of **4e**.

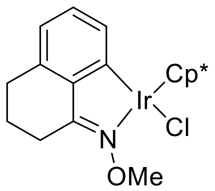

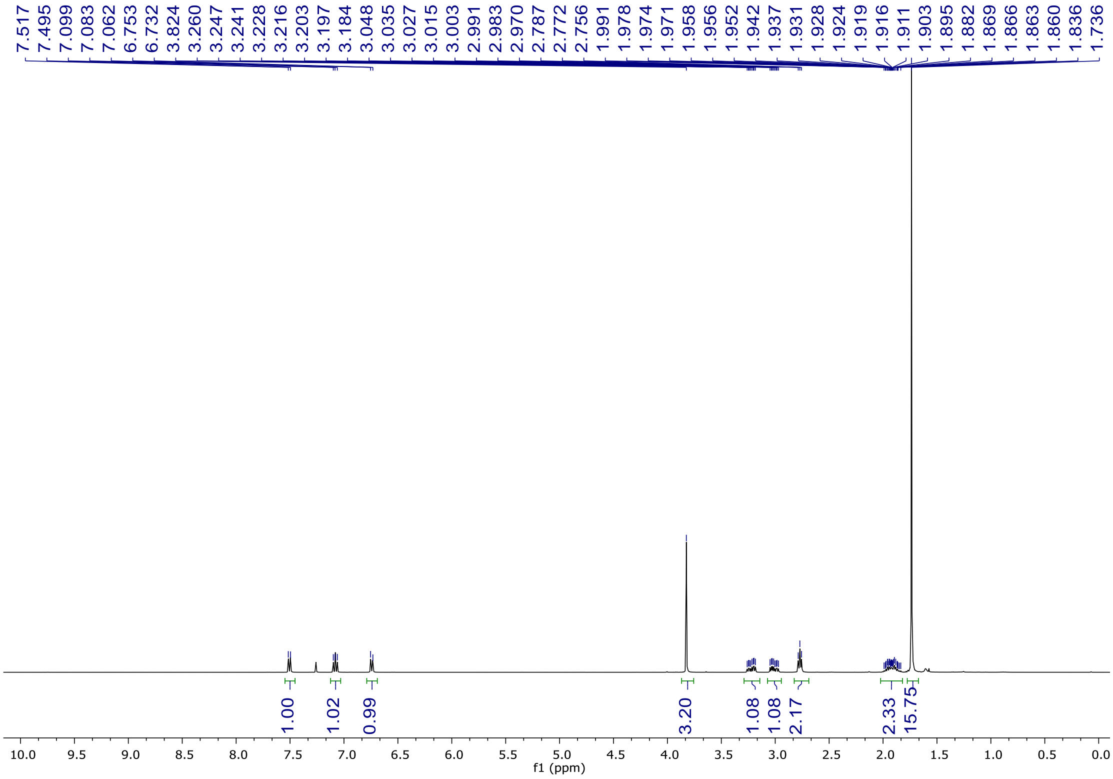

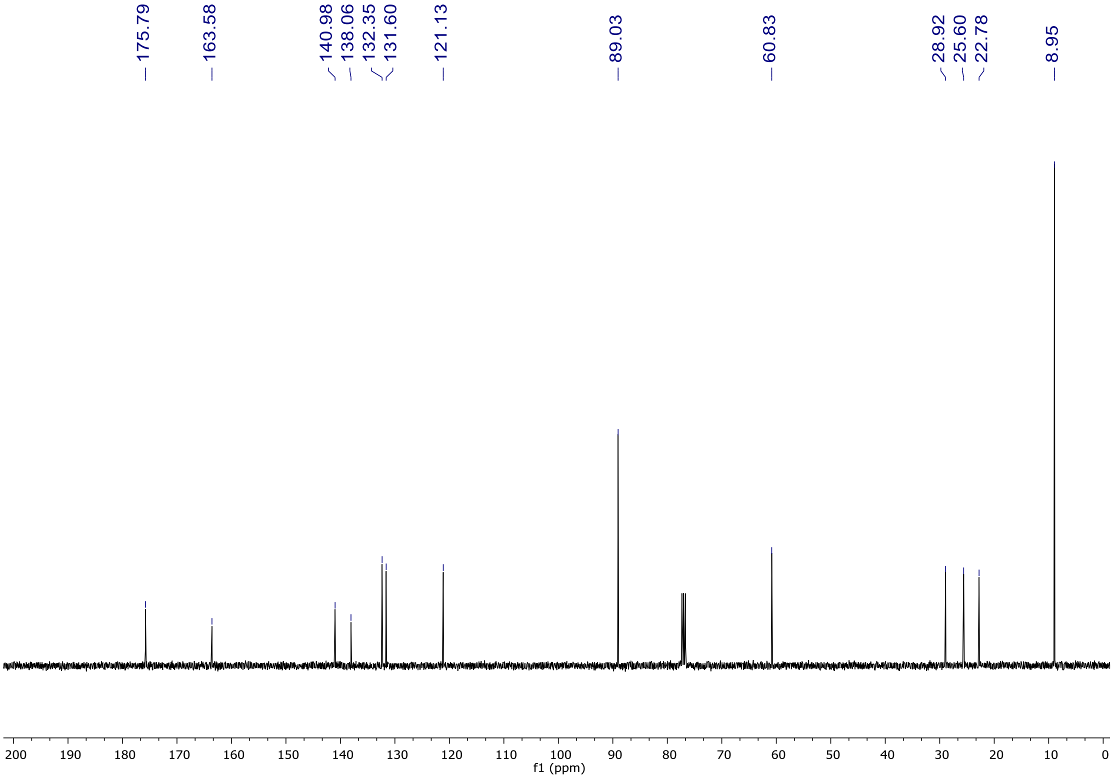

Supplementary Figure 46. ^{1}H NMR and ^{13}C NMR spectra of **4f**.

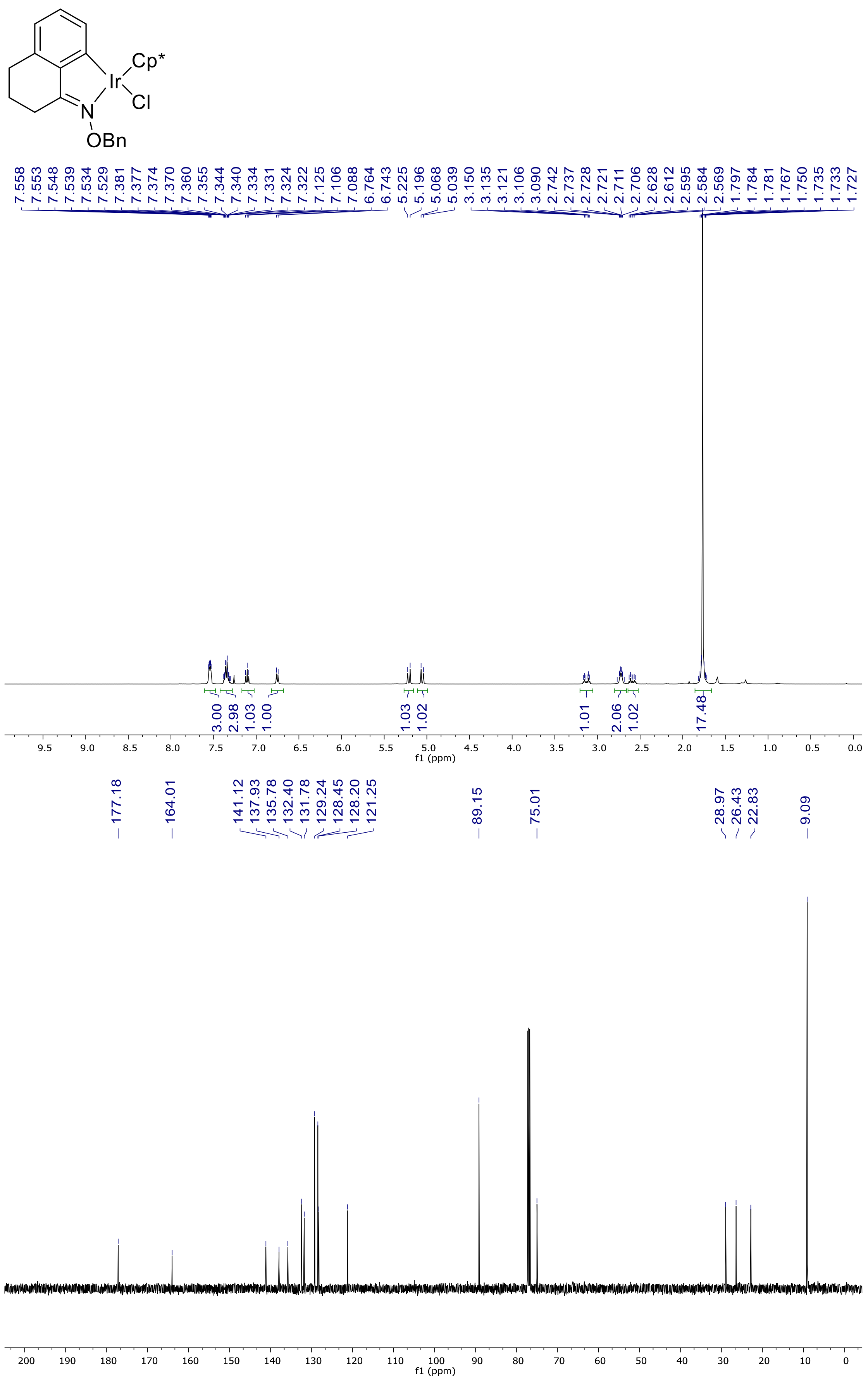

Supplementary Figure 47. ^{1}H NMR and ^{13}C NMR spectra of **4g**.

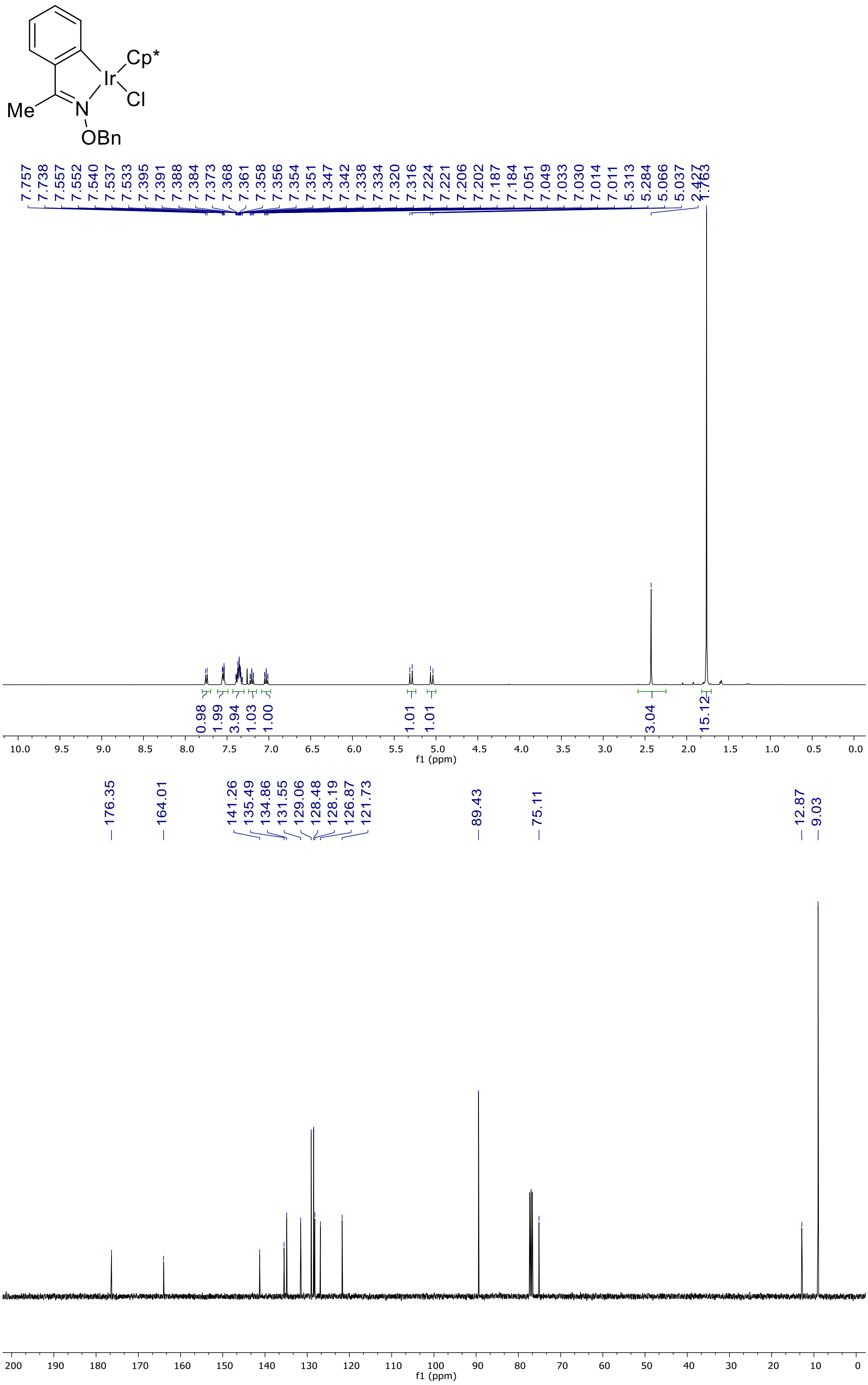

Supplementary Figure 48. ^{1}H NMR and ^{13}C NMR spectra of **4h**.

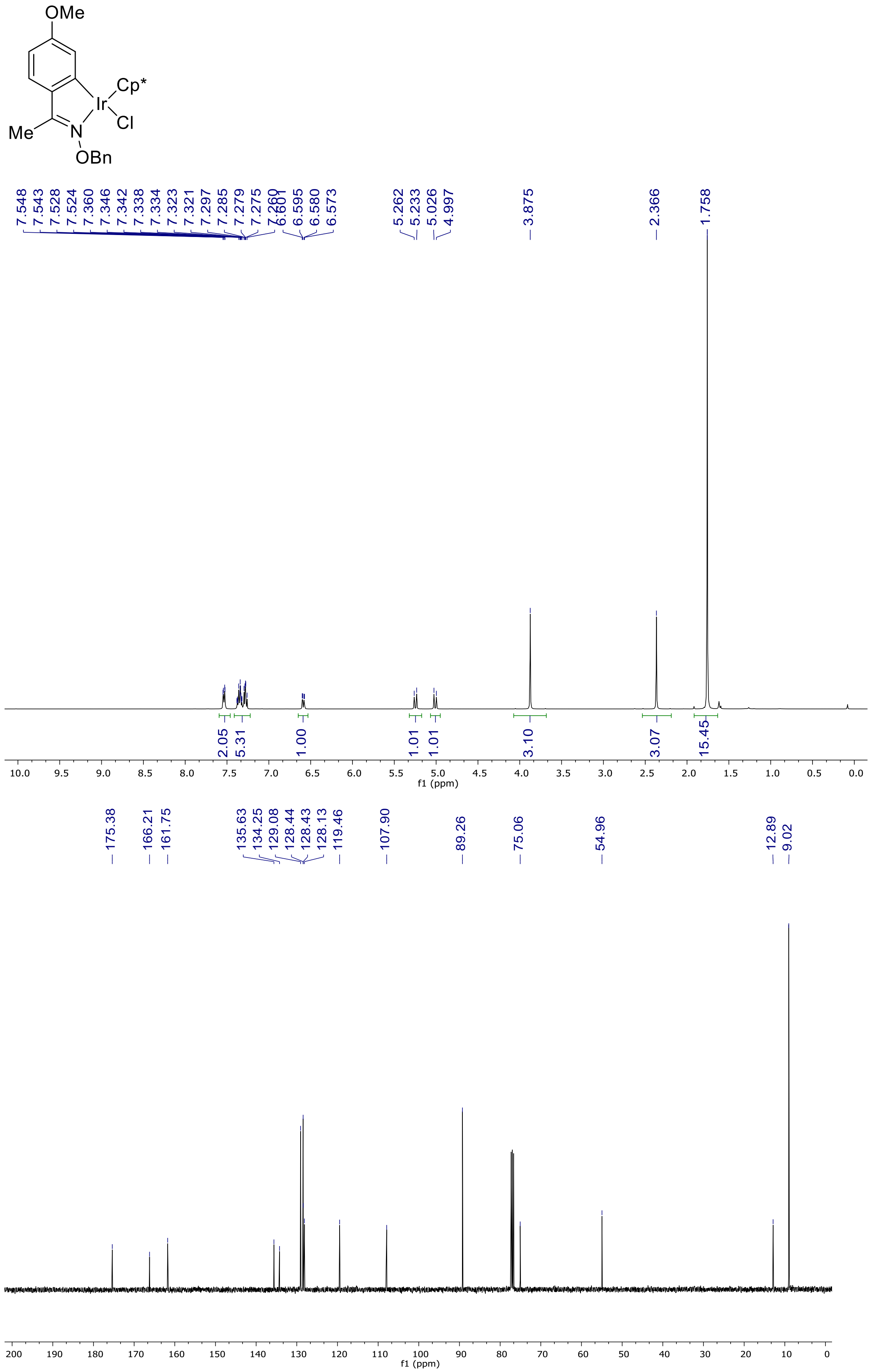

Supplementary Figure 49. ^{1}H NMR and ^{13}C NMR spectra of **4i**.

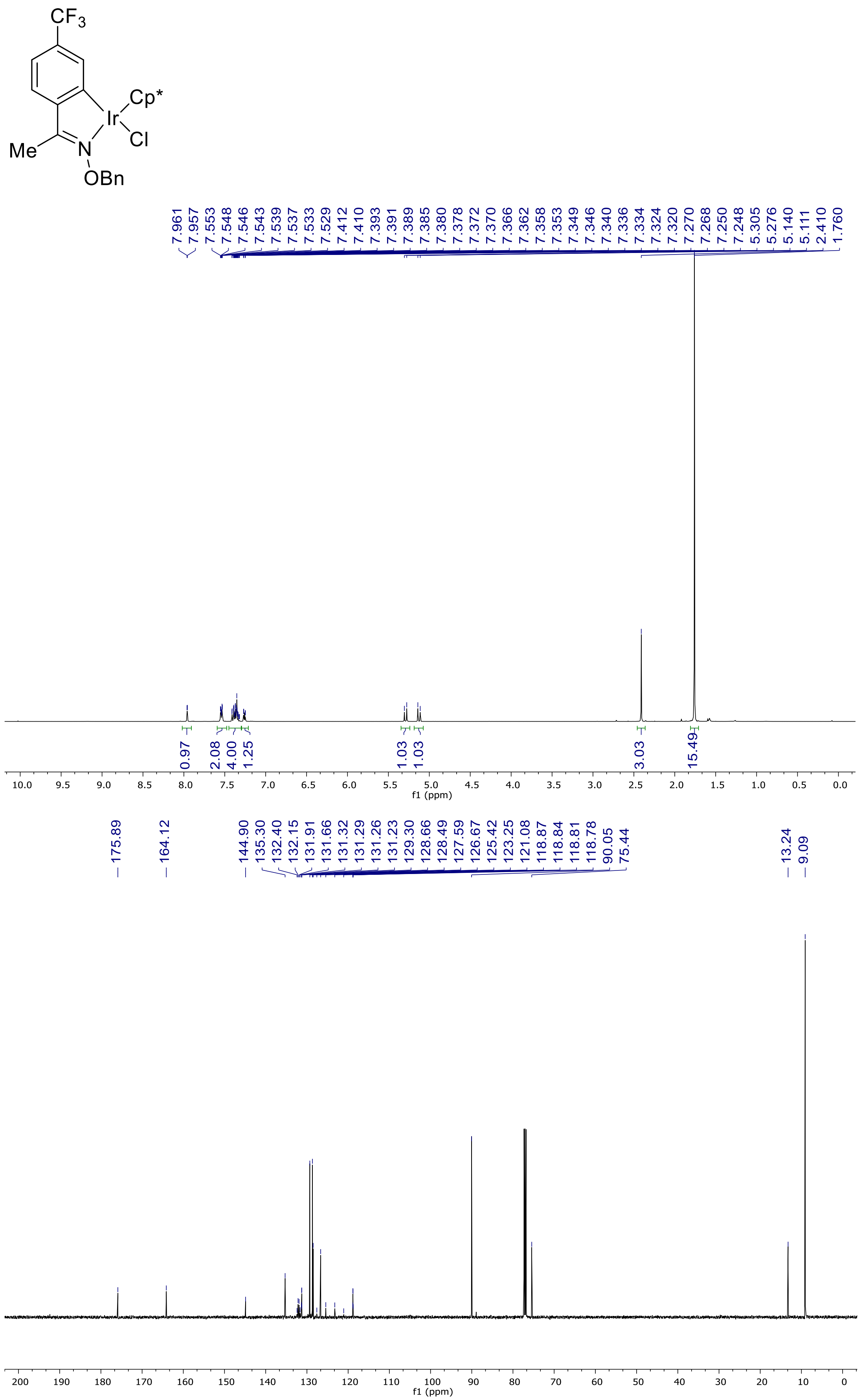

Supplementary Figure 50. ^{1}H NMR and ^{13}C NMR spectra of **4j**.

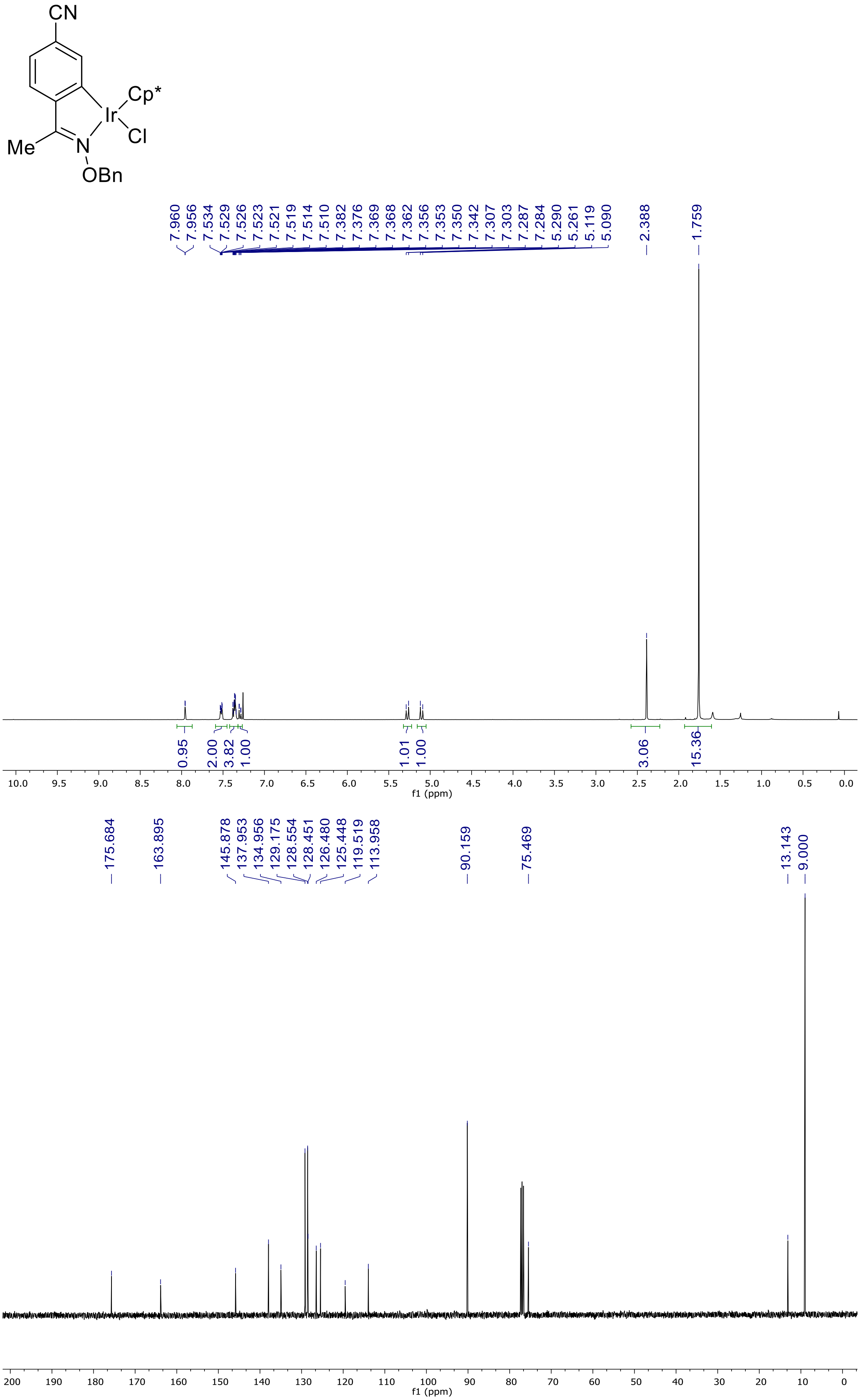

Supplementary Figure 51. ^{1}H NMR and ^{13}C NMR spectra of **4k**.

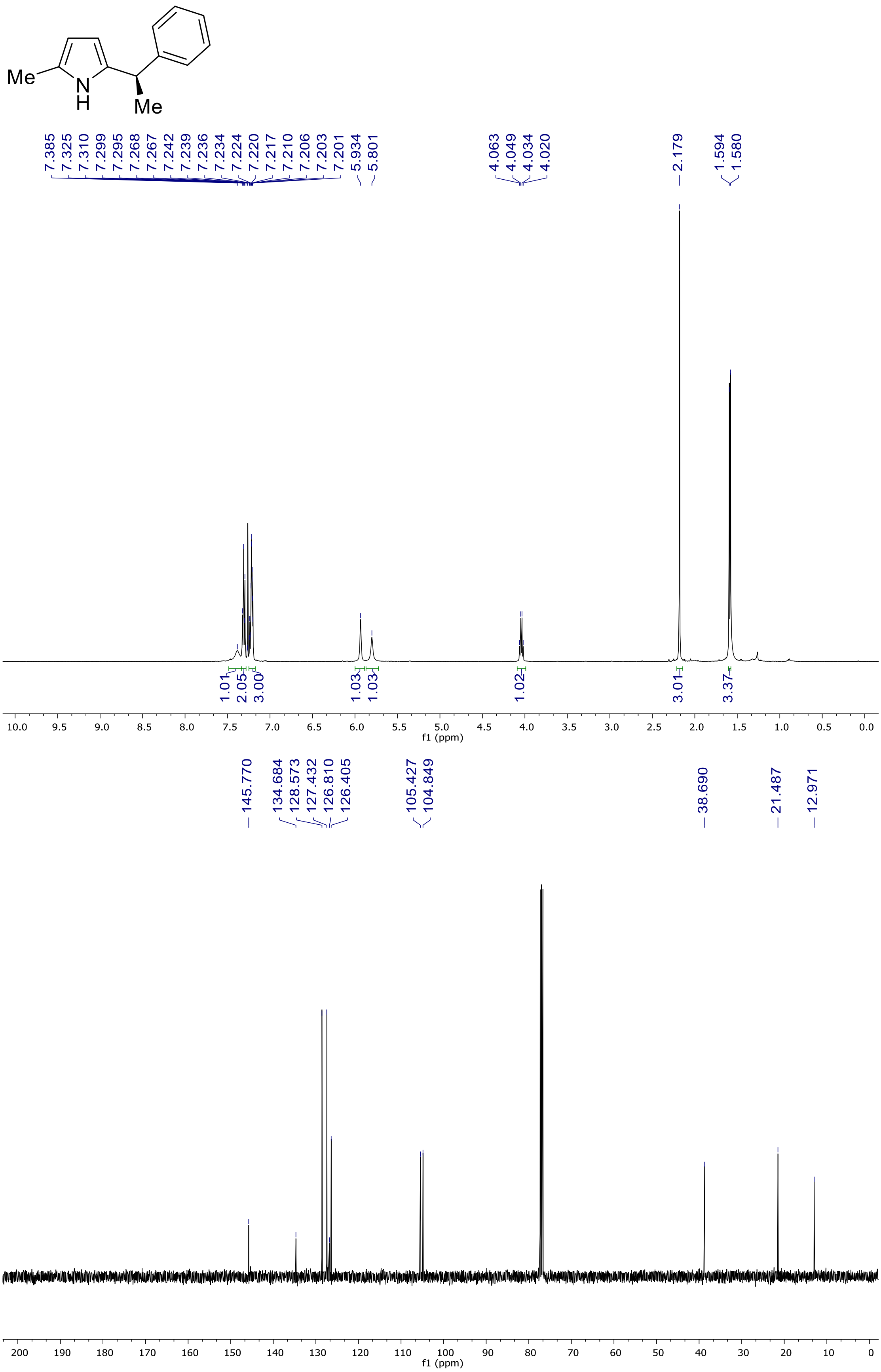

Supplementary Figure 52. ^{1}H NMR and ^{13}C NMR spectra of **3aa**.

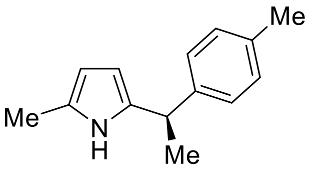

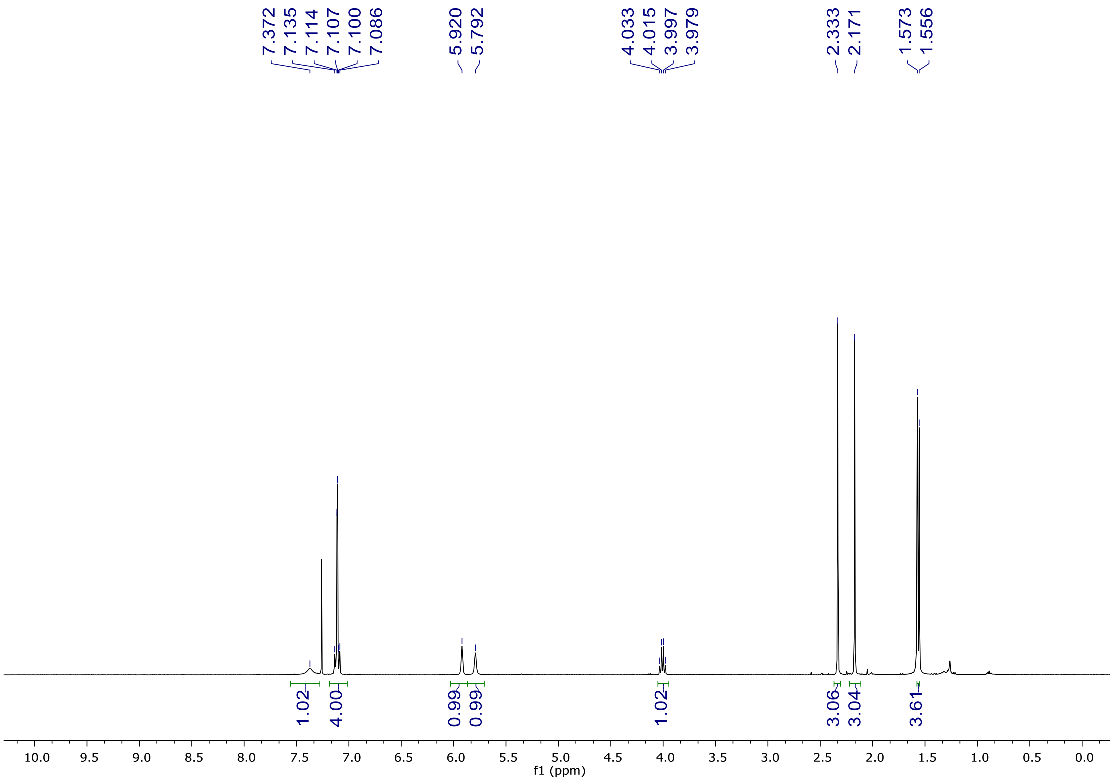

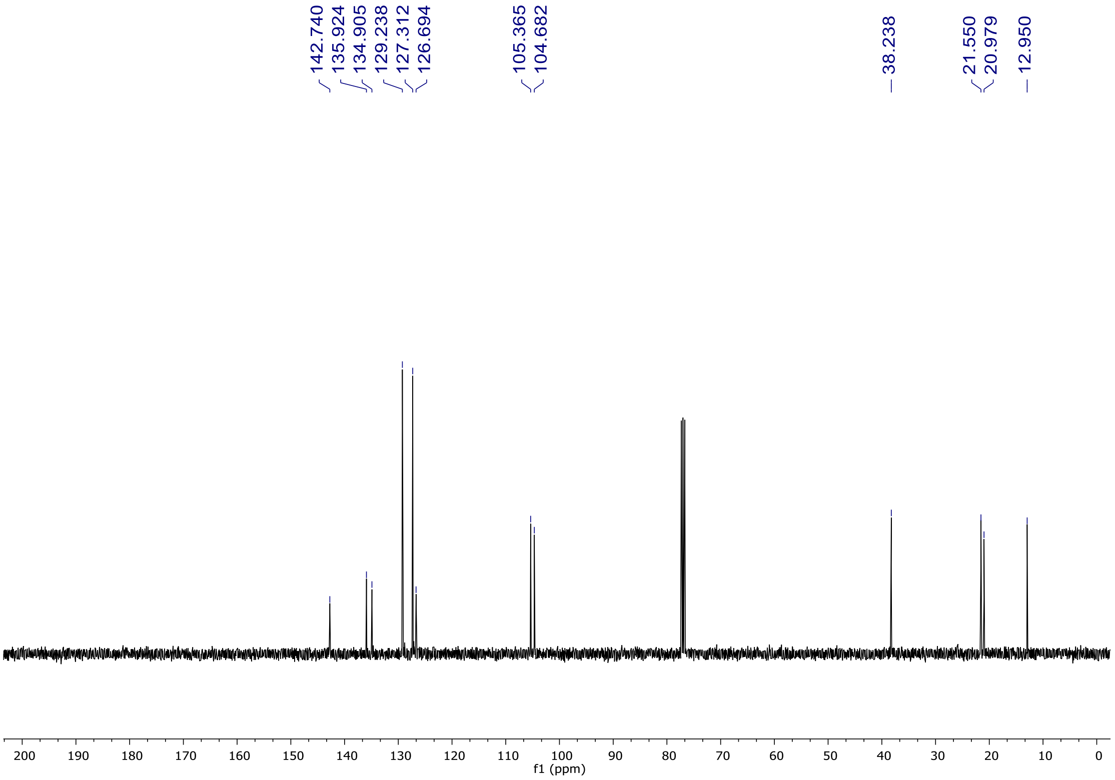

Supplementary Figure 53. ^{1}H NMR and ^{13}C NMR spectra of **3ab**.

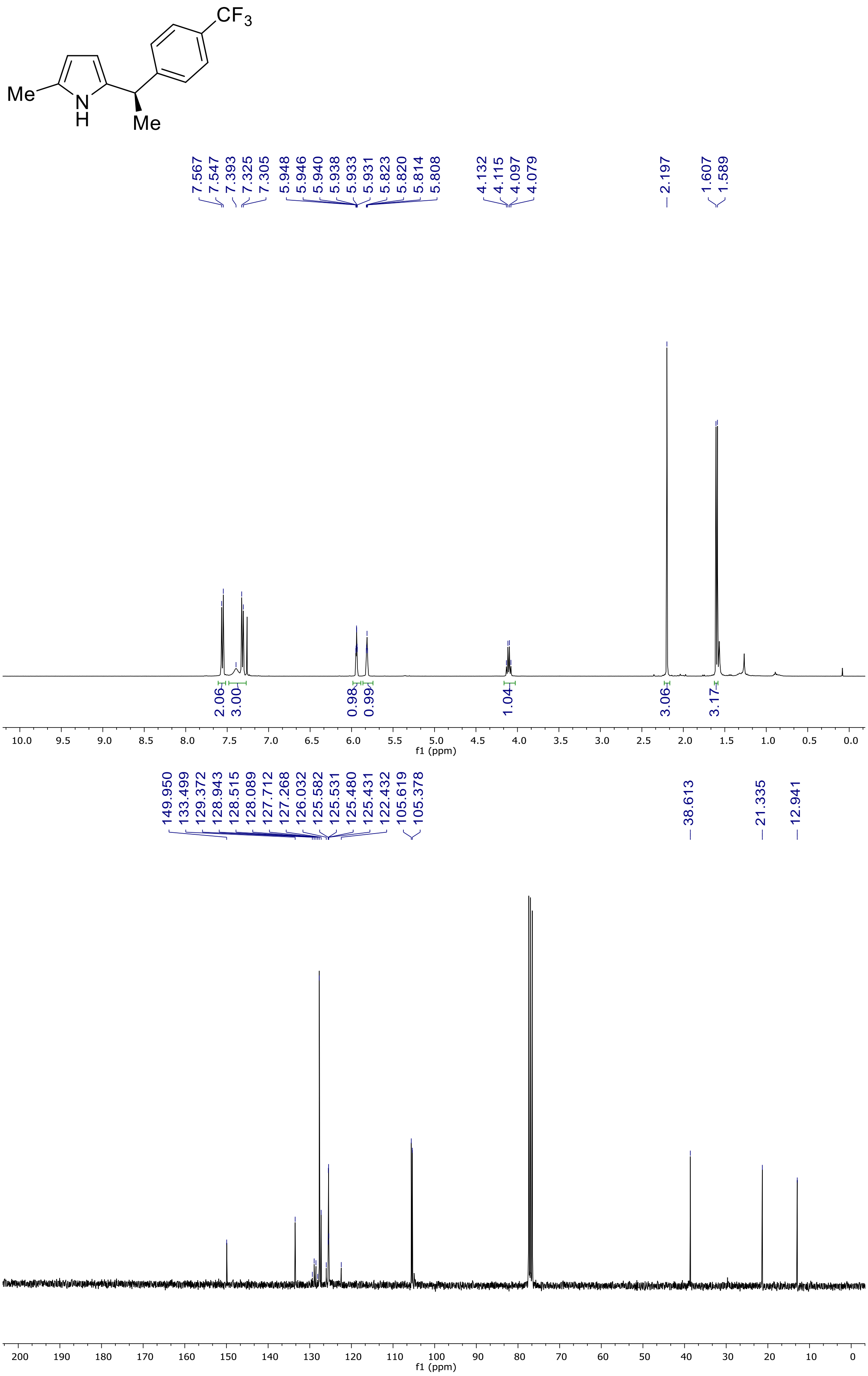

Supplementary Figure 54. ^{1}H NMR and ^{13}C NMR spectra of **3ac**.

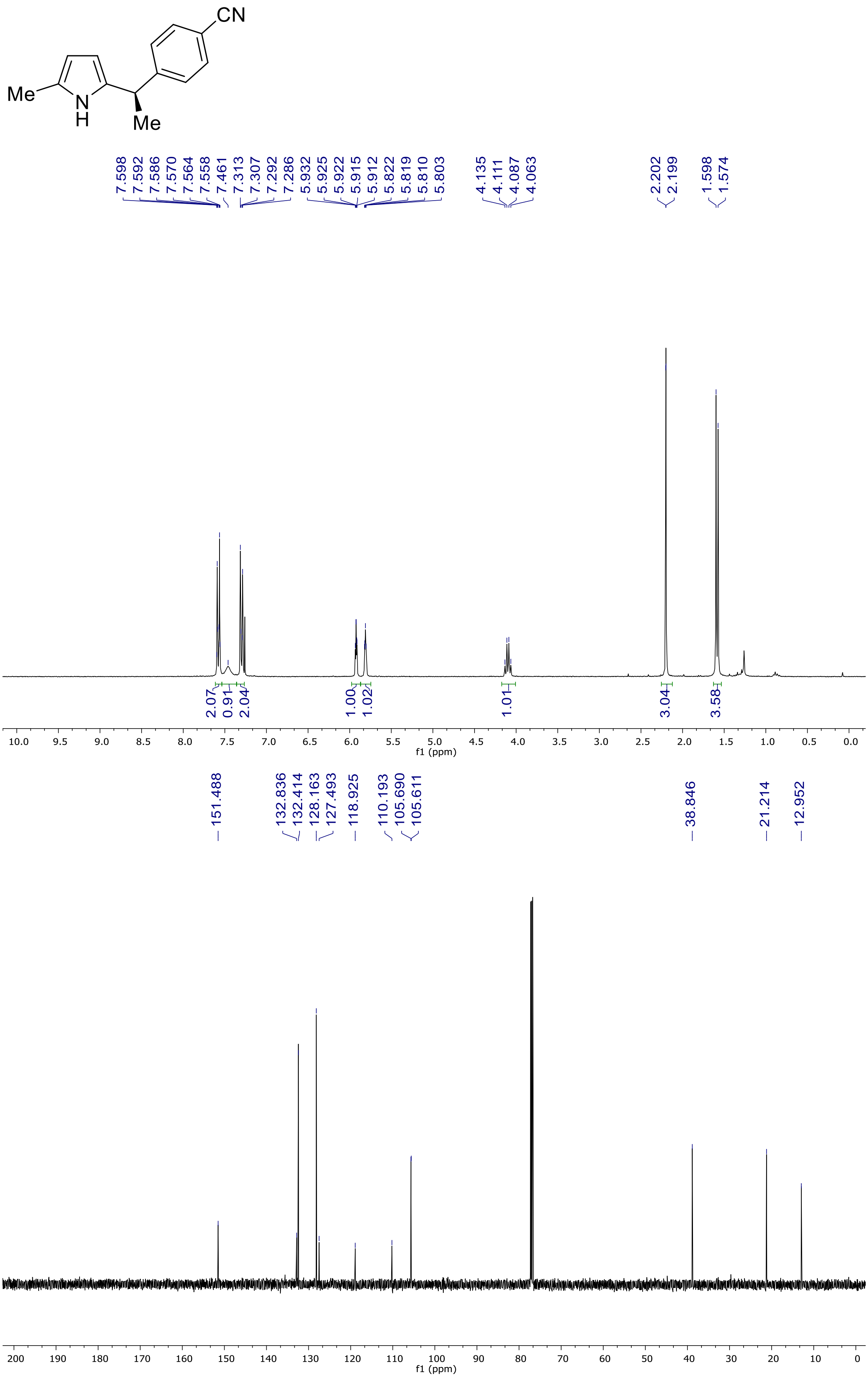

Supplementary Figure 55. ^{1}H NMR and ^{13}C NMR spectra of **3ad**.

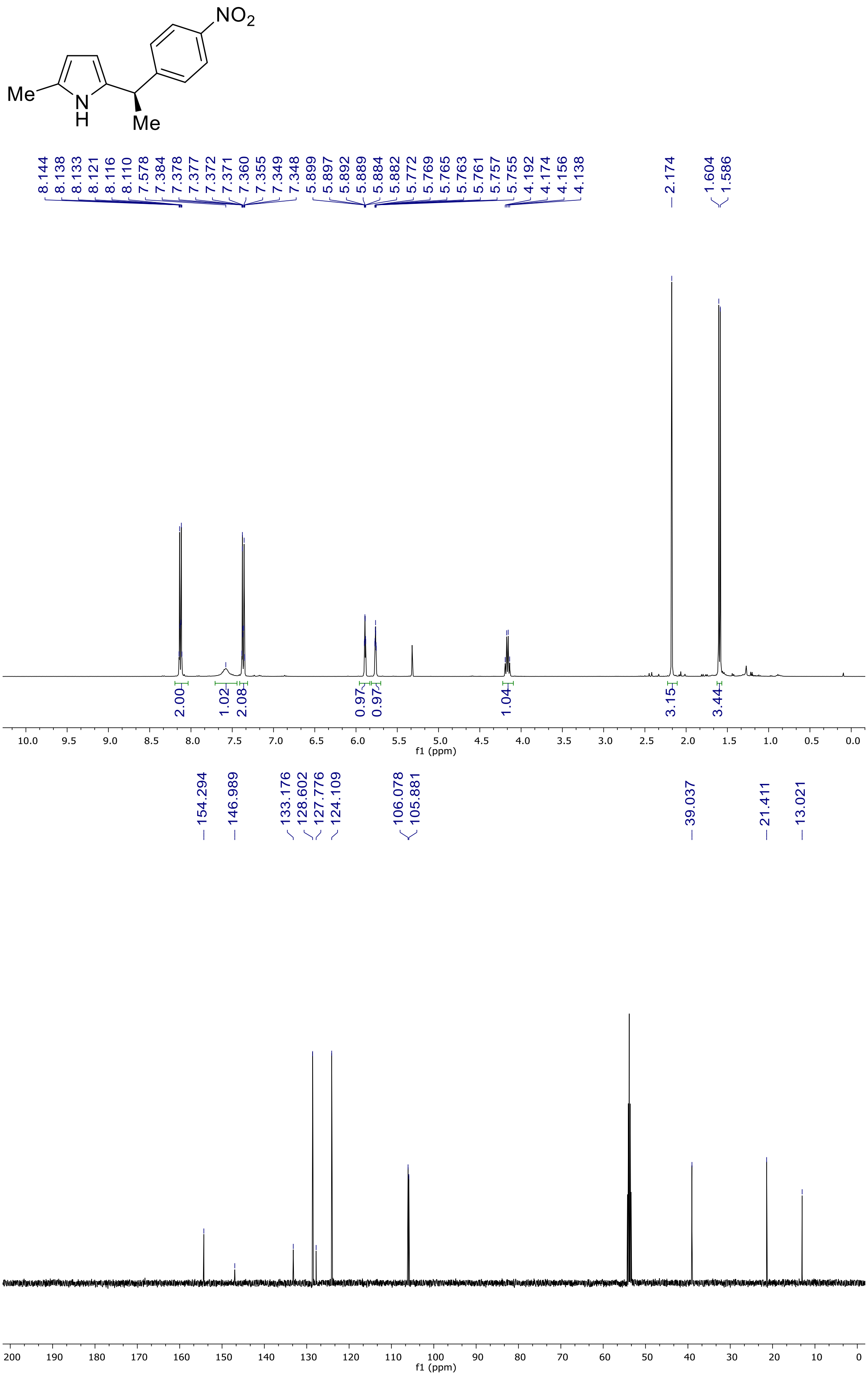

Supplementary Figure 56. ^{1}H NMR and ^{13}C NMR spectra of **3ae**.

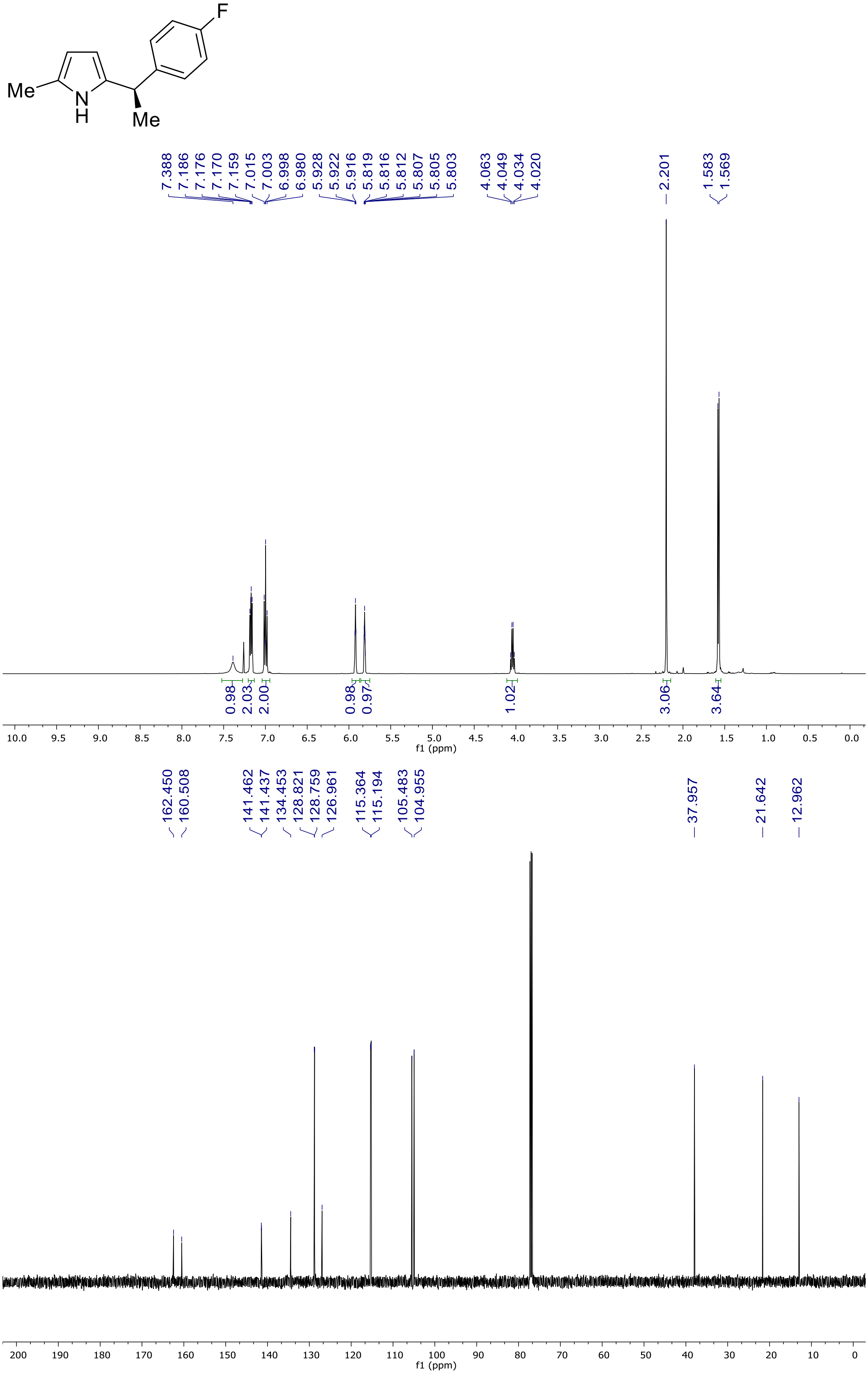

Supplementary Figure 57. ^{1}H NMR and ^{13}C NMR spectra of **3af**.

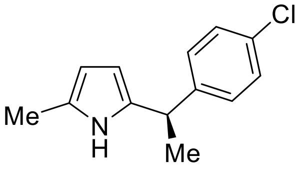

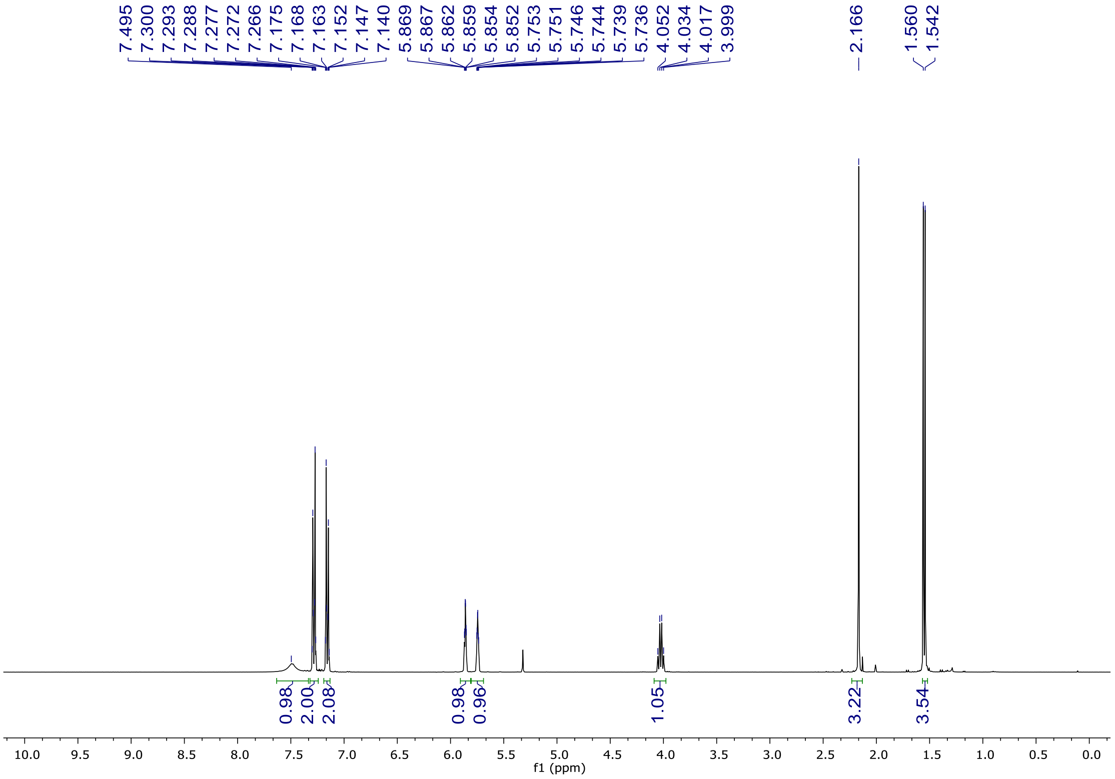

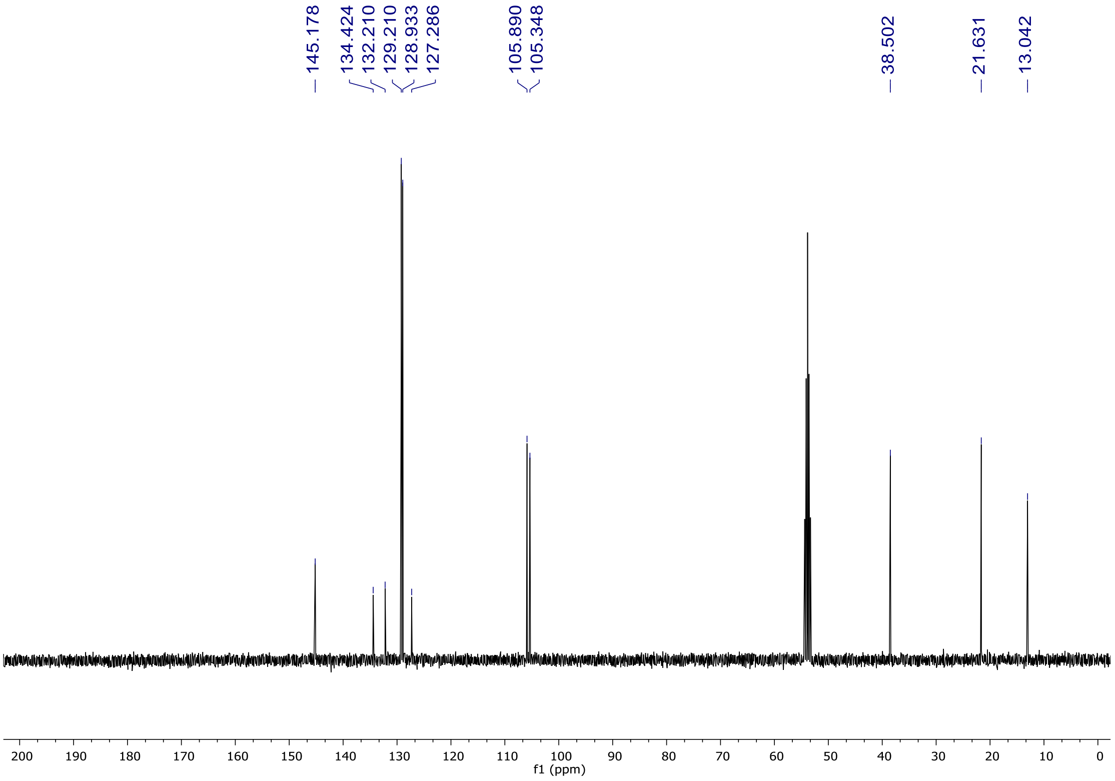

Supplementary Figure 58. ^{1}H NMR and ^{13}C NMR spectra of **3ag**.

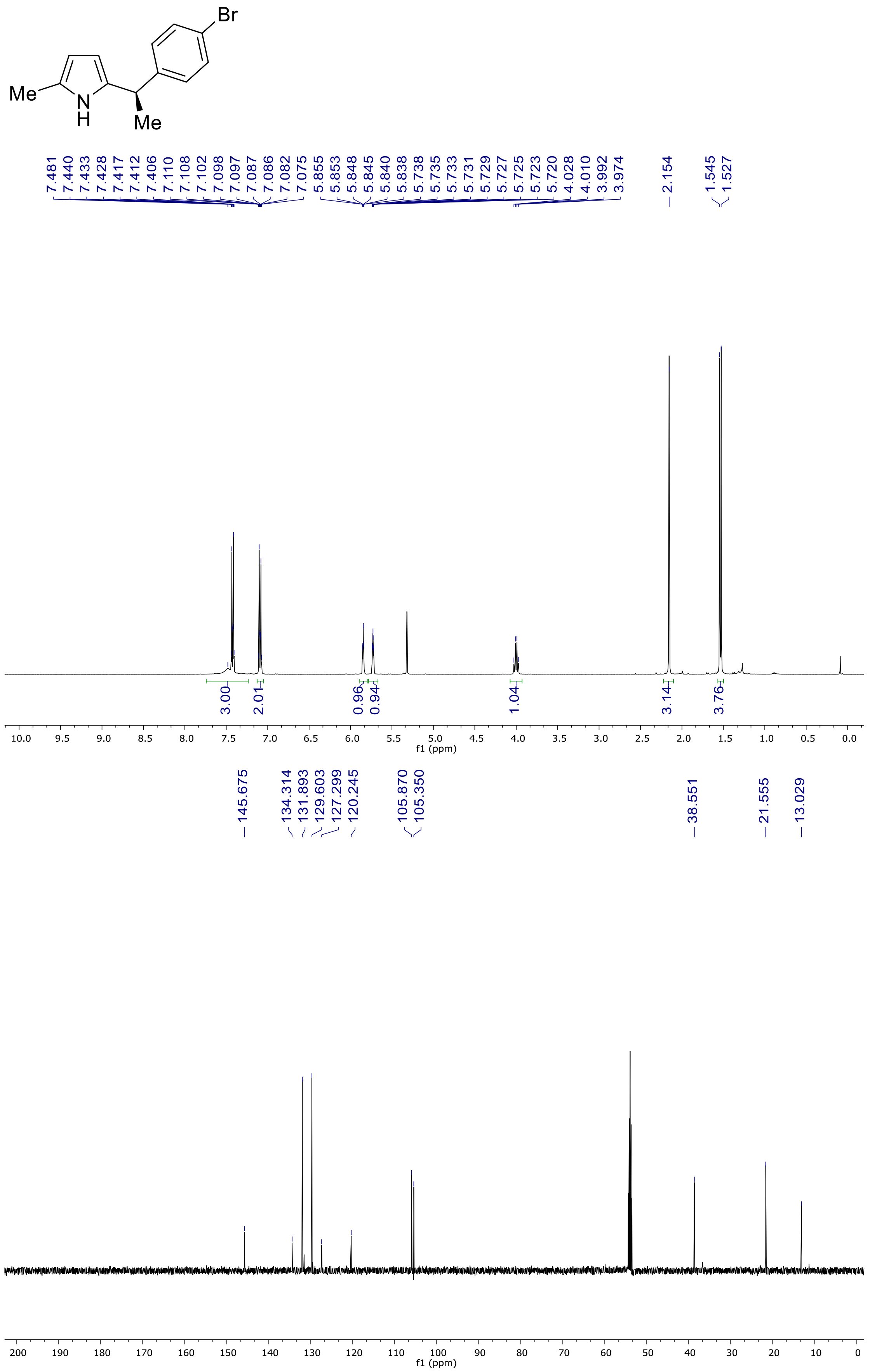

Supplementary Figure 59. ^{1}H NMR and ^{13}C NMR spectra of **3ah**.

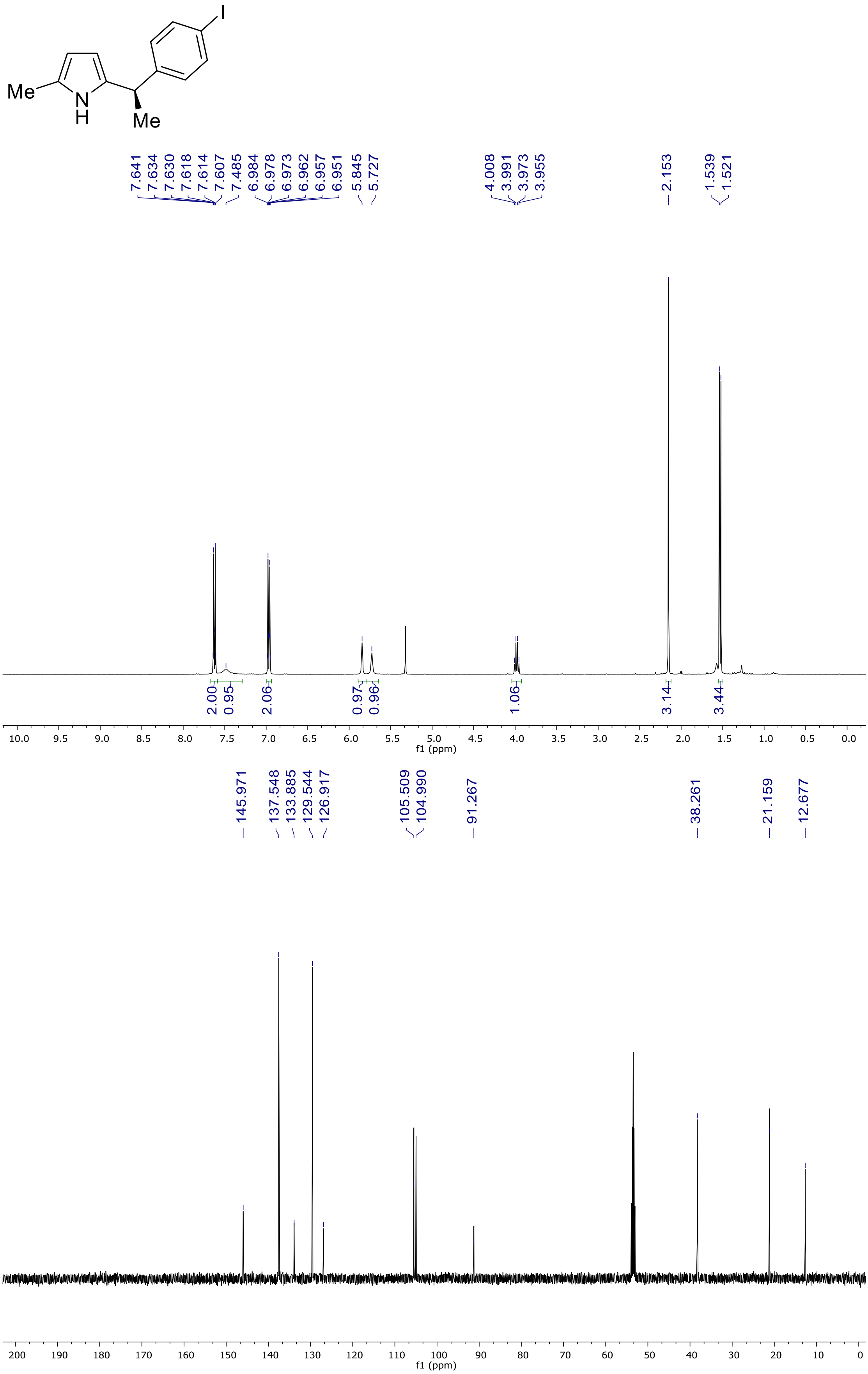

Supplementary Figure 60. ^{1}H NMR and ^{13}C NMR spectra of **3ai**.

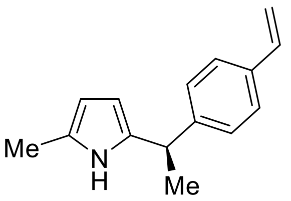

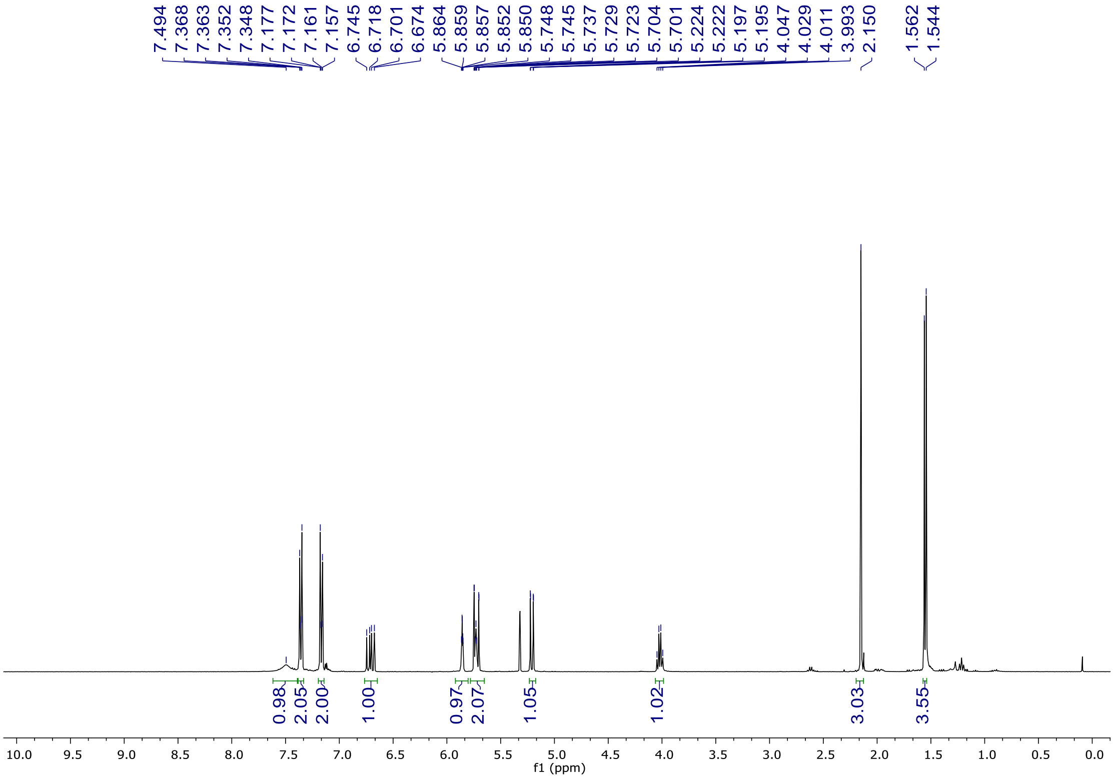

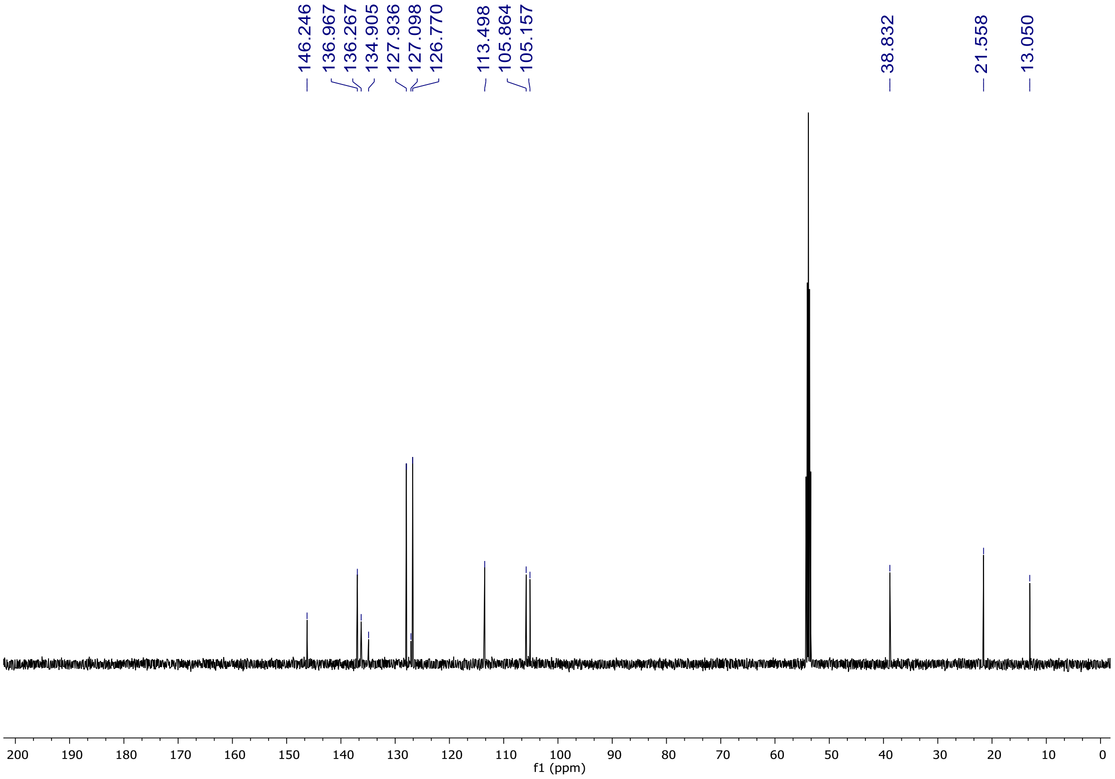

Supplementary Figure 61. ^{1}H NMR and ^{13}C NMR spectra of **3aj**.

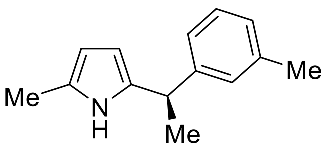

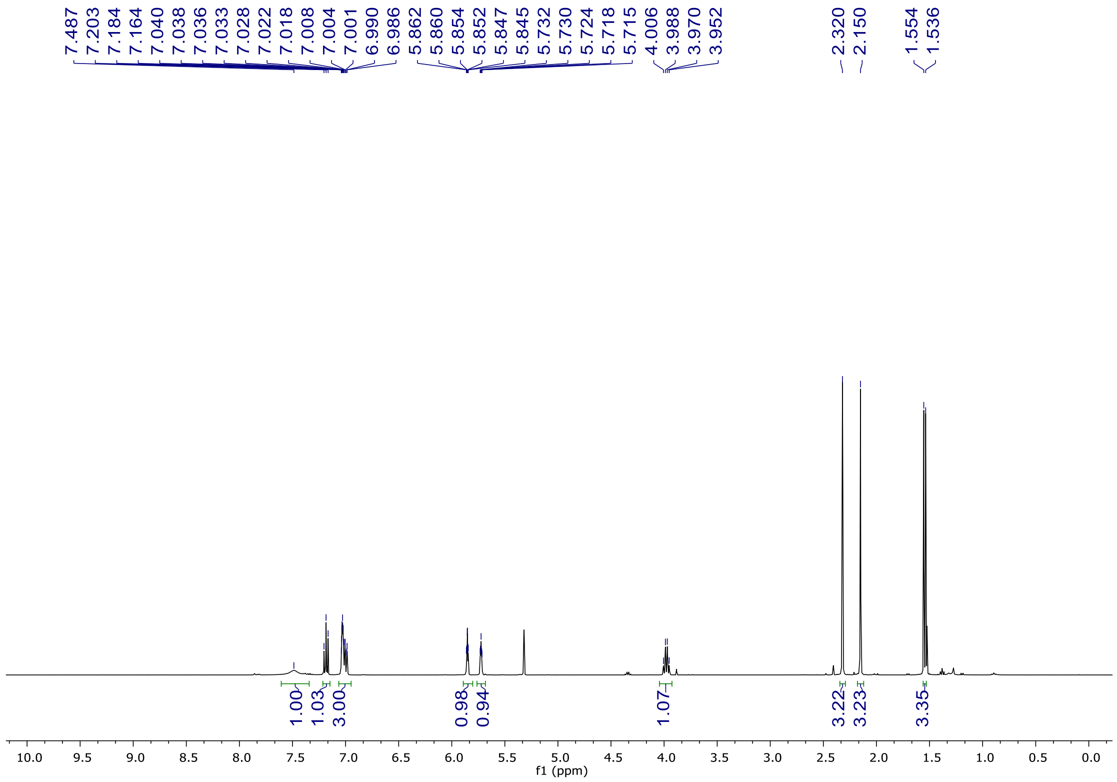

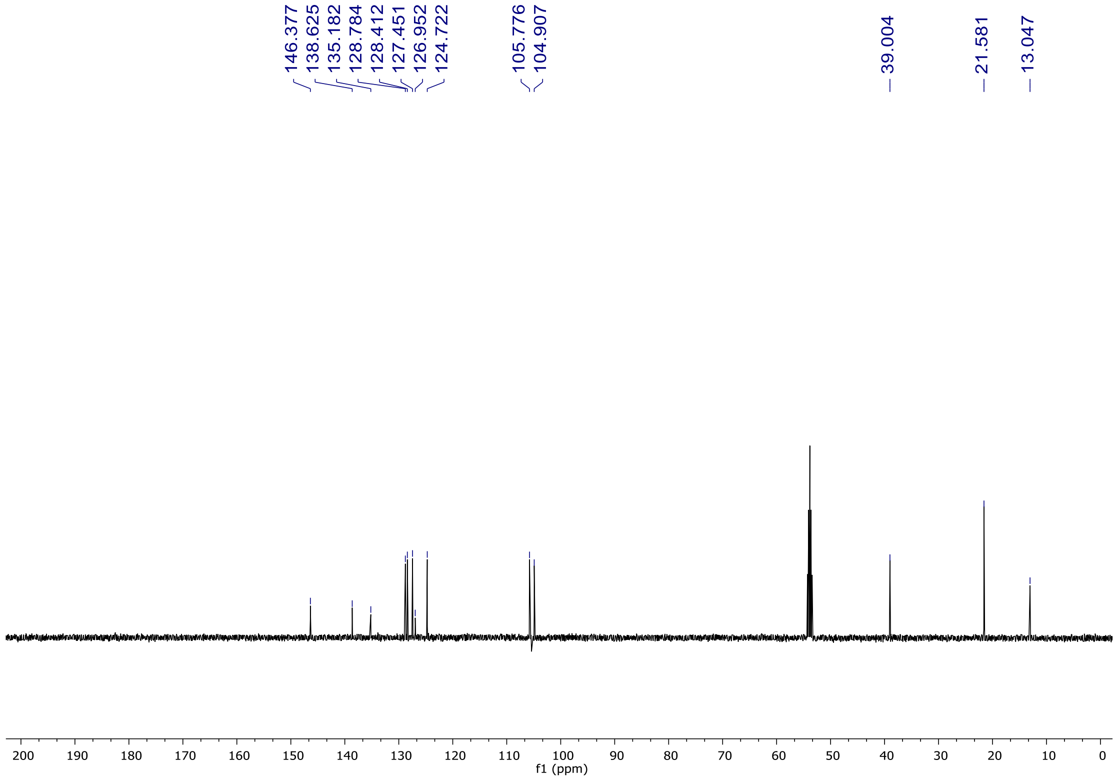

Supplementary Figure 62. ^{1}H NMR and ^{13}C NMR spectra of **3ak**.

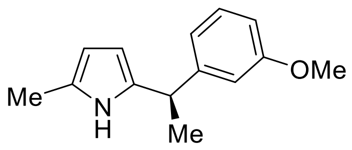

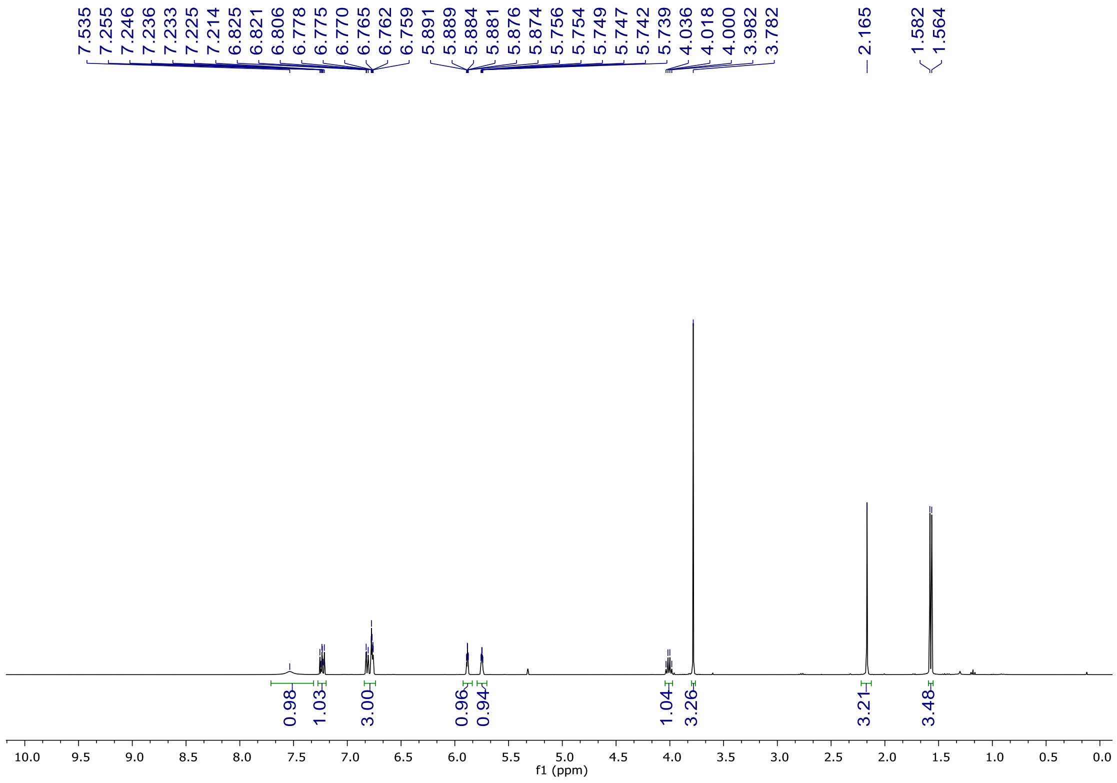

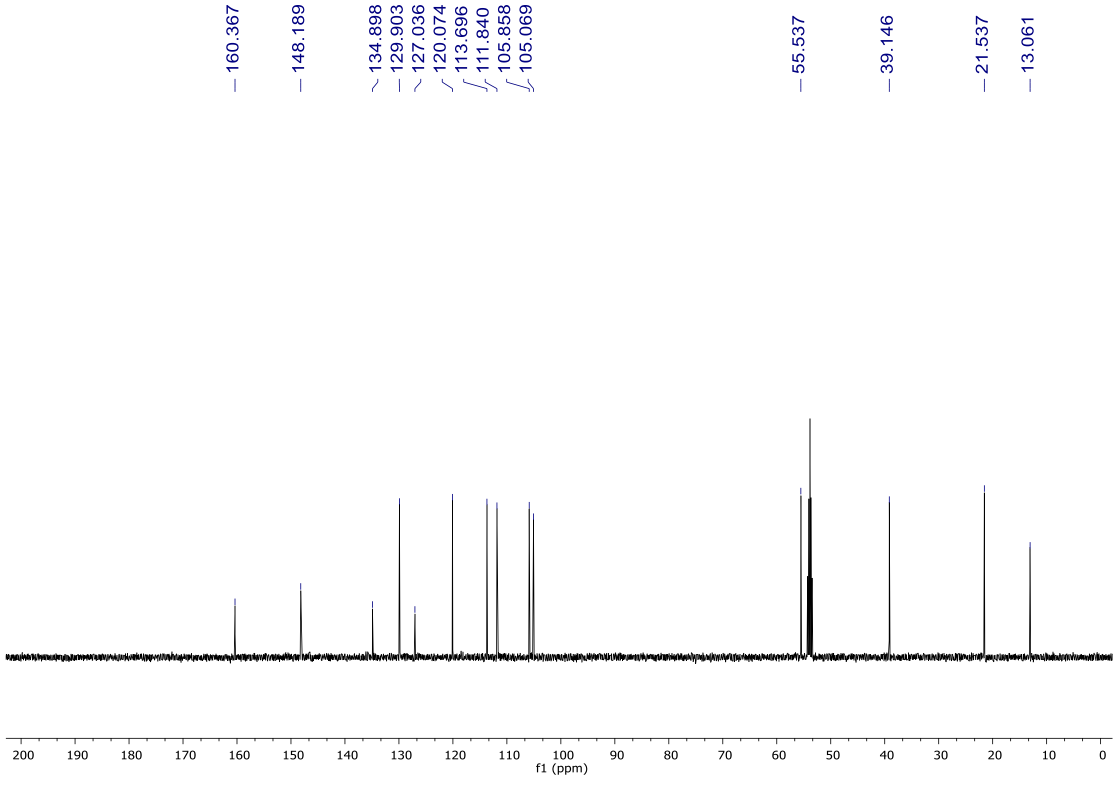

Supplementary Figure 63. ^{1}H NMR and ^{13}C NMR spectra of **3al**.

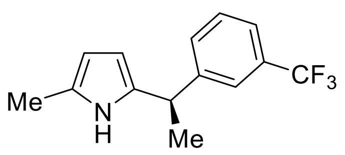

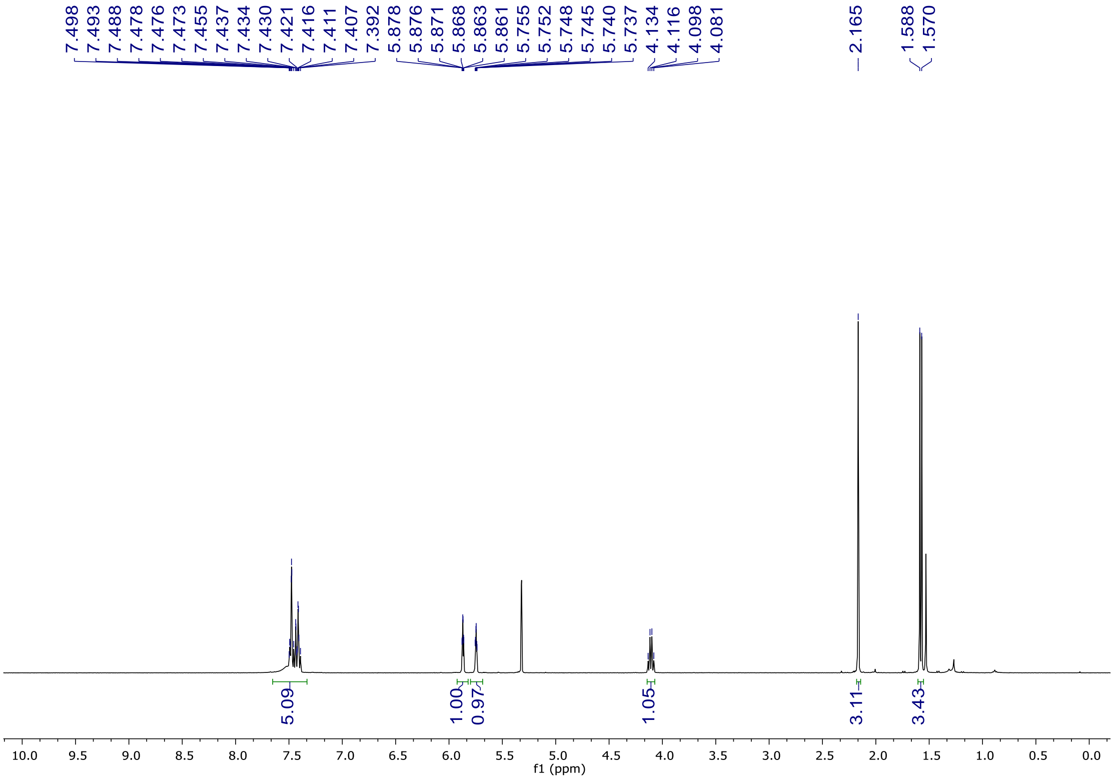

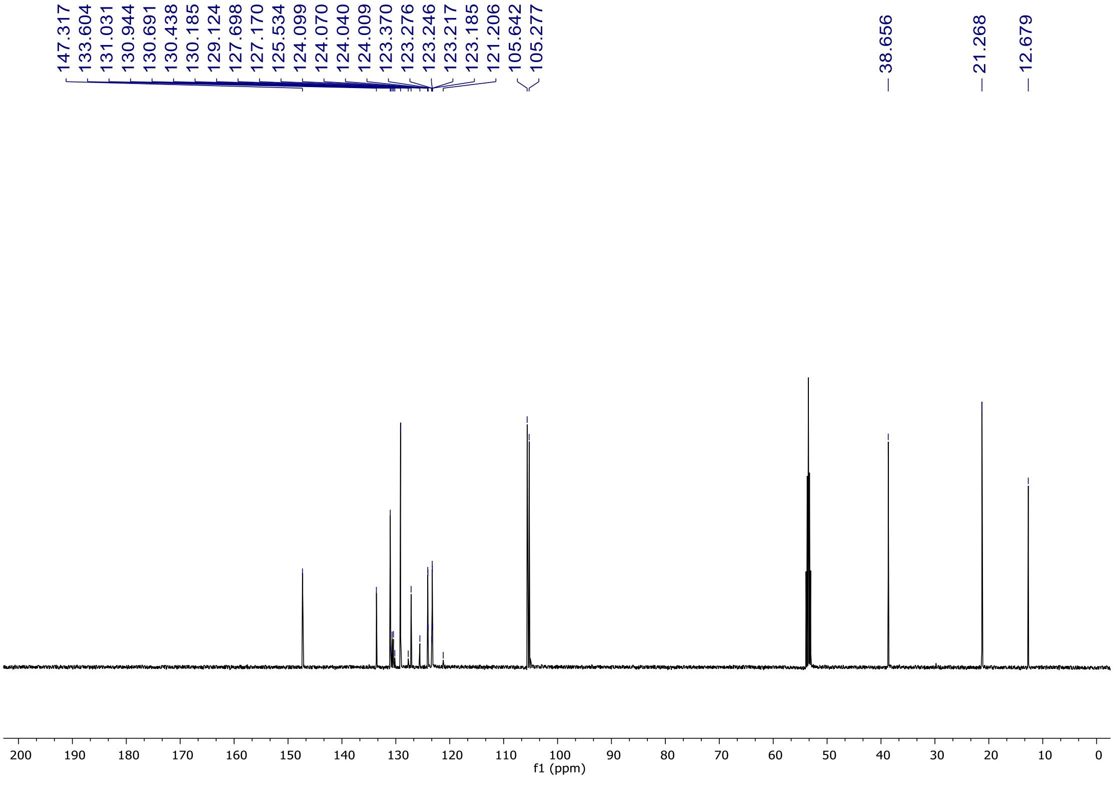

Supplementary Figure 64. ^{1}H NMR and ^{13}C NMR spectra of **3am**.

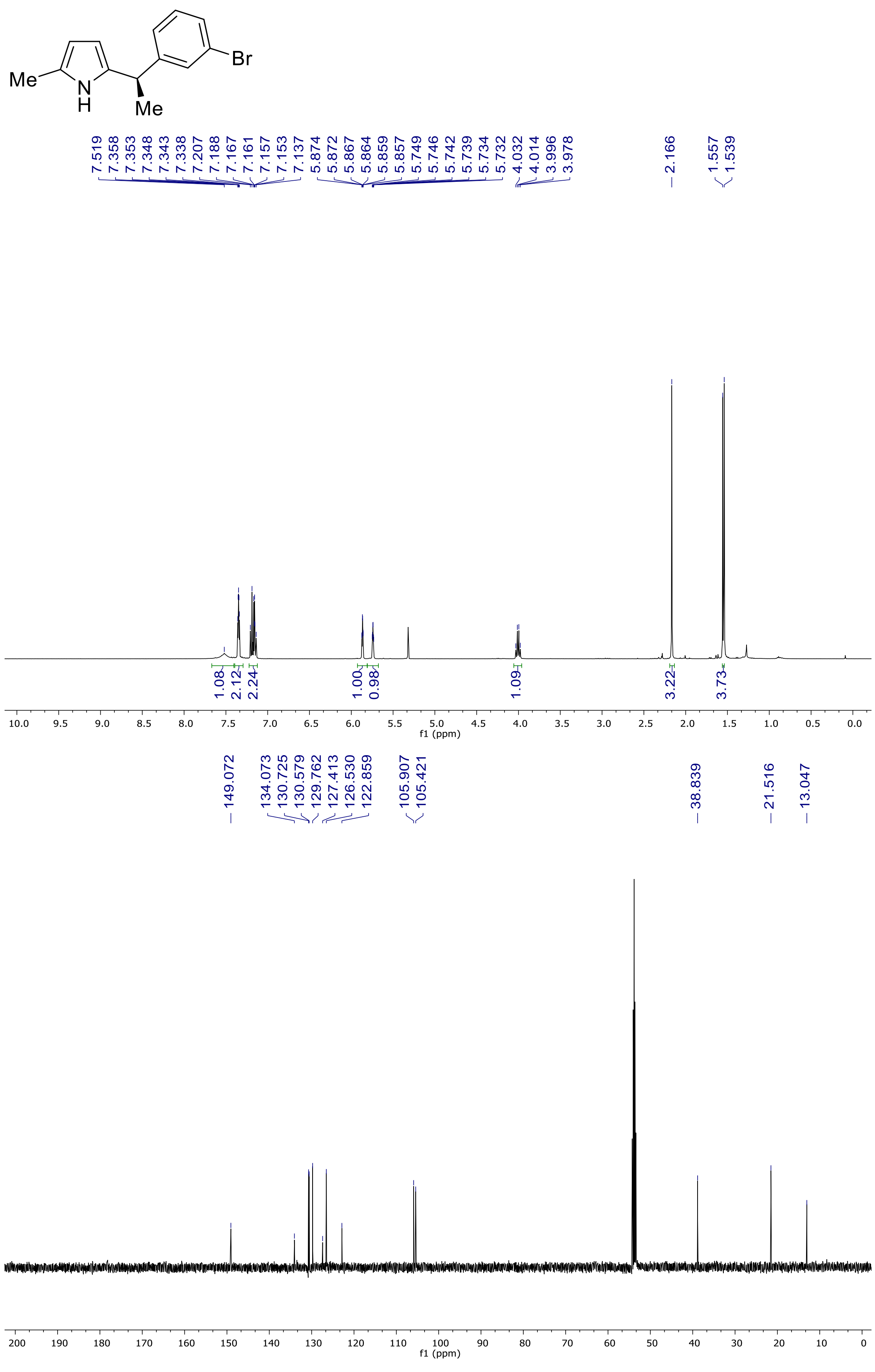

Supplementary Figure 65. ^{1}H NMR and ^{13}C NMR spectra of **3an**.

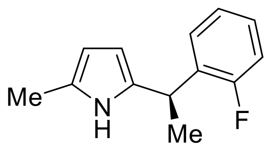

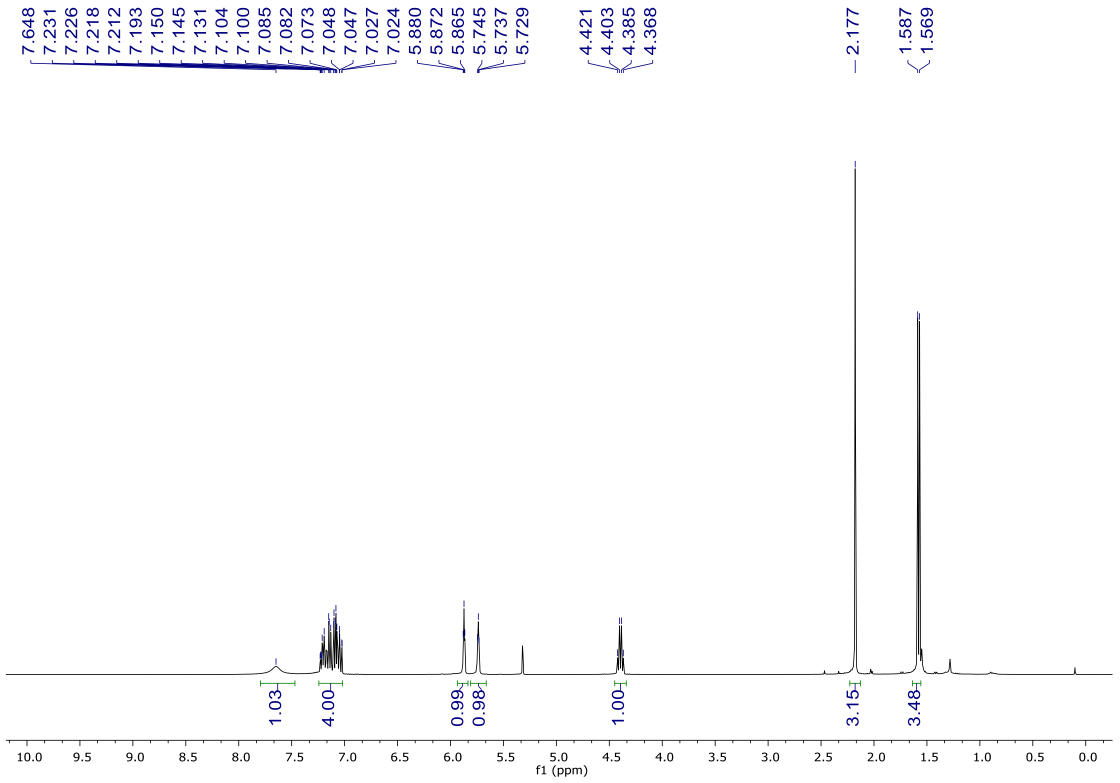

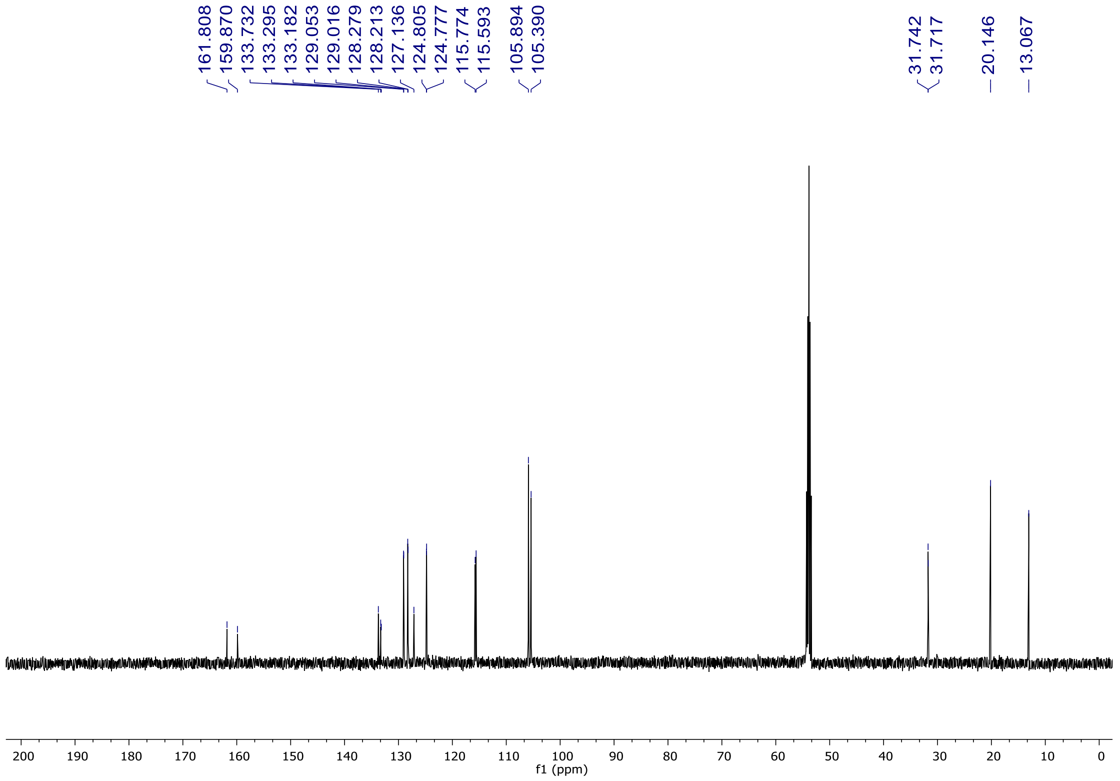

Supplementary Figure 66. ^{1}H NMR and ^{13}C NMR spectra of **3ao**.

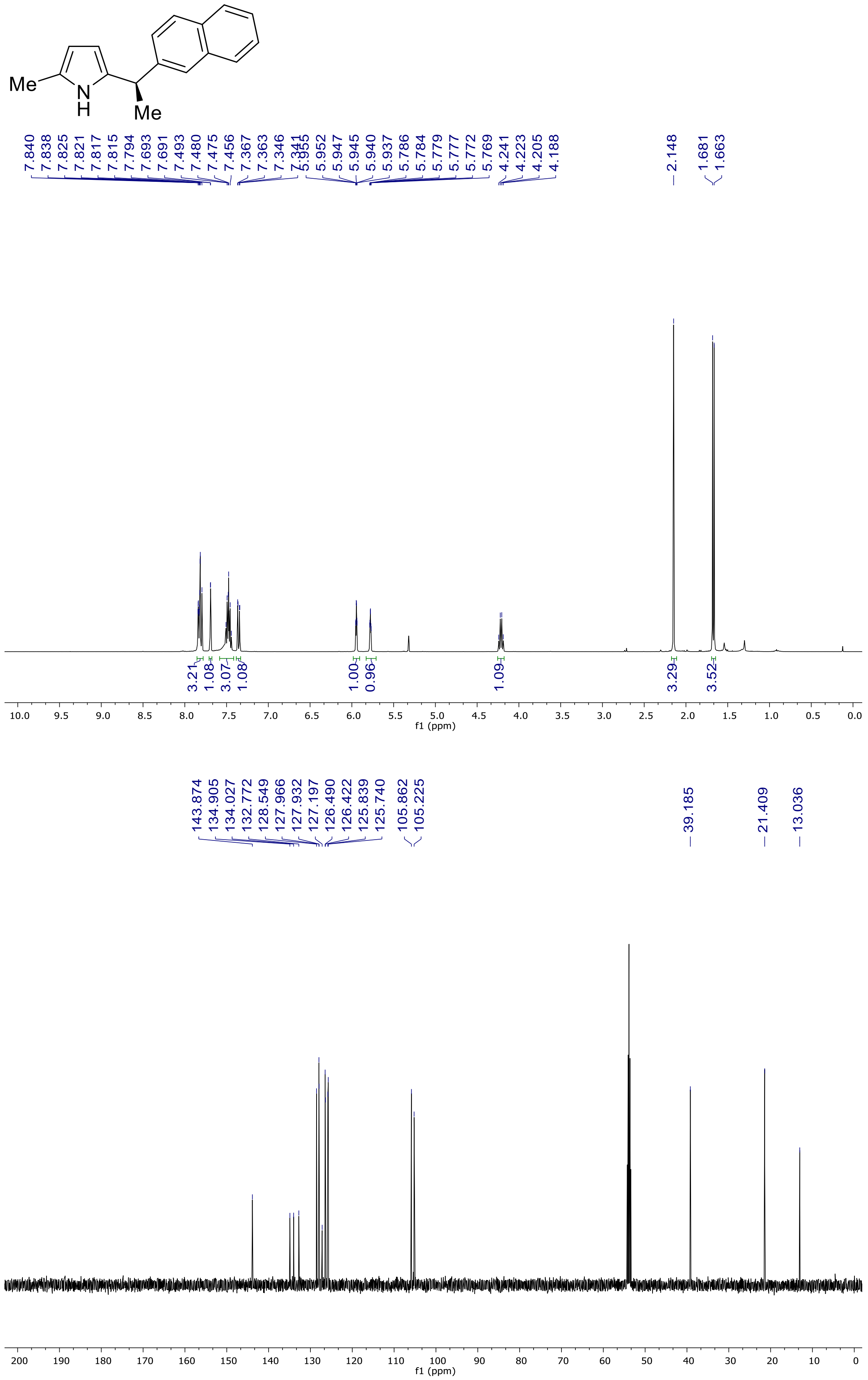

Supplementary Figure 67. ^{1}H NMR and ^{13}C NMR spectra of **3ap**.

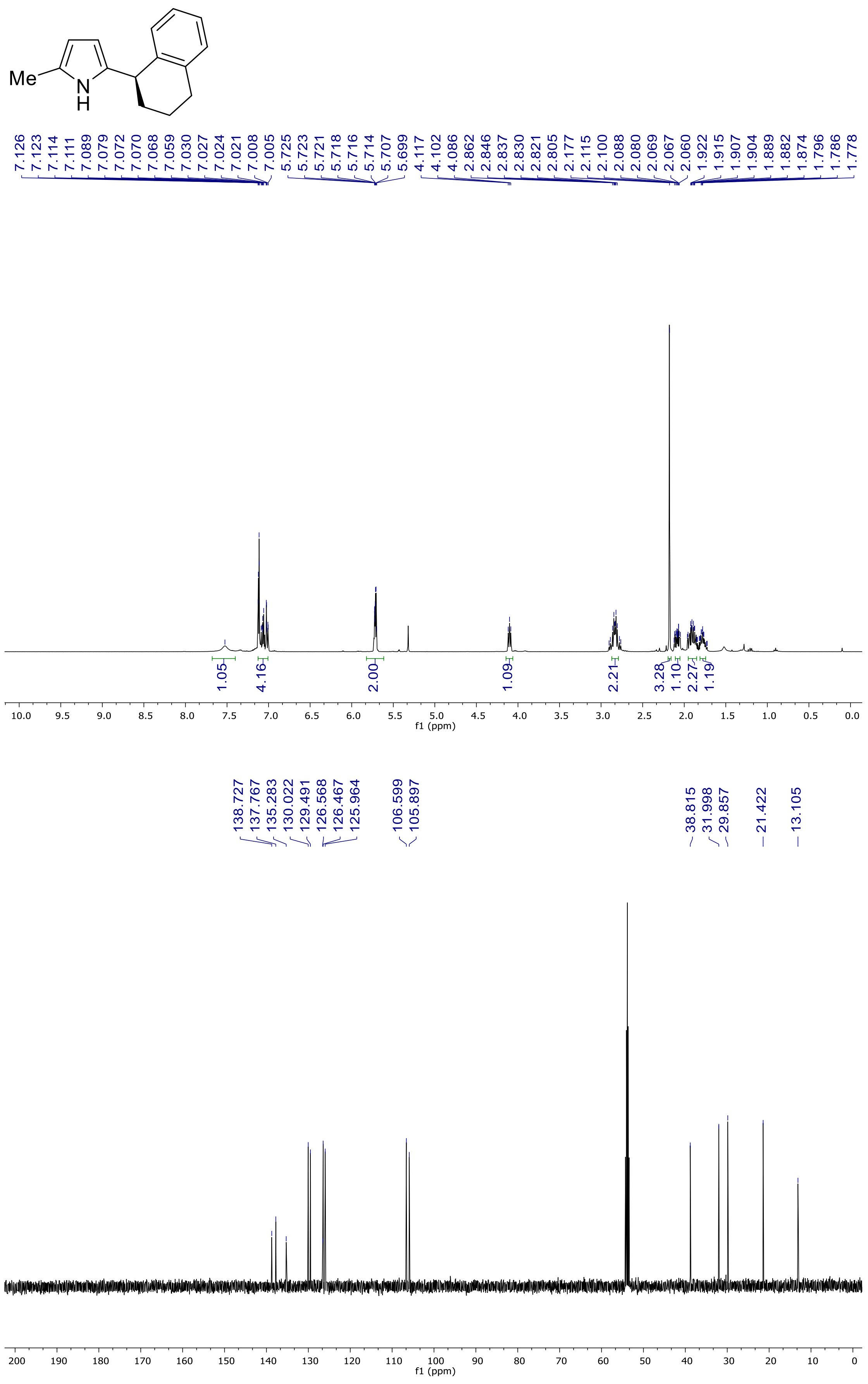

Supplementary Figure 68. ^{1}H NMR and ^{13}C NMR spectra of **3aq**.

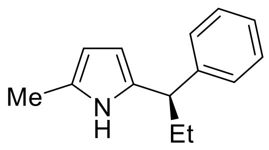

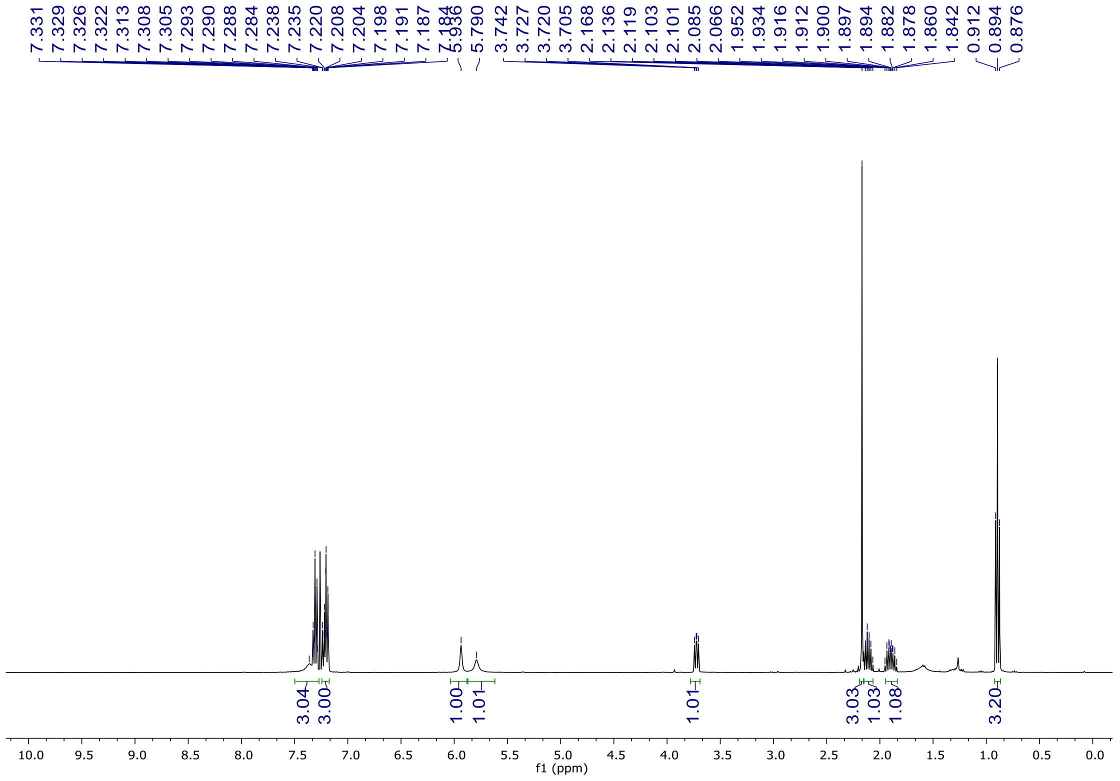

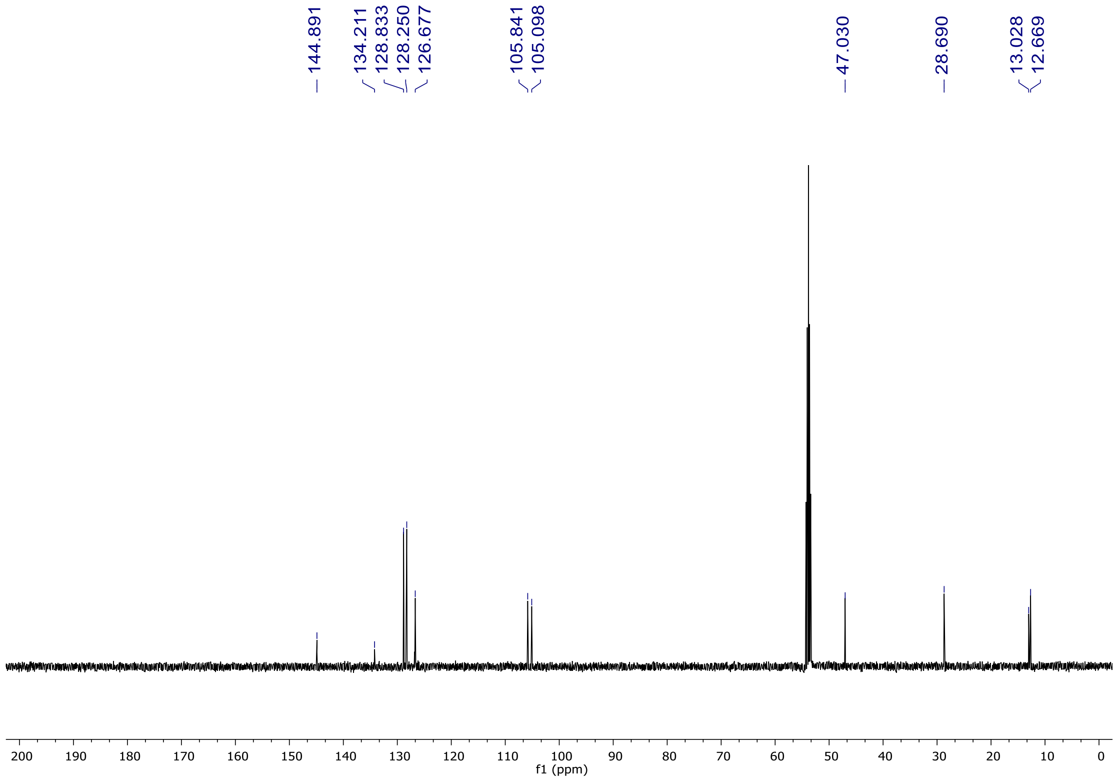

Supplementary Figure 69. ^{1}H NMR and ^{13}C NMR spectra of **3ar**.

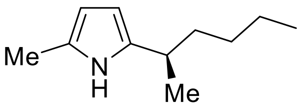

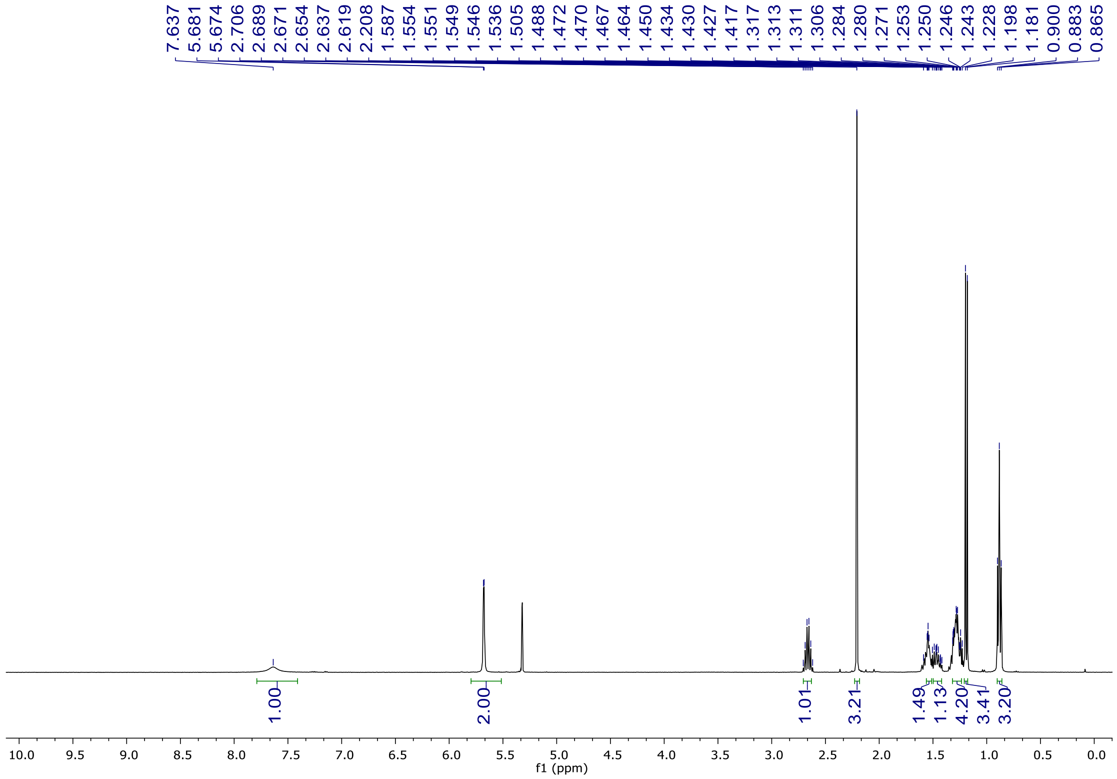

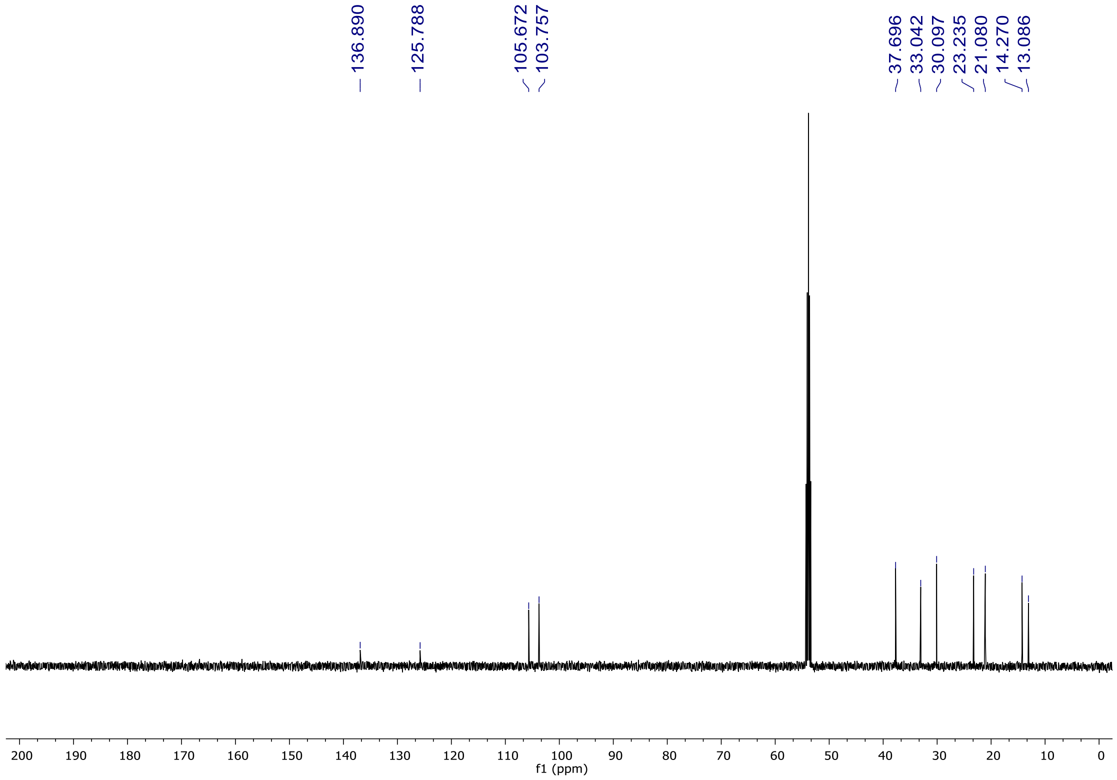

Supplementary Figure 70. ^{1}H NMR and ^{13}C NMR spectra of **3as**.

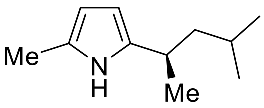

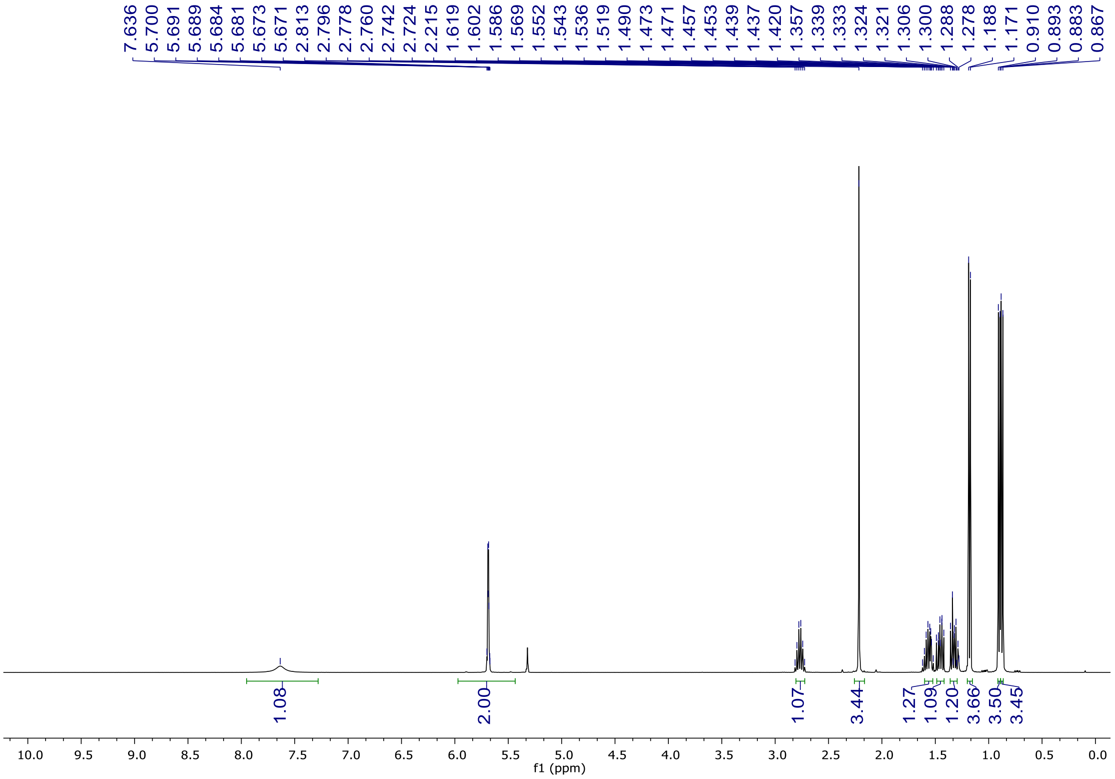

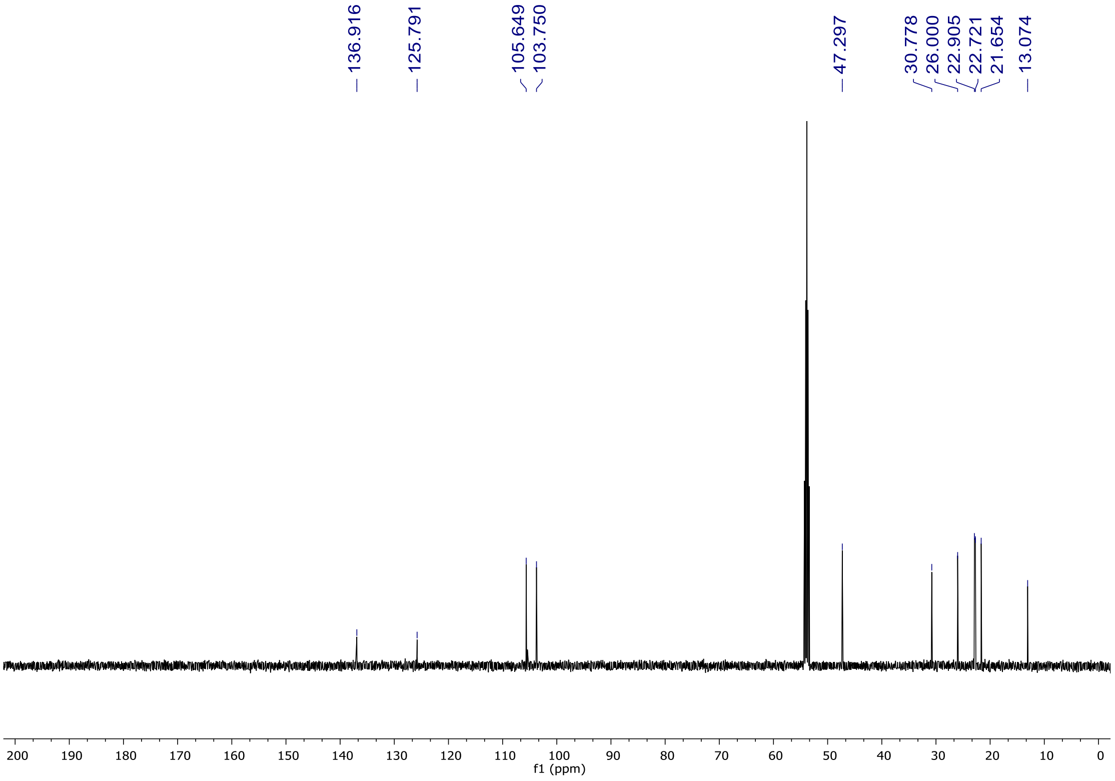

Supplementary Figure 71. ^{1}H NMR and ^{13}C NMR spectra of **3at**.

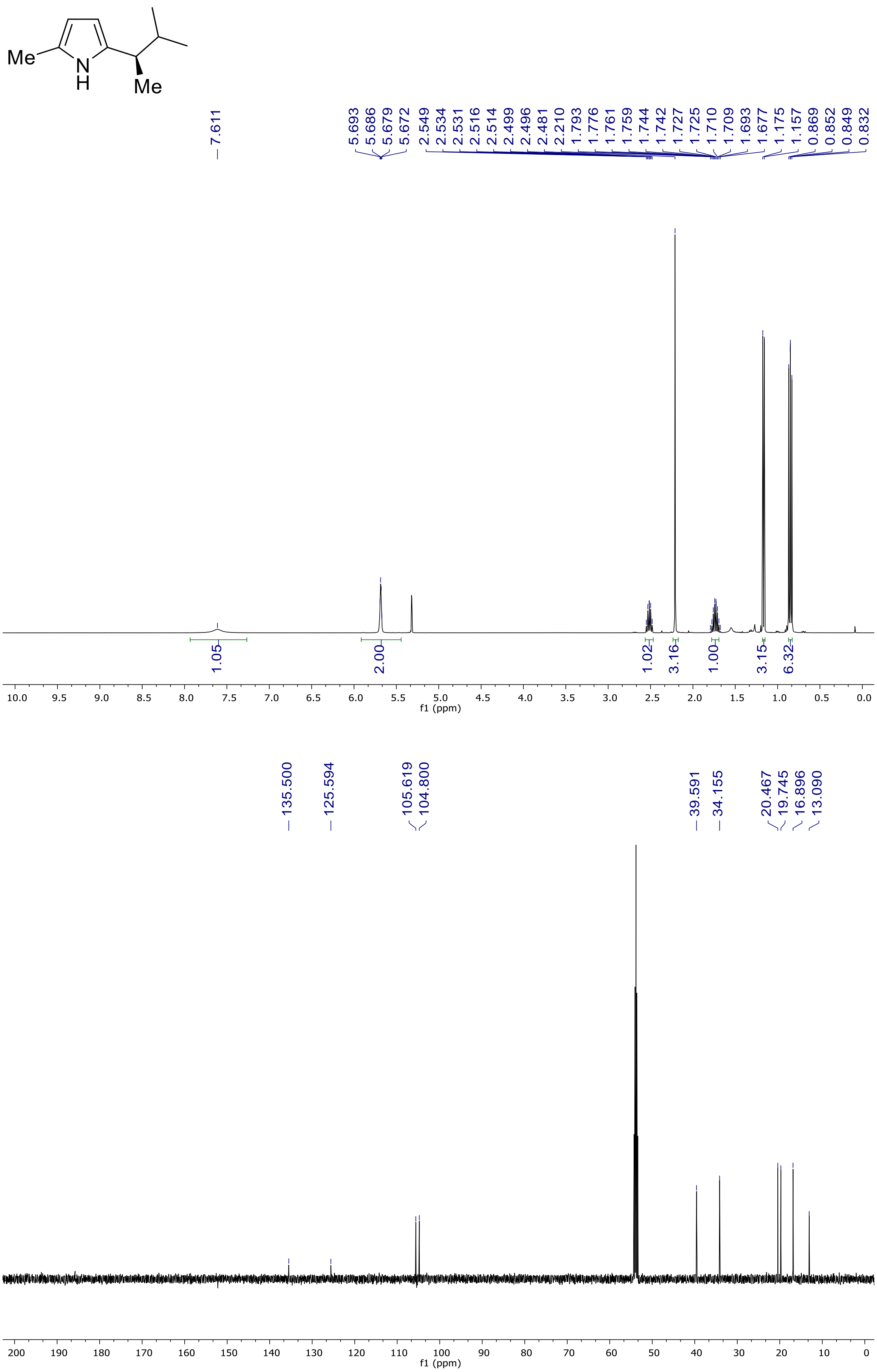

Supplementary Figure 72. ^{1}H NMR and ^{13}C NMR spectra of **3au**.

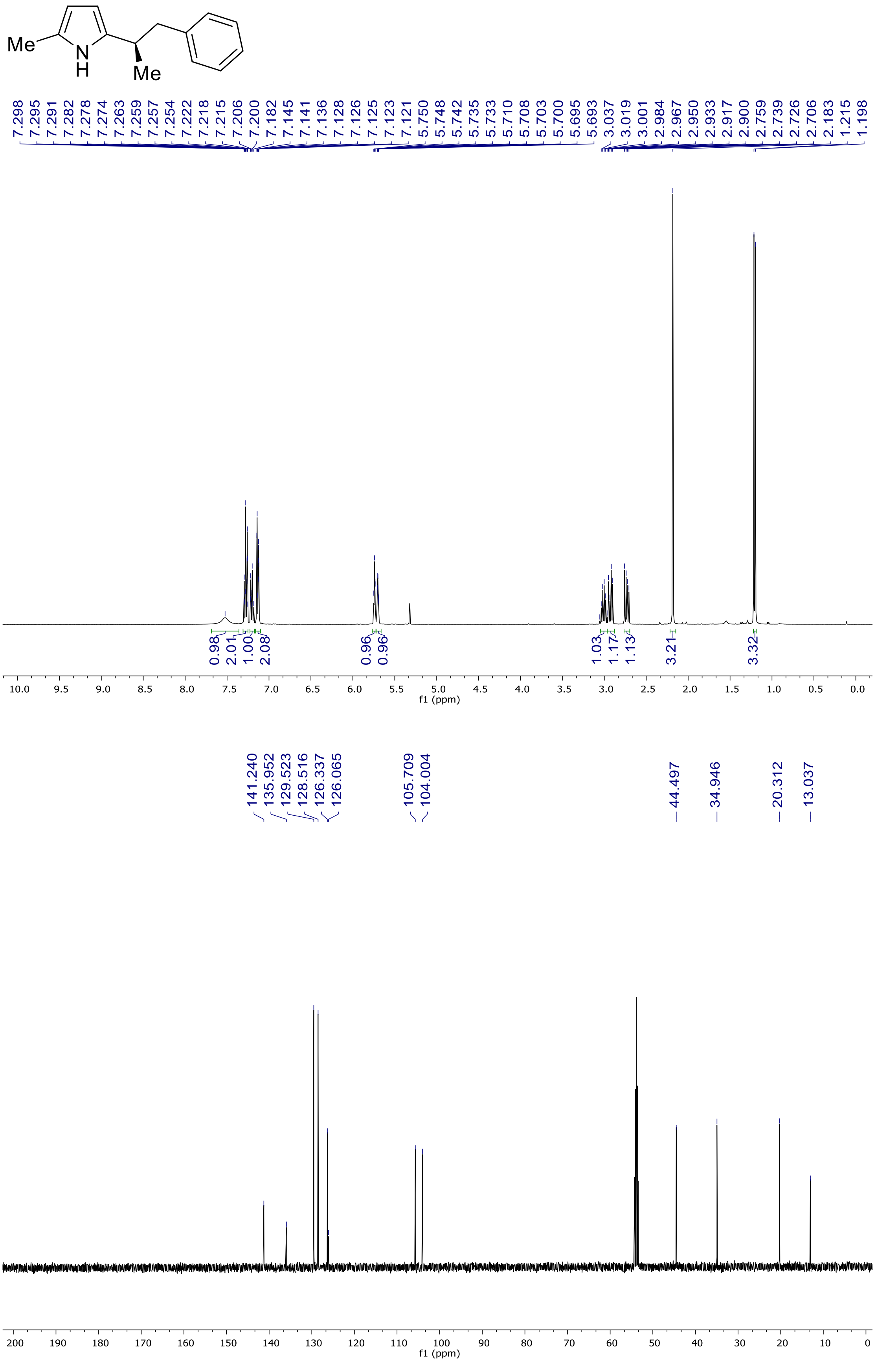

Supplementary Figure 73. ^{1}H NMR and ^{13}C NMR spectra of **3av**.

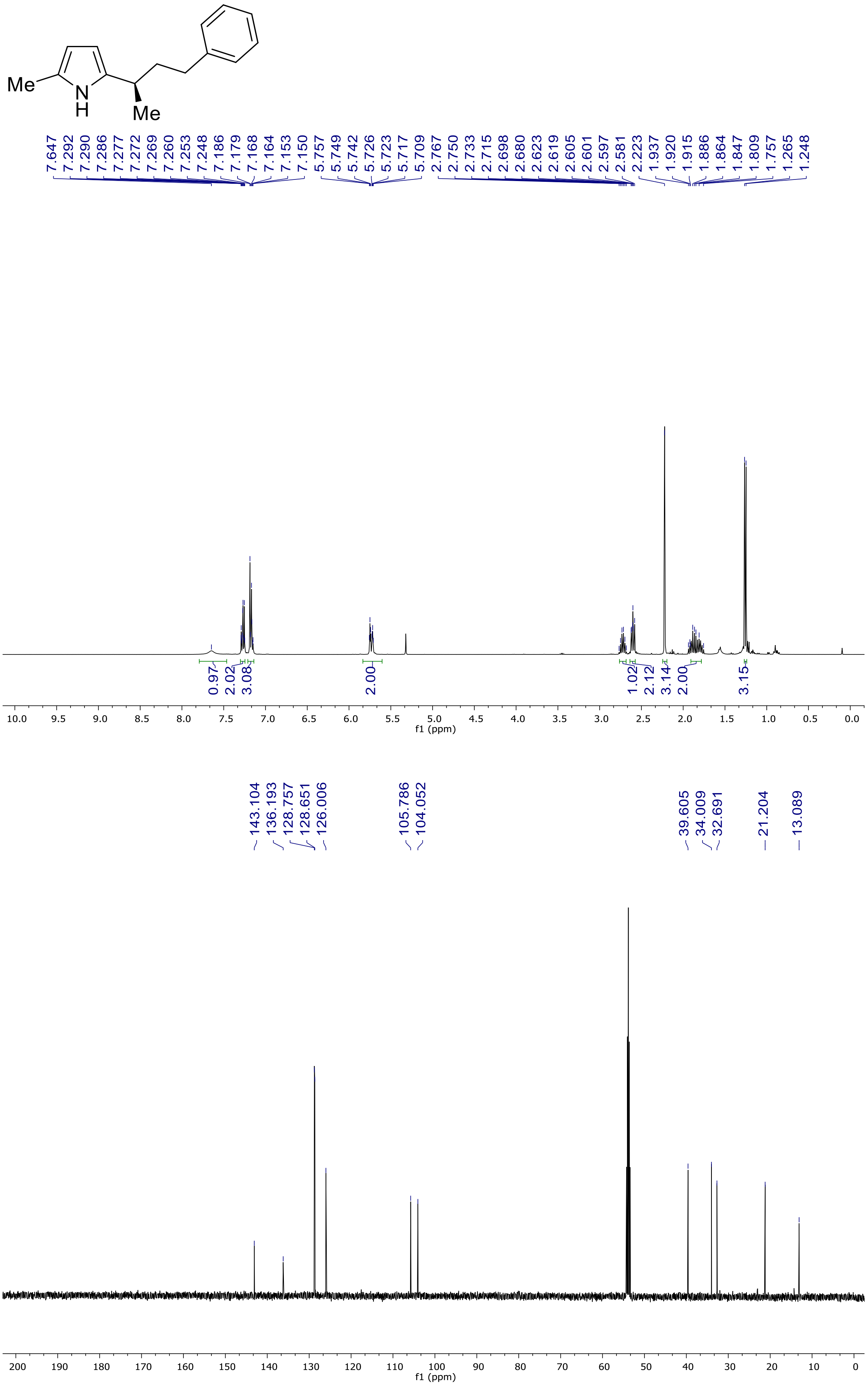

Supplementary Figure 74. ^{1}H NMR and ^{13}C NMR spectra of **3aw**.

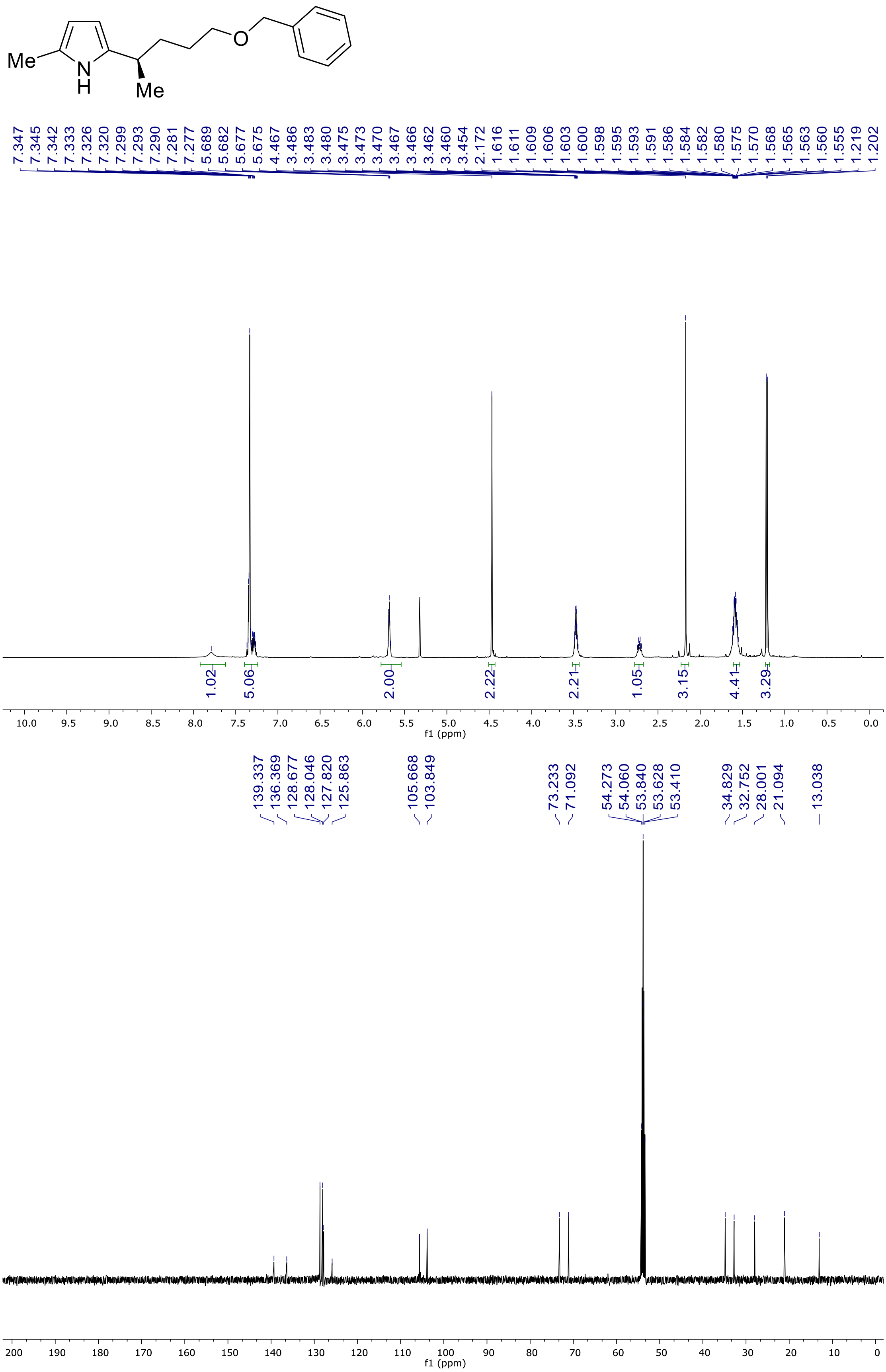

Supplementary Figure 75. ^{1}H NMR and ^{13}C NMR spectra of **3ax**.

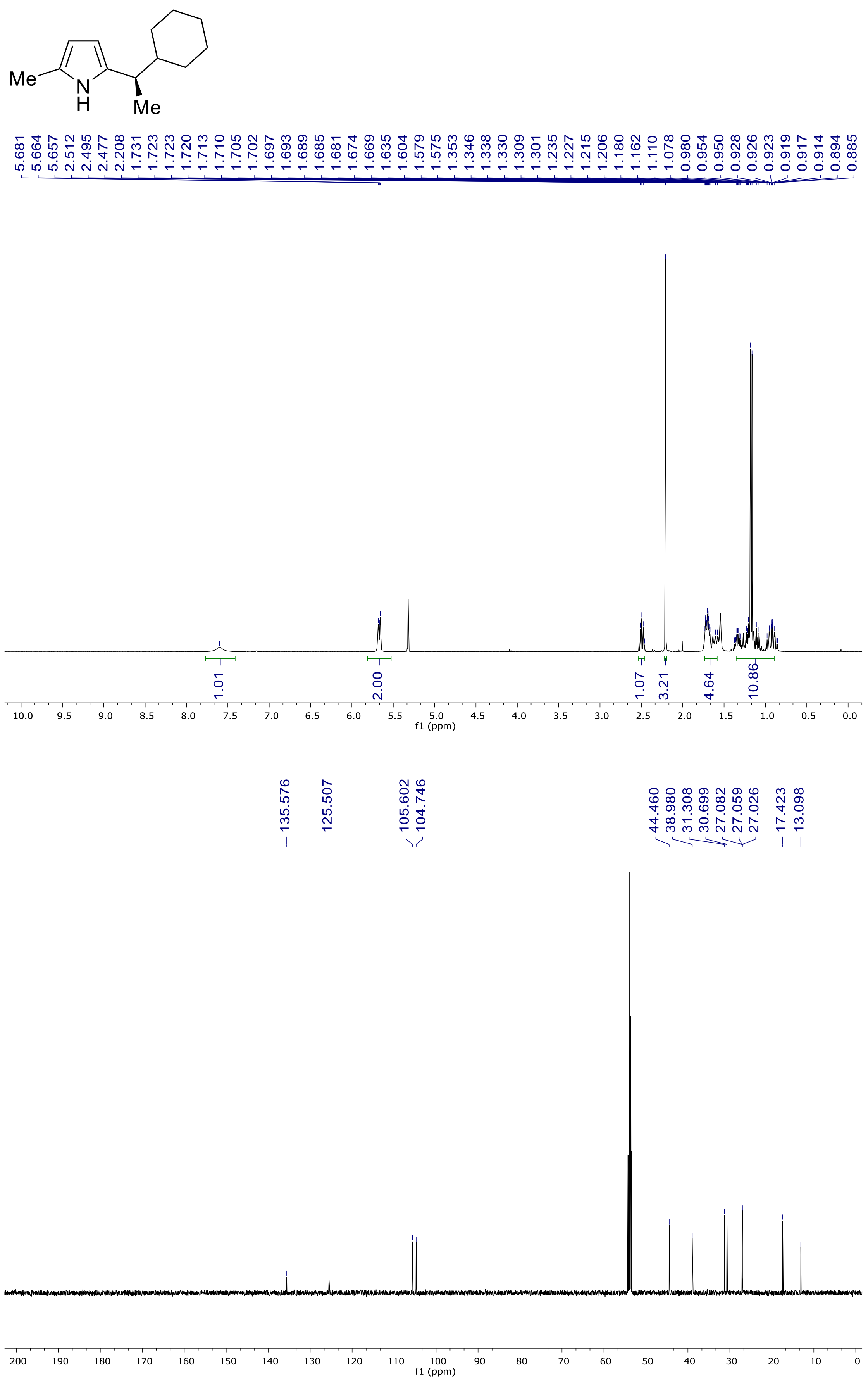

Supplementary Figure 76. ^{1}H NMR and ^{13}C NMR spectra of **3ay**.

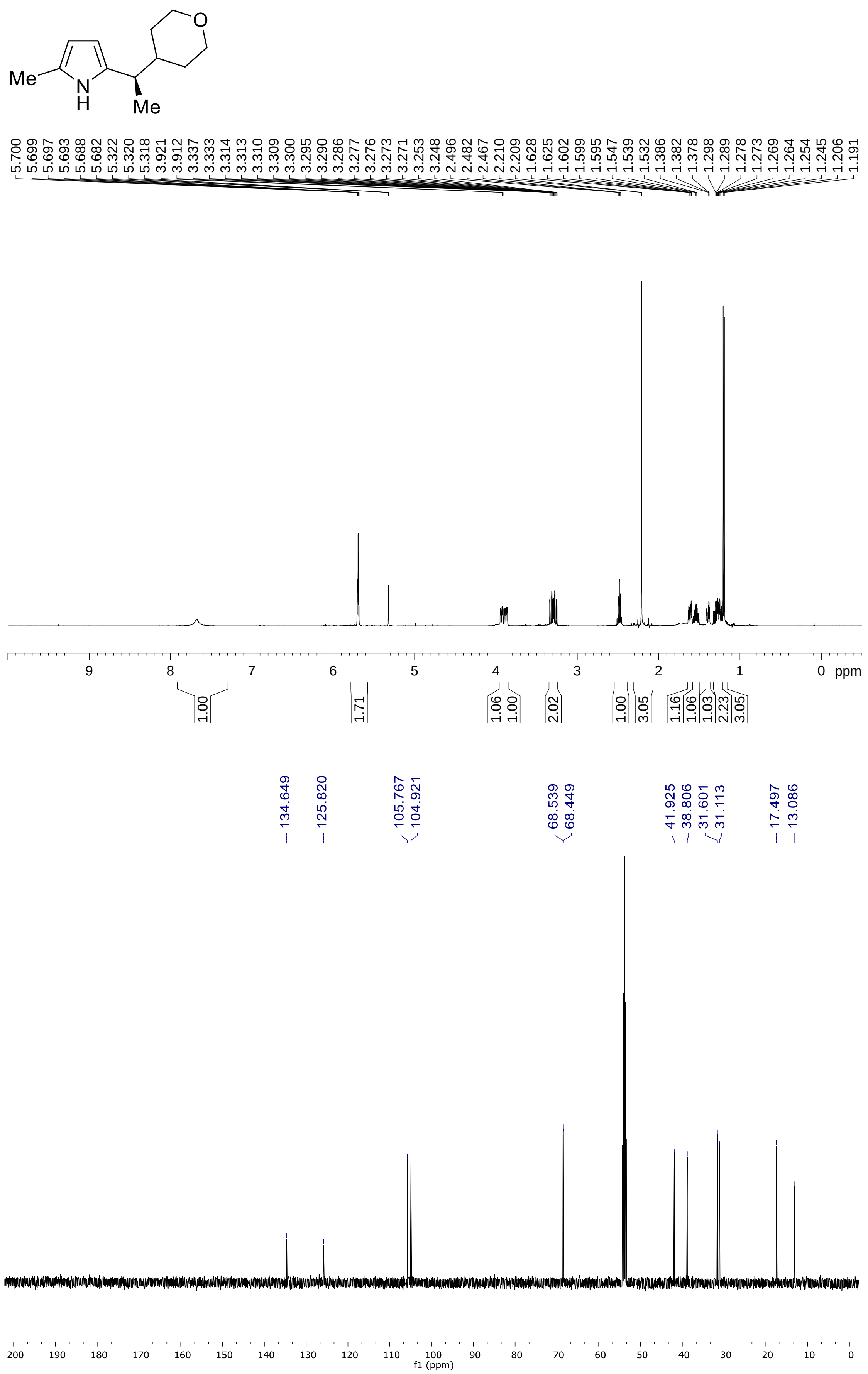

Supplementary Figure 77. ^{1}H NMR and ^{13}C NMR spectra of **3az**.

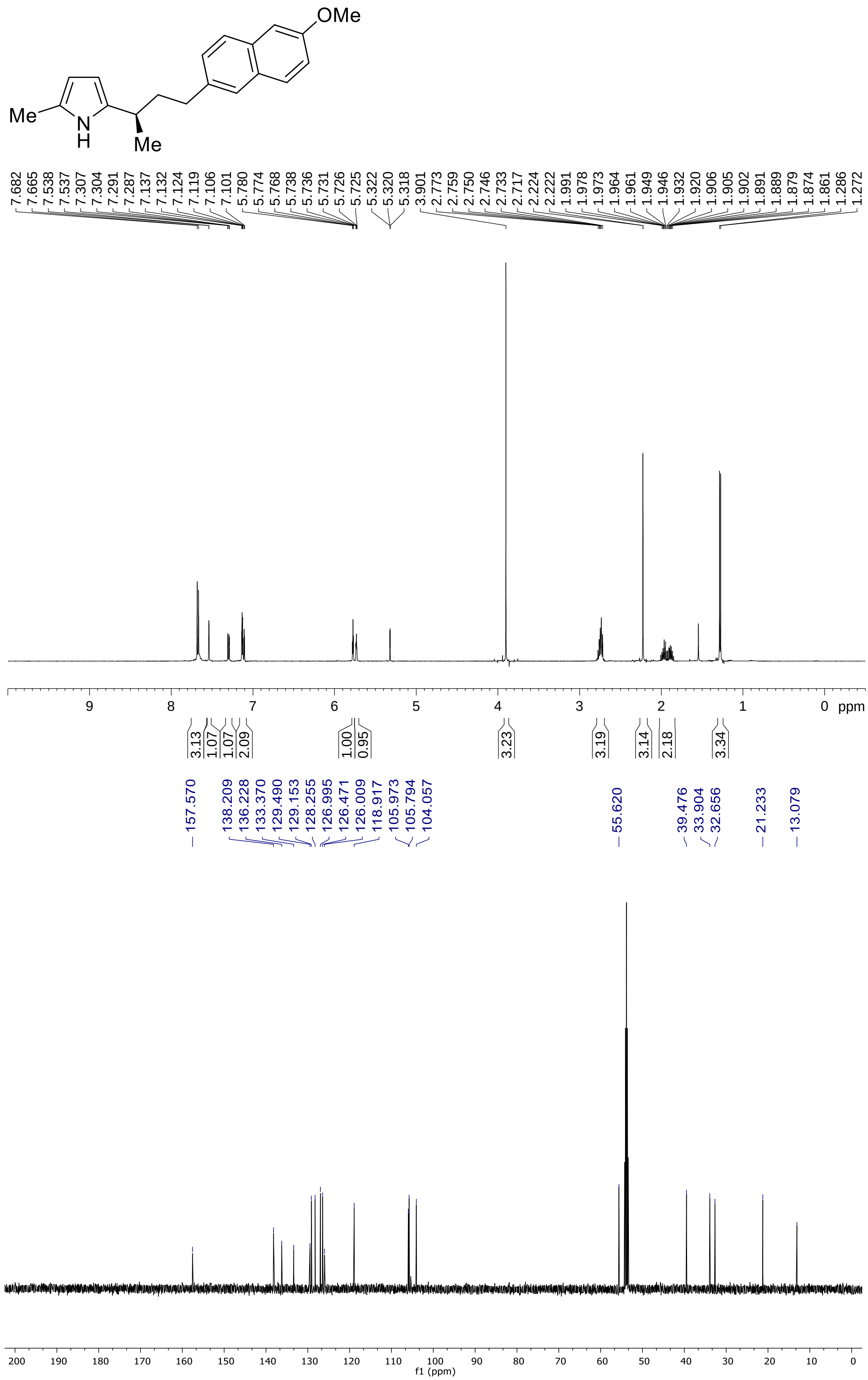

Supplementary Figure 78. ^{1}H NMR and ^{13}C NMR spectra of **3aA**.

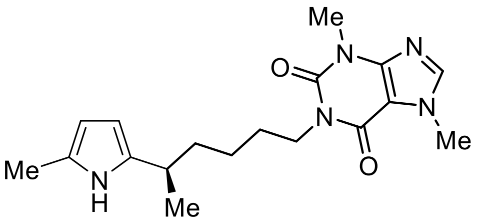

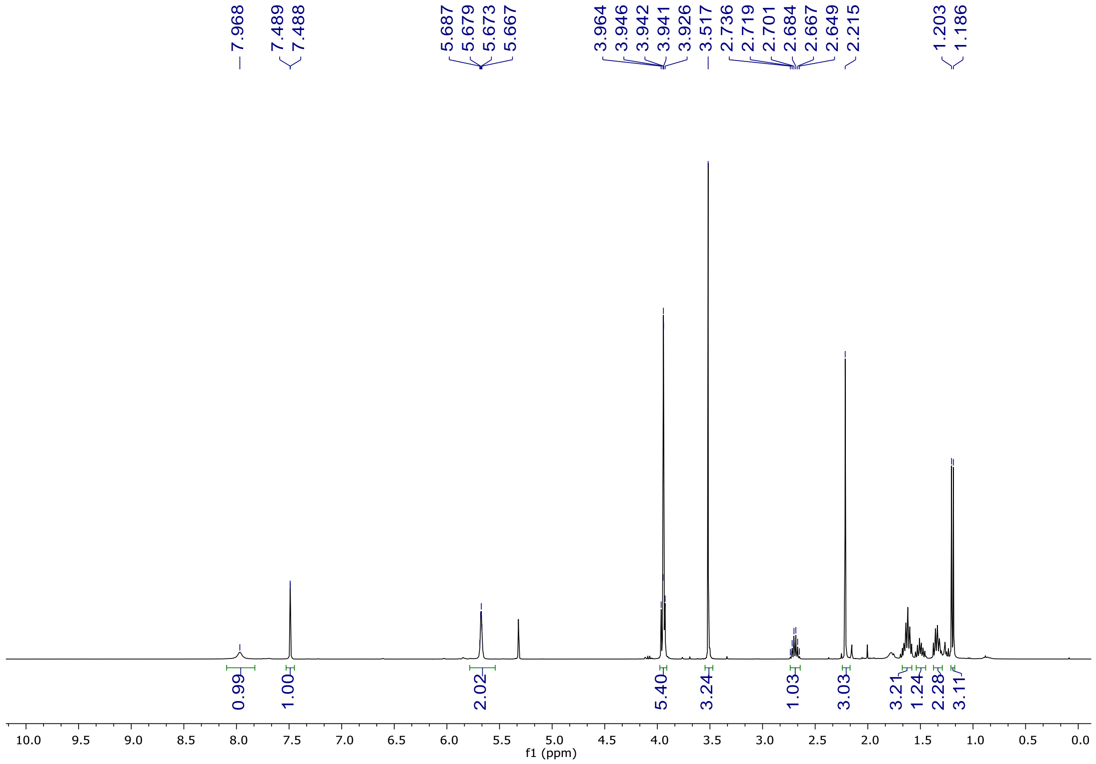

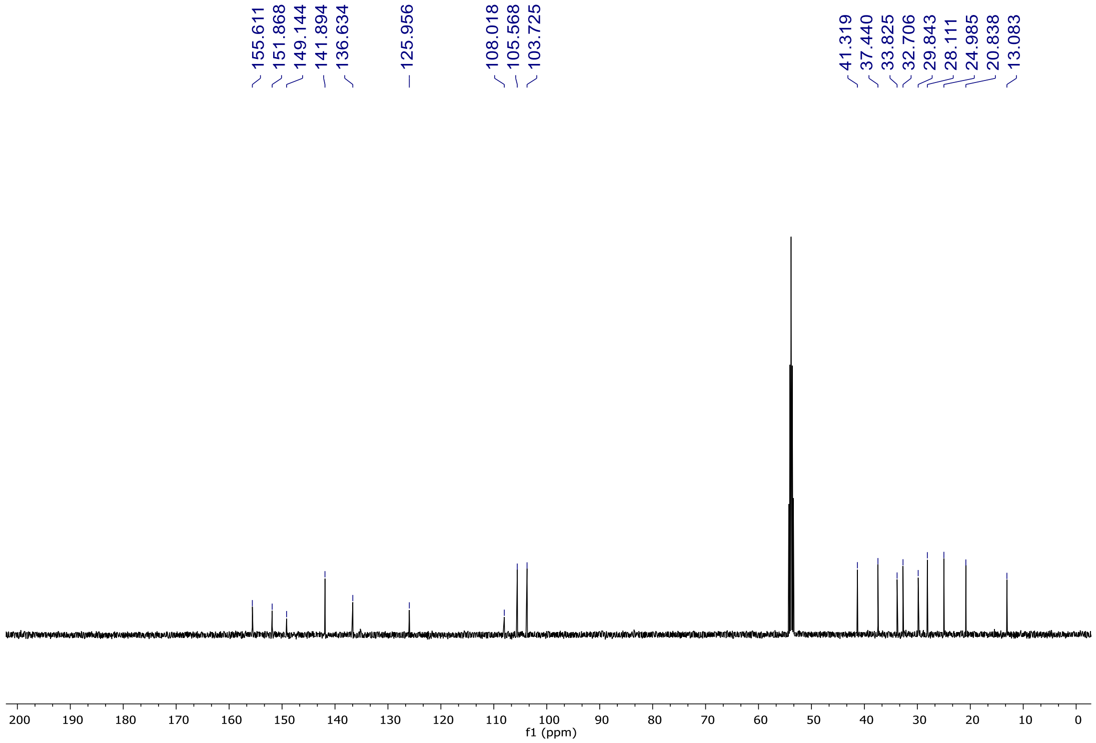

Supplementary Figure 79. ^{1}H NMR and ^{13}C NMR spectra of **3aB**.

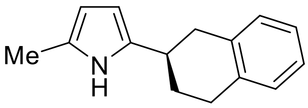

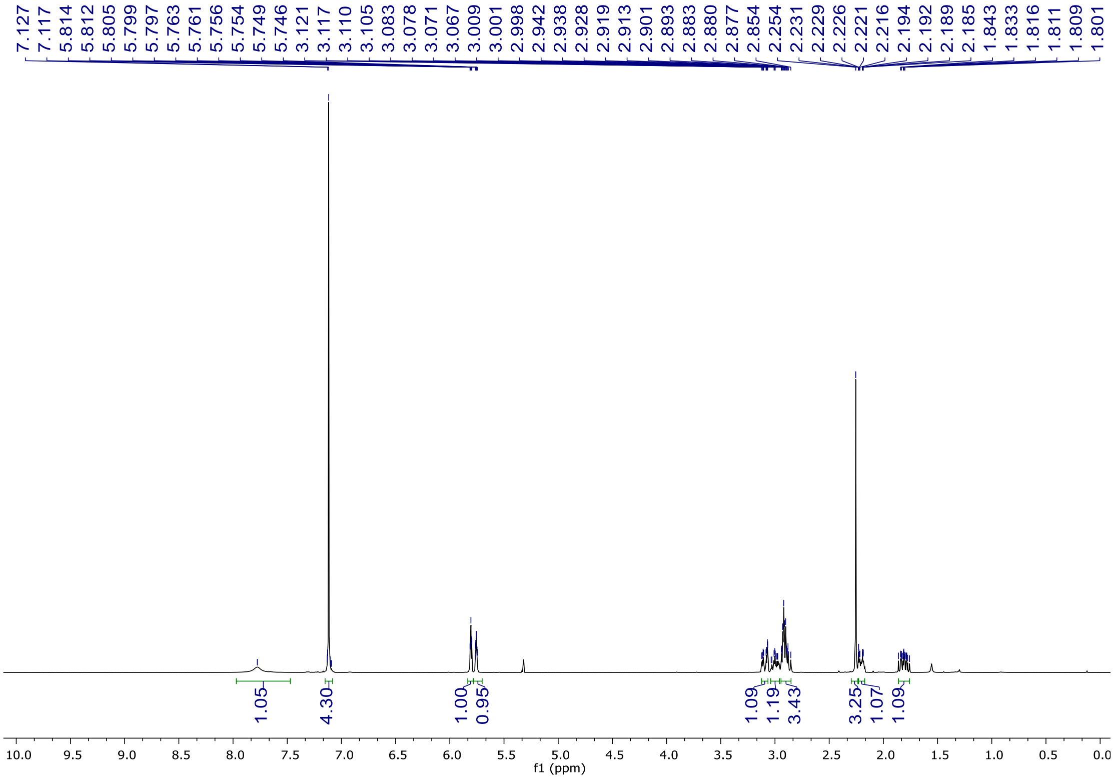

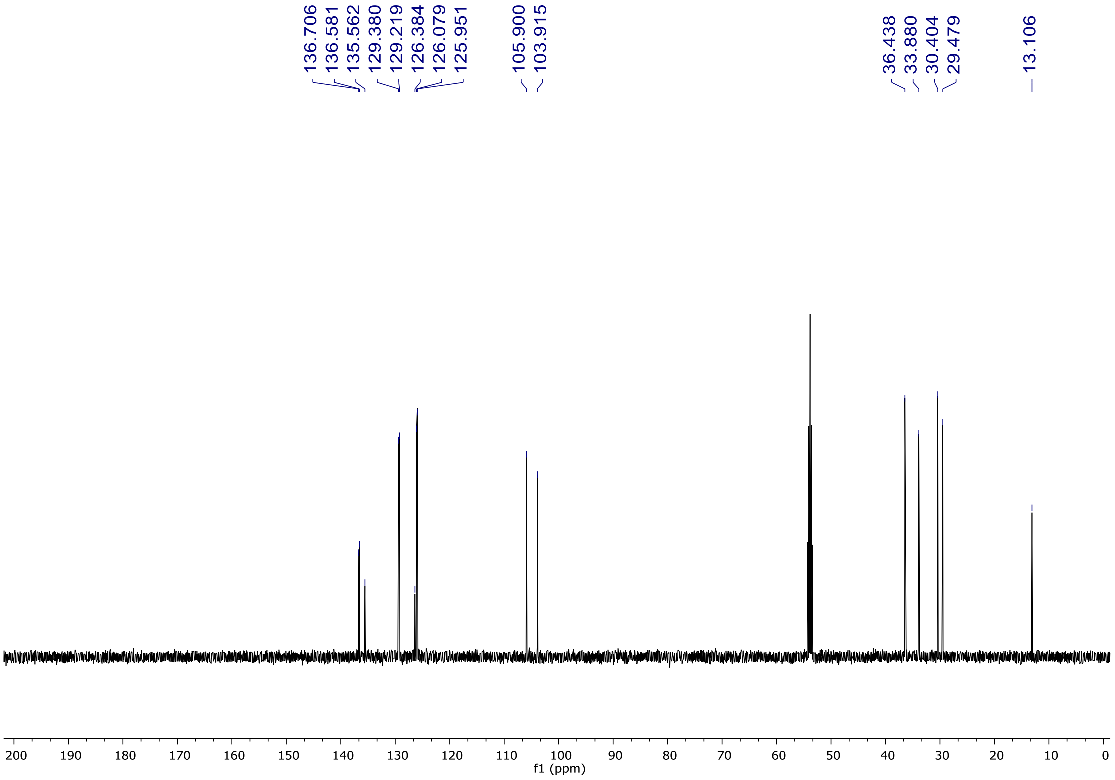

Supplementary Figure 80. ^{1}H NMR and ^{13}C NMR spectra of **3aC**.

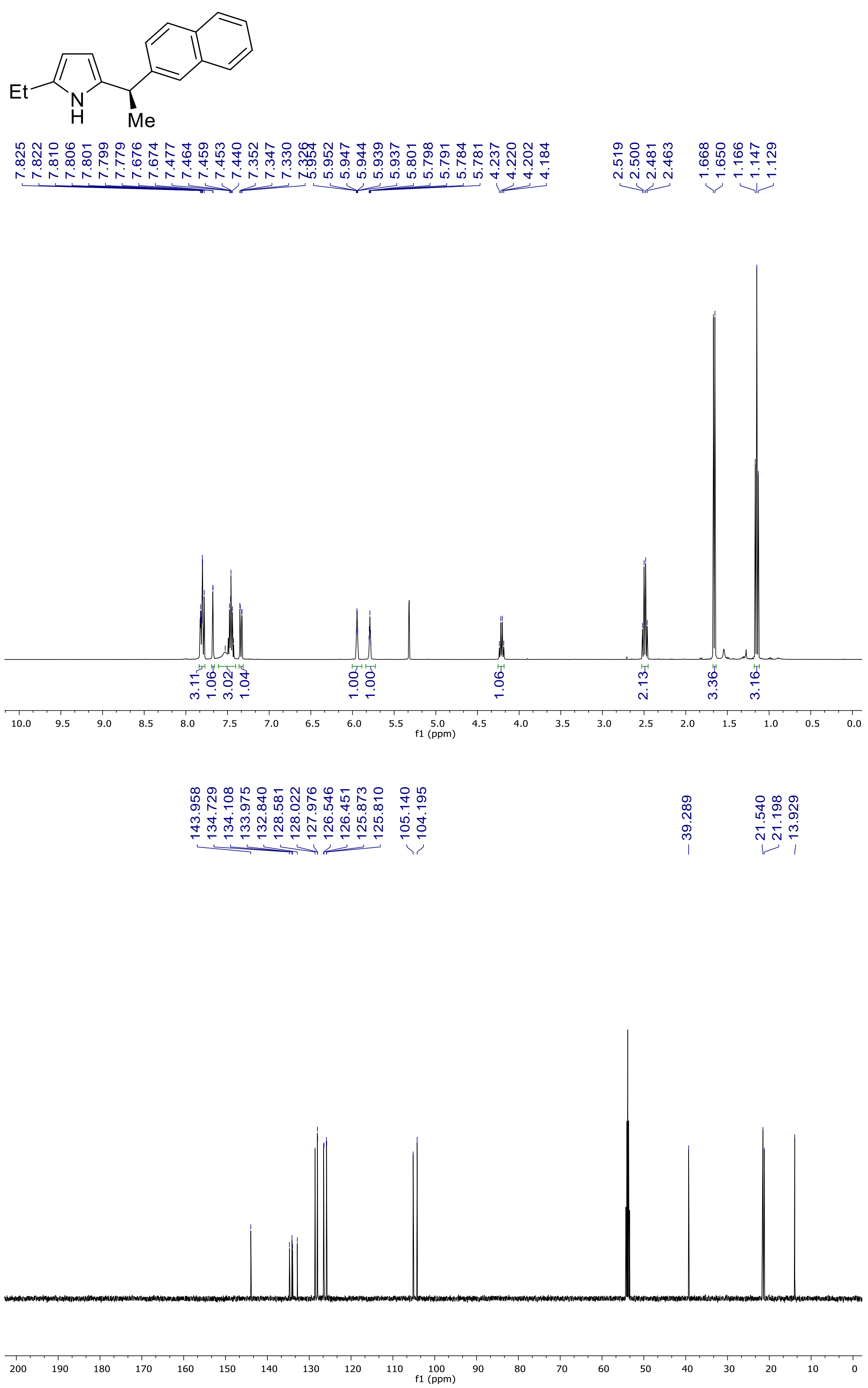

Supplementary Figure 81. ^{1}H NMR and ^{13}C NMR spectra of **3bp**.

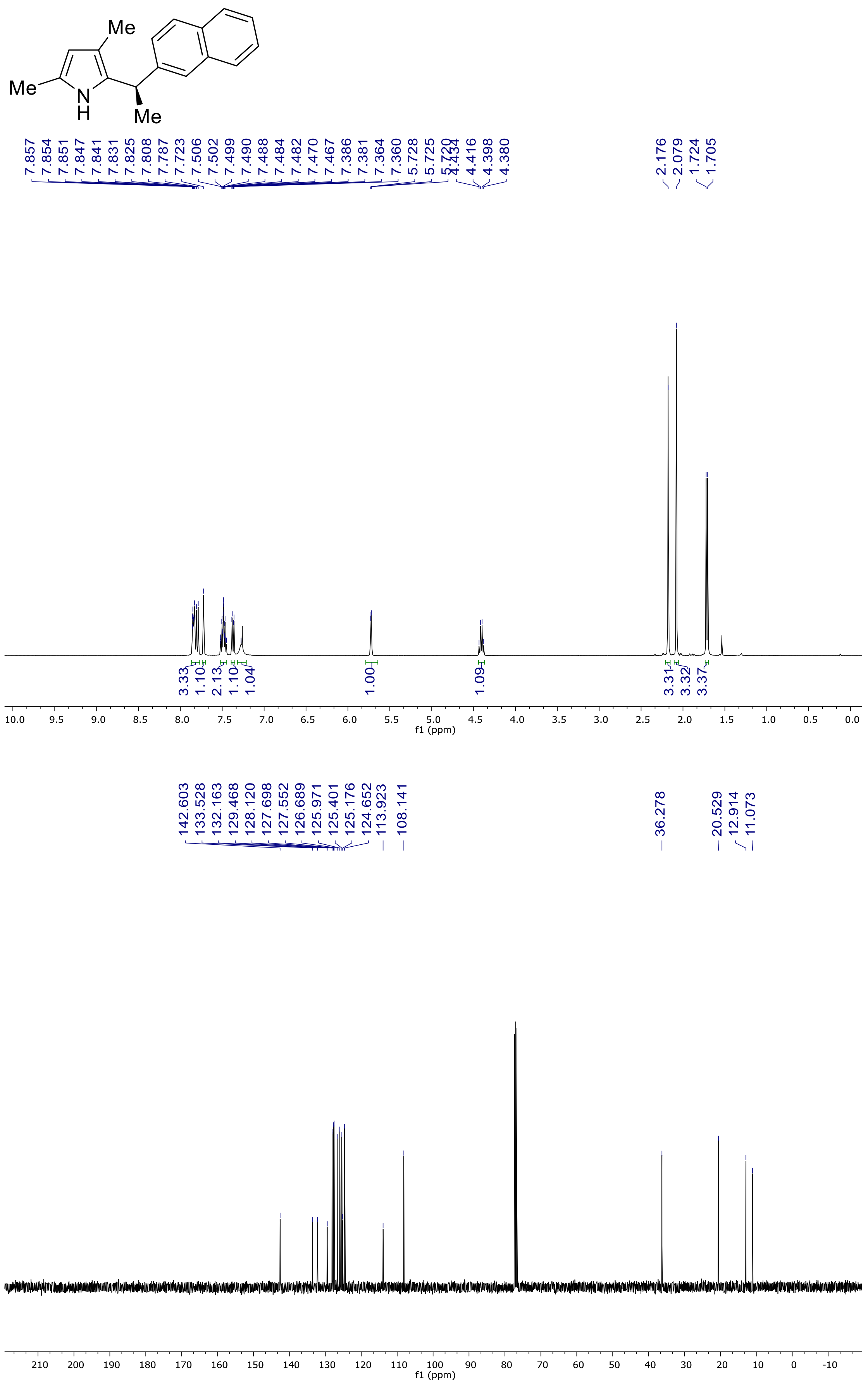

Supplementary Figure 82. ^{1}H NMR and ^{13}C NMR spectra of **3cp**.

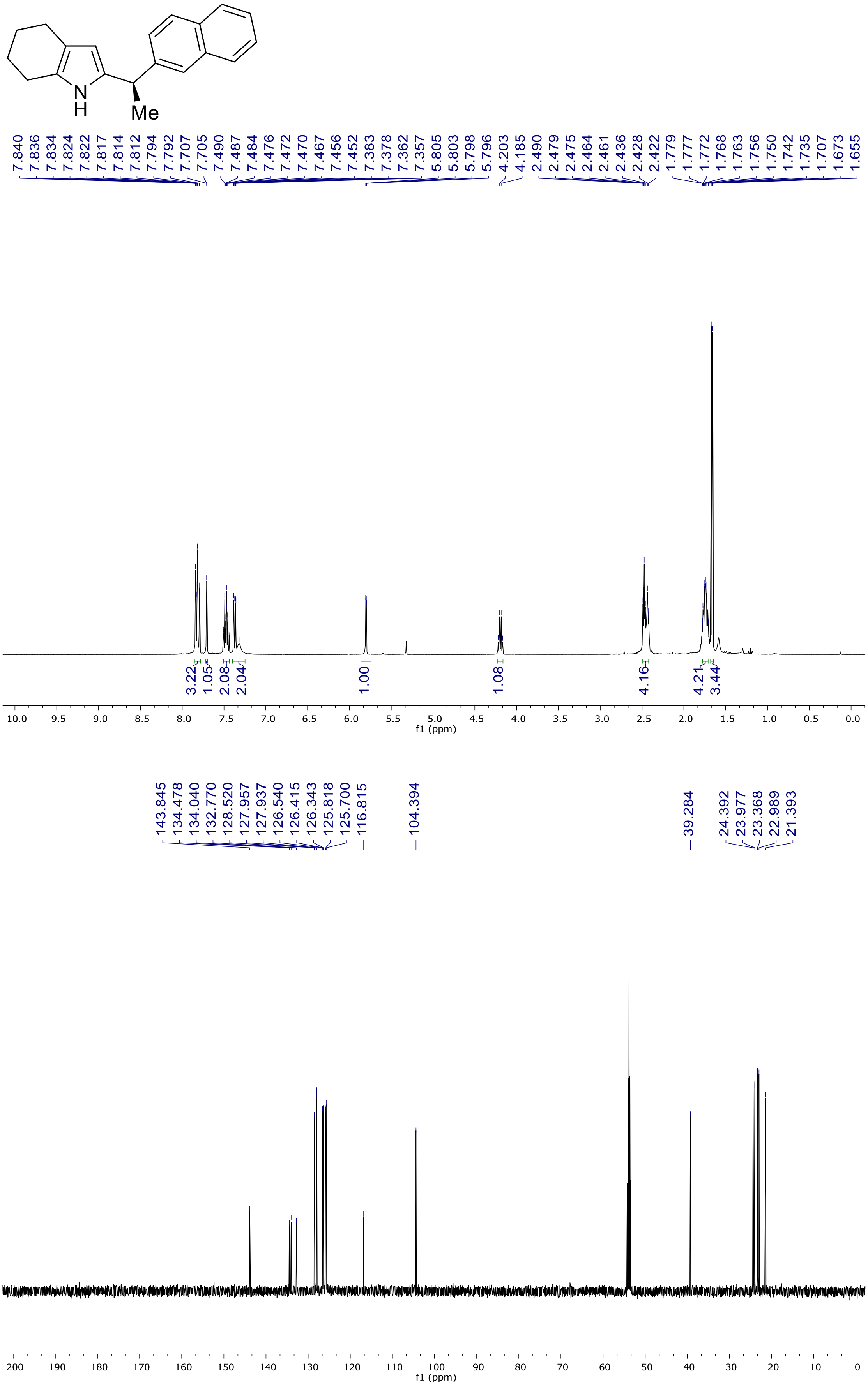

Supplementary Figure 83. ^{1}H NMR and ^{13}C NMR spectra of **3dp**.

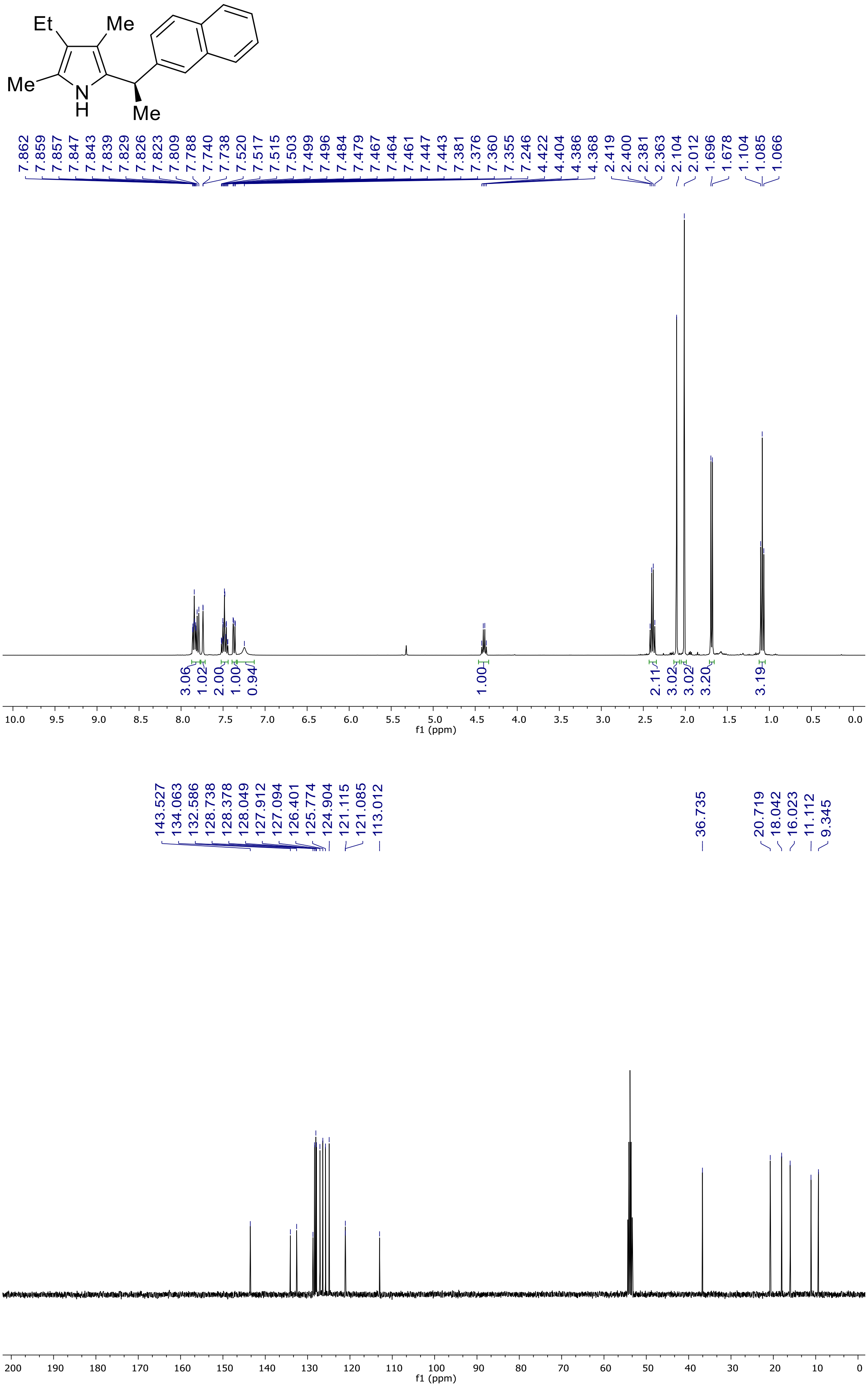

Supplementary Figure 84. ^{1}H NMR and ^{13}C NMR spectra of **3ep**.

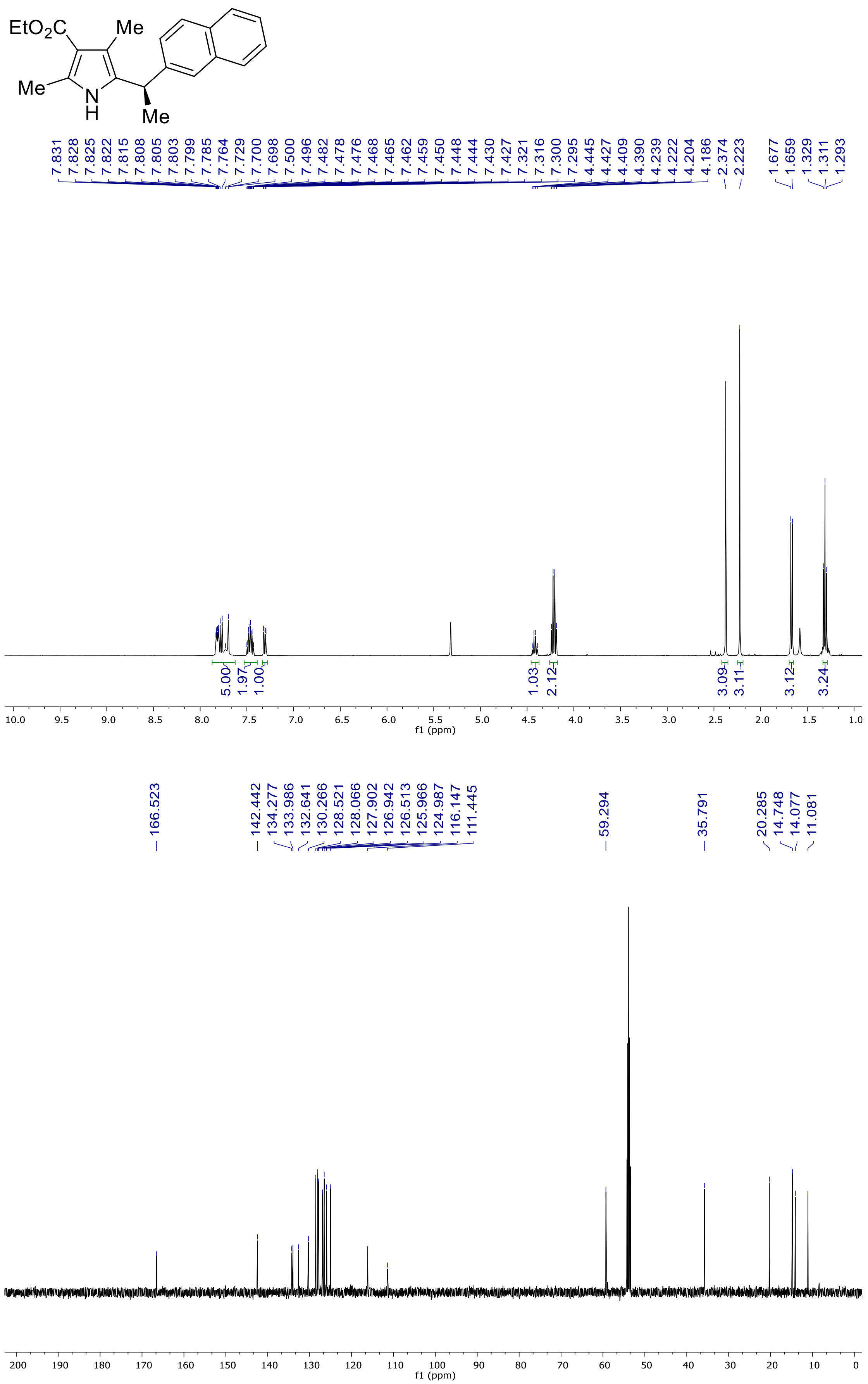

Supplementary Figure 85. ^{1}H NMR and ^{13}C NMR spectra of **3fp**.

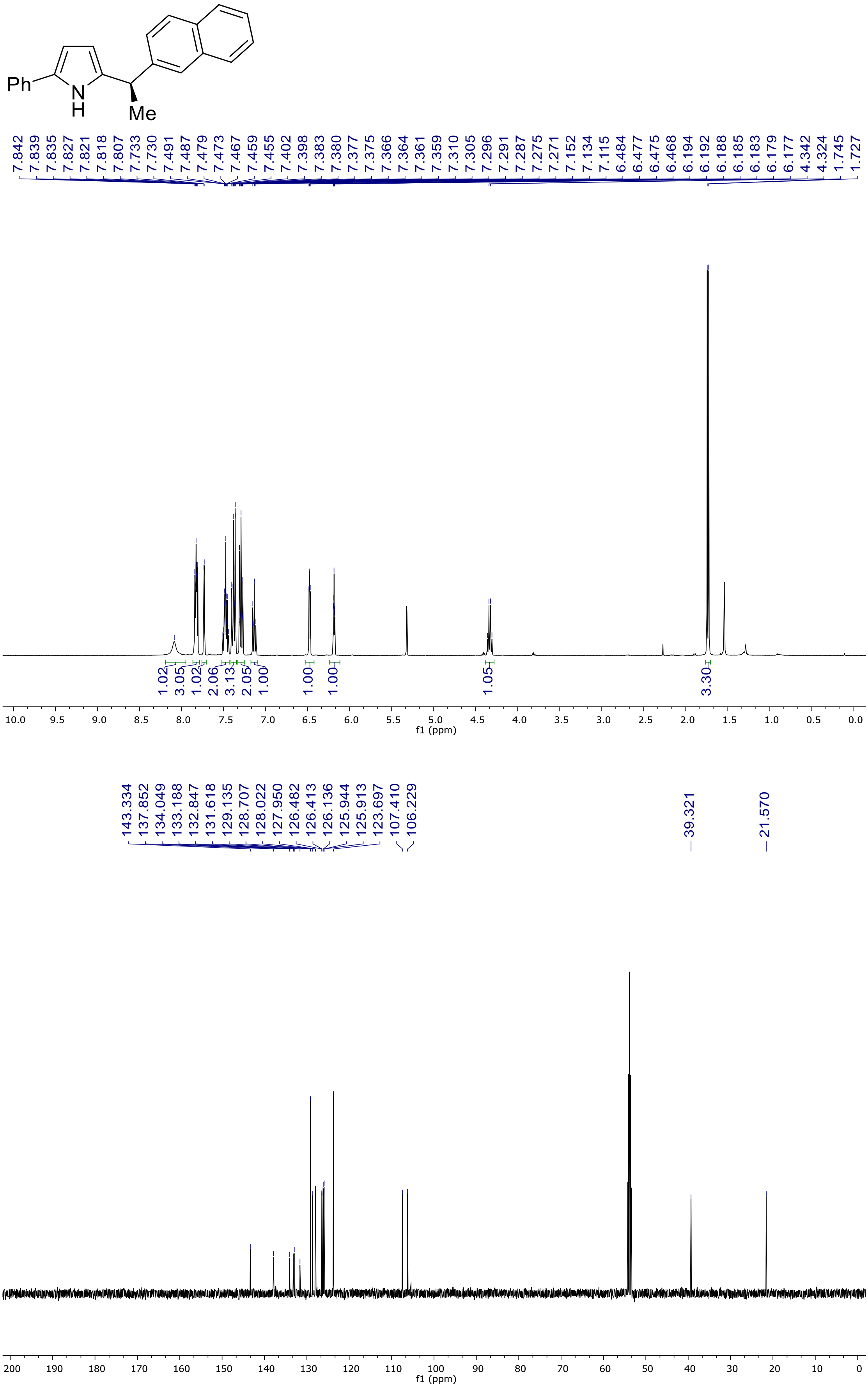

Supplementary Figure 86. ^{1}H NMR and ^{13}C NMR spectra of **3gp**.

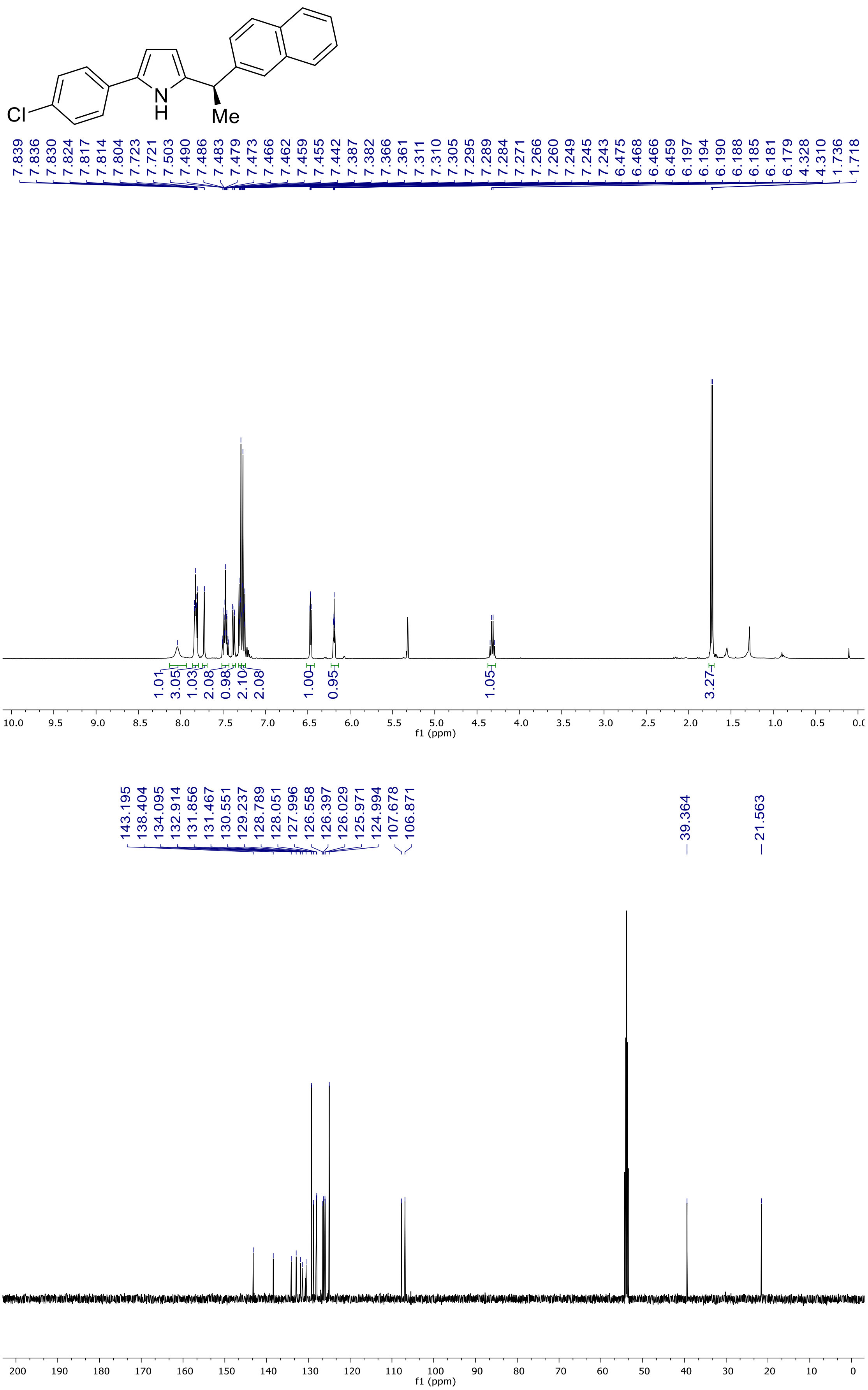

Supplementary Figure 87. ^{1}H NMR and ^{13}C NMR spectra of **3hp**.

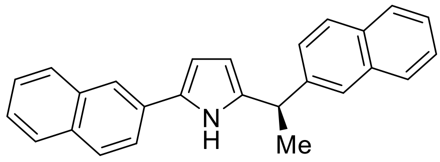

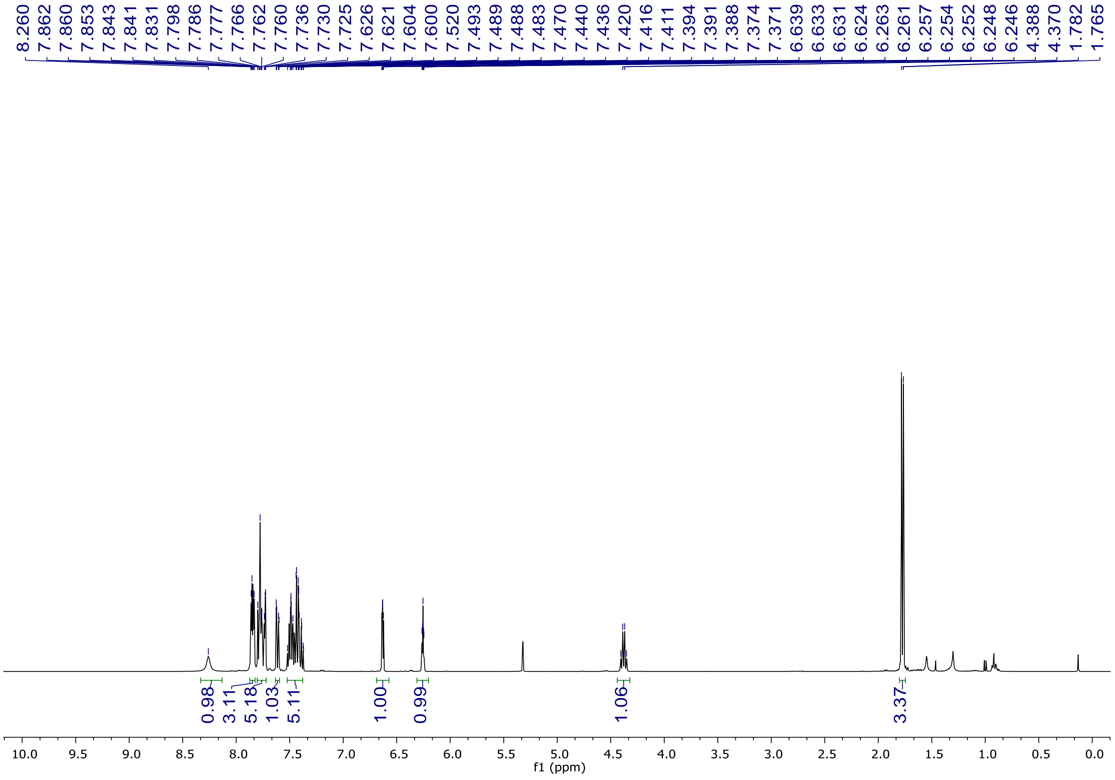

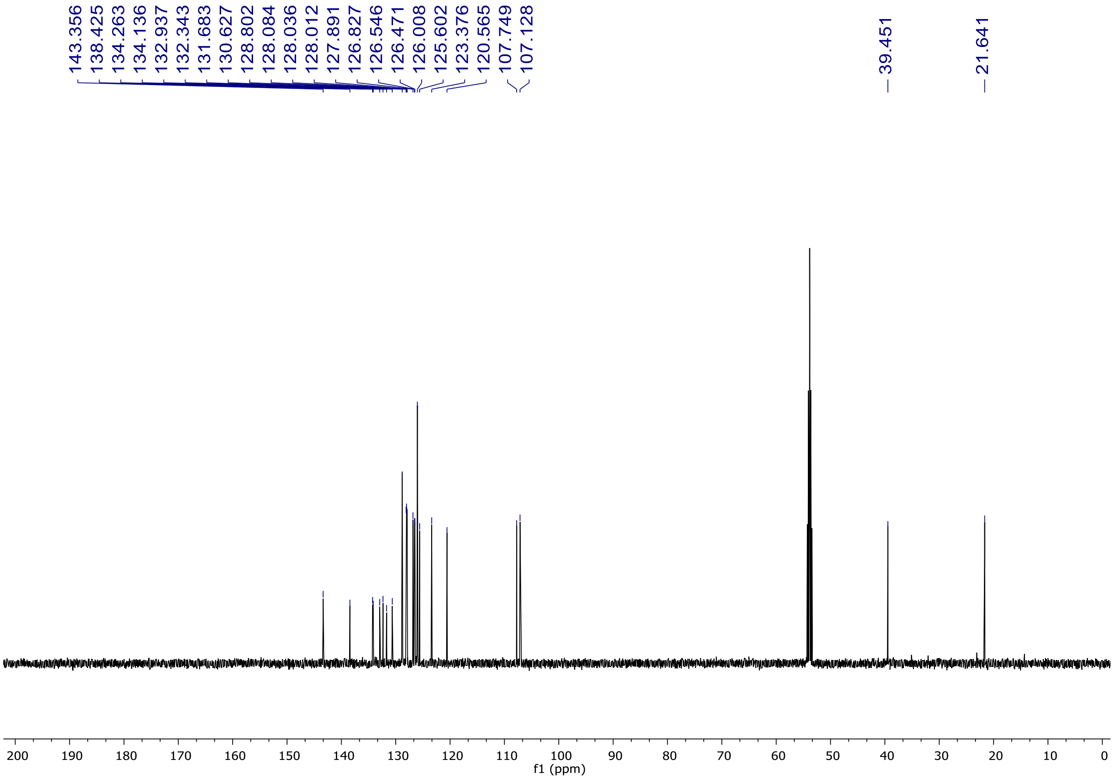

Supplementary Figure 88. ^{1}H NMR and ^{13}C NMR spectra of **3ip**.

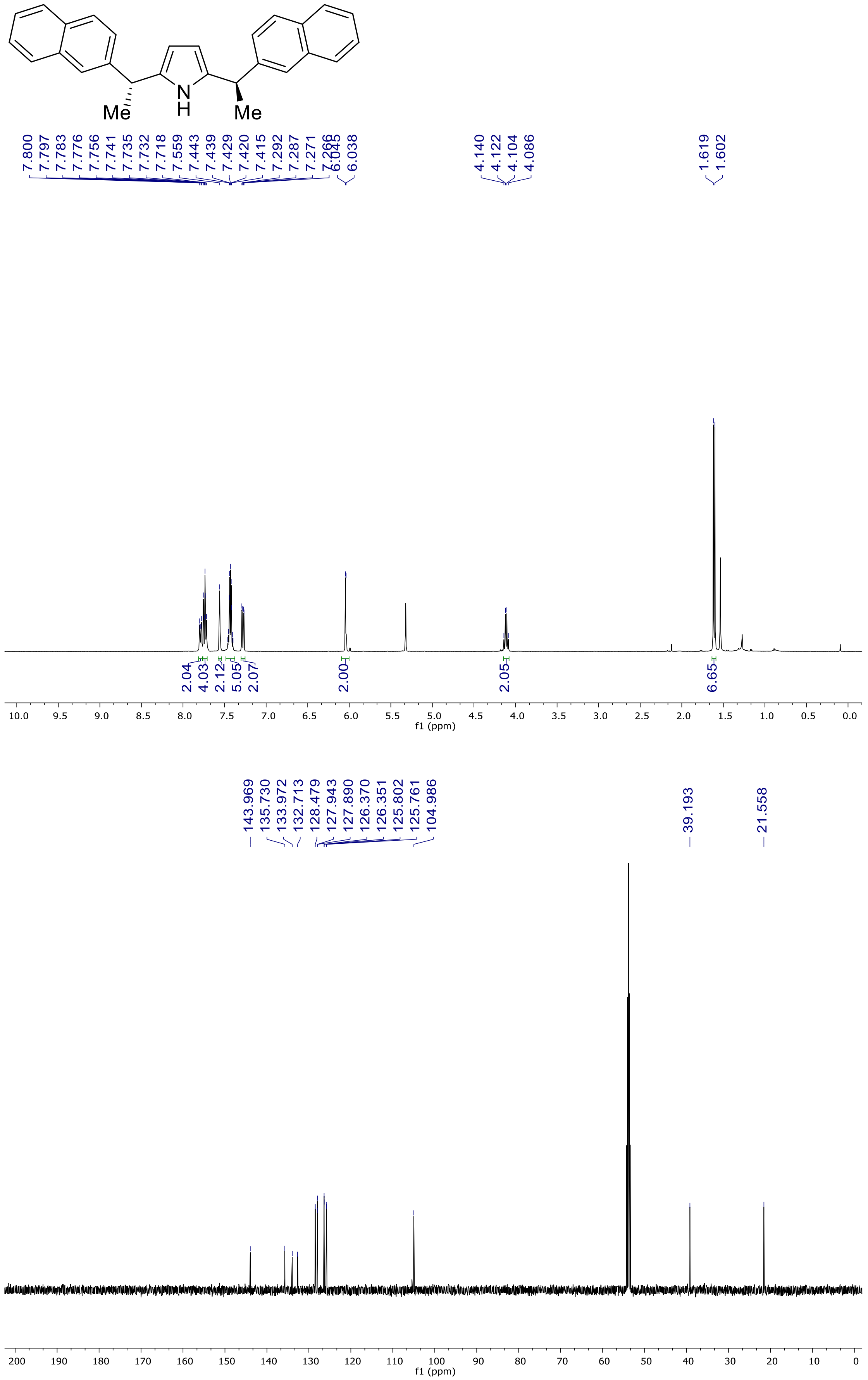

Supplementary Figure 89. ^{1}H NMR and ^{13}C NMR spectra of **3jp**.

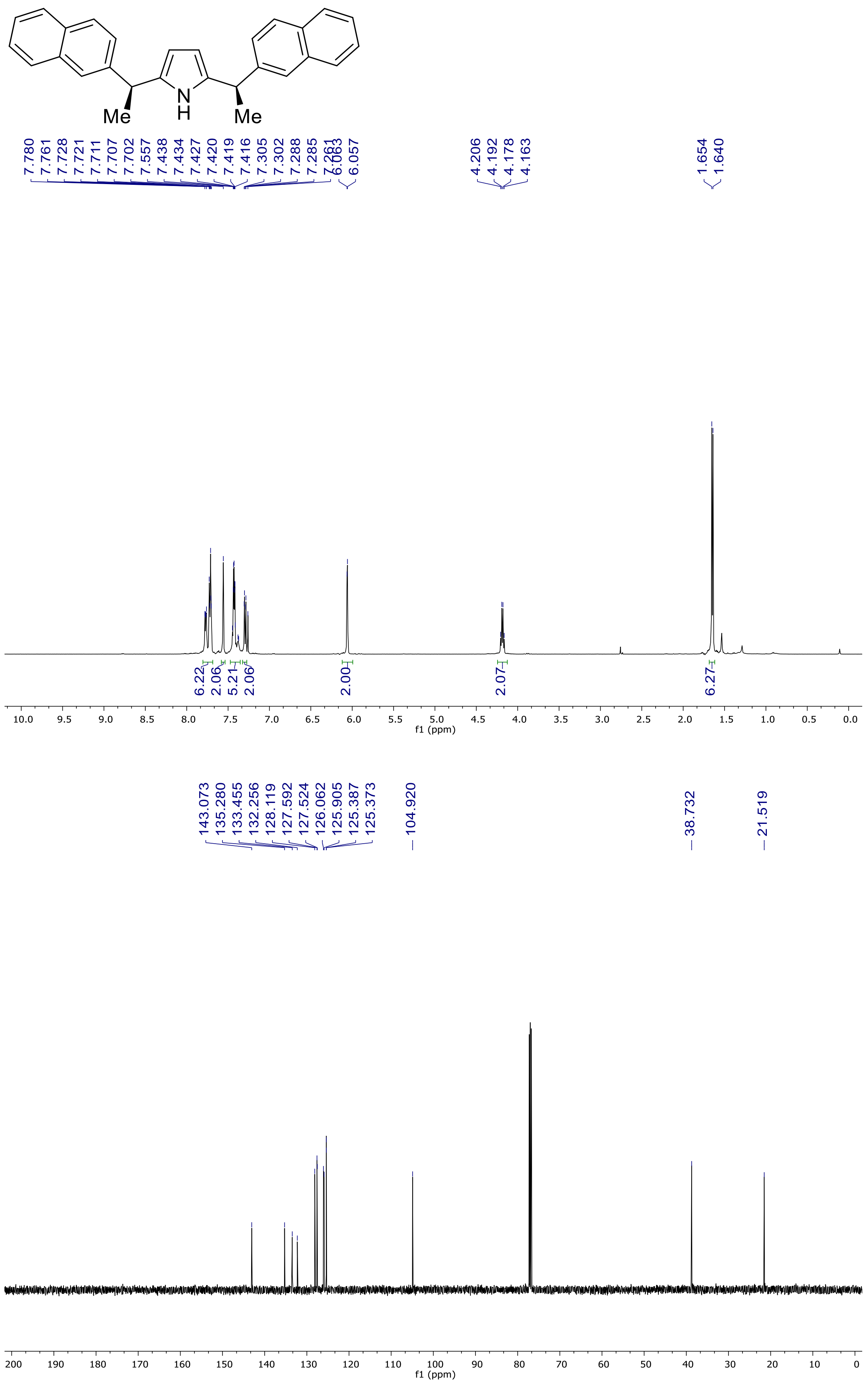

Supplementary Figure 90. ^{1}H NMR and ^{13}C NMR spectra of **3jp′**.

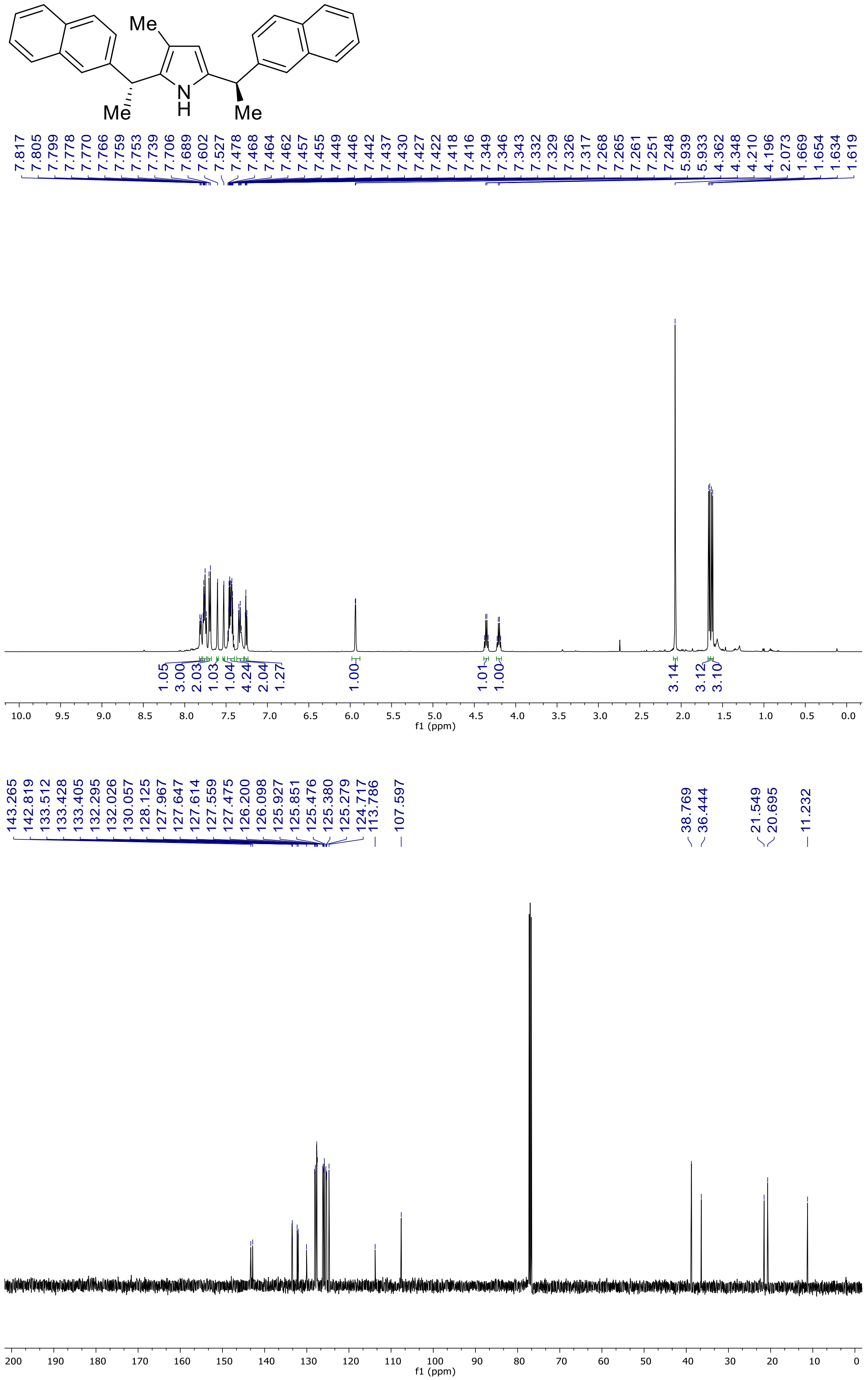

Supplementary Figure 91. ^{1}H NMR and ^{13}C NMR spectra of **3kp**.

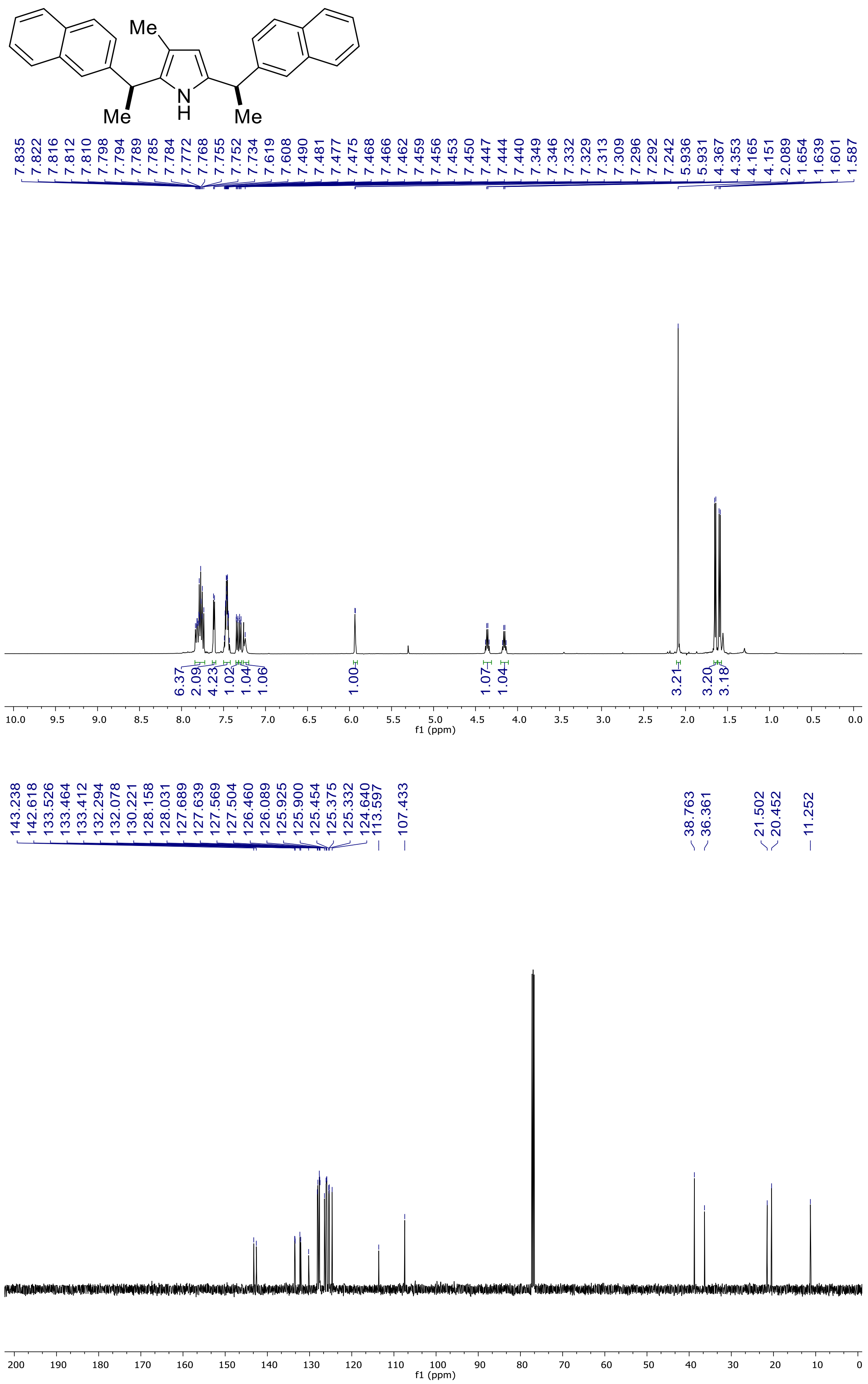

Supplementary Figure 92. ^{1}H NMR and ^{13}C NMR spectra of **3kp′**.

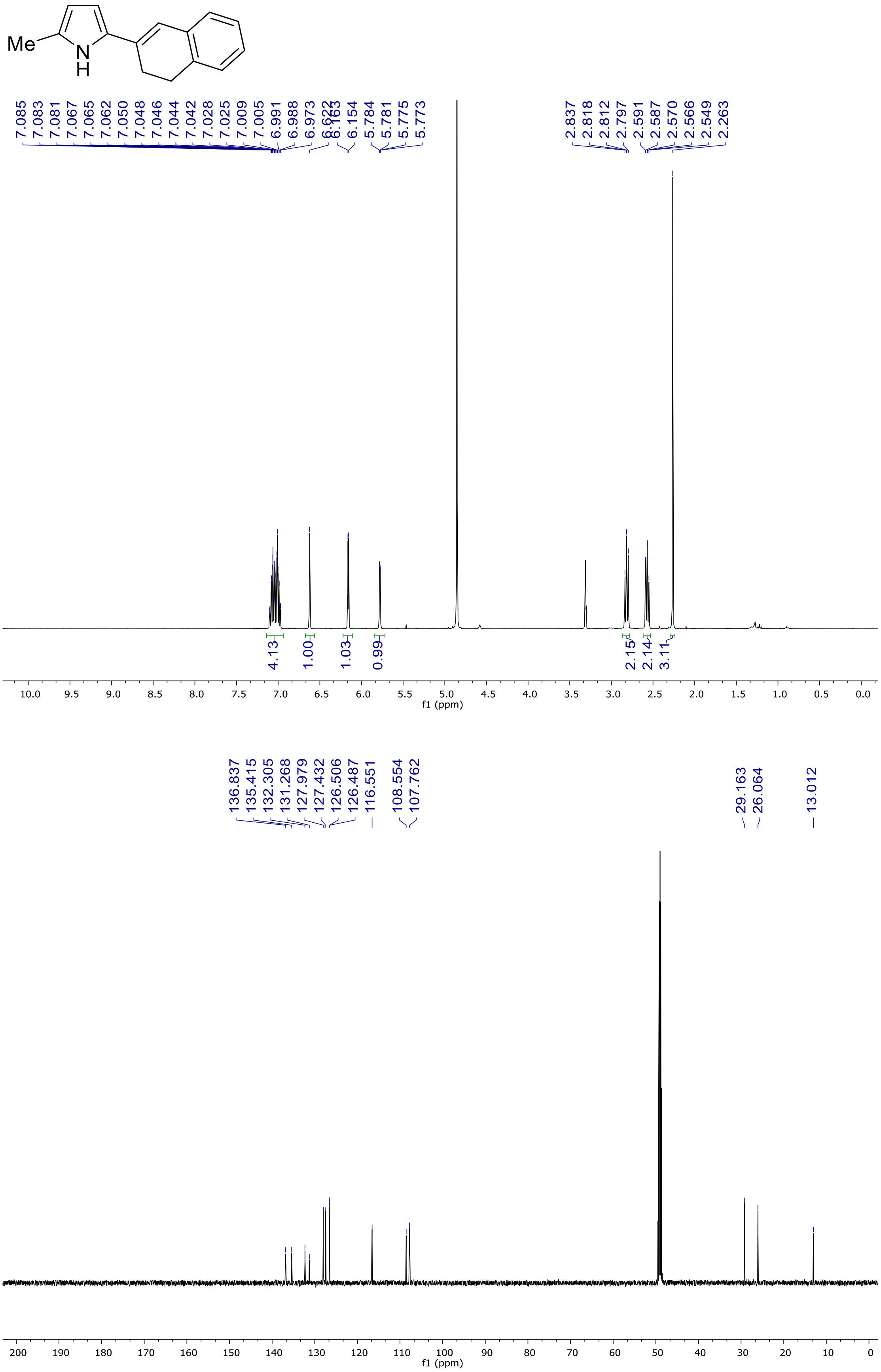

Supplementary Figure 93. ^{1}H NMR and ^{13}C NMR spectra of **7**.

Supplementary References

1. Senadi, G. C., Reddy, M. M., Lu, T.-Y. & Wang, J.-J. Oximes as reusable templates for the synthesis of ureas and carbamates by an in situ generation of carbamoyl oximes. *Green Chem*. **19**, 4272-4277 (2017).
2. Arita, S., Koike, T., Kayaki, Y. & Ikariya, T. Synthesis and reactivities of Cp*Ir Amide and hydride complexes bearing C−N chelate ligands. *Organometallic*s **27**, 2795-2802 (2008).
